# Fetal Zone Steroids Show Discrete Effects on Hyperoxia-Induced Attenuation of Migration in Cultured Oligodendrocyte Progenitor Cells

**DOI:** 10.1155/2022/2606880

**Published:** 2022-05-09

**Authors:** Donna E. Sunny, Elisabeth L. Krüger, Elke Hammer, Uwe Völker, Matthias Heckmann

**Affiliations:** ^1^Department of Neonatology and Pediatric Intensive Care, University Medicine Greifswald, Greifswald 17475, Germany; ^2^Department of Functional Genomics, University Medicine Greifswald, Greifswald 17475, Germany

## Abstract

Cerebral oxygenation disturbances contribute to the pathogenesis of brain lesions in preterm infants with white matter damage. These children are at risk of developing long-term neurodevelopmental disabilities. Preterm birth is associated with sudden hormonal changes along with an untimely increase in oxygen tissue tension. There is a persistent high postnatal production of fetal zone steroids (FZS), which serve in the fetoplacental unit as precursors for placental estrogen synthesis during pregnancy. The role of FZS in events associated with oxygenation differences and their impact on the developing white matter is not well understood. Therefore, we investigated the effect of hyperoxia (80% O_2_) and subsequent administration of FZS on the protein composition and migration capabilities of immature oligodendrocytes using the OLN93 (rat-derived OPC) cell line as an experimental model. We tested the effect of the FZS, dehydroepiandrosterone (DHEA), 16*α*-OH-DHEA, and adiol (5-androstene-3*β*, 17*β*-diol). After 24-hour exposure to hyperoxia, we monitored the changes in the proteome profile following treatment and observed significant alterations in pathways regulating cytoskeletal remodelling, cell migration, and cell survival. Additionally, hyperoxia leads to impaired migration of the OLN93 cells in culture. Administration of the FZS showed positive effects on the migration process under normoxic conditions in general. However, under hyperoxic conditions, the trend was less prominent. The observed effects could be related to changes in levels of cofilin/LIMK pathway-associated proteins. Adiol had a negative effect when administered together with estradiol, and the proteomic data reveal the activation of ephrin receptor signalling that might be responsible for the attenuation of migration. The results suggest that FZS can differentially regulate pathways involved in the migration of OLN93 cells. A deeper insight into the precise role of endogenous FZS would be an essential prerequisite for developing new treatment strategies including supplementation of estradiol and other steroids in preterm infants.

## 1. Introduction

Preterm birth represents a global health problem affecting up to 10% of newborns in the western world [1]. Premature birth is associated with lower volumes of grey and white matter along with changes in the microstructural organization of the brain tissue, leading to a broad spectrum of cognitive and neurological deficits [2], making prematurity the leading cause of neurodevelopmental disability in childhood [3]. White matter constitutes oligodendrocytes in an enormous proportion, and in preterm infants, the occurrence of white matter injury coincides with the period of extensive OL migration and maturation [4]. Oxygen is widely used in neonatal intensive care for resuscitation and treatment of neonatal lung disease [4]. There is increasing evidence that hyperoxia causes oxidative stress and triggers maturation-dependent cell death, maturation arrest of developing oligodendrocytes, and disruption of axon-oligodendrocyte integrity [4, 5]. However, the effects of hyperoxia on the migration capabilities of the immature oligodendrocytes (OLs) and the underlying mechanisms have not been elucidated yet.

During pregnancy, maternal progesterone and estrogen levels increase significantly. Placenta is the main source of estrogen during pregnancy [6], and the fetus provides 90% of the androgenic precursors required for the synthesis of estrogen. The active steroidogenic specialized compartment of the fetal adrenal cortex is called the fetal zone, which together with the placenta forms an effective steroid exchange channel, and this functional association is termed as the “fetoplacental unit” [7]. The fetus is constantly exposed to the changing hormone levels during its crucial stages of development. Estrogen is considered to be important for normal brain development, and several experimental studies have shown that it can act as a potential neuroprotective agent against acute and chronic injuries in the brain [8–10].

However, preterm-born infants experience a drastic drop in estradiol (E2) and progesterone levels within 24 hours along with a dramatic increase of the oxygen tissue tension at a much earlier phase of brain development as compared to infants born at term [9]. This led to the idea of supplementation of these hormones for their neuroprotective properties [11]. However, another important aspect was completely ignored. At birth, after the disruption of the fetoplacental unit, the fetal zone of the preterm infants continues to synthesize steroid precursor molecules, which are collectively called fetal zone steroids (FZS). Dehydroepiandrosterone (DHEA), which is the main precursor molecule for estrogen synthesis, is the major component of FZS. We and others have previously reported that the urinary excretion of FZS in preterm-born infants is significantly higher for a longer period of time and does not approach that of term infants before 40 weeks postmenstrual age [12–14]. Additionally, a persistent higher plasma concentration of DHEA-sulfate (DHEAS) was found in preterm infants from the day of birth until 10 months of age in comparison to term infants [15]. Studies have reported that even though the high DHEAS levels during the 1st week of life may reflect the sudden discontinuation of the placental metabolism of circulating DHEAS into estrogens at birth, it might also partly represent a stress response [16]. Since preterm birth is accompanied by a drop in estrogen level and an increase in the levels of these precursor molecules, the precise role of these precursor molecules in brain development needs to be addressed [7]. Experimental models of steroids and the brain in preterm infants have yet to consider this unique hormone milieu [17]. Moreover, very little is known about the effects of FZS on oligodendrocyte migration. Myelination and remyelination after hyperoxic injury would involve extensive migration of oligodendrocyte precursor cells (OPCs) to axonal tracts over considerable distances [18]. It still needs to be resolved at a molecular level how hyperoxia and the presence of FZS affect this important physiological process that the OLs undergo in order to sustain the complex brain architecture.

We have previously shown that FZS show neuroprotective effects comparable to those of E2, in a hyperoxia-induced cell death model of immature glial cells [19]. We have also demonstrated sex-based differences in the response of OPCs towards oxidative stress and have uncovered the underlying molecular mechanism [20]. Based on this mechanism involving the nucleoporin protein Nup133, nuclear respiratory factor 1 (Nrf1), and the classical estrogen receptor alpha (ER*α*), we have also shown how the FZS have differential effects on the maturation of male- and female-derived OPCs and how these observed differences correlate to concentration differences of these steroids observed in urine samples collected from preterm infants [7].

In the current study, we address the consequences of hyperoxia and subsequent administration of FZS (DHEA, 16*α*-OH-DHEA, and adiol) on the migration capabilities of immature OLs using the OLN93 (rat-derived OPC) cell line as an experimental model. We analysed the proteomic alterations under hyperoxic conditions. The migration capability of the cells following treatment with 80% O_2_ and FZS was tested using a wound-healing assay. Further, we analysed the major proteins of the cofilin/LIMK pathway, implicated in OPC migration using immunoblotting. Thus, we explain how some of the FZS and E2 have a positive effect on the migration of these cells under hyperoxic conditions, but at the same time, the others showed a negative effect. We also explore the consequences of cotreatment of these steroids in combination with E2 and their possible mode of action.

## 2. Materials and Methods

### 2.1. Cell Culture

The OLN93 cell line was obtained from the laboratory of Christiane Richter-Landsberg (Universität Oldenburg, Germany). It is a rat-derived (female), pre-OL adherent cell line derived from spontaneously transformed cells in primary rat brain glial cultures [21]. The cell line was authenticated by the Leibniz Institute DSMZ-German Collection of Microorganisms and Cell Cultures (Braunschweig, Germany). The cells were cultured according to Gerstner et al. [22], in Dulbecco's modified Eagle's medium (DMEM) (with 3.7 g/l NaHCO_3_, 25 mM HEPES, 4.5 g/l D-Glucose, and 4.4 g/l NaCl, Biochrom, Berlin, Germany), supplemented with 10% heat-inactivated fetal calf serum (FCS, Biochrom), 0.01% human serum albumin (HSA, Grifols, Barcelona, Spain), and 1% penicillin-streptomycin solution. Cultures were kept in a 37°C, 5% CO_2_ incubator, and media was exchanged every 2-3 days.

### 2.2. Treatments

For the migration assay, cells were grown in 10% FCS medium. Normoxic control experiments were performed at 21% O_2_, and hyperoxic experiments were performed at 80% O_2_ conditions. Dimethyl sulfoxide (DMSO) was used as the solvent control as the steroids were dissolved in DMSO. Cells were treated with E2 (17*β*-estradiol; Sigma-Aldrich, Taufkirchen, Germany), DHEA (5-andostene-3b-ol-17-one; Sigma-Aldrich), DHEA+E2, 16*α*-OH-DHEA (5-androsten-3*β*, 16*α*-diol-17-one; Steraloids, Newport, RI), 16*α*-OH-DHEA+E2, adiol (5-androsten-3*β*, 17*β*-diol; Steraloids), and adiol+E2. All steroids were added to a final concentration of 100 nM.

### 2.3. Wound-Healing/Migration Assay

For wound-healing assays, cells were seeded in a density of 50,000 cells/well in 200 *μ*l of 10% FCS medium under normoxic conditions for 24 hours in clear bottom 48-well plates. After this time period, a scratch was made through the middle of the well using a 10 *μ*l pipette tip. In order to obtain a straight scratch, a glass cover slip was used as a ruler. Afterwards, the medium containing any loose cells was removed, and 100 *μ*l of respective treatment media was added to each well. At time point 0 hour, images were taken using a 5x objective (Fluorescence microscope DMI4000b, Leica, Wetzlar, Germany) from the middle of the well, plates were placed in 21% or 80% O_2_ for 24-hour treatment, and images were taken again post treatment. All images were then analysed using TScratch software (version: 1.0; RRID: SCR_014282, CSE, Zurich, Switzerland). The program calculates the open wound area, i.e., the percentage of the open image at time point 24 hours compared to the initial time point (0 h). The open wound area after 24 hours is calculated as 100 × (open image area 24 hours)/(open image area 0 hours). The treatments as such were compared amongst each other using one-way repeated measure ANOVA and Tukey's Multiple Comparison Test.

### 2.4. Immunoblot Analysis

Protein extracts from whole cell lysates containing ∼40 *μ*g protein was loaded in each lane of a Mini-Gel module for electrophoresis (Bio-Rad, Munich, Germany). Protein was transferred onto nitrocellulose membrane (Amersham Protran 0.45 *μ*m NC Western Blotting Membrane, GE Healthcare, USA), blocked with 1x blocking buffer (Pierce™ Protein-Free (TBS) Blocking Buffer, Thermo Fisher, Waltham, MA, USA) at room temperature (RT) for 1 h, and incubated in primary antibody at 4°C overnight. GAPDH (Rabbit Anti-GAPDH (D16H11) mAb, Cell Signaling Technology, Danvers, MA, USA) was used as the loading control at a 1 : 1000 dilution. Blots were incubated with the secondary antibody at a 1 : 10000 dilution in blocking buffer for 1 hour at RT. Protein bands were visualized with a SuperSignal™ West Femto Maximum Sensitivity Chemiluminescence Substrate (Thermo Scientific). Densitometric intensities were calculated using Image Lab software (Bio-Rad). Primary antibodies are listed in Table 1.

### 2.5. Preparation of Protein Extracts and Mass Spectrometric Analysis

Data were acquired for five independent biological replicates. Proteins were extracted in 8 M urea/2 M thiourea and subjected to tryptic digestion as described earlier [20]. Desalted peptides were analysed by LC-ESI tandem mass spectrometry on a Q Exactive HF mass spectrometer (Thermo Scientific, Bremen, Germany) in data-independent analysis mode (Supplementary Table S1A). Peptide and protein identifications were carried out using a directDIA algorithm with a RefSeq database limited to *Rattus norvegicus* (03/2019) implemented in Spectronaut (v. 15.6.211220.50606, Biognosys, Schlieren, Switzerland). Quantitative data were extracted by Spectronaut based on MS2 peak areas. Missing values were parsed using an iRT profiling strategy (minimum *Q* − value row selection = 0.001). Only nonidentified precursors were parsed with a *Q* − value > 0.0001. Ion values were parsed when at least 20% of the samples contained high-quality measured values. Peptides were assigned to protein groups, and protein inference was resolved by the automatic workflow implemented in Spectronaut. Only proteins with at least two identified peptides were considered for further analyses. Protein intensities were calculated as MaxLFQ values and median normalized in the software Spectronaut.

### 2.6. Experimental Design and Statistical Analysis

The statistical analysis for Western blot data was performed using the GraphPad Prism 9 software. Data from all bioreplicates (minimum of 3 for each experiment) for each experiment was plotted with the mean value and standard error bars (standard error of mean, SEM). For statistical comparison between two variables, unpaired Student's *t*-test was used, and one-way ANOVA (one-way analysis of variance) with Tukey's/Dunnet's post hoc test was applied to compare between multiple treatment groups. A variance with a *p* value < 0.05 was considered significant. Various levels of significance were depicted as mentioned in the individual figure legends.

In case of proteomics data, comparisons of untreated hyperoxic vs. normoxic samples and steroid-treated samples vs. untreated controls were performed per bioreplicate. Resulting ratios were subjected to analysis by a two-sided paired *t*-test and alterations considered significant at *p* < 0.05. Detailed information is provided in Supplementary Table S1(b). Enrichment analysis for functional categorization was performed in the Ingenuity Pathway Analysis (Qiagen, Hilden, Germany). Activity patterns were inferred from the protein regulation. Provided *z*-scores were considered significant when exceeding |2|.

## 3. Results

### 3.1. Hyperoxia Has a Negative Impact on the Pathways Regulating Cell Migration

**Figure 1:**
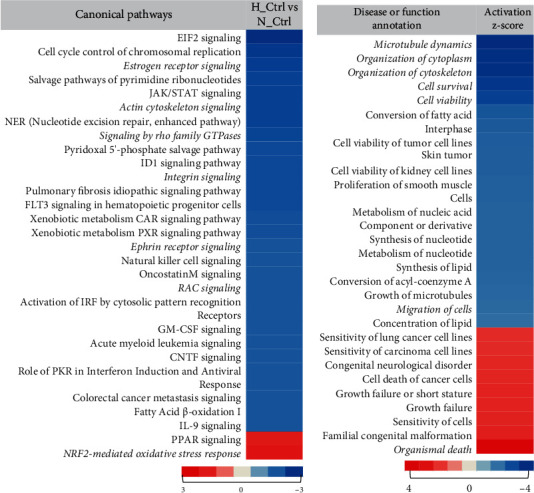
Hyperoxia leads to impairment of migration-related pathways in OLN93 cells. Enrichment of altered proteins (*p* < 0.05) in OLN93 cells post-24-hour 80% O_2_ in comparison to normoxic control treatment in specific functional categories was analysed using IPA: (a) canonical pathways; (b) disease or functions. Heat-map representation of enriched categories are sorted by *z*-score. Negative *z*-scores representing inhibition are indicated in blue, and positive values representing activation are indicated in red. Categories with a *z*-score above 2 or below -2 are only shown. Color code bar represents the maximum and minimum values in accordance to the color. Relevant categories are marked in *italics*. Cutoff *p* value < 0.05 (Fisher's exact test). All results were obtained from the analysis of data of five independent experiments.

Previous reports have shown that hypoxia impairs the migration of OPCs [23]; however, not much is known about the effects of hyperoxia on the migration of OPCs and the molecular signalling involved. Therefore, in order to get a detailed insight into what happens to OPCs under hyperoxic conditions, we treated the OLN93 cells with 80% O_2_ for a period of 24 hours without glucose or serum deprivation. Post treatment, we comparatively analysed the proteome profile of normal and treated cells. In total, 3612 unique protein groups were identified, and 3355 proteins could be successfully assigned to their encoding genes. Comparing the hyperoxia-treated samples to the normoxic control samples, we identified significant (*p* value < 0.05) differences in the ratios for 431 protein candidates. Subsequently, we used the Ingenuity Pathway Analysis (IPA) software to further identify pathways and molecular functions that are affected. Canonical pathway analysis revealed a number of pathways related to cell migration to be altered and differentially regulated upon hyperoxia (Figure 1(a)). In particular, actin cytoskeleton signalling, signalling by Rho family GTPases, ephrin receptor signalling, and Rac signalling were seen to be inactivated. We predict these pathways to be the upstream active effectors that might finally lead to the attenuation of migration under hyperoxic conditions. Furthermore, a strong activation of the Nrf2-mediated oxidative stress response pathway was observed along with RhoGDI signalling. Annotation according to disease function showed a decreased activation of microtubule dynamics, organization of cytoskeleton, and migration of cells along with cell survival. An increased activation was concluded from the protein alterations for organismal death (Figure 1(b)). This indicates the activation of cell death-related signalling pathways, and we anticipate that the cells might redirect their resources and pathways from migration towards survival. Therefore, we hypothesize an impairment of cell migration post hyperoxia.

### 3.2. Hyperoxia Attenuates Migration of OPCs

**Figure 2:**
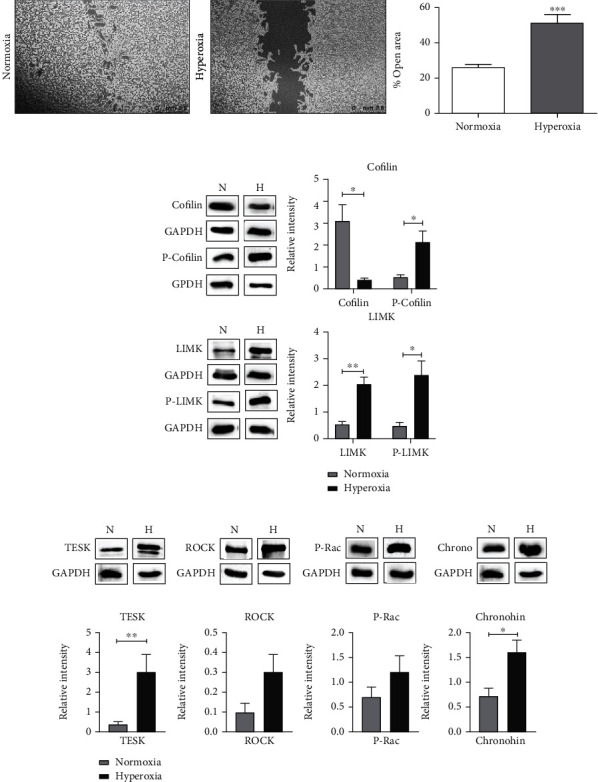
Hyperoxia leads to impairment of migration in OLN93 cells. (a) Representative images of migration (wound healing) assay under normal oxygen conditions (21% O_2_) and post-24-hour 80% O_2_ treatment. Graph represents the combined data from five independent experiments. Scale bar represents 2.5 mm. Western blot analysis of OLN93 cells with (b) cofilin, P-cofilin, LIMK, and P-LIMK; (c) TESK, ROCK, P-Rac, and chronophin antibodies at normal (21% O_2_) conditions and post-24-hour 80% O_2_ treatment. Data is representative of at least three independent experiments. Bars and error represent mean ± SEM of replicate measurements. ^∗^*p* < 0.05, ^∗∗^*p* < 0.01, and ^∗∗∗^*p* < 0.001 (Student's *t*-test).

In view of the proteomics results, we predicted an impaired migration of OPCs under hyperoxic conditions. To test this assumption, we analysed the migration capability of OLN93 cells by means of a classical scratch assay. After 24 hours of hyperoxic treatment, we compared the percentage of the open area in the hyperoxia-treated cells to that in the normoxic control cells. We saw that 24 hours of treatment resulted in significant attenuation of migration. In the normoxic wells, after 24 hours, the scratched area was almost completely occupied by cells that migrated into the scratch/wound area from the surrounding, whereas, in the plates that were kept under 80% O_2_ condition, a significant fraction of the scratched area still remained unoccupied (Figure 2(a)). In order to ensure that this observed occupancy of the wound/scratch area was due to the migration of the surrounding cells and not due to their division, we checked for the doubling time of these cells and determined it to be 32 hours (data not shown) which is more than the treatment time. Additionally, almost complete absence of Ki67 staining after the 24-hour culture time indicated absence of significant numbers of dividing cells at this time point (Figure S1). Therefore, cells in the wound area predominantly represent migratory cells. In order to support the notion from the proteomics profiling that many signalling pathways regulating cell migration were inactivated post hyperoxia, we analysed the levels of downstream effector proteins relevant for cell migration. We focused mainly on the cofilin/LIMK pathway proteins. Actin is the most abundant cytoskeletal component in eukaryotic cells, and the synchronized dynamics between polymerization and depolymerisation of actin filaments and their remodelling is essential for cell division, adhesion, and migration. Cofilin is an actin-binding molecule and plays an essential role in the rapid turnover of actin filaments and actin-based cytoskeletal reorganization [24, 25]. Cofilin stimulates actin filament depolymerisation and severance. Misregulation of cofilin activity and/or expression has been directly linked to cell migration associated with tumour metastasis [26, 27]. The activation of cofilin by direct upstream stimuli is tightly regulated by several mechanisms. The most prominent regulation is achieved by its phosphorylation at Ser3 through LIM kinases (LIMK) and testicular kinases (TESK) [28]. ROCK1 and Rac are upstream effectors of LIMKs in the Rho GTPase signalling pathway, which can indirectly promote the phosphorylation of cofilin [29]. Phosphorylation inhibits the severing of F-actin and induces cytoskeletal rearrangement. In contrast, chronophin, which is a cofilin phosphatase protein, spatiotemporally regulates cofilin activity by activating cofilin, promoting actin-free barbed-end formation, accelerating actin turnover, and enhancing membrane protrusion [28].

After 24 hours of hyperoxic treatment, we saw a significant decrease in the abundance of the cofilin-1 (CFL-1) protein compared to the normoxic control and at the same time a significant increase in the abundance of phospho-cofilin (P-CFL) which shows that hyperoxia triggers inactivation of cofilin (Figure 2(b)). We further looked at LIMK2 and phospho-LIMK and saw that both were significantly more abundant in the hyperoxia-treated cells (Figure 2(c)). TESK was also observed to be significantly high in the treated cells (Figure 2(d)).

Even though we found a significantly higher abundance of chronophin in the cells post treatment, we believe that this is a counterfeedback mechanism to maintain the balance between activation and inactivation of cofilin in view of a very strong inactivation through LIMK (via ROCK and Rac) and TESK.

### 3.3. Fetal Zone Steroids Show Distinct Effects on Hyperoxia-Induced Attenuation of Migration

**Figure 3:**
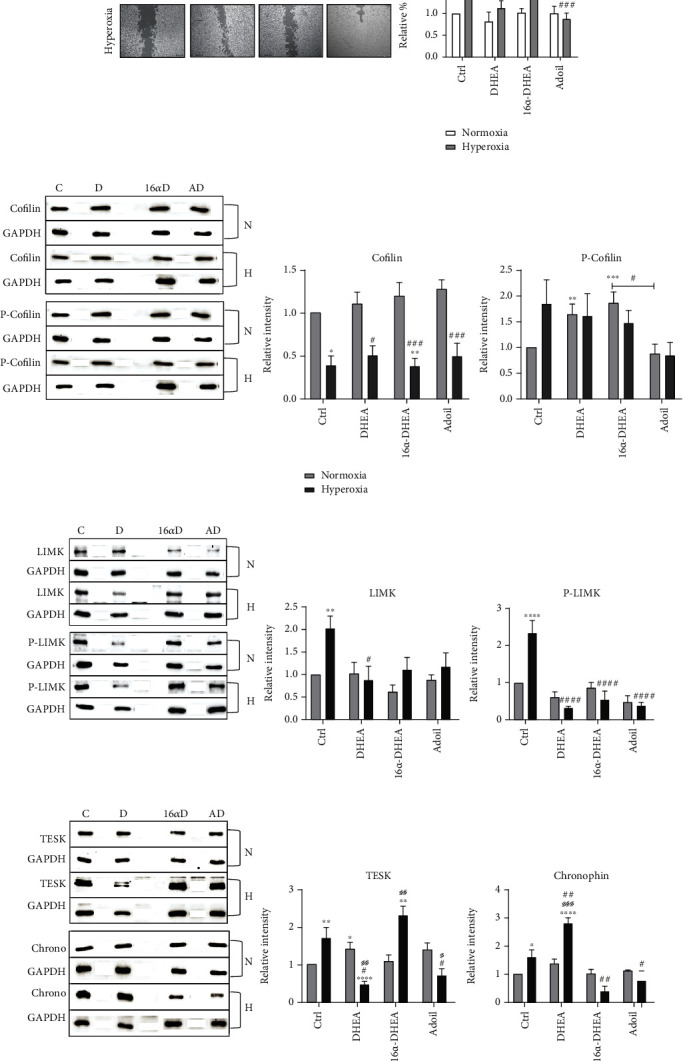
Impact of FZS on the migration of OPCs. (a) Representative images of migration (wound healing) assay under normal oxygen conditions (21% O_2_) and post-24-hour 80% O_2_ with respective steroid treatments are displayed. Graph represents the combined data from five independent experiments. Scale bar represents 2.5 mm. Western blot analysis of OLN93 cells with (b) cofilin and P-cofilin, (c) LIMK and P-LIMK, and (d) TESK and chronophin antibody at normal (21% O_2_) conditions and post-24-hour 80% O_2_ with respective steroid treatments. Data are representative of at least three independent experiments. Bars and error represent mean ± SEM of replicate measurements. Labels indicate statistically significant differences in comparison to normoxic control (∗), in comparison to hyperoxic control (#), and between normoxic and hyperoxic treatments within the same group (§). Single signs represent a *p* value < 0.05, double signs represent *p* < 0.01, triple signs represent *p* < 0.001, and quadruple signs represent *p* < 0.0001 (Student's *t*-test).

To evaluate the impact of steroid hormones on the migration of OPCs, we tested the effect of DHEA, 16*α*-OH-DHEA, and adiol under hyperoxic conditions. In the same migration assay as described above, the cells were treated with the respective steroids before exposure to hyperoxia. After 24 hours, again the percentage of open area was assessed, and the final calculations were performed relative to the normoxic control. Under normoxic conditions, addition of the FZS did not trigger differences in gap closure compared to the control condition. However, the FZS DHEA and adiol showed a significant protective effect under hyperoxic conditions since the influence of these steroids increased migration in comparison to untreated cells (Figure 3(a)).

Looking further at the mechanistic details, treatment with FZS showed overall significant low abundance of cofilin under hyperoxic conditions in comparison to the normoxic conditions. However, P-cofilin levels were even lower in case of the adiol-treated group compared to cells exposed to hyperoxic conditions in the absence of FZS (Figure 3(b)). Compared to hyperoxia-exposed control cells, significantly reduced levels of LIMK were observed in the DHEA-treated cells and a uniform reduction in P-LIMK levels in all steroid treatment groups post hyperoxia (Figure 3(c)). The TESK level was observed to be significantly higher and comparable to the control cells in the 16*α*-OH-DHEA-treated cells post hyperoxia, whereas, in the case of the other steroids, a significant reduction was observed post hyperoxia (Figure 3(d)). Chronophin showed significantly increased levels in the control hyperoxia and DHEA hyperoxic groups, while when treated with the other steroids, it showed a decrease in abundance post hyperoxia (Figure 3(d)).

Combining the observations from the migration assay with the protein abundance profiles, we attempted to decipher the mechanism of action of individual steroids via the cofilin/LIMK pathway. As shown in Table 2, DHEA treatment kept the P-cofilin levels high and decreased the abundance of LIMK, P-LIMK, and TESK under hyperoxia. However, the increased abundance of chronophin indicated a positive regulation on existing free cofilin. This result also points towards a possible inhibition of the upstream Rho GTPase signalling pathway. In case of 16*α*-OH-DHEA, its negative effect on migration was reflected by a comparatively high abundance of P-cofilin, and the increase in abundance of TESK pointed towards a negative regulation of cofilin through TESK as the chronophin level was observed to be low. This was interesting because TESK has been shown to be regulated by the integrin-mediated actin reorganization signalling [30]. Therefore, we see completely different pathways being activated by these two steroids. Adiol, on the other hand, showed a positive effect on migration, but interestingly, all the investigated proteins except cofilin were present in lower abundance in adiol-treated cells post hyperoxia. This indicates that adiol probably achieves the cytoskeletal rearrangement required for efficient migration via some other pathway that still needs to be identified as hyperoxic stress can itself result in altered regulations of the classic pathways.

### 3.4. Adiol Shows an Activation of Actin Cytoskeleton Signalling under Hyperoxic Conditions

**Figure 4:**
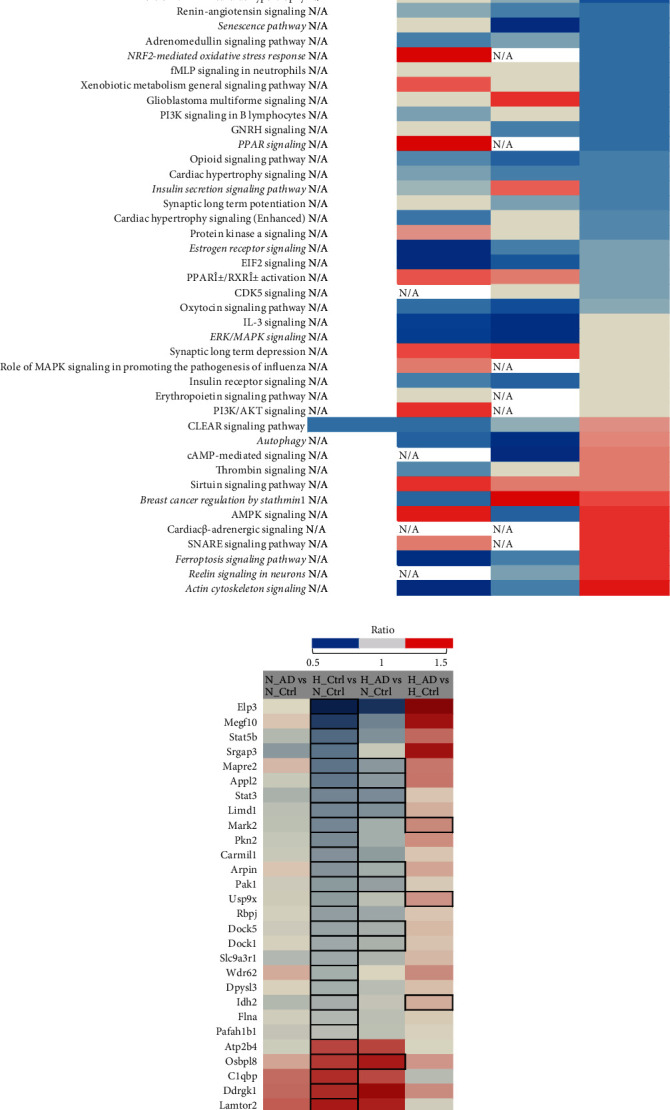
Adiol alters regulation of pathways affected by hyperoxia. (a) Canonical pathway analysis of significantly enriched proteins in the OLN93 cells post different treatment conditions with adiol in comparison to normoxic and hyperoxic controls using IPA. Heat-map representation of enriched pathways sorted by *z*-score. Higher negative *z*-score is indicated in blue, and higher positive *z*-score values are indicated in red. Cutoff *p* value < 0.05 (Fisher's exact test). (b) Heat-map representation of cell migration-related proteins that were dysregulated in the different treatment groups with adiol post-24-hour 80% O_2_ treatment in comparison to normoxic and hyperoxic controls. Protein abundance ratios are depicted with a color scale (downregulated proteins are indicated in blue, intermediate in grey, and upregulated proteins in red). Proteins are sorted according to Gene Ontology (biological process). Dark outlined cells represent the significantly altered proteins (*p* < 0.05) in each group. All data are representative of five independent experiments.

We performed a proteome analysis of the samples obtained after treatment with adiol and cotreatment with adiol and E2 under normoxic and hyperoxic conditions and compared the results to the control samples not treated with hormones. Most of the alterations were specific to one of the conditions, which are illustrated by the low number of overlapping candidates in the Venn diagram (Figure 4(a)). The by-far lowest overlap was observed between specific adiol treatment and cotreatment with E2 indicating that the two conditions elicit largely different responses. We saw more overlap between the groups that were analysed in comparison to the normoxic control than those that were analysed in comparison to the hyperoxic control. Even though we could not detect a significant regulation of cofilin, we detected changes in the upstream regulators of the cofilin/LIMK pathway. IPA analysis of the proteins displaying significantly altered levels identified the pathways differentially regulated by adiol under hyperoxia in comparison to control hyperoxic condition. One of the prominent findings was the inverse activity pattern of the actin cytoskeleton signalling, which was significantly inhibited under hyperoxia compared to normoxia but showed a rather activated pattern upon adiol treatment (Figure 4(b)). A detailed inspection of the proteins involved in cell migration revealed a completely opposite regulation upon adiol treatment post hyperoxia. Whereas the majority of the proteins showed a significant lower ratio in the control hyperoxia-treated group, this trend was reversed upon treatment with adiol (Figure 4(c)). This supports our observation in the functional assay where we saw that treatment with adiol had a positive effect on the migration of cells (Table 2). However, we also saw a significant inactivation of calcium signalling in the adiol-treated cells under hyperoxia, which is the opposite in the case of the control hyperoxia-treated cells (Figure 4(b)). Since calcium signalling is closely associated with actin cytoskeleton signalling, we assume that there are some subtle differential regulations that are brought about by adiol which eventually support cell migration. However, the main drivers of this regulation remain to be identified.

### 3.5. Effect of Treatment with 17*β*-Estradiol and Cotreatment with FZS

**Figure 5:**
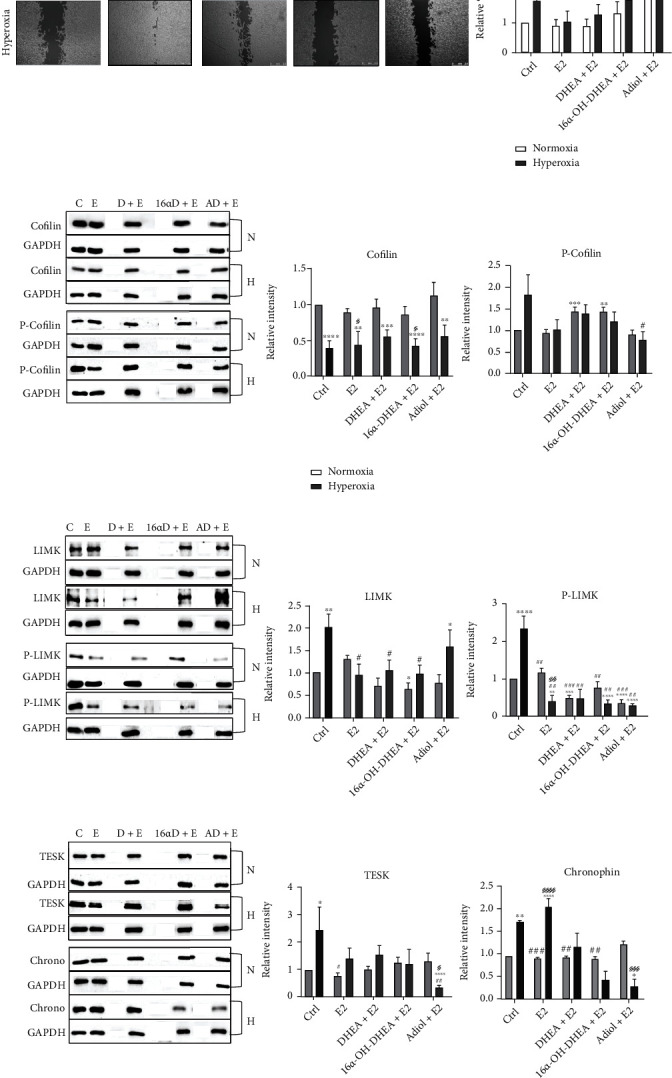
Impact of cotreatment on the migration of OLN93 cells. (a) Representative images of migration (wound healing) assay under normal oxygen conditions (21% O_2_) and post-24-hour 80% O_2_ with respective steroid cotreatments. Graph represents the combined data from five independent experiments. Scale bar represents 2.5 mm. Western blot analysis of OLN93 cells with (b) cofilin and P-cofilin, (c) LIMK and P-LIMK, and (d) TESK and chronophin antibody at normal (21% O_2_) conditions and post-24-hour 80% O_2_ with respective steroid and cotreatments. Data are representative of at least three independent experiments. Bars and error represent mean ± SEM of replicate measurements. Labels indicate statistically significant differences in comparison to normoxic control (∗), in comparison to hyperoxic control (#), and between normoxic and hyperoxic treatments within the same group (§). Single signs represent a *p* value < 0.05, double signs represent *p* < 0.01, triple signs represent *p* < 0.001, and quadruple signs represent *p* < 0.0001 (Student's *t*-test).

Previous studies in cultured oligodendrocytes have shown that 17*β*-estradiol (E2) can cause rapid changes in the structure of the cytoskeleton [24] and that E2 promotes proliferation and migration of glial and neural progenitor cells [31]. Since preterm birth is associated with a drop in estrogen and progesterone levels, it has been advocated that supplementing E2 and progesterone to intrauterine levels can be beneficial for a better neurological outcome. However, preliminary clinical studies in human preterm infants which attempted to do so failed to confirm significantly improved neurological outcomes [32–34]. These studies however did not consider the high amounts of circulating FZS in preterm infants [12]. Here, we treated the OLN93 cells with E2 alone and with E2 in combination with the FZS to monitor the effects of these treatments on the migration of these cells under hyperoxic stress. We saw that E2 had a positive effect, as the observed migration of the cells under hyperoxia was comparable to that under the normoxic control (Figure 4(a)). DHEA+E2 cotreatment showed a similar trend as was seen in case of DHEA treatment alone. Though not significant, this cotreatment showed some positive effect. Similarly, 16*α*-OH-DHEA+E2 also showed the same trend as with 16*α*-OH-DHEA treatment alone with a significant negative effect under hyperoxic conditions. However, the cotreatment of E2 with adiol showed a completely different trend. In contrast to adiol single treatment, cotreatment with E2 showed a significant attenuation of migration even under normoxic conditions (Figure 5(a)). Under hyperoxic condition, the cotreatment again attenuated migration, whereas adiol alone showed a positive effect (Figure 3(a)).

Next, we analysed protein levels and observed that the treatment with E2 brought down the P-cofilin levels comparable to control normoxia (Figure 5(b)). Similarly, it also decreased the abundance of LIMK, P-LIMK, and TESK (Figures 5(c) and 5(d)) and significantly increased chronophin levels under hyperoxia (Figure 5(d)). This makes it evident that E2 acts through the cofilin/LIMK pathway proteins to aid migration of OPCs under hyperoxic stress. In the cotreatment group with DHEA, we saw a slight increase in P-cofilin post hyperoxia but LIMK, P-LIMK, and TESK remained less abundant (Figures 5(b)–5(d)). Chronophin however decreased upon hyperoxia (Figure 5(d)).

In the 16*α*-OH-DHEA cotreatment group, we saw a negative effect on the migration of the cells post hyperoxia which was reflected by the low abundance of cofilin and chronophin and a rather high abundance of P-cofilin and LIMK (Figures 5(a)–5(c)). Here, we see a shift in the regulation as now cofilin activation seemed to be regulated by LIMK as we do not see such a high increase of TESK levels as was observed with the 16*α*-OH-DHEA single treatment (Figures 5(d) and 3(d)). The cotreatment with adiol showed the P-cofilin level comparable to the normoxic control under both conditions (Figure 5(b)). We saw an increased LIMK abundance post hyperoxia. However, P-LIMK remained low under both conditions (Figure 5(c)). TESK and chronophin showed significant low abundance under hyperoxic conditions (Figure 5(d)). As it is difficult to see a clear regulation of cofilin in this cotreatment under both normoxic and hyperoxic conditions, in order to better understand the possible mechanism of action of adiol, we performed a mass spectrometric analysis of the proteins.

### 3.6. Cotreatment of Adiol with E2 Activates Ephrin Receptor Pathway under Normoxic Conditions

**Figure 6:**
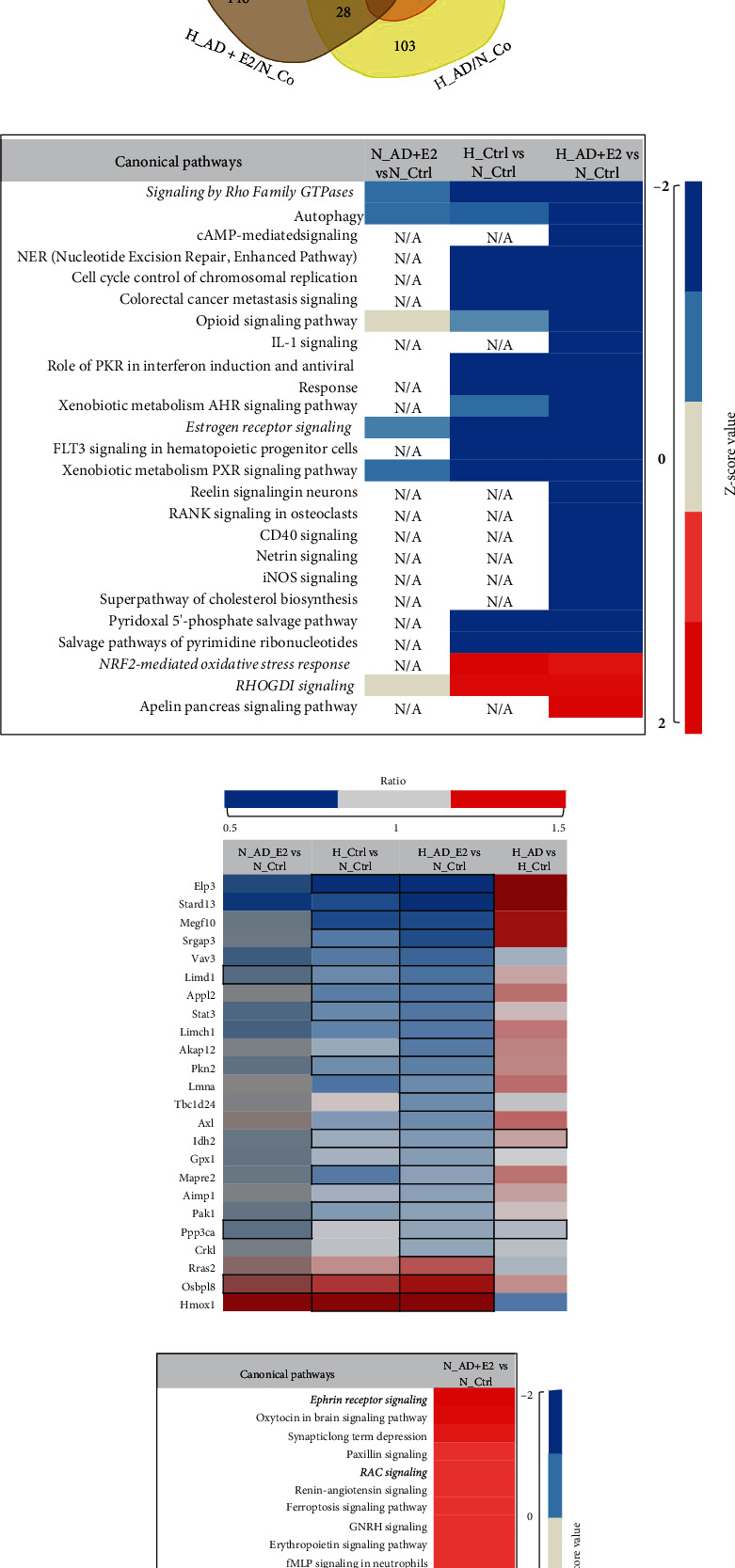
Adiol+E2 shows regulation of different pathways under hyperoxia and normoxia. (a) Venn diagram of significantly altered proteins (normalized to the normoxic control and hyperoxic control as depicted above each bubble) in OLN93 cells post different treatments. Cutoff *p* value < 0.05. Abbreviations: H_Co: hyperoxic control; H_AD: hyperoxic adiol treatment; H_AD+E2: hyperoxic adiol+E2 cotreatment; N_Co: normoxic control. The “/” mark represents “in comparison to.” (b) Canonical pathway analysis of significantly enriched proteins in the OLN93 cells post cotreatment of adiol+E2 in comparison to normoxic and hyperoxic controls using IPA. Heat-map representation of enriched pathways upon adiol+E2 treatment under hyperoxic condition sorted by *z*-score. Higher negative *z*-score is indicated in blue, and higher positive *z*-score values are indicated in red. Cutoff *p* value < 0.05 (Fisher's exact test). No enrichment was obtained in the H_AD+E2 vs. H_Ctrl comparison and therefore has not been depicted. Complete heat map is provided as supplementary figure S3. (c) Heat-map representation of cell migration-related proteins that were dysregulated in the different steroid treatment groups post-24-hour 80% O_2_ treatment in comparison to normoxic and hyperoxic controls. Protein abundance ratios are depicted with a color scale (downregulated proteins are indicated in blue, intermediate in grey, and upregulated proteins in red). Proteins are sorted according to Gene Ontology (biological process). Dark outlined cells represent the significantly altered proteins (*p* < 0.05) in each group. (d) Canonical pathway analysis of significantly enriched proteins in the OLN93 cells post adiol+E2 cotreatment under normoxic condition in comparison to normoxic control using IPA. Heat-map representation of enriched pathways sorted by *z*-score. Pathways with a *z*-score value of >1 or <-1 are shown. Negative and positive *z*-score values are indicated in blue and red, respectively. Cutoff *p* value < 0.05 (Fisher's exact test). All data are representative of five independent experiments.

Cotreatment of adiol with E2 had a negative effect on the migration of OLN93 cells under hyperoxic as well as normoxic conditions even though these steroids showed a positive effect when administered separately (Table 2 and Figure 5). Supporting these findings, IPA analysis results revealed a strong inhibition of the signalling by Rho family GTPases as well as of the estrogen receptor signalling (Figure 6(a)). In contrast, an activation of the RhoGDI signalling was concluded from the protein alterations. RhoGDI signalling inhibits the Rho family GTPase function by preventing nucleotide exchange and membrane association [35]. Reviewing individual proteins involved in migration, we found a similar trend as observed in the hyperoxic control group (Figure 6(b)). In the case of the cotreatment under normoxic conditions, we observed a significant activation of the ephrin receptor pathway while no pathways were detected to be significantly inactivated (Figure 6(c)). The ephrin pathway can influence migration due to the characteristic repulsive effects of the ephrin receptor forward signalling that includes process retraction and inhibition of cell migration [36]. Hence, we assume that the ephrin receptor pathway plays a role in the observed attenuation of migration.

## 4. Discussion

Mature oligodendrocytes (OLs) produce myelin, a lipid-rich structure that envelops central nervous system (CNS) axons. Myelination is achieved by extending many branched processes that wrap around the axon multiple times. But prior to the onset of myelination, newborn OL precursor cells (OPCs) undergo a phase of local proliferation followed by extensive migration to different parts of the brain from their point of origin [23]. Therefore, in a developing brain, proper myelination and remyelination are highly dependent on the capacity of OPCs to migrate to their target destinations and initiate differentiation. All these different stages of OL maturation (proliferation, migration, and differentiation) involve drastic morphological transformations and require extensive cytoskeletal reorganization [24]. It is known that perinatal hyperoxia can disrupt this complex orchestration of events in the developing brain, leading to periventricular white matter damage which is prevalent in preterm infants [37]. Since oxygen can influence the transcription pattern [38] and subsequent signalling that determines the cellular activity at different stages of maturation of OPCs, we wanted to understand the changes that are brought about by hyperoxia in these signalling pathways. To investigate these processes in more detail, we choose a cell culture model using the OLN93 cells as a simplified model that would be best suitable to study the molecular mechanisms in a reproducible manner (compared to primary cells). We treated the cells with an increasing concentration of oxygen and subsequently chose the condition, in which the cells still remained adhered (positive stress response) and at the same time showed molecular (protein) as well as phenotypic alterations (migration). This was achieved at the 80% O_2_ concentration. Moreover, 80% O_2_ has been widely used to induce cell death/disrupt myelin formation in experimental white matter disease, and the effects are well characterized [9, 37, 39]. Furthermore, we selected 24 hours as an appropriate incubation time for the treatment with hormones, as within this time period, we could observe the effects of the treatments without harming the control cells beyond a threshold limit that would otherwise initiate mass cell death.

Meanwhile, the functional assay showed that hyperoxia impaired the migration of the OLN93 cells. Proteomic data showed that in comparison to the normal cells, the hyperoxia-treated cells showed significant alterations in more than 400 proteins. Upon analysing these proteins, we saw that oxygen treatment resulted in a significantly strong activation of the Nrf2-mediated oxidative stress response signalling. Nuclear factor E2-related factor 2 (Nrf2) is a transcription factor which is one of the major transcriptional regulators of the antioxidative stress response. In the nucleus, it binds to antioxidant response elements (ARE), found in the promoter region of a variety of genes encoding for cytoprotective proteins such as NADPH quinone oxidoreductase, heme oxygenase 1, and sulfiredoxin. In line with this knowledge, we observed a 3.15-fold increase of heme oxygenase 1 in our data post hyperoxia (Figure S2). These enzymes in turn can efficiently prevent oxidative stress-associated cell damage and the initiation of apoptosis. Therefore, an activation of this pathway is essential for cell survival under circumstances of increased oxidative stress [40]. On the other hand, we saw a strong significant inactivation of the actin cytoskeleton signalling, signalling by Rho Family GTPases, ephrin receptor signalling, integrin signalling, and Rac signalling. These findings were additionally confirmed by the reduced levels of proteins of these pathways in primary mouse brain-derived oligodendrocytes upon hyperoxia (data not shown). We identified Raf1 which is known to play an essential role as a spatial regulator of Rho downstream signalling during cell migration, by means of its kinase-independent function [41], to be significantly low abundant in the cells post hyperoxia (Figure S2). Similarly, we also found a downregulation of the Cdc42 effector protein 4 (Cdc42ep4) in the hyperoxia-treated cells along with an upregulation of the Pak1 protein (Figure S2). Pak1 phosphorylates LIMK [42] and attenuates migration by inactivating cofilin. This gives us an insight into the further upstream regulators of LIMK and cofilin under hyperoxic stress. Therefore, we see that under oxidative stress conditions, there is a change in the regulation of active pathways in these cells. The cells switch the regulation of the various interconnected pathways to concentrate on stress response and survival by rather switching off other processes like migration.

A number of steroids including estrogen have been shown to influence the migration capability of OPCs. However, in the case of preterm infants, the role of FZS, which are present at significantly high amounts during the early postnatal period, which coincides with the period of maximum oxidative stress and the resulting outcome of this coincidence on brain development, has not been studied much. Our previous efforts have shown that these precursor molecules do have potent steroid activity, they act as neuroprotective agents [19], and they might be capable of regulating multiple pathways leading to specific cellular effects. DHEA and its sulphated form, DHEAS, are considered important neurosteroids as they might protect the neurons from neurotoxic stress [43]. Their antioxidant properties as well as their role in preventing lipid peroxidation have been widely studied [44, 45]. They, along with other androgens, are also known to alter neuronal and glial calcium oscillations, which in turn regulate cell migration and maturation [46]. Other studies have shown concentration-dependent opposing effects of DHEA on cell proliferation and migration in different cell types including cancer cells [47]. This implies that DHEA can show specific effects in specific cell types and that its effect can be influenced by its concentration. Therefore, it is intriguing to know more about the effects of these steroids specifically on the oligodendroglial cells. Currently, we only know that in preterm infants, during the early postnatal period, there is a higher amount of DHEA and other FZS in circulation, but we still lack information on their concentrations in the brain. Apart from DHEA, E2 is well known to exert numerous protective and antioxidant actions in the brain resulting in increased neural function and resilience that promote neuronal survival [48]. Here, we saw that E2 clearly has a positive effect on the migration of OLN93 cells, and its effect was evident through the regulation of cofilin and its effector proteins. We also saw that DHEA showed a positive effect on migration under hyperoxia through upregulation of chronophin. However, upon cotreatment with E2, that effect was not that prominent though the levels of chronophin were similar to the normoxic control, again indicating towards the complexity of cellular regulations that are altered under subtle changes in the cellular environment. On the other hand, 16*α*-OH-DHEA has a negative impact on cell migration, which is driven by TESK and reflected by a higher level of P-cofilin in the presence of reduced LIMK and P-LIMK. This shows the differential regulation of cofilin by these two molecules. However, when we treated the cells with adiol, it did show a positive effect on migration under hyperoxia, but we could not find any clear regulation of cofilin through its investigated effector proteins to explain the observed effect. The proteomic analysis-based view on the molecular background showed that the actin cytoskeleton signalling was slightly activated, and calcium signalling was strongly inhibited. Moreover, we found elongator complex protein 3 (Elp3) to be significantly lower in abundance upon adiol+E2 cotreatment under hyperoxia but showed an inverse trend in the adiol-only treated cells post hyperoxia. Elp3 is a member of the multisubunit histone acetyltransferase elongator complex. It is known to acetylate *α*-tubulin and interact with microtubules and is involved in the migration and branching of projection neurons during corticogenesis [49]. Since these two signalling pathways are closely connected, and not much is known about the functions of Elp3 in oligodendrocytes, we are looking forward to finding the clues that might link these observations in the future.

### 4.1. Limitations and Translational Aspects

Our results on the effects of hyperoxia are particularly relevant with respect to the clinical settings, as preterm infants are untimely exposed to a several-fold increase in oxygen tension after birth (arterial oxygen tension of 65–80 mmHg) in comparison to in utero conditions (PaO_2_ at 24–28 mmHg), even without supplemental oxygen [50]. However, after birth, most preterm babies are treated with supplemental oxygen (O_2_) therapy. This exposure to a high concentration of O_2_ is known to be the key pathogenic factor of morbidities like chronic lung disease, brain injury including PVL, and retinopathy of prematurity [51]. Therefore, oxygen saturation targets in preterm infants are critical to reduce these morbidities and improve outcome [52]. Furthermore, oxygen supplementation in human preterm infants ranges from hours to months, and the extent of oxygen supplementation is conversely associated with gestational age as are the incidences of oxygen-related neonatal morbidities [53]. However, owing to the limitations of a cell culture model and reasons already mentioned, here, we have only tested 80% O_2_ for a duration of 24 hours.

Due to the rapid decrease (up to 100-fold) in estrogen and progesterone plasma concentrations after preterm birth, supplementation of these hormones was tested in clinical trials with no significant benefit [11]. These studies, however, did not consider the continuing postnatal synthesis of fetal zone steroids by the adrenal of the preterm infant. Our current and previous findings [7, 19] have elaborated the neuroprotective effects of FZS and either no or even harmful effects when FZS are combined with estradiol. Therefore, here, we add new insights to clarify the question whether maintaining the placental hormone level after preterm birth is a promising therapeutic strategy, and our approach considers the unique hormonal milieu of the preterm infant with high postnatal production of FZS.

However, we still lack a clear picture regarding the actual steroid concentration differences in the brain of preterm infants. It is important from a clinical point of view to gather further knowledge about the brain-specific concentration profile of these FZS as well as gonadal steroids in preterm infants. In this study, we have used a simple cell culture model to decipher the molecular mechanisms in a particular brain cell type. However, in an *in vivo* system, these mechanisms might be altered; in particular, the effects of the steroids might be altered due to circulatory factors and possible enzymatic interconversions as wells as extensive regulations via interactions between the various cell types.

## 5. Conclusions

In the past, researchers have endeavored to dissect the extracellular cues and the intracellular machinery governing OPC migration. However, these mechanisms are tightly regulated, and they are capable of responding differentially according to the eminent cellular cues. This is probably achieved by crosslinking these pathways where a number of effector molecules, regulated by distinct upstream signalling, can bring about the same change in their downstream target leading to enhanced or suppressed cellular effect. Here, we have shown how hyperoxia leads to impairment of migration in the OLN93 cells by inactivating all the major pathways regulating cytoskeletal reorganization like actin cytoskeletal signalling, signalling by Rho family GTPases, RAC signalling, integrin signalling, and ephrin receptor signalling.

The most important finding, however, is regarding the effect of FZS in this context. There are studies in other glial cell types like astrocytes where it has been shown that astrocytes of the developing brain are permanently differentiated by the neonatal hormonal milieu, resulting in sexually dimorphic cell morphology, synaptic patterning, and density in males and females [54]. However, not much is known about such effects in the oligodendroglial lineage cells. Our results suggest that FZS can differentially regulate the pathways involved in the migration of OLN93 cells. According to the present results, we see that cotreatments bring about a change in the trend and activate completely different pathways. This makes it important to assess the intersteroid concentration differences in the brain which might give us more clues about how they would affect the different cell types when present all together under normal as well as oxidative stress conditions. In particular, the effect of interaction between FZS and gonadal/sex steroids like E2 must be further explored when considering treatment strategies like supplementation of estradiol and other steroids in preterm infants.

## Figures and Tables

**Table 1:** List of antibodies.

Antibody	Manufacturer	Dilution
GAPDH (D16H11) XP® rabbit mAb	Cell Signaling Technology	1 : 1000
Cofilin (D3F9) XP® rabbit mAb 5175	Cell Signaling Technology	1 : 1000
Phospho-cofilin (Ser3) (77G2) Rabbit mAb 3313	Cell Signaling Technology	1 : 1000
LIMK2 (8C11) rabbit mAb 3845	Cell Signaling Technology	1 : 1000
TESK1 (D49D4) rabbit mAb 4655	Cell Signaling Technology	1 : 1000
ROCK1 (C8F7) rabbit mAb 4035	Cell Signaling Technology	1 : 1000
Phospho-LIMK1 (Thr508)/LIMK2 (Thr505) antibody	Cell Signaling Technology	1 : 1000
Phospho-Rac1/cdc42 (Ser71) antibody 2461	Cell Signaling Technology	1 : 1000
Antirabbit IgG, HRP-linked antibody	Cell Signaling Technology	1 : 10000
Antimouse IgG POD	Boehringer Mannheim	1 : 10000

**Table 2:** Impact of steroids on OPC migration and protein levels upon hyperoxia.

	Control	DHEA	16*α*-OH-DHEA	Adiol
Migration	↓-	↑+	↓-	↑+
Cofilin	↓-	↑(+)	n.a.	↑(+)
P-cofilin	↑+	n.a.	n.a.	↓-
LIMK	↑+	↓-	↓-	↓-
P-LIMK	↑+	↓-	↓-	↓-
TESK	↑+	↓-	↑+	↓-
Chronophin	↑+	↑+	↓-	↓-

Hyperoxic control has been considered as the baseline, and the effects of the steroids on migration and protein levels under hyperoxic conditions have been interpreted accordingly. Furthermore, the effect of hyperoxia compared to the normoxic control is indicated. The abbreviation “n.a.” represents “not altered” and “(+)” represents a trend towards increase.

## Data Availability

The mass spectrometry proteomics data used to support the findings of this study has been deposited in the ProteomeXchange Consortium via the PRIDE partner repository [55] (http://www.ebi.ac.uk/pride+) with the dataset identifier PXD031878. A part of the supporting data has also been included within the supplementary information files.

## Supplementary Materials

Supplementary Materials Figure S1 (supplementary figure 1): Ki67 staining of OLN93 cells post-24 hours of normoxic and hyperoxic (80% O_2_) treatments. Representative immunofluorescence images of OLN93 cells stained for Ki67 proliferation marker. Upper panel represents images taken post-24 hours of normoxic treatment. Lower panel represents images taken post-24 hours of hyperoxic treatment. Scale bar represents 75 *μ*m. Data are representative of three individual experiments. Figure S2 (supplementary figure 2): changes in specific migration-related proteins post treatments. Intensities of (a) Hmox1, (b) PAK1, (c) RAF1, and (d) Cdc42ep4 plotted from the mass spectrometry results. Graphs show the changes in protein intensities upon different treatment conditions. Data are representative of five independent experiments. Bars and error represent mean ± SEM of replicate measurements. ∗ represents statistically significant differences in comparison to normoxic control, # represents statistically significant differences in comparison to hyperoxic control, and § represents statistically significant differences between normoxic and hyperoxic treatments within the same group. Single signs represent a *p* value < 0.05, double signs represent *p* < 0.01, triple signs represent *p* < 0.001, and quadruple signs represent *p* < 0.0001(Student's *t*-test). Figure S3 (supplementary figure 3): complete heat map of canonical pathway analysis of significantly enriched proteins in the OLN93 cells post cotreatment of adiol+E2 in comparison to normoxic and hyperoxic controls using IPA. Negative *z*-score values are indicated in blue, and positive *z*-score values are indicated in red. Cutoff *p* value < 0.05 (Fisher's exact test). Table T1 (supplementary table 1): details of mass spectrometry procedure. (a) LC-MS/MS parameter (data independent mode; quantitative data). (b) Spectronaut parameters for peptide/protein identification and intensity extraction. Table T2 (supplementary table 2): functional categorization of proteins with differential abundance under different treatment conditions with adiol (AD) and AD+estradiol (E2) in comparison to normoxic (N) and hyperoxic (H) controls and prediction of activity of “canonical pathways.” Proteins with *p* < 0.05 were considered. Categories are sorted by ascending activation score *z*. Table T3 (supplementary table 3): functional categorization of proteins with differential abundance under normoxic and hyperoxic conditions and prediction of activity of “diseases and functions.” Proteins with *p* < 0.05 were considered. Categories are sorted by ascending activation score *z*.
