# Supplementary material for: Fetal Zone Steroids Show Discrete Effects on Hyperoxia-Induced Attenuation of Migration in Cultured Oligodendrocyte Progenitor Cells
Source: Oxid Med Cell Longev. 2022 May 9;2022:2606880. doi: 10.1155/2022/2606880 (PMC9110221; doi:10.1155/2022/2606880)
Supplement: Supplementary Materials — Figure S1 (supplementary figure 1): Ki67 staining of OLN93 cells post-24 hours of normoxic and hyperoxic (80% O2) treatments. Representative immunofluorescence images of OLN93 cells stained for Ki67 proliferation marker. Upper panel represents images taken post-24 hours of normoxic treatment. Lower panel represents images taken post-24 hours of hyperoxic treatment. Scale bar represents 75 μm. Data are representative of three individual experiments. Figure S2 (supplementary figure 2): changes in specific migration-related proteins post treatments. Intensities of (a) Hmox1, (b) PAK1, (c) RAF1, and (d) Cdc42ep4 plotted from the mass spectrometry results. Graphs show the changes in protein intensities upon different treatment conditions. Data are representative of five independent experiments. Bars and error represent mean ± SEM of replicate measurements. ∗ represents statistically significant differences in comparison to normoxic control, # represents statistically significant differences in comparison to hyperoxic control, and § represents statistically significant differences between normoxic and hyperoxic treatments within the same group. Single signs represent a p value < 0.05, double signs represent p < 0.01, triple signs represent p < 0.001, and quadruple signs represent p < 0.0001(Student's t-test). Figure S3 (supplementary figure 3): complete heat map of canonical pathway analysis of significantly enriched proteins in the OLN93 cells post cotreatment of adiol+E2 in comparison to normoxic and hyperoxic controls using IPA. Negative z-score values are indicated in blue, and positive z-score values are indicated in red. Cutoff p value < 0.05 (Fisher's exact test). Table T1 (supplementary table 1): details of mass spectrometry procedure. (a) LC-MS/MS parameter (data independent mode; quantitative data). (b) Spectronaut parameters for peptide/protein identification and intensity extraction. Table T2 (supplementary table 2): functional categorization of proteins with [file 2606880.f1.zip › Figure S3.pdf]

Z-score value

-2 0 2

Canonical Pathways	N_AD+E2 vs N_Ctrl	H_Ctrl vs N_Ctrl	H_AD+E2 vs N_Ctrl
Signaling by Rho Family GTPases			
Autophagy			
cAMP-mediated signaling	N/A	N/A	
NER (Nucleotide Excision Repair, Enhanced Pathway)	N/A		
Cell Cycle Control of Chromosomal Replication	N/A		
Colorectal Cancer Metastasis Signaling	N/A		
Opioid Signaling Pathway			
IL-1 Signaling	N/A	N/A	
Role of PKR in Interferon Induction and Antiviral Response	N/A		
Xenobiotic Metabolism AHR Signaling Pathway	N/A		
Estrogen Receptor Signaling			
FLT3 Signaling in Hematopoietic Progenitor Cells	N/A		
Xenobiotic Metabolism PXR Signaling Pathway			
Reelin Signaling in Neurons	N/A	N/A	
RANK Signaling in Osteoclasts	N/A	N/A	
CD40 Signaling	N/A	N/A	
Netrin Signaling	N/A	N/A	
iNOS Signaling	N/A	N/A	
Superpathway of Cholesterol Biosynthesis	N/A	N/A	
Pyridoxal 5'-phosphate Salvage Pathway	N/A		
Salvage Pathways of Pyrimidine Ribonucleotides	N/A		
Pulmonary Fibrosis Idiopathic Signaling Pathway	N/A		
Regulation Of The Epithelial Mesenchymal Transition By Growth Factors Pathway	N/A	N/A	
BMP signaling pathway	N/A	N/A	
Natural Killer Cell Signaling	N/A		
Xenobiotic Metabolism CAR Signaling Pathway	N/A		
JAK/STAT Signaling	N/A		
Cardiac Hypertrophy Signaling (Enhanced)			
CLEAR Signaling Pathway			
Pulmonary Healing Signaling Pathway	N/A		
HGF Signaling	N/A		
IL-6 Signaling	N/A	N/A	
PAK Signaling	N/A	N/A	
Agrin Interactions at Neuromuscular Junction	N/A	N/A	
CNTF Signaling	N/A		
ILK Signaling	N/A		
Paxillin Signaling		N/A	
RAC Signaling			
IL-3 Signaling	N/A		
Renin-Angiotensin Signaling			
Necroptosis Signaling Pathway	N/A	N/A	
Dopamine-DARPP32 Feedback in cAMP Signaling			
p38 MAPK Signaling	N/A	N/A	
IGF-1 Signaling	N/A	N/A	
Melanocyte Development and Pigmentation Signaling	N/A	N/A	
Mouse Embryonic Stem Cell Pluripotency	N/A	N/A	
Corticotropin Releasing Hormone Signaling	N/A	N/A	
IL-7 Signaling Pathway	N/A	N/A	
Activation of IRF by Cytosolic Pattern Recognition Receptors	N/A		
GM-CSF Signaling	N/A		
Acute Myeloid Leukemia Signaling	N/A		
Growth Hormone Signaling	N/A		
Apoptosis Signaling	N/A		
Aryl Hydrocarbon Receptor Signaling	N/A		
Integrin Signaling	N/A		
EIF2 Signaling			
Thrombin Signaling			
Cardiac Hypertrophy Signaling			
Role of NFAT in Cardiac Hypertrophy			
Synaptogenesis Signaling Pathway			
ERK/MAPK Signaling			
Senescence Pathway			
Dilated Cardiomyopathy Signaling Pathway	N/A	N/A	
Acute Phase Response Signaling	N/A	N/A	
Angiopoietin Signaling	N/A		
Pancreatic Adenocarcinoma Signaling	N/A		
PDGF Signaling	N/A		
Calcium Signaling			
Adrenomedullin signaling pathway			
Oxytocin Signaling Pathway			
Insulin Secretion Signaling Pathway			
TGF-Î² Signaling	N/A	N/A	
PEDF Signaling	N/A	N/A	
Type II Diabetes Mellitus Signaling	N/A	N/A	
Melatonin Signaling	N/A	N/A	
Estrogen-mediated S-phase Entry	N/A	N/A	

Renal Cell Carcinoma Signaling	N/A	N/A	
GDNF Family Ligand-Receptor Interactions	N/A	N/A	
Inhibition of ARE-Mediated mRNA Degradation Pathway	N/A	N/A	
Oncostatin M Signaling	N/A		
Ephrin Receptor Signaling			
Ferroptosis Signaling Pathway			
G Beta Gamma Signaling			
GNRH Signaling			
Sirtuin Signaling Pathway	N/A		
NGF Signaling	N/A	N/A	
Androgen Signaling	N/A	N/A	
CCR3 Signaling in Eosinophils	N/A		
SNARE Signaling Pathway	N/A		
Nitric Oxide Signaling in the Cardiovascular System	N/A		
EGF Signaling	N/A		
FGF Signaling	N/A		
Production of Nitric Oxide and Reactive Oxygen Species in Macrophages	N/A		
BEX2 Signaling Pathway	N/A		
Thrombopoietin Signaling	N/A		
Prolactin Signaling	N/A		
P2Y Purigenic Receptor Signaling Pathway	N/A		
PI3K Signaling in B Lymphocytes			
Neuropathic Pain Signaling In Dorsal Horn Neurons			
ERBB Signaling	N/A	N/A	
HIF1Î± Signaling	N/A		
Synaptic Long Term Potentiation			
HER-2 Signaling in Breast Cancer	N/A		
Actin Cytoskeleton Signaling			
CDK5 Signaling	N/A	N/A	
Endocannabinoid Developing Neuron Pathway	N/A	N/A	
Macropinocytosis Signaling	N/A	N/A	
Glioma Signaling	N/A	N/A	
Pyroptosis Signaling Pathway	N/A	N/A	
ERB2-ERBB3 Signaling	N/A	N/A	
Apelin Adipocyte Signaling Pathway	N/A	N/A	
NF-ÎºB Activation by Viruses	N/A		
Apelin Endothelial Signaling Pathway	N/A		
Cyclins and Cell Cycle Regulation	N/A		
FcÎ³ Receptor-mediated Phagocytosis in Macrophages and Monocytes	N/A		
Sperm Motility			
Fc Epsilon RI Signaling	N/A	N/A	
PI3K/AKT Signaling	N/A		
Semaphorin Neuronal Repulsive Signaling Pathway	N/A		
CXCR4 Signaling	N/A		
Erythropoietin Signaling Pathway			
fMLP Signaling in Neutrophils			
GÎ±q Signaling			
Insulin Receptor Signaling			
Protein Kinase A Signaling			
Cardiac Î²-adrenergic Signaling	N/A	N/A	
IL-8 Signaling	N/A	N/A	
PXR/RXR Activation	N/A	N/A	
UVA-Induced MAPK Signaling	N/A	N/A	
UVC-Induced MAPK Signaling	N/A	N/A	
ERBB4 Signaling	N/A	N/A	
Cell Cycle: G1/S Checkpoint Regulation	N/A	N/A	
FcÎ³RIIB Signaling in B Lymphocytes	N/A	N/A	
VEGF Family Ligand-Receptor Interactions	N/A	N/A	
LPS-stimulated MAPK Signaling	N/A		
Cholecystokinin/Gastrin-mediated Signaling	N/A		
Aldosterone Signaling in Epithelial Cells	N/A		
Huntington's Disease Signaling	N/A		
Breast Cancer Regulation by Stathmin1	N/A		
ID1 Signaling Pathway	N/A		
Endocannabinoid Neuronal Synapse Pathway		N/A	
GPCR-Mediated Nutrient Sensing in Enteroendocrine Cells			
D-myo-inositol (3,4,5,6)-tetrakisphosphate Biosynthesis		N/A	
D-myo-inositol (1,4,5,6)-Tetrakisphosphate Biosynthesis		N/A	
3-phosphoinositide Degradation			
3-phosphoinositide Biosynthesis			
AMPK Signaling			
Superpathway of Inositol Phosphate Compounds			
Hepatic Fibrosis Signaling Pathway			
PTEN Signaling	N/A	N/A	
Endothelin-1 Signaling			
PPARÎ±/RXRÎ± Activation			
GÎ±i Signaling	N/A	N/A	
14-3-3-mediated Signaling	N/A	N/A	
Chemokine Signaling	N/A	N/A	
Neuregulin Signaling	N/A	N/A	
Apelin Cardiomyocyte Signaling Pathway	N/A		

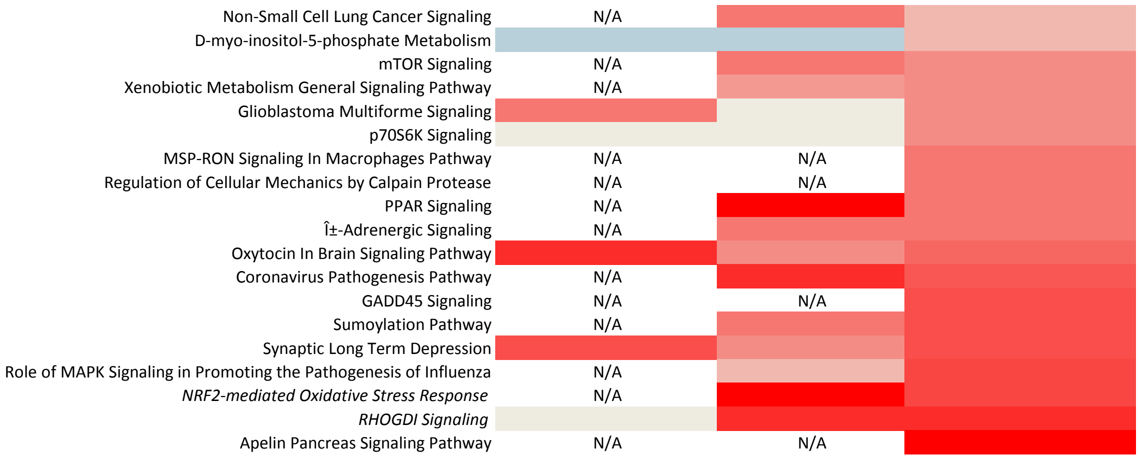

Figure S3: Cannonical pathway analysis of significantly enriched proteins in the OLN93 cells post co-treatment of adiol+E2 in comparison to normoxia and hyperoxia controls using IPA. Heat-map representation of enriched pathways sorted by z-score. Higher negative z-score is indicated in blue and higher positive z-score values are indicated in red. Cut off p value < 0.05 (Fisher's exact test).
