# Supplementary material for: Fetal Zone Steroids Show Discrete Effects on Hyperoxia-Induced Attenuation of Migration in Cultured Oligodendrocyte Progenitor Cells
Source: Oxid Med Cell Longev. 2022 May 9;2022:2606880. doi: 10.1155/2022/2606880 (PMC9110221; doi:10.1155/2022/2606880)
Supplement: Supplementary Materials — Figure S1 (supplementary figure 1): Ki67 staining of OLN93 cells post-24 hours of normoxic and hyperoxic (80% O2) treatments. Representative immunofluorescence images of OLN93 cells stained for Ki67 proliferation marker. Upper panel represents images taken post-24 hours of normoxic treatment. Lower panel represents images taken post-24 hours of hyperoxic treatment. Scale bar represents 75 μm. Data are representative of three individual experiments. Figure S2 (supplementary figure 2): changes in specific migration-related proteins post treatments. Intensities of (a) Hmox1, (b) PAK1, (c) RAF1, and (d) Cdc42ep4 plotted from the mass spectrometry results. Graphs show the changes in protein intensities upon different treatment conditions. Data are representative of five independent experiments. Bars and error represent mean ± SEM of replicate measurements. ∗ represents statistically significant differences in comparison to normoxic control, # represents statistically significant differences in comparison to hyperoxic control, and § represents statistically significant differences between normoxic and hyperoxic treatments within the same group. Single signs represent a p value < 0.05, double signs represent p < 0.01, triple signs represent p < 0.001, and quadruple signs represent p < 0.0001(Student's t-test). Figure S3 (supplementary figure 3): complete heat map of canonical pathway analysis of significantly enriched proteins in the OLN93 cells post cotreatment of adiol+E2 in comparison to normoxic and hyperoxic controls using IPA. Negative z-score values are indicated in blue, and positive z-score values are indicated in red. Cutoff p value < 0.05 (Fisher's exact test). Table T1 (supplementary table 1): details of mass spectrometry procedure. (a) LC-MS/MS parameter (data independent mode; quantitative data). (b) Spectronaut parameters for peptide/protein identification and intensity extraction. Table T2 (supplementary table 2): functional categorization of proteins with [file 2606880.f1.zip › Supplemental Table T1 (1).pdf]

Supplemental Table S1_(a)

LC-MS/MS parameter (data independent mode; quantitative data)

Data independent analyses (DIA)

Reversed phase liquid chromatography	**Ultimate 3000 RSLC (Thermo Scientific)**
Trap column	75 µm inner diameter, packed with 3 µm C18 particles (Acclaim PepMap100, Thermo Scientific)
Analytical column	75 µm inner diameter, packed with 2.6 µm C18 particles (Accucore, 25 cm, Thermo Scientific)
Flow rate	300 nl/min
column oven temperature	40°C
buffer system	binary buffer system consisting of 0.1% acetic acid in HPLC-grade water (buffer A) and 100% ACN in 0.1% acetic acid (buffer B)
gradient	gradient of buffer B: 2min 2% to 5 %, 8min 5%, 120min 5% to 25%, 5min 25 to 40%, 2 min 40% to 90%, 5 min 90%, 3 min 90% to 2%, 5 min 2%
Mass spectrometer	**Q Exactive HF**
operation mode	data-independent
electrospray	Nanospray Flex Ion Source
Full MS	
MS scan resolution	60,000
AGC target	5e6
maximum ion injection time for the MS scan	200 ms
Scan range	333 to 1650 *m/z*
Spectra data type	profile
dd-MS2	
Resolution	30,000
MS/MS AGC target	3e6
maximum ion injection time for the MS/MS scans	auto
Spectra data type	profile
selection for MS/MS	1
isolation window	56 windows *m/z* 13
Fixed first mass	200
dissociation mode	higher energy collisional dissociation (HCD)
normalized collision energy	stepped, 27.5
dissociation mode	HCD

Supplemental Table S1_(b)

Spectronaut parameters for peptide/Protein identification and intensity extraction

Spectronaut 15.6.211220.50606	
Computer Name: AGVOE-SPECTRONA	
User Domain Name: AGVOE-SPECTRONA	
User Name: spectronaut	
Analysis Mode: UI	
Analysis Type: directDIA	
Analysis Date: 28-January-2022 23:52:08 UTC+1	
Settings Used:	
Pulsar Search\Peptides	
Toggle N-terminal M:	True
Min Peptide Length:	7
Max Peptide Length:	52
Missed Cleavages:	2
Digest Type:	Specific
Enzymes / Cleavage Rules:	Trypsin/P
Pulsar Search\Labeling	
Channels:	
Channel 1:	False
Channel 2:	False
Channel 3:	False
DIA Analysis\Data Extraction	
MS1 Mass Tolerance Strategy:	Dynamic
Correction Factor:	1
MS2 Mass Tolerance Strategy:	Dynamic
Correction Factor:	1
Intensity Extraction MS1:	Maximum Intensity
Intensity Extraction MS2:	Maximum Intensity
DIA Analysis\XIC Extraction	
XIC IM Extraction Window:	Dynamic
Correction Factor:	1
XIC RT Extraction Window:	Dynamic
Correction Factor:	1
Pulsar Search\Modifications	
Max Variable Modifications:	5
Database	
Original File:	Rattus_RefSeq_190326.fasta
Select Modifications:	
Fixed Modifications::	Carbamidomethyl (C)
Variable Modifications: :	Acetyl (Protein N-term), Oxidation (M)
DIA Analysis\Calibration	

MS1 Mass Tolerance Strategy:	System Default
MS2 Mass Tolerance Strategy:	System Default
Precision iRT:	True
iRT <-> RT Regression Type:	Local (Non-Linear) Regression
Exclude Deamidated Peptides:	True
MZ Extraction Strategy:	Maximum Intensity
Allow source specific iRT Calibration:	True
DIA Analysis\Identification	
Generate Decoys:	True
Decoy Limit Strategy:	Dynamic
Library Size Fraction:	0.1
Decoy Method:	Mutated
Preferred Fragment Source:	NN Predicted Fragments
Machine Learning:	Per Run
Exclude Duplicate Assays:	True
Precursor PEP Cutoff:	1
Protein Qvalue Cutoff (Experiment):	0.01
Protein Qvalue Cutoff (Run):	0.05
Exclude Single Hit Proteins:	False
Pvalue Estimator:	Kernel Density Estimator
Precursor Qvalue Cutoff:	0.001
Single Hit Definition:	By Stripped Sequence
DIA Analysis\Quantification	
Interference Correction:	True
MS1 Min:	2
MS2 Min:	3
Exclude All Multi-Channel Interferences:	True
Only Identified Peptides:	True
Protein LFQ Method:	Automatic
Major (Protein) Grouping:	by Protein Group Id
Minor (Peptide) Grouping:	by Stripped Sequence
Minor Group Top N:	False
Minor Group Quantity:	Sum precursor quantity
Major Group Top N:	True
Min:	2
Max:	3
Major Group Quantity:	Mean peptide quantity
Quantity MS-Level:	MS2
Quantity Type:	Area
Proteotypicity Filter:	None
Data Filtering:	Qvalue percentile
Fraction:	0.2
Imputing Strategy:	No Imputing
Cross Run Normalization:	False

DIA Analysis\PTM Workflow	
PTM Localization:	True
Probability Cutoff:	0.75
PTM Analysis:	True
Multiplicity:	True
Run Clustering:	False
PTM Consolidation:	Sum
Flanking Region:	7
DIA Analysis\Workflow	
MS2 DeMultiplexing:	Automatic
Run Limit for directDIA Library:	-1
Method Evaluation:	False
Profiling Strategy:	iRT Profiling
Profiling Row Selection:	Minimum Qvalue Row Selection
Qvalue Threshold:	0.001
Profiling Target Selection:	Profile only non-identfied Precursor
Identification Criterion:	Qvalue
Threshold:	0.001
Carry-over exact Peak Boundaries:	False
Unify Peptide Peaks Strategy:	Select corresponding Peak
DIA Analysis\Protein Inference	
Protein Inference Workflow:	Automatic
Inference Algorithm:	IDPicker
DIA Analysis\Post Analysis	
Calculate Sample Correlation Matrix:	True
Calculate Explained TIC:	Quick
Differential Abundance Grouping:	Major Group (Quantification Settings)
Smallest Quantitative Unit:	Precursor Ion (Quantification Settings)
Use All MS-Level Quantities:	False
Differential Abundance Testing:	Paired t-test
Group-Wise Testing Correction:	False
Run Clustering:	True
Distance Metric:	Manhattan Distance
Linkage Strategy:	Ward's Method
Z-score transformation:	False
Order Runs by Clustering:	True
DIA Analysis\Pipeline Mode	
Post Analysis Reports:	
Scoring Histograms:	True
Data Completeness Bar Chart:	True
Run Identifications Bar Chart:	True
CV Density Line Chart:	True
CVs Below X Bar Chart:	True
Generate SNE File:	True

Store Iontraces in SNE:	False
Report Schema:	C_FunGene_complex (Normal)
Reporting Unit:	Across Experiment
Pulsar Search\Identification	
Peptide FDR:	0.01
Protein Group FDR:	0.01
PSM FDR:	0.01
Pulsar Search\Tolerances	
Tolerance Parameters:	
Thermo Orbitrap:	
Calibration Search:	Dynamic
MS1 Correction Factor:	1
MS2 Correction Factor:	1
Main Search:	Dynamic
MS1 Correction Factor:	1
MS2 Correction Factor:	1
TOF:	
Calibration Search:	Dynamic
MS1 Correction Factor:	1
MS2 Correction Factor:	1
Main Search:	Dynamic
MS1 Correction Factor:	1
MS2 Correction Factor:	1
Thermo IonTrap:	
Calibration Search:	Dynamic
MS1 Correction Factor:	1
MS2 Correction Factor:	1
Main Search:	Dynamic
MS1 Correction Factor:	1
MS2 Correction Factor:	1
Pulsar Search\Workflow	
Use DNN Predicted Ion Mobility:	Auto
Fragment Ion Selection Strategy:	Intensity Based
In-Silico Generate Missing Channels:	False
Pulsar Search\Result Filters	
Precursors:	
Best N Fragments per Peptide:	True
Min:	6
Max:	10
Channel Count:	False
Modifications:	None
Amino Acids:	False
Best N Peptides per Protein Group:	False
FASTA Matched:	False

Missed Cleavage:	False
Peptide Charge:	False
Proteotypicity:	False
__Fragment Ions:__	
m/z :	True
Min:	300
Max:	1800
Ion Charge:	False
Ion Loss Type:	False
Ion Type:	False
Ion AA Length:	True
N:	3
Relative Intensity:	True
Min:	5
