# Supplementary material for: Fetal Zone Steroids Show Discrete Effects on Hyperoxia-Induced Attenuation of Migration in Cultured Oligodendrocyte Progenitor Cells
Source: Oxid Med Cell Longev. 2022 May 9;2022:2606880. doi: 10.1155/2022/2606880 (PMC9110221; doi:10.1155/2022/2606880)
Supplement: Supplementary Materials — Figure S1 (supplementary figure 1): Ki67 staining of OLN93 cells post-24 hours of normoxic and hyperoxic (80% O2) treatments. Representative immunofluorescence images of OLN93 cells stained for Ki67 proliferation marker. Upper panel represents images taken post-24 hours of normoxic treatment. Lower panel represents images taken post-24 hours of hyperoxic treatment. Scale bar represents 75 μm. Data are representative of three individual experiments. Figure S2 (supplementary figure 2): changes in specific migration-related proteins post treatments. Intensities of (a) Hmox1, (b) PAK1, (c) RAF1, and (d) Cdc42ep4 plotted from the mass spectrometry results. Graphs show the changes in protein intensities upon different treatment conditions. Data are representative of five independent experiments. Bars and error represent mean ± SEM of replicate measurements. ∗ represents statistically significant differences in comparison to normoxic control, # represents statistically significant differences in comparison to hyperoxic control, and § represents statistically significant differences between normoxic and hyperoxic treatments within the same group. Single signs represent a p value < 0.05, double signs represent p < 0.01, triple signs represent p < 0.001, and quadruple signs represent p < 0.0001(Student's t-test). Figure S3 (supplementary figure 3): complete heat map of canonical pathway analysis of significantly enriched proteins in the OLN93 cells post cotreatment of adiol+E2 in comparison to normoxic and hyperoxic controls using IPA. Negative z-score values are indicated in blue, and positive z-score values are indicated in red. Cutoff p value < 0.05 (Fisher's exact test). Table T1 (supplementary table 1): details of mass spectrometry procedure. (a) LC-MS/MS parameter (data independent mode; quantitative data). (b) Spectronaut parameters for peptide/protein identification and intensity extraction. Table T2 (supplementary table 2): functional categorization of proteins with [file 2606880.f1.zip › Supplemental Table T2.pdf]

Supplemental Table S2. Functional categorization of proteins with differential abundance under different treatment conditions with

adiol (AD) and AD+estradiol (E2) in comparison to normoxic (N) and hyperoxic (H) control and prediction of activity of "Cannonical pathways ".

Proteins with $p < 0.05$ were considered. Categories are sorted by ascending activation score Z.

© 2000-2022 QIAGEN. All rights reserved.

Canonical Pathways	N_AD vs N_Ctrl	N_AD_E2 vs N_Ctrl	H_Ctrl vs N_Ctrl	H_AD vs N_Ctrl	H_AD_E2 vs N_Ctrl	H_AD vs H_Ctrl	H_AD_E2 vs H_Ctrl
Cell Cycle Control of Chromosomal Replication	N/A	N/A	-2.646	N/A	-2.449	N/A	N/A
Actin Cytoskeleton Signaling	N/A	0.0	-2.449	-0.816	-0.577	1.342	N/A
NER (Nucleotide Excision Repair, Enhanced Pathway)	N/A	N/A	-2.449	-2.449	-2.449	N/A	N/A
Salvage Pathways of Pyrimidine Ribonucleotides	N/A	N/A	-2.449	-1.342	-2.0	N/A	N/A
JAK/STAT Signaling	N/A	N/A	-2.449	-1.342	-1.89	N/A	N/A
Signaling by Rho Family GTPases	N/A	-1.0	-2.333	-0.816	-3.0	N/A	N/A
FLT3 Signaling in Hematopoietic Progenitor Cells	N/A	N/A	-2.236	-1.633	-2.121	N/A	N/A
Pulmonary Fibrosis Idiopathic Signaling Pathway	N/A	N/A	-2.236	-1.633	-1.897	N/A	N/A
Integrin Signaling	N/A	N/A	-2.236	-1.134	-1.342	N/A	N/A
Pyridoxal 5'-phosphate Salvage Pathway	N/A	N/A	-2.236	N/A	-2.0	N/A	N/A
ID1 Signaling Pathway	N/A	N/A	-2.236	0.0	0.0	N/A	N/A
Xenobiotic Metabolism PXR Signaling Pathway	N/A	-1.0	-2.121	-0.447	-2.121	-2.236	N/A
Xenobiotic Metabolism CAR Signaling Pathway	0.0	N/A	-2.121	N/A	-1.89	-2.0	N/A
Ferroptosis Signaling Pathway	N/A	1.0	-1.897	-0.816	-1.0	1.0	N/A
ERK/MAPK Signaling	N/A	0.0	-1.667	-1.897	-1.155	0.0	N/A
IL-3 Signaling	N/A	N/A	-1.633	-1.89	-1.508	0.0	N/A
HGF Signaling	N/A	N/A	-1.342	-1.414	-1.667	N/A	N/A
PDGF Signaling	N/A	N/A	-1.342	-1.342	-1.134	N/A	N/A
HER-2 Signaling in Breast Cancer	N/A	N/A	-1.342	-1.342	-0.632	N/A	N/A
Thrombopoietin Signaling	N/A	N/A	-1.342	-1.0	-0.816	N/A	N/A
Prolactin Signaling	N/A	N/A	-1.342	-1.0	-0.816	N/A	N/A
Aryl Hydrocarbon Receptor Signaling	N/A	N/A	-1.342	N/A	-1.342	N/A	N/A
P2Y Purigenic Receptor Signaling Pathway	N/A	N/A	-1.342	N/A	-0.816	N/A	N/A
Autophagy	N/A	-1.0	-1.134	-1.897	-2.887	0.378	N/A
Synaptogenesis Signaling Pathway	N/A	0.0	-1.134	-0.632	-1.265	N/A	N/A
Breast Cancer Regulation by Stathmin1	N/A	N/A	-1.069	1.342	0.0	0.816	N/A
AMPK Signaling	N/A	0.0	1.265	-1.134	0.0	1.0	N/A
Calcium Signaling	N/A	0.447	1.342	0.816	-1.134	-2.0	N/A
Coronavirus Pathogenesis Pathway	N/A	N/A	1.633	0.447	1.265	N/A	N/A
RHOGDI Signaling	N/A	0.0	1.633	N/A	1.633	N/A	N/A
NRF2-mediated Oxidative Stress Response	N/A	N/A	2.121	N/A	1.414	-1.0	N/A
Role of NFAT in Cardiac Hypertrophy	N/A	0.0	0.0	-0.378	-1.265	-1.342	N/A
Erythropoietin Signaling Pathway	N/A	1.0	0.0	N/A	-0.378	0.0	N/A
Synaptic Long Term Potentiation	N/A	0.0	0.0	-0.447	-0.707	-0.816	N/A
Senescence Pathway	N/A	-0.447	0.0	-2.121	-1.155	-1.0	N/A

Glioblastoma Multiforme Signaling	N/A	1.0	0.0	1.0	0.816	-1.0	N/A
GNRH Signaling	N/A	1.0	0.0	-0.816	-0.905	-1.0	N/A
fMLP Signaling in Neutrophils	N/A	1.0	0.0	0.0	-0.378	-1.0	N/A
Hepatic Fibrosis Signaling Pathway	N/A	1.0	0.0	-1.0	0.277	N/A	N/A
EGF Signaling	N/A	N/A	0.0	-1.342	-0.816	N/A	N/A
FGF Signaling	N/A	N/A	0.0	-1.342	-0.816	N/A	N/A
HIF1Î± Signaling	N/A	N/A	0.0	-1.342	-0.707	N/A	N/A
3-phosphoinositide Biosynthesis	N/A	0.0	0.0	1.0	0.0	N/A	N/A
p70S6K Signaling	N/A	0.0	0.0	0.0	0.816	N/A	N/A
Production of Nitric Oxide and Reactive Oxygen Species in Macrophage	N/A	N/A	0.0	N/A	-0.816	N/A	N/A
BEX2 Signaling Pathway	N/A	N/A	0.0	N/A	-0.816	N/A	N/A
Huntington's Disease Signaling	N/A	N/A	0.0	N/A	0.0	N/A	N/A
Insulin Secretion Signaling Pathway	N/A	-0.707	-0.277	0.632	-1.069	-0.816	N/A
Protein Kinase A Signaling	N/A	-1.0	0.333	0.0	-0.302	-0.707	N/A
GÎ±q Signaling	N/A	0.0	0.378	N/A	-0.378	N/A	N/A
Dopamine-DARPP32 Feedback in cAMP Signaling	N/A	-0.447	-0.378	-1.0	-1.414	-1.342	N/A
Renin-Angiotensin Signaling	N/A	1.0	-0.378	-0.816	-1.508	-1.0	N/A
Superpathway of Inositol Phosphate Compounds	N/A	-0.447	-0.378	1.0	0.0	N/A	N/A
CXCR4 Signaling	N/A	N/A	-0.378	-0.816	-0.378	N/A	N/A
Role of MAPK Signaling in Promoting the Pathogenesis of Influenza	N/A	N/A	0.447	N/A	1.414	0.0	N/A
SNARE Signaling Pathway	N/A	N/A	0.447	N/A	-0.816	1.0	N/A
ILK Signaling	N/A	N/A	0.447	-1.0	-1.633	N/A	N/A
Apoptosis Signaling	N/A	N/A	0.447	N/A	-1.342	N/A	N/A
Nitric Oxide Signaling in the Cardiovascular System	N/A	N/A	0.447	N/A	-0.816	N/A	N/A
Neuropathic Pain Signaling In Dorsal Horn Neurons	N/A	0.0	0.447	N/A	-0.816	N/A	N/A
Sperm Motility	N/A	0.0	0.447	N/A	-0.447	N/A	N/A
GPCR-Mediated Nutrient Sensing in Enteroendocrine Cells	N/A	0.447	0.447	N/A	0.0	N/A	N/A
Semaphorin Neuronal Repulsive Signaling Pathway	N/A	N/A	0.447	N/A	-0.378	N/A	N/A
Aldosterone Signaling in Epithelial Cells	N/A	N/A	0.447	N/A	0.0	N/A	N/A
Cholecystokinin/Gastrin-mediated Signaling	N/A	N/A	0.447	0.0	0.0	N/A	N/A
Cardiac Hypertrophy Signaling	N/A	0.0	-0.447	-0.816	-1.265	-0.816	N/A
PI3K Signaling in B Lymphocytes	N/A	0.447	-0.447	0.0	-0.816	-1.0	N/A
D-myo-inositol-5-phosphate Metabolism	N/A	-0.447	-0.447	N/A	0.447	N/A	N/A
FcÎ³ Receptor-mediated Phagocytosis in Macrophages and Monocytes	N/A	N/A	-0.447	N/A	-0.447	N/A	N/A
3-phosphoinositide Degradation	N/A	0.0	-0.447	N/A	0.0	N/A	N/A
PPARÎ±/RXRÎ± Activation	N/A	0.0	0.707	0.447	0.378	-0.447	N/A
Xenobiotic Metabolism General Signaling Pathway	N/A	N/A	0.707	0.0	0.816	-1.0	N/A
Thrombin Signaling	N/A	0.447	-0.707	0.0	-1.265	0.447	N/A
Opioid Signaling Pathway	N/A	0.0	-0.707	-1.134	-2.324	-0.816	N/A
Synaptic Long Term Depression	N/A	1.342	0.816	1.0	1.342	0.0	N/A
Oxytocin In Brain Signaling Pathway	N/A	1.633	0.816	0.447	1.134	N/A	N/A
Endothelin-1 Signaling	N/A	0.816	0.816	0.0	0.378	N/A	N/A
Insulin Receptor Signaling	N/A	-0.447	-0.816	-1.134	-0.302	0.0	N/A
Adrenomedullin signaling pathway	N/A	0.0	-0.816	-0.447	-1.134	-1.0	N/A
Pancreatic Adenocarcinoma Signaling	N/A	N/A	-0.816	-2.0	-1.134	N/A	N/A

Cardiac Hypertrophy Signaling (Enhanced)	N/A	-0.632	-0.905	0.0	-1.807	-0.707	N/A
PI3K/AKT Signaling	N/A	N/A	1.0	N/A	-0.378	0.0	N/A
Sirtuin Signaling Pathway	N/A	N/A	1.0	0.447	-0.832	0.447	N/A
mTOR Signaling	N/A	N/A	1.0	-1.0	0.816	N/A	N/A
Sumoylation Pathway	N/A	N/A	1.0	N/A	1.342	N/A	N/A
GPCR-Mediated Integration of Enteroendocrine Signaling Exemplified I	N/A	1.0	1.0	N/A	N/A	N/A	N/A
Î±-Adrenergic Signaling	N/A	N/A	1.0	N/A	1.0	N/A	N/A
Apelin Cardiomyocyte Signaling Pathway	N/A	N/A	1.0	N/A	0.447	N/A	N/A
Non-Small Cell Lung Cancer Signaling	N/A	N/A	1.0	N/A	0.447	N/A	N/A
Antioxidant Action of Vitamin C	N/A	N/A	1.0	N/A	N/A	N/A	N/A
ATM Signaling	N/A	N/A	1.0	N/A	N/A	N/A	N/A
CLEAR Signaling Pathway	-1.0	-0.816	-1.0	-0.333	-1.698	0.333	N/A
Oxytocin Signaling Pathway	N/A	0.632	-1.0	-1.414	-1.069	-0.378	N/A
Xenobiotic Metabolism AHR Signaling Pathway	-1.0	N/A	-1.0	N/A	-2.236	N/A	N/A
G Beta Gamma Signaling	N/A	0.447	-1.0	-1.0	-1.0	N/A	N/A
CCR3 Signaling in Eosinophils	N/A	N/A	-1.0	-1.0	-0.816	N/A	N/A
NF-ÎºB Activation by Viruses	N/A	N/A	-1.0	-1.0	-0.447	N/A	N/A
Growth Hormone Signaling	N/A	N/A	-1.0	N/A	-1.342	N/A	N/A
Angiopoietin Signaling	N/A	N/A	-1.0	N/A	-1.134	N/A	N/A
LPS-stimulated MAPK Signaling	N/A	N/A	-1.0	-1.0	0.0	N/A	N/A
Apelin Endothelial Signaling Pathway	N/A	N/A	-1.0	N/A	-0.447	N/A	N/A
Cyclins and Cell Cycle Regulation	N/A	N/A	-1.0	N/A	-0.447	N/A	N/A
Pulmonary Healing Signaling Pathway	N/A	N/A	-1.89	-1.0	-1.667	N/A	N/A
PPAR Signaling	N/A	N/A	2.0	N/A	1.0	-1.0	N/A
Role of PKR in Interferon Induction and Antiviral Response	N/A	N/A	-2.0	-2.0	-2.236	N/A	N/A
Ephrin Receptor Signaling	N/A	2.0	-2.0	-1.134	-1.0	N/A	N/A
Colorectal Cancer Metastasis Signaling	N/A	N/A	-2.0	-1.342	-2.333	N/A	N/A
Activation of IRF by Cytosolic Pattern Recognition Receptors	N/A	N/A	-2.0	-2.236	-1.342	N/A	N/A
Natural Killer Cell Signaling	N/A	N/A	-2.0	-1.633	-1.89	N/A	N/A
RAC Signaling	N/A	1.0	-2.0	-0.447	-1.633	N/A	N/A
CNTF Signaling	N/A	N/A	-2.0	-1.342	-1.633	N/A	N/A
GM-CSF Signaling	N/A	N/A	-2.0	-1.0	-1.342	N/A	N/A
Oncostatin M Signaling	N/A	N/A	-2.0	-1.0	-1.0	N/A	N/A
Acute Myeloid Leukemia Signaling	N/A	N/A	-2.0	N/A	-1.342	N/A	N/A
Fatty Acid Î²-oxidation I	N/A	N/A	-2.0	N/A	N/A	N/A	N/A
IL-9 Signaling	N/A	N/A	-2.0	N/A	N/A	N/A	N/A
Estrogen Receptor Signaling	N/A	-0.816	-2.53	-0.816	-2.138	-0.447	N/A
EIF2 Signaling	N/A	-1.0	-3.0	-1.265	-1.342	-0.447	N/A
cAMP-mediated signaling	N/A	N/A	N/A	-2.0	-2.449	0.447	N/A
CDK5 Signaling	N/A	N/A	N/A	0.0	-0.447	-0.447	N/A
Reelin Signaling in Neurons	N/A	N/A	N/A	-0.447	-2.0	1.0	N/A
Cardiac Î²-adrenergic Signaling	N/A	N/A	N/A	N/A	0.0	1.0	N/A
Dilated Cardiomyopathy Signaling Pathway	N/A	N/A	N/A	N/A	-1.134	-2.0	N/A
RANK Signaling in Osteoclasts	N/A	N/A	N/A	-2.0	-2.0	N/A	N/A
CD40 Signaling	N/A	N/A	N/A	-2.0	-2.0	N/A	N/A

Regulation Of The Epithelial Mesenchymal Transition By Growth Factoı	N/A	N/A	N/A	-1.342	-1.89	N/A	N/A
Paxillin Signaling	N/A	1.0	N/A	-0.447	-1.633	N/A	N/A
IL-6 Signaling	N/A	N/A	N/A	-1.342	-1.633	N/A	N/A
BMP signaling pathway	N/A	N/A	N/A	-1.0	-1.89	N/A	N/A
Acute Phase Response Signaling	N/A	N/A	N/A	-1.633	-1.134	N/A	N/A
p38 MAPK Signaling	N/A	N/A	N/A	-1.342	-1.342	N/A	N/A
Agrin Interactions at Neuromuscular Junction	N/A	N/A	N/A	-1.0	-1.633	N/A	N/A
TGF-Î² Signaling	N/A	N/A	N/A	-1.342	-1.0	N/A	N/A
Melanocyte Development and Pigmentation Signaling	N/A	N/A	N/A	-1.0	-1.342	N/A	N/A
Mouse Embryonic Stem Cell Pluripotency	N/A	N/A	N/A	-1.0	-1.342	N/A	N/A
Corticotropin Releasing Hormone Signaling	N/A	N/A	N/A	-1.0	-1.342	N/A	N/A
IL-1 Signaling	N/A	N/A	N/A	N/A	-2.236	N/A	N/A
Necroptosis Signaling Pathway	N/A	N/A	N/A	-0.816	-1.414	N/A	N/A
NGF Signaling	N/A	N/A	N/A	-1.342	-0.816	N/A	N/A
PAK Signaling	N/A	N/A	N/A	-0.447	-1.633	N/A	N/A
Netrin Signaling	N/A	N/A	N/A	N/A	-2.0	N/A	N/A
iNOS Signaling	N/A	N/A	N/A	N/A	-2.0	N/A	N/A
Apelin Pancreas Signaling Pathway	N/A	N/A	N/A	N/A	2.0	N/A	N/A
Superpathway of Cholesterol Biosynthesis	N/A	N/A	N/A	N/A	-2.0	N/A	N/A
PEDF Signaling	N/A	N/A	N/A	-1.0	-1.0	N/A	N/A
IL-13 Signaling Pathway	N/A	N/A	N/A	-2.0	N/A	N/A	N/A
Endocannabinoid Developing Neuron Pathway	N/A	N/A	N/A	-1.342	-0.447	N/A	N/A
IGF-1 Signaling	N/A	N/A	N/A	-0.447	-1.342	N/A	N/A
ERBB Signaling	N/A	N/A	N/A	-1.0	-0.707	N/A	N/A
PTEN Signaling	N/A	N/A	N/A	-1.0	0.378	N/A	N/A
IL-7 Signaling Pathway	N/A	N/A	N/A	N/A	-1.342	N/A	N/A
GADD45 Signaling	N/A	N/A	N/A	N/A	1.342	N/A	N/A
Type II Diabetes Mellitus Signaling	N/A	N/A	N/A	N/A	-1.0	N/A	N/A
Melatonin Signaling	N/A	N/A	N/A	N/A	-1.0	N/A	N/A
IL-8 Signaling	N/A	N/A	N/A	-1.0	0.0	N/A	N/A
MSP-RON Signaling In Macrophages Pathway	N/A	N/A	N/A	N/A	1.0	N/A	N/A
Estrogen-mediated S-phase Entry	N/A	N/A	N/A	N/A	-1.0	N/A	N/A
Renal Cell Carcinoma Signaling	N/A	N/A	N/A	N/A	-1.0	N/A	N/A
GDNF Family Ligand-Receptor Interactions	N/A	N/A	N/A	N/A	-1.0	N/A	N/A
Inhibition of ARE-Mediated mRNA Degradation Pathway	N/A	N/A	N/A	N/A	-1.0	N/A	N/A
Regulation of Cellular Mechanics by Calpain Protease	N/A	N/A	N/A	N/A	1.0	N/A	N/A
Androgen Signaling	N/A	N/A	N/A	N/A	-0.816	N/A	N/A
14-3-3-mediated Signaling	N/A	N/A	N/A	N/A	0.447	N/A	N/A
Chemokine Signaling	N/A	N/A	N/A	N/A	0.447	N/A	N/A
Glioma Signaling	N/A	N/A	N/A	N/A	-0.447	N/A	N/A
Macropinocytosis Signaling	N/A	N/A	N/A	0.0	-0.447	N/A	N/A
STAT3 Pathway	N/A	N/A	N/A	-0.447	N/A	N/A	N/A
Pyroptosis Signaling Pathway	N/A	N/A	N/A	N/A	-0.447	N/A	N/A
ERB2-ERBB3 Signaling	N/A	N/A	N/A	N/A	-0.447	N/A	N/A
Endocannabinoid Neuronal Synapse Pathway	N/A	0.447	N/A	0.0	0.0	N/A	N/A

Apelin Adipocyte Signaling Pathway	N/A	N/A	N/A	N/A	-0.447	N/A	N/A
GÎ±i Signaling	N/A	N/A	N/A	0.0	0.447	N/A	N/A
Neuregulin Signaling	N/A	N/A	N/A	N/A	0.447	N/A	N/A
Fc Epsilon RI Signaling	N/A	N/A	N/A	N/A	-0.378	N/A	N/A
BAG2 Signaling Pathway	N/A	N/A	N/A	N/A	N/A	N/A	N/A
UVB-Induced MAPK Signaling	N/A	N/A	N/A	N/A	N/A	N/A	N/A
PFKFB4 Signaling Pathway	N/A	N/A	N/A	N/A	N/A	N/A	N/A
Amyotrophic Lateral Sclerosis Signaling	N/A	N/A	N/A	N/A	N/A	N/A	N/A
Mevalonate Pathway I	N/A	N/A	N/A	N/A	N/A	N/A	N/A
nNOS Signaling in Neurons	N/A	N/A	N/A	N/A	N/A	N/A	N/A
GÎ±s Signaling	N/A	N/A	N/A	N/A	N/A	N/A	N/A
NADH Repair	N/A	N/A	N/A	N/A	N/A	N/A	N/A
Oleate Biosynthesis II (Animals)	N/A	N/A	N/A	N/A	N/A	N/A	N/A
Guanosine Nucleotides Degradation III	N/A	N/A	N/A	N/A	N/A	N/A	N/A
Superpathway of Methionine Degradation	N/A	N/A	N/A	N/A	N/A	N/A	N/A
PRPP Biosynthesis I	N/A	N/A	N/A	N/A	N/A	N/A	N/A
Caveolar-mediated Endocytosis Signaling	N/A	N/A	N/A	N/A	N/A	N/A	N/A
Fatty Acid Î±-oxidation	N/A	N/A	N/A	N/A	N/A	N/A	N/A
Telomere Extension by Telomerase	N/A	N/A	N/A	N/A	N/A	N/A	N/A
FXR/RXR Activation	N/A	N/A	N/A	N/A	N/A	N/A	N/A
Hereditary Breast Cancer Signaling	N/A	N/A	N/A	N/A	N/A	N/A	N/A
DNA Double-Strand Break Repair by Non-Homologous End Joining	N/A	N/A	N/A	N/A	N/A	N/A	N/A
Glutamate Degradation X	N/A	N/A	N/A	N/A	N/A	N/A	N/A
Glycolysis I	N/A	N/A	N/A	N/A	N/A	N/A	N/A
Proline Biosynthesis I	N/A	N/A	N/A	N/A	N/A	N/A	N/A
Histamine Degradation	N/A	N/A	N/A	N/A	N/A	N/A	N/A
Glutamate Degradation II	N/A	N/A	N/A	N/A	N/A	N/A	N/A
TCA Cycle II (Eukaryotic)	N/A	N/A	N/A	N/A	N/A	N/A	N/A
B Cell Activating Factor Signaling	N/A	N/A	N/A	N/A	N/A	N/A	N/A
S-methyl-5-thio-Î±-D-ribose 1-phosphate Degradation	N/A	N/A	N/A	N/A	N/A	N/A	N/A
IL-12 Signaling and Production in Macrophages	N/A	N/A	N/A	N/A	N/A	N/A	N/A
Citrulline-Nitric Oxide Cycle	N/A	N/A	N/A	N/A	N/A	N/A	N/A
Tryptophan Degradation X (Mammalian, via Tryptamine)	N/A	N/A	N/A	N/A	N/A	N/A	N/A
Pyrimidine Ribonucleotides Interconversion	N/A	N/A	N/A	N/A	N/A	N/A	N/A
Diphthamide Biosynthesis	N/A	N/A	N/A	N/A	N/A	N/A	N/A
Mismatch Repair in Eukaryotes	N/A	N/A	N/A	N/A	N/A	N/A	N/A
Regulation of eIF4 and p70S6K Signaling	N/A	N/A	N/A	N/A	N/A	N/A	N/A
Arginine Degradation VI (Arginase 2 Pathway)	N/A	N/A	N/A	N/A	N/A	N/A	N/A
Valine Degradation I	N/A	N/A	N/A	N/A	N/A	N/A	N/A
Hypoxia Signaling in the Cardiovascular System	N/A	N/A	N/A	N/A	N/A	N/A	N/A
LPS/IL-1 Mediated Inhibition of RXR Function	N/A	N/A	N/A	N/A	N/A	N/A	N/A
Noradrenaline and Adrenaline Degradation	N/A	N/A	N/A	N/A	N/A	N/A	N/A
Vitamin-C Transport	N/A	N/A	N/A	N/A	N/A	N/A	N/A
Role of JAK family kinases in IL-6-type Cytokine Signaling	N/A	N/A	N/A	N/A	N/A	N/A	N/A
Gap Junction Signaling	N/A	N/A	N/A	N/A	N/A	N/A	N/A

Î³-linolenate Biosynthesis II (Animals)	N/A	N/A	N/A	N/A	N/A	N/A	N/A
S-adenosyl-L-methionine Biosynthesis	N/A	N/A	N/A	N/A	N/A	N/A	N/A
SPINK1 General Cancer Pathway	N/A	N/A	N/A	N/A	N/A	N/A	N/A
Molecular Mechanisms of Cancer	N/A	N/A	N/A	N/A	N/A	N/A	N/A
Circadian Rhythm Signaling	N/A	N/A	N/A	N/A	N/A	N/A	N/A
Endometrial Cancer Signaling	N/A	N/A	N/A	N/A	N/A	N/A	N/A
Adenine and Adenosine Salvage VI	N/A	N/A	N/A	N/A	N/A	N/A	N/A
2-oxobutanoate Degradation I	N/A	N/A	N/A	N/A	N/A	N/A	N/A
Cellular Effects of Sildenafil (Viagra)	N/A	N/A	N/A	N/A	N/A	N/A	N/A
Ethanol Degradation II	N/A	N/A	N/A	N/A	N/A	N/A	N/A
IL-17A Signaling in Airway Cells	N/A	N/A	N/A	N/A	N/A	N/A	N/A
Role of Macrophages, Fibroblasts and Endothelial Cells in Rheumatoid	N/A	N/A	N/A	N/A	N/A	N/A	N/A
PXR/RXR Activation	N/A	N/A	N/A	N/A	0.0	N/A	N/A
GABA Receptor Signaling	N/A	N/A	N/A	N/A	N/A	N/A	N/A
4-hydroxyproline Degradation I	N/A	N/A	N/A	N/A	N/A	N/A	N/A
Fatty Acid Î²-oxidation III (Unsaturated, Odd Number)	N/A	N/A	N/A	N/A	N/A	N/A	N/A
Glucocorticoid Receptor Signaling	N/A	N/A	N/A	N/A	N/A	N/A	N/A
Glutathione Redox Reactions II	N/A	N/A	N/A	N/A	N/A	N/A	N/A
4-1BB Signaling in T Lymphocytes	N/A	N/A	N/A	N/A	N/A	N/A	N/A
Glutamine Biosynthesis I	N/A	N/A	N/A	N/A	N/A	N/A	N/A
UVA-Induced MAPK Signaling	N/A	N/A	N/A	N/A	0.0	N/A	N/A
Arginine Degradation I (Arginase Pathway)	N/A	N/A	N/A	N/A	N/A	N/A	N/A
Alanine Degradation III	N/A	N/A	N/A	N/A	N/A	N/A	N/A
Tryptophan Degradation III (Eukaryotic)	N/A	N/A	N/A	N/A	N/A	N/A	N/A
D-myo-inositol (3,4,5,6)-tetrakisphosphate Biosynthesis	N/A	0.0	N/A	N/A	0.0	N/A	N/A
Î²-alanine Degradation I	N/A	N/A	N/A	N/A	N/A	N/A	N/A
Thio-molybdenum Cofactor Biosynthesis	N/A	N/A	N/A	N/A	N/A	N/A	N/A
Role of Tissue Factor in Cancer	N/A	N/A	N/A	N/A	N/A	N/A	N/A
Urate Biosynthesis/Inosine 5'-phosphate Degradation	N/A	N/A	N/A	N/A	N/A	N/A	N/A
VDR/RXR Activation	N/A	N/A	N/A	N/A	N/A	N/A	N/A
L-cysteine Degradation I	N/A	N/A	N/A	N/A	N/A	N/A	N/A
Role of JAK2 in Hormone-like Cytokine Signaling	N/A	N/A	N/A	N/A	N/A	N/A	N/A
GDP-L-fucose Biosynthesis I (from GDP-D-mannose)	N/A	N/A	N/A	N/A	N/A	N/A	N/A
MYC Mediated Apoptosis Signaling	N/A	N/A	N/A	N/A	N/A	N/A	N/A
Role of Osteoblasts, Osteoclasts and Chondrocytes in Rheumatoid Arth	N/A	N/A	N/A	N/A	N/A	N/A	N/A
Superpathway of Citrulline Metabolism	N/A	N/A	N/A	N/A	N/A	N/A	N/A
UVC-Induced MAPK Signaling	N/A	N/A	N/A	N/A	0.0	N/A	N/A
Cell Cycle Regulation by BTG Family Proteins	N/A	N/A	N/A	N/A	N/A	N/A	N/A
Dopamine Degradation	N/A	N/A	N/A	N/A	N/A	N/A	N/A
Dopamine Receptor Signaling	N/A	N/A	N/A	N/A	N/A	N/A	N/A
Lactose Degradation III	N/A	N/A	N/A	N/A	N/A	N/A	N/A
D-mannose Degradation	N/A	N/A	N/A	N/A	N/A	N/A	N/A
Fatty Acid Biosynthesis Initiation II	N/A	N/A	N/A	N/A	N/A	N/A	N/A
Clathrin-mediated Endocytosis Signaling	N/A	N/A	N/A	N/A	N/A	N/A	N/A
Hepatic Cholestasis	N/A	N/A	N/A	N/A	N/A	N/A	N/A

Glycine Degradation (Creatine Biosynthesis)	N/A	N/A	N/A	N/A	N/A	N/A	N/A
Calcium Transport I	N/A	N/A	N/A	N/A	N/A	N/A	N/A
Phenylalanine Degradation IV (Mammalian, via Side Chain)	N/A	N/A	N/A	N/A	N/A	N/A	N/A
Glioma Invasiveness Signaling	N/A	N/A	N/A	N/A	N/A	N/A	N/A
Superpathway of Geranylgeranyldiphosphate Biosynthesis I (via Meval	N/A	N/A	N/A	N/A	N/A	N/A	N/A
Prostate Cancer Signaling	N/A	N/A	N/A	N/A	N/A	N/A	N/A
Purine Nucleotides Degradation II (Aerobic)	N/A	N/A	N/A	N/A	N/A	N/A	N/A
Glutathione Redox Reactions I	N/A	N/A	N/A	N/A	N/A	N/A	N/A
Glutamine Degradation I	N/A	N/A	N/A	N/A	N/A	N/A	N/A
Leptin Signaling in Obesity	N/A	N/A	N/A	N/A	N/A	N/A	N/A
Role of JAK1 and JAK3 in Î³c Cytokine Signaling	N/A	N/A	N/A	N/A	N/A	N/A	N/A
Telomerase Signaling	N/A	N/A	N/A	N/A	N/A	N/A	N/A
Heme Biosynthesis from Uroporphyrinogen-III I	N/A	N/A	N/A	N/A	N/A	N/A	N/A
ERBB4 Signaling	N/A	N/A	N/A	N/A	0.0	N/A	N/A
Gluconeogenesis I	N/A	N/A	N/A	N/A	N/A	N/A	N/A
Arginine Biosynthesis IV	N/A	N/A	N/A	N/A	N/A	N/A	N/A
Superpathway of D-myo-inositol (1,4,5)-trisphosphate Metabolism	N/A	N/A	N/A	N/A	N/A	N/A	N/A
G Protein Signaling Mediated by Tubby	N/A	N/A	N/A	N/A	N/A	N/A	N/A
Ketolysis	N/A	N/A	N/A	N/A	N/A	N/A	N/A
Methylglyoxal Degradation III	N/A	N/A	N/A	N/A	N/A	N/A	N/A
Ascorbate Recycling (Cytosolic)	N/A	N/A	N/A	N/A	N/A	N/A	N/A
Induction of Apoptosis by HIV1	N/A	N/A	N/A	N/A	N/A	N/A	N/A
4-aminobutyrate Degradation I	N/A	N/A	N/A	N/A	N/A	N/A	N/A
Aspartate Degradation II	N/A	N/A	N/A	N/A	N/A	N/A	N/A
Glutaryl-CoA Degradation	N/A	N/A	N/A	N/A	N/A	N/A	N/A
D-myo-inositol (1,4,5)-trisphosphate Degradation	N/A	N/A	N/A	N/A	N/A	N/A	N/A
Stearate Biosynthesis I (Animals)	N/A	N/A	N/A	N/A	N/A	N/A	N/A
Glutamate Biosynthesis II	N/A	N/A	N/A	N/A	N/A	N/A	N/A
Thiosulfate Disproportionation III (Rhodanese)	N/A	N/A	N/A	N/A	N/A	N/A	N/A
VEGF Signaling	N/A	N/A	N/A	N/A	N/A	N/A	N/A
Iron homeostasis signaling pathway	N/A	N/A	N/A	N/A	N/A	N/A	N/A
Sertoli Cell-Sertoli Cell Junction Signaling	N/A	N/A	N/A	N/A	N/A	N/A	N/A
FAT10 Signaling Pathway	N/A	N/A	N/A	N/A	N/A	N/A	N/A
Asparagine Degradation I	N/A	N/A	N/A	N/A	N/A	N/A	N/A
DNA Double-Strand Break Repair by Homologous Recombination	N/A	N/A	N/A	N/A	N/A	N/A	N/A
Sonic Hedgehog Signaling	N/A	N/A	N/A	N/A	N/A	N/A	N/A
Glycerol-3-phosphate Shuttle	N/A	N/A	N/A	N/A	N/A	N/A	N/A
Cancer Drug Resistance By Drug Efflux	N/A	N/A	N/A	N/A	N/A	N/A	N/A
Histidine Degradation III	N/A	N/A	N/A	N/A	N/A	N/A	N/A
Colanic Acid Building Blocks Biosynthesis	N/A	N/A	N/A	N/A	N/A	N/A	N/A
Leukotriene Biosynthesis	N/A	N/A	N/A	N/A	N/A	N/A	N/A
Î³-glutamyl Cycle	N/A	N/A	N/A	N/A	N/A	N/A	N/A
TNFR1 Signaling	N/A	N/A	N/A	N/A	N/A	N/A	N/A
Cell Cycle: G1/S Checkpoint Regulation	N/A	N/A	N/A	N/A	0.0	N/A	N/A
April Mediated Signaling	N/A	N/A	N/A	N/A	N/A	N/A	N/A

Putrescine Degradation III	N/A	N/A	N/A	N/A	N/A	N/A	N/A
Myo-inositol Biosynthesis	N/A	N/A	N/A	N/A	N/A	N/A	N/A
Pyrimidine Deoxyribonucleotides De Novo Biosynthesis I	N/A	N/A	N/A	N/A	N/A	N/A	N/A
IL-2 Signaling	N/A	N/A	N/A	N/A	N/A	N/A	N/A
Protein Ubiquitination Pathway	N/A	N/A	N/A	N/A	N/A	N/A	N/A
Tumoricidal Function of Hepatic Natural Killer Cells	N/A	N/A	N/A	N/A	N/A	N/A	N/A
Urea Cycle	N/A	N/A	N/A	N/A	N/A	N/A	N/A
NAD Biosynthesis from 2-amino-3-carboxymuconate Semialdehyde	N/A	N/A	N/A	N/A	N/A	N/A	N/A
FcÎ³RIIB Signaling in B Lymphocytes	N/A	N/A	N/A	N/A	0.0	N/A	N/A
Axonal Guidance Signaling	N/A	N/A	N/A	N/A	N/A	N/A	N/A
Methylthiopropionate Biosynthesis	N/A	N/A	N/A	N/A	N/A	N/A	N/A
Maturity Onset Diabetes of Young (MODY) Signaling	N/A	N/A	N/A	N/A	N/A	N/A	N/A
Sucrose Degradation V (Mammalian)	N/A	N/A	N/A	N/A	N/A	N/A	N/A
Pyrimidine Ribonucleotides De Novo Biosynthesis	N/A	N/A	N/A	N/A	N/A	N/A	N/A
Ketogenesis	N/A	N/A	N/A	N/A	N/A	N/A	N/A
VEGF Family Ligand-Receptor Interactions	N/A	N/A	N/A	N/A	0.0	N/A	N/A
Acetyl-CoA Biosynthesis III (from Citrate)	N/A	N/A	N/A	N/A	N/A	N/A	N/A
Formaldehyde Oxidation II (Glutathione-dependent)	N/A	N/A	N/A	N/A	N/A	N/A	N/A
Folate Transformations I	N/A	N/A	N/A	N/A	N/A	N/A	N/A
BER (Base Excision Repair) Pathway	N/A	N/A	N/A	N/A	N/A	N/A	N/A
GDP-mannose Biosynthesis	N/A	N/A	N/A	N/A	N/A	N/A	N/A
RAN Signaling	N/A	N/A	N/A	N/A	N/A	N/A	N/A
Semaphorin Signaling in Neurons	N/A	N/A	N/A	N/A	N/A	N/A	N/A
Mitochondrial Dysfunction	N/A	N/A	N/A	N/A	N/A	N/A	N/A
Cholesterol Biosynthesis II (via 24,25-dihydrolanosterol)	N/A	N/A	N/A	N/A	N/A	N/A	N/A
Inosine-5'-phosphate Biosynthesis II	N/A	N/A	N/A	N/A	N/A	N/A	N/A
Phenylethylamine Degradation I	N/A	N/A	N/A	N/A	N/A	N/A	N/A
D-myo-inositol (1,4,5,6)-Tetrakisphosphate Biosynthesis	N/A	0.0	N/A	N/A	0.0	N/A	N/A
Biotin-carboxyl Carrier Protein Assembly	N/A	N/A	N/A	N/A	N/A	N/A	N/A
Virus Entry via Endocytic Pathways	N/A	N/A	N/A	N/A	N/A	N/A	N/A
Xenobiotic Metabolism Signaling	N/A	N/A	N/A	N/A	N/A	N/A	N/A
Ephrin B Signaling	N/A	N/A	N/A	N/A	N/A	N/A	N/A
IL-22 Signaling	N/A	N/A	N/A	N/A	N/A	N/A	N/A
Sulfate Activation for Sulfonation	N/A	N/A	N/A	N/A	N/A	N/A	N/A
Tetrahydrofolate Salvage from 5,10-methenyltetrahydrofolate	N/A	N/A	N/A	N/A	N/A	N/A	N/A
Glutathione-mediated Detoxification	N/A	N/A	N/A	N/A	N/A	N/A	N/A
5-aminoimidazole Ribonucleotide Biosynthesis I	N/A	N/A	N/A	N/A	N/A	N/A	N/A
Role of JAK1, JAK2 and TYK2 in Interferon Signaling	N/A	N/A	N/A	N/A	N/A	N/A	N/A
Spermine Biosynthesis	N/A	N/A	N/A	N/A	N/A	N/A	N/A
Isoleucine Degradation I	N/A	N/A	N/A	N/A	N/A	N/A	N/A
Role of IL-17F in Allergic Inflammatory Airway Diseases	N/A	N/A	N/A	N/A	N/A	N/A	N/A
Amyloid Processing	N/A	N/A	N/A	N/A	N/A	N/A	N/A
Purine Nucleotides De Novo Biosynthesis II	N/A	N/A	N/A	N/A	N/A	N/A	N/A
Role of CHK Proteins in Cell Cycle Checkpoint Control	N/A	N/A	N/A	N/A	N/A	N/A	N/A
Methylmalonyl Pathway	N/A	N/A	N/A	N/A	N/A	N/A	N/A

Chronic Myeloid Leukemia Signaling	N/A	N/A	N/A	N/A	N/A	N/A	N/A
Proline Biosynthesis II (from Arginine)	N/A	N/A	N/A	N/A	N/A	N/A	N/A
Rapoport-Luebering Glycolytic Shunt	N/A	N/A	N/A	N/A	N/A	N/A	N/A
Glutathione Biosynthesis	N/A	N/A	N/A	N/A	N/A	N/A	N/A
Cholesterol Biosynthesis III (via Desmosterol)	N/A	N/A	N/A	N/A	N/A	N/A	N/A
Selenocysteine Biosynthesis II (Archaea and Eukaryotes)	N/A	N/A	N/A	N/A	N/A	N/A	N/A
Alanine Biosynthesis II	N/A	N/A	N/A	N/A	N/A	N/A	N/A
Remodeling of Epithelial Adherens Junctions	N/A	N/A	N/A	N/A	N/A	N/A	N/A
Leucine Degradation I	N/A	N/A	N/A	N/A	N/A	N/A	N/A
Heme Degradation	N/A	N/A	N/A	N/A	N/A	N/A	N/A
Superoxide Radicals Degradation	N/A	N/A	N/A	N/A	N/A	N/A	N/A
Melanoma Signaling	N/A	N/A	N/A	N/A	N/A	N/A	N/A
Mechanisms of Viral Exit from Host Cells	N/A	N/A	N/A	N/A	N/A	N/A	N/A
Mitotic Roles of Polo-Like Kinase	N/A	N/A	N/A	N/A	N/A	N/A	N/A
Choline Biosynthesis III	N/A	N/A	N/A	N/A	N/A	N/A	N/A
Proline Degradation	N/A	N/A	N/A	N/A	N/A	N/A	N/A
Phosphatidylethanolamine Biosynthesis II	N/A	N/A	N/A	N/A	N/A	N/A	N/A
Epoxysqualene Biosynthesis	N/A	N/A	N/A	N/A	N/A	N/A	N/A
Eumelanin Biosynthesis	N/A	N/A	N/A	N/A	N/A	N/A	N/A
Cholesterol Biosynthesis I	N/A	N/A	N/A	N/A	N/A	N/A	N/A
Phagosome Maturation	N/A	N/A	N/A	N/A	N/A	N/A	N/A
Serotonin Degradation	N/A	N/A	N/A	N/A	N/A	N/A	N/A
Galactose Degradation I (Leloir Pathway)	N/A	N/A	N/A	N/A	N/A	N/A	N/A
Aspartate Biosynthesis	N/A	N/A	N/A	N/A	N/A	N/A	N/A
Ethanol Degradation IV	N/A	N/A	N/A	N/A	N/A	N/A	N/A
Mitochondrial L-carnitine Shuttle Pathway	N/A	N/A	N/A	N/A	N/A	N/A	N/A
Tight Junction Signaling	N/A	N/A	N/A	N/A	N/A	N/A	N/A
Trans, trans-farnesyl Diphosphate Biosynthesis	N/A	N/A	N/A	N/A	N/A	N/A	N/A
L-cysteine Degradation III	N/A	N/A	N/A	N/A	N/A	N/A	N/A
Hypusine Biosynthesis	N/A	N/A	N/A	N/A	N/A	N/A	N/A
IL-10 Signaling	N/A	N/A	N/A	N/A	N/A	N/A	N/A
RAR Activation	N/A	N/A	N/A	N/A	N/A	N/A	N/A
L-carnitine Biosynthesis	N/A	N/A	N/A	N/A	N/A	N/A	N/A
Coronavirus Replication Pathway	N/A	N/A	N/A	N/A	N/A	N/A	N/A
Palmitate Biosynthesis I (Animals)	N/A	N/A	N/A	N/A	N/A	N/A	N/A
Fatty Acid Activation	N/A	N/A	N/A	N/A	N/A	N/A	N/A
Alanine Biosynthesis III	N/A	N/A	N/A	N/A	N/A	N/A	N/A
