# Supplementary material for: Fetal Zone Steroids Show Discrete Effects on Hyperoxia-Induced Attenuation of Migration in Cultured Oligodendrocyte Progenitor Cells
Source: Oxid Med Cell Longev. 2022 May 9;2022:2606880. doi: 10.1155/2022/2606880 (PMC9110221; doi:10.1155/2022/2606880)
Supplement: Supplementary Materials — Figure S1 (supplementary figure 1): Ki67 staining of OLN93 cells post-24 hours of normoxic and hyperoxic (80% O2) treatments. Representative immunofluorescence images of OLN93 cells stained for Ki67 proliferation marker. Upper panel represents images taken post-24 hours of normoxic treatment. Lower panel represents images taken post-24 hours of hyperoxic treatment. Scale bar represents 75 μm. Data are representative of three individual experiments. Figure S2 (supplementary figure 2): changes in specific migration-related proteins post treatments. Intensities of (a) Hmox1, (b) PAK1, (c) RAF1, and (d) Cdc42ep4 plotted from the mass spectrometry results. Graphs show the changes in protein intensities upon different treatment conditions. Data are representative of five independent experiments. Bars and error represent mean ± SEM of replicate measurements. ∗ represents statistically significant differences in comparison to normoxic control, # represents statistically significant differences in comparison to hyperoxic control, and § represents statistically significant differences between normoxic and hyperoxic treatments within the same group. Single signs represent a p value < 0.05, double signs represent p < 0.01, triple signs represent p < 0.001, and quadruple signs represent p < 0.0001(Student's t-test). Figure S3 (supplementary figure 3): complete heat map of canonical pathway analysis of significantly enriched proteins in the OLN93 cells post cotreatment of adiol+E2 in comparison to normoxic and hyperoxic controls using IPA. Negative z-score values are indicated in blue, and positive z-score values are indicated in red. Cutoff p value < 0.05 (Fisher's exact test). Table T1 (supplementary table 1): details of mass spectrometry procedure. (a) LC-MS/MS parameter (data independent mode; quantitative data). (b) Spectronaut parameters for peptide/protein identification and intensity extraction. Table T2 (supplementary table 2): functional categorization of proteins with [file 2606880.f1.zip › Supplemental Table T3.pdf]

Supplemental Table S3. Functional categorization of proteins with differential abundance under normoxic and hyperoxic conditions and prediction of activity of "Diseases and Functions".
Proteins with p< 0.05 were considered. Categories are sorted by ascending activation score Z.

© 2000-2022 QIAGEN. All rights reserved.

Categories	Diseases or Functions Annotation	p-value	Predicted Activation State	Activation z-score	Molecules	# Molecules
Cellular Assembly and Organization,Cellular Function and Maintenance	Microtubule dynamics	1,79E-07	Decreased	-4,081	ACACA,ADI1,Akap9,APPL2,ATXN10,C1QBP,CAMSAP2,CANX,CARMIL1,CDC42EP4,CDK1,CSRP1,CYFIP1,DOCK1,DOCK5,DPYSL2,DPYSL3,DYNC2H1,EML1,EXOC2,EXOC4,FASN,FLNA,FUT8,GDA,GTSE1,HDGFL3,HMMR,IFT74,INPPL1,ITPR1,KATNB1,KIFBP,MARK2,MTSS1,NDC80,NPTXR,NQO1,OGT,PAFAH1B1,PAK1,PDE4DIP,PRKACB,PRKCA,RAF1,RO60,SH3GLB1,SLC9A3R1,SLC9A3R2,SNX1,SRGAP3,STAT3,TLN1,TRAPPC4,TRIM3,TRIOBP,TRIP10,TTC21B,UBAP2L,UBE2I,USP8,USP9X,VAMP7,WIPF1	64
Cellular Assembly and Organization,Cellular Function and Maintenance	Organization of cytoplasm	9,55E-14	Decreased	-3,796	ACACA,ACP2,ADD1,ADI1,AGFG1,Akap9,APPL2,ARFGEF2,ARHGEF1,ARHGEF18,ATXN10,C1QBP,CAMSAP2,CANX,CARMIL1,CCDC47,CDC42EP4,CDK1,CERT1,CNN2,CSNK1A1,CSRP1,CYFIP1,DCTN6,DOCK1,DOCK5,DPYSL2,DPYSL3,DYNC2H1,EMD,EML1,EXOC2,EXOC4,FASN,FLII,FLNA,FLNB,FUT8,GABPA,GDA,GTSE1,HDGFL3,HIKESHI,HMGCL,HMMR,IFT74,INPPL1,ITPR1,KANK1,KATNB1,KIFBP,LIMD1,MARK2,MKLN1,MTSS1,NDC80,NPTXR,NQO1,OGT,PAFAH1B1,PAK1,PDE4DIP,PRKACB,PRKCA,PTPN1,RAB32,RAB6A,RAF1,RO60,SEC61A1,SH3GLB1,SLC9A3R1,SLC9A3R2,SNX1,SPP1,SQSTM1,SRGAP3,STAT3,TLN1,TRAPPC4,TRIM3,TRIOBP,TRIP10,TTC21B,UBAP2L,UBE2I,USP8,USP9X,VAMP7,VCPIP1,VPS4B,WDR62,WIPF1	93
Cellular Assembly and Organization,Cellular Function and Maintenance	Organization of cytoskeleton	2,67E-10	Decreased	-3,796	ACACA,ADD1,ADI1,AGFG1,Akap9,APPL2,ARHGEF1,ARHGEF18,ATXN10,C1QBP,CAMSAP2,CANX,CARMIL1,CDC42EP4,CDK1,CNN2,CSNK1A1,CSRP1,CYFIP1,DCTN6,DOCK1,DOCK5,DPYSL2,DPYSL3,DYNC2H1,EML1,EXOC2,EXOC4,FASN,FLII,FLNA,FLNB,FUT8,GDA,GTSE1,HDGFL3,HMMR,IFT74,INPPL1,ITPR1,KANK1,KATNB1,KIFBP,LIMD1,MARK2,MKLN1,MTSS1,NDC80,NPTXR,NQO1,OGT,PAFAH1B1,PAK1,PDE4DIP,PRKACB,PRKCA,PTPN1,RAF1,RO60,SH3GLB1,SLC9A3R1,SLC9A3R2,SNX1,SPP1,SRGAP3,STAT3,TLN1,TRAPPC4,TRIM3,TRIOBP,TRIP10,TTC21B,UBAP2L,UBE2I,USP8,USP9X,VAMP7,WDR62,WIPF1	79
Cell Death and Survival		3,05E-10	Decreased	-3,489	ACACA,AGA,AK3,APPL2,ARFGEF2,BIRC6,CCDC47,CDK1,CDK4,CDK6,CSNK1A1,CUL3,DDX5,DNMT1,DOCK1,DTYMK,EEF2,EEF2K,EMC7,EXOC2,FADD,FASN,FLII,FLNA,FUT8,GCLC,GLRX,HMGCS1,HMOX1,HMOX2,HNRNPUL1,IDH2,INPP1,ITPR1,ITPR3,LAMTOR3,MAT2A,MTDH,MTSS1,NCLN,NDC80,NFKB2,NFS1,NRBP1,NUP54,OGT,PAK1,PARG,PFKM,PMPCB,POLD1,PPAT,PRDX2,PRKACB,PRKCA,PRPF19,PTPN1,RAD51,RAF1,RBPJ,RPA1,RPL27,RPN2,RRM1,RRM2,SFR1,SPP1,SQLE,SQSTM1,STAM2,STAT1,STAT3,STAT5B,TM9SF3,TNFAIP8,TRIM28,UBE2I,USP24,USP47,USP8,USP9X,VCP,VPS13A,WFS1,WIPF1,XRN1	86
Cell Death and Survival	Cell viability	3,55E-10	Decreased	-3,218	ACACA,AGA,AK3,APPL2,ARFGEF2,BIRC6,CCDC47,CDK1,CDK6,CSNK1A1,CUL3,DDX5,DNMT1,DOCK1,DTYMK,EEF2,EEF2K,EMC7,EXOC2,FADD,FASN,FLNA,FUT8,GCLC,HMGCS1,HMOX1,HMOX2,HNRNPUL1,IDH2,INPP1,ITPR1,ITPR3,LAMTOR3,MAT2A,MTDH,MTSS1,NCLN,NDC80,NFKB2,NFS1,NRBP1,NUP54,OGT,PAK1,PARG,PFKM,PMPCB,POLD1,PPAT,PRDX2,PRKACB,PRKCA,PRPF19,PTPN1,RAD51,RAF1,RBPJ,RPA1,RPL27,RPN2,RRM1,RRM2,SFR1,SPP1,SQLE,SQSTM1,STAM2,STAT1,STAT3,STAT5B,TM9SF3,TNFAIP8,TRIM28,UBE2I,USP24,USP47,USP8,USP9X,VCP,VPS13A,WFS1,WIPF1,XRN1	83
Lipid Metabolism,Small Molecule Biochemistry	Conversion of fatty acid	0,0000478	Decreased	-2,607	ACACA,ACSL3,ACSL4,CRAT,FADS1,FASN,LTA4H	7
Cell Cycle	Interphase	0,0000149	Decreased	-2,48	Anp32b,CDK1,CDK4,CDK6,CUL3,DDB1,DGKZ,DNMT1,EEF2K,FADD,FASN,FBXL15,FLNA,HAUS7,HMOX1,MTDH,NEK9,OGT,PAFAH1B1,PAK1,PARG,PDE4DIP,PHLDA1,PLCB1,POLA1,PRKCA,PRPF19,RAD51,RAF1,RNASEH2B,RPA1,RPA2,RPL5,RPL7A,SPP1,STAT1,STAT3,USP24,USP8,VCP	40
Cell Death and Survival	Cell viability of tumor cell lines	4,07E-08	Decreased	-2,413	ACACA,AGA,AK3,ARFGEF2,BIRC6,CDK6,CSNK1A1,DOCK1,EEF2,EEF2K,FADD,FASN,FLNA,GCLC,HMOX1,HNRNPUL1,IDH2,INPP1,ITPR3,LAMTOR3,MTDH,MTSS1,NDC80,NFKB2,NRBP1,PAK1,PARG,PFKM,PMPCB,POLD1,PPAT,PRDX2,PRKACB,PRKCA,PRPF19,PTPN1,RAD51,RAF1,RPA1,RPL27,RRM1,RRM2,SFR1,SPP1,SQSTM1,STAT3,STAT5B,TM9SF3,TNFAIP8,TRIM28,UBE2I,USP24,USP47,USP8,VCP,WFS1,XRN1	57
Cancer,Dermatological Diseases and Conditions,Organismal Injury and Abnormalities	Skin tumor	0,00000599	Decreased	-2,373	AARSD1,ABCD4,ACO1,ACO2,ACP2,ACSF2,ACSL3,ACSL4,ADD1,ADI1,AGA,AGFG1,AGL,AKR1B1,AKR1B10,ALDH2,ALDH4A1,ALDH6A1,ALDH9A1,ANAPC7,ANKRD13A,ANKRD46,ANXA4,APPL2,ARFGAP2,ARFGAP3,ARFGEF2,ARHGEF1,ARHGEF12,ARHGEF18,ARPIN/ARPIN-AP3S2,ASRGL1,ATIC,ATP2A2,ATP2B4,ATP6AP1,ATP6V1A,ATPAF2,ATXN10,BCAS1,BCAT2,BIRC6,CAMSAP2,CAND1,CANX,CARMIL1,CAVIN1,CDC37L1,CDK1,CDK4,CDK6,CEP55,CNEP1R1,CNN2,CNPY2,CRAT,CSNK1A1,CSRP1,CTBP2,CTTNBP2,CUL3,CYB5R1,DDB1,DDRGK1,DDX5,DGKZ,DGLUCY,DNAJC5,DNMT1,DNPEP,DOCK1,DOCK5,DPY19L1,DPYSL2,DPYSL3,DYNC2H1,ECI2,EEF1B2,EEF2,EEF2K,EIF2B4,ELOA,ELP3,EML1,ERLIN2,EXOC2,EXTL3,FADD,FAF2,FASN,FBXL15,FLII,FLNA,FLNB,FRMD4A,FUT8,GATM,GCDH,GCLC,GDA,GMDS,GMPPB,GOT2,GPT2,GRB14,GTSE1,HAUS7,HDGFL3,HMGCL,HMGCS1,HMOX2,HNRNPUL1,IDH2,IKBIP,INPP1,INPPL1,IRGQ,ITPR1,ITPR3,ITPRID2,KANK1,KANK2,KATNB1,KPNA2,LARP7,LAYN,LETM1,LIMD1,LPP,LRRC40,LTA4H,MARK2,MAT2A,MEGF10,MKLN1,MMUT,MON2,MPI,MPST,MTSS1,NAA25,NAB2,NAPA,NAXE,NCLN,NEK9,NFKB2,NFS1,NPTXR,NQO1,NRDC,OAT,OSBPL8,PAK1,PAPSS1,PCBP2,PDE4DIP,PEPD,PGD,PHLDA1,PHYH,PIGU,PITRM1,PLCB1,POLA1,POLD1,POLD3,POLDIP3,PPOX,PPP4R3A,PRKACB,PRKCA,PRPF19,PRRC2A,PRRX1,QSOX2,RAB32,RAD51,RAF1,RAI14,RANGAP1,RASSF4,RBPJ,RIOK3,RPA1,RPL13,RPL3,RPL5,RPN1,RPN2,RRM1,RRM2,SAMD4B,SEC14L1,SEC24A,SEC24D,SEC61A1,SERPINE2,SFR1,SGTA,SLC1A4,SOAT1,SPP1,SQLE,SRGAP3,STARD3NL,STAT1,STAT3,STAT5B,STRN4,STT3A,SUCLG2,TAX1BP1,TCF12,TDRKH,TKFC,TLE3,TLN1,TM9SF4,TMEM43,TNS3,TOM1L2,TRIM2,TRIM28,TRIM3,TRIM47,TRIOBP,TRIP12,TRIP13,TRMT2A,TST,TTC21B,UBAP2L,UBE2O,UBR7,USP24,USP47,USP8,USP9X,VAMP7,VAT1,VCP,VPS11,VPS13A,VPS4B,VWA5A,WDR12,WDR45B,WDR62,WFS1,XRN1	251
Cell Death and Survival,Renal and Urological System Development and Function	Cell viability of kidney cell lines	0,000342	Decreased	-2,36	CCDC47,EMC7,FADD,FUT8,NCLN,PAK1,PFKM,PRDX2,VPS13A	9
Cellular Development,Cellular Growth and Proliferation,Organ Development,Skeletal and Muscular System Development and Function,Tissue Development	Proliferation of smooth muscle cells	0,000303	Decreased	-2,314	AKR1B1,ATP2A2,CDK4,CDK6,CSNK1A1,EMD,HMOX1,NQO1,PAK1,PRKCA,PRRX1,PTPN1,RBPJ,SPP1,STAT1,STAT3,STAT5B	17
Nucleic Acid Metabolism	Metabolism of nucleic acid component or derivative	4,39E-10	Decreased	-2,249	ACAA2,ACACA,ACO2,ACSF2,AK3,ALDH2,ALDH6A1,ANXA4,ATIC,CARMIL1,CDK1,DNMT1,DPYSL2,DTYMK,FASN,FUT8,GCDH,GDA,GFPT1,GMDS,GMPPB,HMGCL,HMOX1,IDH2,LETM1,MPI,NQO1,PAPSS1,PGD,PPAT,RRM1,RRM2,SH3GLB1,SLC9A3R1,SNX1,SOAT1,SPP1,STAT3,SUCLG2,VCP	40
Nucleic Acid Metabolism,Small Molecule Biochemistry	Synthesis of nucleotide	0,00000258	Decreased	-2,249	ACO2,AK3,ALDH2,ANXA4,CDK1,DTYMK,FASN,GMDS,GMPPB,HMOX1,IDH2,LETM1,MPI,PAPSS1,PGD,PPAT,RRM1,RRM2,SH3GLB1,SLC9A3R1,SNX1,SOAT1,SPP1,STAT3,VCP	25
Nucleic Acid Metabolism,Small Molecule Biochemistry	Metabolism of nucleotide	0,00000825	Decreased	-2,249	ACO2,AK3,ALDH2,ANXA4,CDK1,DTYMK,FASN,FUT8,GDA,GMDS,GMPPB,HMOX1,IDH2,LETM1,MPI,NQO1,PAPSS1,PGD,PPAT,RRM1,RRM2,SH3GLB1,SLC9A3R1,SNX1,SOAT1,SPP1,STAT3,VCP	28
Lipid Metabolism,Small Molecule Biochemistry	Synthesis of lipid	0,0000119	Decreased	-2,247	ACACA,ACO2,ACSL3,ACSL4,AKR1B1,CDIPT,CDK4,CERT1,CNEP1R1,CTBP2,DGKZ,ERLIN2,FADS1,FAF2,FASN,FDFT1,GCDH,GRB14,HACD3,HMGCS1,HMOX1,HMOX2,IMPA1,INPPL1,LPCAT3,LTA4H,MGST2,PGD,PIGU,PLCB1,PPAT,PRDX2,PRKCA,PRPF19,SERPINE2,SH3GLB1,SLC9A3R1,SLC9A3R2,SOAT1,SPP1,SQLE,STAT3,STAT5B	43
Lipid Metabolism,Nucleic Acid Metabolism,Small Molecule Biochemistry	Conversion of acyl-coenzyme A	0,0000142	Decreased	-2,215	ACACA,ACSL3,ACSL4,CRAT,FASN	5
Cellular Assembly and Organization,Cellular Function and Maintenance	Growth of microtubules	0,000135	Decreased	-2,156	CAMSAP2,DOCK5,MARK2,PAK1,PDE4DIP	5
Cellular Movement	Migration of cells	0,00000168	Decreased	-2,106	ACACA,ACSL4,ADD1,ADI1,APPL2,ARFGAP3,ARFGEF2,ARHGEF1,ARHGEF12,ARPIN/ARPIN-AP3S2,ATP2A2,ATP2B4,C1QBP,CANX,CARMIL1,CDK1,CDK4,CDK6,CERT1,CNN2,CNN3,CTBP2,DDRGK1,DNMT1,DOCK1,DOCK5,DPYSL2,DPYSL3,ELP3,FASN,FLNA,FLNB,FUT8,GLRX,GRB14,GSDMD,GTSE1,HMMR,HMOX1,IKBIP,INPPL1,ITPR1,KANK1,KPNA2,LAMTOR2,LAYN,LPP,Mapre2,MARK2,MEGF10,MTDH,MTSS1,NAA25,NAB2,NARS1,NFKB2,NQO1,NRDC,OSBPL8,PAFAH1B1,PAK1,PARG,PDE4DIP,PHLDA1,PRDX2,PRKCA,PRRX1,PTPN1,RAF1,RAP2C,RIOK3,RRM1,SERPINE2,SLC9A3R1,SLC9A3R2,SNX17,SORBS3,SPP1,STAT1,STAT3,STAT5B,TAGLN2,TAX1BP1,TCF12,TLN1,TNFAIP8,TNS3,TPD52,TRIM2,TRIP10,UBE2I,USP9X,VAMP7,VCP,WIPF1	95
Lipid Metabolism,Molecular Transport,Small Molecule Biochemistry	Concentration of lipid	0,0000418	Decreased	-2,021	ACACA,ACO2,ACSL3,ACSL4,Akap9,AKR1B1,AKR1B10,Anp32b,ATP2A2,BCAT2,CDK4,CERT1,CNEP1R1,CNPY2,CRAT,DDX5,DGKZ,FADD,FASN,FDFT1,GATM,HMOX1,INPPL1,ITPR1,ITPRID2,LPCAT3,LTA4H,MARK2,NQO1,OGT,OSBPL8,PAFAH1B1,PCK2,PHYH,PRKCA,PRPF19,PTPN1,SEC24A,SLC9A3R1,SOAT1,SPP1,SRGAP3,STAT3	43
Cellular Assembly and Organization,Cellular Function and Maintenance,Tissue Development	Formation of microtubules	0,0000346		-1,942	CAMSAP2,DOCK5,DPYSL2,DPYSL3,GDA,HDGFL3,INPPL1,PAFAH1B1,PAK1,UBAP2L	10
Cellular Compromise,Inflammatory Response	Degranulation of phagocytes	2,33E-07		-1,937	AGA,AGL,ARMC8,CAND1,CNN2,CYFIP1,DGKZ,DNAJC5,DOCK5,EEF2,FAF2,GHDC,GSDMD,HEBP2,HMOX1,INPPL1,LAMTOR2,LAMTOR3,LTA4H,NIT2,PAK1,RAB6A,RAP2C,SLC9A3R1,SLC9A3R2,STAT5B,VAMP7,VAT1,VCP,WIPF1	30
Cellular Compromise,Inflammatory Response	Degranulation of myeloid cells	3,14E-07		-1,937	AGA,AGL,ARMC8,CAND1,CNN2,CYFIP1,DGKZ,DNAJC5,DOCK5,EEF2,FAF2,GHDC,GSDMD,HEBP2,HMOX1,INPPL1,LAMTOR2,LAMTOR3,LTA4H,NIT2,PAK1,RAB6A,RAP2C,SLC9A3R1,SLC9A3R2,STAT5B,VAMP7,VAT1,VCP,WIPF1	30
Cellular Development,Cellular Growth and Proliferation	Cell proliferation of tumor cell lines	0,0000103		-1,909	ACACA,ACO2,ACSL4,AGA,AKR1B10,ATP2A2,ATP6AP1,BIRC6,C1QBP,CACYBP,CDK1,CDK4,CDK6,CNN2,CSNK1A1,CTBP2,DDB1,DDX5,DNMT1,DPYSL3,DTYMK,EEF1B2,EEF2K,EIF2B5,EXOC2,EXTL3,FADD,FASN,FBXL15,FDFT1,FLNA,FUT8,HMGCS1,HMMR,HMOX1,IDH2,ITPR1,KPNA2,MPI,MTDH,NAB2,NFKB2,NFS1,NQO1,OGT,PAFAH1B1,PAK1,PARG,PCK2,PDE4DIP,PGD,PHLDA1,PRDX2,PRKACB,PRKCA,PTPN1,RAD51,RAF1,RASSF4,RRM1,RRM2,SFR1,SH3GLB1,SLC9A3R1,SNX1,SPP1,SQSTM1,STAT1,STAT3,STAT5B,TAGLN2,TCF12,TNFAIP8,TNS3,TPD52,TRIM2,TRIM28,UBE2C,UBE2I,USP8,USP9X,VCP,WDR12	83
Inflammatory Response	Degranulation	1,22E-08		-1,894	AGA,AGL,ARMC8,CAND1,CDC37L1,CNN2,CYB5R1,CYFIP1,DGKZ,DNAJC5,DOCK5,EEF2,EXOC2,FAF2,FLNA,GHDC,GLRX,GSDMD,HEBP2,HMOX1,INPPL1,LAMTOR2,LAMTOR3,LTA4H,NIT2,PAK1,RAB6A,RAP2C,SLC9A3R1,SLC9A3R2,STAM2,STAT5B,TAGLN2,TLN1,VAMP7,VAT1,VCP,WIPF1	38
Cellular Movement	Cell movement of sarcoma cell lines	0,000014		-1,83	FASN,FLNA,FLNB,GTSE1,NRDC,PTPN1,RAF1,SLC9A3R1,SLC9A3R2,TLN1,VAMP7,VCP	12
Cell Morphology	Ruffling	0,0000301		-1,826	ADD1,CYFIP1,EXOC2,FLNA,FLNB,HMOX1,INPPL1,MTSS1,PAK1,SPP1	10
Cellular Development,Cellular Growth and Proliferation,Nervous System Development and Function,Tissue Development	Proliferation of neuronal cells	0,0000124		-1,776	CDK1,CNPY2,CSRP1,CYFIP1,DOCK1,DPYSL2,DPYSL3,EML1,EXOC4,EXTL3,HDGFL3,HMGCS1,IMPA1,INPP1,KIFBP,MARK2,OGT,PAFAH1B1,PAK1,PRKCA,RAF1,RBPJ,SERPINE2,SNX1,SPP1,SRGAP3,STAT3,TRIM3,USP47,VAMP7,WDR62	31
DNA Replication, Recombination, and Repair	Repair of DNA	0,0000585		-1,775	CDK1,DDB1,DTYMK,FASN,HMOX1,KPNA2,PARG,POLA1,POLD1,POLD3,PRPF19,RAD51,RNASEH2B,RPA1,RPA2,RRM1,TRIM28,TRIP13,UBE2I,USP47,VCP,ZMPSTE24	22
Cellular Assembly and Organization	Organization of organelle	0,0000553		-1,715	ACP2,AGFG1,ARFGEF2,ATP2A2,CARMIL1,CCDC47,CERT1,CSNK1A1,CTTNBP2,DPYSL2,DYNC2H1,EMD,FLNA,GABPA,HIKESHI,HMGCL,INPPL1,KIFBP,MTSS1,NDC80,PAFAH1B1,RAB32,RAB6A,RPA1,SEC61A1,SH3GLB1,SLC9A3R1,SQSTM1,USP8,VCP,VCPIP1,VPS4B,ZMPSTE24	33
Cell Cycle	Cell cycle progression	0,000137		-1,674	BIRC6,C1QBP,CBX3,CDK1,CDK4,CDK6,CSNK1A1,CUL3,DGKZ,DNMT1,ELOA,FADD,FASN,FLNA,GTSE1,HAUS7,HMOX1,LAMTOR2,LAMTOR3,LETM1,NDC80,NEK9,NFKB2,NLE1,OGT,PAFAH1B1,PAK1,PKN2,POLA1,POLD1,PRKCA,PTPN1,RAF1,RASSF4,RPA1,RRM1,SLC9A3R1,SPP1,STAT1,STAT3,STAT5B,UBE2C,UBE2I,VCP,VCPIP1,WDR12,WDR62,ZMPSTE24	48
Cell Death and Survival	Survival of stem cell lines	0,000203		-1,667	DTYMK,HMGCS1,MAT2A,NFS1,NUP54,RPA1,RPN2,SQLE,TRIM28	9
Cellular Movement	Cell movement	8,97E-07		-1,649	ACACA,ACO2,ACSL4,ADD1,ADI1,AKR1B1,ALDH2,APPL2,ARFGAP3,ARFGEF2,ARHGEF1,ARHGEF12,ARPIN/ARPIN-AP3S2,ATP2A2,ATP2B4,C1QBP,CANX,CARMIL1,CDC42EP4,CDK1,CDK4,CDK6,CERT1,CNN2,CNN3,CTBP2,DDRGK1,DNMT1,DOCK1,DOCK5,DPYSL2,DPYSL3,ELP3,EMD,FADD,FASN,FLNA,FLNB,FUT8,GLRX,GRB14,GSDMD,GTSE1,HMMR,HMOX1,IKBIP,INPPL1,ITPR1,KANK1,KPNA2,LAMTOR2,LAYN,LPP,Mapre2,MARK2,MEGF10,MTDH,MTSS1,NAA25,NAB2,NARS1,NFKB2,NQO1,NRDC,OSBPL8,PAFAH1B1,PAK1,PARG,PDE4DIP,PHLDA1,PRDX2,PRKCA,PRRX1,PTPN1,RAF1,RAP2C,RIOK3,RRM1,SERPINE2,SLC9A3R1,SLC9A3R2,SNX17,SORBS3,SPP1,SQSTM1,STAT1,STAT3,STAT5B,TAGLN2,TAX1BP1,TCF12,TLN1,TNFAIP8,TNS3,TPD52,TRIM2,TRIM3,TRIP10,UBE2I,USP9X,VAMP7,VCP,VPS13A,WIPF1	104
Infectious Diseases	Viral Infection	5,57E-08		-1,632	AGFG1,AIP,ALDH9A1,ARHGEF12,ATP2A2,ATP6AP1,ATP6V1A,CARMIL1,CDK4,CERT1,CTBP2,CUL3,CYFIP1,DDB1,DDX5,DNMT1,DYNC2H1,EEF2,EIF2B5,ELOA,FADD,FADS1,FASN,FDFT1,FLII,FLNA,GCLC,GPT2,GSDMD,HDGFL3,HMGCS1,HMOX1,ITPR1,KATNB1,KPNA2,LTA4H,MARK2,MAT2A,MMUT,MON2,NEK9,NQO1,NRBP1,NUP54,OGT,PAK1,PCBP2,PFKM,PKN2,PLCB1,POLA1,POLD1,PRDX2,PRKCA,PTPN1,RAB32,RAB6A,RAF1,RBPJ,RO60,RPL3,RPL5,RRM1,RRM2,SDF4,SEC14L1,SLC9A3R1,SOAT1,SPCS3,SPP1,SQSTM1,STARD3NL,STAT1,STAT3,STAT5B,STT3A,SUCLG2,TAGLN2,TKFC,UBE2C,UBE2I,VCP,VPS11,VPS4B,WIPF1,XRN1,ZMPSTE24	87
Cellular Movement	Migration of sarcoma cell lines	0,0000719		-1,578	FASN,FLNA,FLNB,GTSE1,NRDC,RAF1,SLC9A3R1,SLC9A3R2,VAMP7,VCP	10
Cellular Development,Cellular Growth and Proliferation,Nervous System Development and Function,Tissue Development	Growth of neurites	0,0000663		-1,557	CDK1,CNPY2,CSRP1,CYFIP1,DOCK1,DPYSL2,DPYSL3,EXOC4,EXTL3,HDGFL3,HMGCS1,IMPA1,INPP1,KIFBP,MARK2,OGT,PAFAH1B1,PAK1,RAF1,SERPINE2,SNX1,SRGAP3,STAT3,TRIM3,USP47,VAMP7	26
Molecular Transport	Transport of molecule	1,53E-07		-1,529	ABCD4,ACACA,ACADS,ACO1,ACSL3,ACSL4,AGFG1,AIP,AP3S2,APPL2,ARFGEF2,ARHGEF12,ATP2A2,ATP2B4,ATP6AP1,ATP6V1A,CAND1,CANX,CAVIN1,CDK1,CDK4,CERT1,CRAT,CUL3,CYB5R1,DDX5,DGKZ,DNAJC5,DPYSL2,EXOC4,FADD,FBXO7,FLNA,FRMD4A,GOT2,HIKESHI,HMOX1,HMOX2,ITPR1,ITPR3,KPNA2,LETM1,LPCAT3,MTDH,NAPA,NAXE,NUP54,OGT,OSBPL8,PAFAH1B1,PCK2,PITRM1,PMPCB,POLDIP3,PRKCA,PTPN1,RAB6A,RANGAP1,SEC14L1,SEC24A,SLC1A4,SLC9A3R1,SLC9A3R2,SNX1,SOAT1,SQSTM1,STAT1,STAT3,TMEM120A,TPD52,TRAPPC4,TRIM28,UBAP2L,UBE2I,VAMP7,VPS11,VPS4B,WFS1	78
Cellular Assembly and Organization	Development of cytoplasm	1,1E-08		-1,52	ACO2,ADD1,ANKRD13A,ARHGEF1,ARHGEF12,ARHGEF18,ATP2A2,CAMSAP2,CARMIL1,CDK4,CDK6,CERT1,CUL3,Cyb5r3,DOCK5,DPYSL2,DPYSL3,FLNA,GDA,HDGFL3,INPPL1,KANK1,KANK2,LETM1,MARK2,MTSS1,PAFAH1B1,PAK1,PKN2,SORBS3,STAT3,TNS3,TRIP10,UBAP2L,VCP,WDR45B,WIPF1	37
Tissue Development	Growth of nervous tissue	0,0000179		-1,497	APPL2,CDK1,CDK4,CDK6,CNPY2,CSRP1,CYFIP1,DNMT1,DOCK1,DPYSL2,DPYSL3,EML1,EXOC4,EXTL3,FLNA,HDGFL3,HMGCS1,IDH2,IMPA1,INPP1,KIFBP,MARK2,OGT,PAFAH1B1,PAK1,PRKCA,RAF1,RBPJ,SERPINE2,SNX1,SPP1,SRGAP3,STAT3,TRIM3,USP47,VAMP7,WDR62	37

Categories	Diseases or Functions Annotation	p-value	Predicted Activation State	Activation z-score	Molecules	# Molecules
Lipid Metabolism,Small Molecule Biochemistry	Fatty acid metabolism	1,8E-08		-1,444	ACAA2,ACACA,ACADS,ACSF2,ACSL3,ACSL4,AKR1B1,ARHGEF12,CANX,CERT1,CRAT,CUL3,ECI2,ERLIN2,FADD,FADS1,FASN,GCDH,GOT2,GRB14,HACD3,HMGCL,HMOX1,HMOX2,LPCAT3,LTA4H,MGST2,NAXE,NQO1,OSBPL8,PRKCA,SEC24A,SLC9A3R1,SLC9A3R2,SOAT1,STAT3,STAT5B,SUCLG2,VAMP7,VPS4B	40
Cellular Compromise,Inflammatory Response	Degranulation of cells	9,65E-08		-1,441	AGA,AGL,ARMC8,CAND1,CDC37L1,CNN2,CYB5R1,CYFIP1,DGKZ,DNAJC5,DOCK5,EEF2,EXOC2,FAF2,FLNA,GHDC,GSDMD,HEBP2,HMOX1,INPPL1,LAMTOR2,LAMTOR3,LTA4H,NIT2,PAK1,RAB6A,RAP2C,SLC9A3R1,SLC9A3R2,STAT5B,TAGLN2,TLN1,VAMP7,VAT1,VCP,WIPF1	36
Cellular Assembly and Organization,Tissue Development	Fibrogenesis	0,0000137		-1,422	ADD1,ARHGEF1,ARHGEF12,ARHGEF18,CAMSAP2,CARMIL1,CDK4,CDK6,CERT1,CUL3,Cyb5r3,DOCK5,DPYSL2,DPYSL3,GDA,HDGFL3,INPPL1,KANK1,KANK2,MARK2,MTSS1,PAFAH1B1,PAK1,PKN2,SORBS3,STAT3,TNS3,TRIP10,UBAP2L	29
Infectious Diseases	Infection by RNA virus	0,000196		-1,39	AIP,ALDH9A1,ARHGEF12,CYFIP1,DDX5,DNMT1,DYNC2H1,EEF2,EIF2B5,ELOA,FADD,FADS1,FDFT1,FLII,FLNA,GPT2,GSDMD,HMGCS1,MAT2A,MMUT,NEK9,NQO1,NRBP1,PAK1,PCBP2,PFKM,PKN2,PLCB1,POLA1,POLD1,PRKCA,RAB6A,RPL3,RPL5,RRM1,RRM2,SDF4,SEC14L1,SOAT1,SPCS3,SPP1,STARD3NL,STAT1,STT3A,SUCLG2,TAGLN2,UBE2C,VCP,VPS11,ZMPSTE24	50
Molecular Transport,Protein Trafficking	Import of protein	9,57E-07		-1,342	AIP,APPL2,CDK1,DDX5,FBXO7,FLNA,HIKESHI,KPNA2,NUP54,PITRM1,RANGAP1,SQSTM1,STAT3,TRIM28	14
Cell Morphology,Cellular Movement	Cell spreading	0,000199		-1,337	CNN2,DOCK1,DOCK5,FLNA,FLNB,INPPL1,LPP,PAK1,RAF1,RAP2C,SORBS3,SPP1,STAT3,TLN1,TNS3,TRIM3,VAMP7	17
Gene Expression	Expression of RNA	0,000173		-1,302	ACO1,ANXA4,ATP2B4,C1QBP,CAND1,CAVIN1,CBX3,CC2D1B,CDK1,CDK4,CDK6,CNN2,CNPY2,CSNK1A1,CTBP2,CUL3,CYFIP1,DDB1,DDRGK1,DDX5,DNMT1,EIF2B4,EIF2B5,ELOA,ELP1,ELP2,ELP3,FADD,FLII,FLNA,FUT8,GABPA,GCDH,GCLC,GTF2B,Hmgb3,IFT74,INPPL1,KANK2,KPNA2,LARP7,LIMD1,LOXL3,MTDH,NAB2,Nelfe,NFKB2,NQO1,NRBP1,OGT,PAK1,PCBP2,PFKM,PKN2,PLCB1,POLA1,PPP2R5D,PRKCA,PRRX1,RAF1,RBPJ,RNASEH2B,RO60,RPL5,RPL6,SEC61A1,SFR1,SNF8,SORBS3,SPP1,SQSTM1,STAT1,STAT3,STAT5B,TCF12,TLE3,TRIM28,TRIP13,TTC21B,UBE2I,USP47,USP9X,WFS1,XRN1,ZMPSTE24	85
Carbohydrate Metabolism	Gluconeogenesis	0,000164		-1,246	GATM,GOT2,GPT2,HMGCL,ITPR1,NLN,NQO1,OGT,PCK2,PPP4R3A	10
Cancer,Neurological Disease,Organismal Injury and Abnormalities	Nervous system neoplasm	0,0000163		-1,231	ABCD4,ACADS,ACP2,ACSF2,ADD1,AGA,AGFG1,AIP,AKR1B1,AKR1B10,ALDH2,ALDH9A1,ANAPC7,AP3S2,APPL2,ARFGAP2,ARFGAP3,ARFGEF2,ARHGEF1,ARHGEF18,ARPIN/ARPIN-AP3S2,ASRGL1,ATIC,ATP2A2,ATP2B4,ATP6AP1,ATP6V1A,ATPAF2,ATXN10,BCAS1,BCAT2,BIRC6,CACYBP,CAMSAP2,CAND1,CANX,CARMIL1,CC2D1B,CCDC47,CDC42EP4,CDK1,CDK4,CDK6,CEP55,CNN2,CNN3,CRAT,CSRP1,CTBP2,CTTNBP2,CUL3,CWF19L1,CYFIP1,DDB1,DDRGK1,DDX5,DGKZ,DNMT1,DNPEP,DOCK1,DOCK5,DPY19L1,DYNC2H1,EEF2K,EIF2B4,EIF2B5,ELP1,ELP2,ELP3,ERLIN2,EXOC4,FADD,FASN,FBXL15,FBXO7,FLII,FLNA,FLNB,FUT8,GABPA,GCDH,GCLC,GCLM,GDA,GFPT1,GMDS,GMPPB,GOT2,GPT2,GRB14,GSDMD,GTSE1,HDGFL3,HEBP2,HMGCS1,HMMR,HMOX1,IDH2,INPPL1,ITPR1,ITPR3,ITPRID2,KANK1,KANK2,KATNB1,KPNA2,LETM1,LPP,LRRC40,MARK2,MEGF10,MKLN1,MMUT,MON2,MPI,MTDH,MTSS1,NAA25,NCLN,NFKB2,NIT2,NLE1,NLN,NRBP1,NRDC,NT5C3B,OGT,OXCT1,PAFAH1B1,PAK1,PAPSS1,PCBP2,PCK2,PDE4DIP,PDZRN3,PEPD,PFKM,PHLDA1,PHYH,PIP4P2,PITRM1,PKN2,PLCB1,POLA1,POLD1,POLD3,PPAT,PPP4R3A,PRKCA,PRRC2A,PTPN1,QSOX2,RAB32,RABGGTA,RAF1,RANGAP1,RASSF4,RBPJ,RPA1,RPA2,RPL5,RPN1,RPN2,RRM1,RRM2,SAMD4B,SDF4,SEC14L1,SEC24A,SEC24D,SH3GLB1,SLC1A4,SLC9A3R1,SNX1,SNX17,SOAT1,SORBS3,SPCS3,SPP1,SQSTM1,SRGAP3,STARD3NL,STAT1,STAT3,STAT5B,STRN4,STT3A,TAX1BP1,TCF12,TDRKH,TLE3,TLN1,TM9SF4,TMEM43,TNS3,TOM1L2,TPD52,TRIM2,TRIM28,TRIOBP,TRIP10,TRIP12,UBE2C,UBE2O,UBR7,USP24,USP47,USP8,USP9X,VCP,VCPIP1,VPS13A,VPS36,VWA5A,WDR62,WFS1,WIPF1,XRN1	218
Cellular Assembly and Organization,Tissue Development	Formation of filaments	0,00000356		-1,211	ADD1,ARHGEF1,ARHGEF12,ARHGEF18,CAMSAP2,CARMIL1,CDK4,CDK6,CUL3,Cyb5r3,DOCK5,DPYSL2,DPYSL3,GDA,HDGFL3,INPPL1,KANK1,KANK2,MARK2,MTSS1,PAFAH1B1,PAK1,PKN2,SORBS3,STAT3,TNS3,TRIP10,UBAP2L	28
Nucleic Acid Metabolism,Small Molecule Biochemistry	Synthesis of purine nucleotide	0,00000314		-1,199	ACO2,AK3,ALDH2,CDK1,FASN,GMDS,GMPPB,HMOX1,IDH2,LETM1,MPI,PGD,PPAT,SH3GLB1,SOAT1,STAT3,VCP	17
Cellular Assembly and Organization	Formation of cytoskeleton	5,52E-08		-1,188	ADD1,ARHGEF1,ARHGEF12,ARHGEF18,CAMSAP2,CARMIL1,CDK4,CDK6,CUL3,Cyb5r3,DOCK5,DPYSL2,DPYSL3,FLNA,GDA,HDGFL3,INPPL1,KANK1,KANK2,MARK2,MTSS1,PAFAH1B1,PAK1,PKN2,SORBS3,STAT3,TNS3,TRIP10,UBAP2L,WIPF1	30
Cancer,Neurological Disease,Organismal Injury and Abnormalities	Glioma	0,0000149		-1,172	ABCD4,ACADS,ACP2,ACSF2,ADD1,AGA,AGFG1,AIP,AKR1B1,AKR1B10,ALDH2,ALDH9A1,ANAPC7,AP3S2,APPL2,ARFGAP2,ARFGAP3,ARFGEF2,ARHGEF1,ARHGEF18,ARPIN/ARPIN-AP3S2,ASRGL1,ATIC,ATP2A2,ATP2B4,ATP6AP1,ATP6V1A,ATPAF2,ATXN10,BCAS1,BCAT2,BIRC6,CACYBP,CAMSAP2,CAND1,CANX,CARMIL1,CC2D1B,CCDC47,CDC42EP4,CDK1,CDK4,CDK6,CEP55,CNN2,CNN3,CRAT,CSRP1,CTBP2,CTTNBP2,CUL3,CWF19L1,CYFIP1,DDB1,DDRGK1,DDX5,DNMT1,DNPEP,DOCK1,DOCK5,DPY19L1,DYNC2H1,EEF2K,EIF2B4,EIF2B5,ELP1,ELP2,ELP3,ERLIN2,EXOC4,FADD,FASN,FBXL15,FBXO7,FLII,FLNA,FLNB,FUT8,GABPA,GCLC,GCLM,GDA,GFPT1,GMDS,GMPPB,GPT2,GRB14,GSDMD,GTSE1,HDGFL3,HEBP2,HMGCS1,HMMR,HMOX1,IDH2,INPPL1,ITPR1,ITPR3,ITPRID2,KANK1,KANK2,KATNB1,KPNA2,LETM1,LPP,LRRC40,MARK2,MEGF10,MKLN1,MMUT,MON2,MPI,MTDH,MTSS1,NAA25,NCLN,NFKB2,NIT2,NLE1,NLN,NRBP1,NRDC,NT5C3B,OGT,OXCT1,PAFAH1B1,PAK1,PAPSS1,PCBP2,PCK2,PDE4DIP,PDZRN3,PEPD,PFKM,PHLDA1,PHYH,PIP4P2,PITRM1,PKN2,PLCB1,POLA1,POLD1,PPAT,PPP4R3A,PRRC2A,PTPN1,QSOX2,RAB32,RABGGTA,RAF1,RANGAP1,RASSF4,RBPJ,RPA1,RPL5,RPN1,RPN2,RRM1,RRM2,SAMD4B,SDF4,SEC14L1,SEC24A,SEC24D,SH3GLB1,SLC1A4,SLC9A3R1,SNX1,SNX17,SOAT1,SORBS3,SPCS3,SPP1,SQSTM1,SRGAP3,STARD3NL,STAT1,STAT3,STAT5B,STRN4,STT3A,TAX1BP1,TCF12,TDRKH,TLE3,TLN1,TM9SF4,TMEM43,TNS3,TOM1L2,TPD52,TRIM2,TRIM28,TRIOBP,TRIP10,TRIP12,UBE2C,UBE2O,UBR7,USP24,USP47,USP8,USP9X,VCP,VCPIP1,VPS13A,VPS36,VWA5A,WDR62,WFS1,WIPF1,XRN1	212
Cancer,Neurological Disease,Organismal Injury and Abnormalities	Central nervous system solid tumor	0,0000206		-1,172	ABCD4,ACADS,ACP2,ACSF2,ADD1,AGA,AGFG1,AIP,AKR1B1,AKR1B10,ALDH2,ALDH9A1,ANAPC7,AP3S2,APPL2,ARFGAP2,ARFGAP3,ARFGEF2,ARHGEF1,ARHGEF18,ARPIN/ARPIN-AP3S2,ASRGL1,ATIC,ATP2A2,ATP2B4,ATP6AP1,ATP6V1A,ATPAF2,ATXN10,BCAS1,BCAT2,BIRC6,CACYBP,CAMSAP2,CAND1,CANX,CARMIL1,CC2D1B,CCDC47,CDC42EP4,CDK1,CDK4,CDK6,CEP55,CNN2,CNN3,CRAT,CSRP1,CTBP2,CTTNBP2,CUL3,CWF19L1,CYFIP1,DDB1,DDRGK1,DDX5,DGKZ,DNMT1,DNPEP,DOCK1,DOCK5,DPY19L1,DYNC2H1,EEF2K,EIF2B4,EIF2B5,ELP1,ELP2,ELP3,ERLIN2,EXOC4,FADD,FASN,FBXL15,FBXO7,FLII,FLNA,FLNB,FUT8,GABPA,GCDH,GCLC,GCLM,GDA,GFPT1,GMDS,GMPPB,GOT2,GPT2,GRB14,GSDMD,GTSE1,HDGFL3,HEBP2,HMGCS1,HMMR,HMOX1,IDH2,INPPL1,ITPR1,ITPR3,ITPRID2,KANK1,KANK2,KATNB1,KPNA2,LETM1,LPP,LRRC40,MARK2,MEGF10,MKLN1,MMUT,MON2,MPI,MTDH,MTSS1,NAA25,NCLN,NFKB2,NIT2,NLE1,NLN,NRBP1,NRDC,NT5C3B,OGT,OXCT1,PAFAH1B1,PAK1,PAPSS1,PCBP2,PCK2,PDE4DIP,PDZRN3,PEPD,PFKM,PHLDA1,PHYH,PIP4P2,PITRM1,PKN2,PLCB1,POLA1,POLD1,PPAT,PPP4R3A,PRKCA,PRRC2A,PTPN1,QSOX2,RAB32,RABGGTA,RAF1,RANGAP1,RASSF4,RBPJ,RPA1,RPL5,RPN1,RPN2,RRM1,RRM2,SAMD4B,SDF4,SEC14L1,SEC24A,SEC24D,SH3GLB1,SLC1A4,SLC9A3R1,SNX1,SNX17,SOAT1,SORBS3,SPCS3,SPP1,SQSTM1,SRGAP3,STARD3NL,STAT1,STAT3,STAT5B,STRN4,STT3A,TAX1BP1,TCF12,TDRKH,TLE3,TLN1,TM9SF4,TMEM43,TNS3,TOM1L2,TPD52,TRIM2,TRIM28,TRIOBP,TRIP10,TRIP12,UBE2C,UBE2O,UBR7,USP24,USP47,USP8,USP9X,VCP,VCPIP1,VPS13A,VPS36,VWA5A,WDR62,WFS1,WIPF1,XRN1	216
Cellular Assembly and Organization,Cellular Function and Maintenance	Formation of vesicles	0,0000129		-1,126	AGFG1,ARFGEF2,BIRC6,FLNA,SERPINE2,SH3GLB1,SNF8,SOAT1,SPP1,SQSTM1,STAM2,VAMP7,VPS36,VPS4B	14
Cancer,Organismal Injury and Abnormalities	Melanoma	0,00000256		-1,091	AARSD1,ABCD4,ACAA2,ACADS,ACO1,ACO2,ACP2,ACSF2,ACSL3,ACSL4,ADD1,ADI1,AGA,AGFG1,AGL,AKR1B1,AKR1B10,ALDH2,ALDH4A1,ALDH6A1,ALDH9A1,ANAPC7,ANKRD13A,ANKRD46,ANXA4,APPL2,ARFGAP2,ARFGAP3,ARFGEF2,ARHGEF1,ARHGEF12,ARHGEF18,ARPIN/ARPIN-AP3S2,ASRGL1,ATIC,ATP2A2,ATP6AP1,ATP6V1A,ATPAF2,BCAS1,BCAT2,BIRC6,C17orf49,C1QBP,CAMSAP2,CAND1,CANX,CARMIL1,CAVIN1,CC2D1B,CDC37L1,CDC42EP4,CDK1,CDK4,CEP55,CNEP1R1,CNN2,CNN3,CNPY2,CRAT,CSNK1A1,CSRP1,CTBP2,CTTNBP2,CUL3,DDB1,DDRGK1,DGKZ,DGLUCY,DNAJC5,DNMT1,DNPEP,DOCK1,DOCK5,DPY19L1,DPYSL2,DPYSL3,DYNC2H1,ECI2,EEF1B2,EEF2,EEF2K,EIF2B4,EIF2B5,ELOA,ELP3,EMC7,EML1,ERLIN2,EXOC2,EXTL3,FADD,FASN,FLII,FLNA,FLNB,FRMD4A,FUT8,GATM,GCDH,GCLC,GDA,GFPT1,GHDC,GMDS,GMPPB,GOT2,GPT2,GRB14,GTSE1,HAUS7,HDGFL3,HMGCL,HMGCS1,HMOX1,HMOX2,HNRNPUL1,HSPA4L,IDH2,IKBIP,INPPL1,IRGQ,ITPR1,ITPR3,ITPRID2,KANK1,KANK2,KATNB1,KPNA2,LARP7,LAYN,LIMD1,LOXL3,LPP,LTA4H,MARK2,MAT2A,MEGF10,MKLN1,MMUT,MON2,MPI,MPST,MTSS1,NAA25,NAB2,NAPA,NAXE,NCLN,NDUFAF7,NEK9,NFKB2,NFS1,NIT2,NPTXR,NRDC,NUP54,OAT,OGT,OSBPL8,PAFAH1B1,PAK1,PAPSS1,PCBP2,PDE4DIP,PEPD,PGD,PHLDA1,PHYH,PIGU,PITRM1,PLCB1,POLA1,POLD1,POLDIP3,PPP4R3A,PRKACB,PRKCA,PRRC2A,PRRX1,QSOX2,RAB32,RAD51,RAF1,RAI14,RANGAP1,RASSF4,RBPJ,RER1,RETSAT,RIOK3,RPA1,RPL13,RPL3,RPL5,RPN1,RPN2,RRM1,RRM2,SAMD4B,SEC14L1,SEC24A,SEC24D,SEC61A1,SERPINE2,SFR1,SH3GLB1,SLC1A4,SORBS3,SPP1,SQLE,SRGAP3,STAT1,STAT3,STAT5B,STT3A,TCF12,TDRKH,TKFC,TLE3,TLN1,TM9SF3,TM9SF4,TMEM43,TNS3,TOM1L2,TRIM2,TRIM28,TRIM3,TRIM47,TRIOBP,TRIP12,TRIP13,TRMT2A,TST,TTC21B,UBAP2L,UBE2O,UBR7,USP24,USP47,USP8,USP9X,VAMP7,VAT1,VCP,VPS11,VPS13A,VPS4B,VWA5A,WDR12,WDR45B,WDR62,WFS1,XRN1,ZMPSTE24	256
Cancer,Organismal Injury and Abnormalities	Growth of tumor	0,0000359		-1,087	ACO2,ACSL4,AGA,AKR1B1,AKR1B10,CACYBP,CDK1,CDK4,CDK6,CYFIP1,DDRGK1,DNMT1,FADD,FASN,FLNA,FUT8,GSTZ1,HMMR,HMOX1,IDH2,LAYN,LIMD1,MTDH,NFKB2,OGT,PAK1,PARG,PDE4DIP,PFKM,PGD,PHLDA1,PRKCA,RAD51,RAF1,RBPJ,RIOK3,RRM1,RRM2,SLC9A3R2,SPP1,SQSTM1,STAT1,STAT3,STAT5B,TLN1,TPD52,TRIM2,UBE2C,USP9X,VCP	50
Cellular Movement	Cell movement of tumor cell lines	0,0000668		-1,079	ACSL4,ARFGEF2,ARHGEF1,C1QBP,CERT1,CTBP2,DNMT1,DOCK1,DOCK5,DPYSL2,DPYSL3,FADD,FASN,FLNA,FLNB,GTSE1,HMMR,HMOX1,INPPL1,ITPR1,KANK1,KPNA2,MTDH,MTSS1,NRDC,PAK1,PHLDA1,PRDX2,PRKCA,PRRX1,PTPN1,RAF1,RAP2C,RIOK3,SLC9A3R1,SLC9A3R2,SPP1,SQSTM1,STAT1,STAT3,TAGLN2,TCF12,TLN1,TNFAIP8,TNS3,TPD52,TRIM2,TRIM3,TRIP10,UBE2I,USP9X,VAMP7,VCP	53
Lipid Metabolism,Small Molecule Biochemistry,Vitamin and Mineral Metabolism	Synthesis of cholesterol	0,0000484		-1,067	ACACA,ERLIN2,FASN,FDFT1,HMGCS1,LPCAT3,SOAT1,SPP1,SQLE	9
Gene Expression	Transcription of RNA	0,00016		-1,066	ANXA4,ATP2B4,C1QBP,CAND1,CAVIN1,CBX3,CC2D1B,CDK1,CDK4,CDK6,CTBP2,CUL3,DDB1,DDRGK1,DDX5,DNMT1,EIF2B5,ELOA,ELP1,ELP2,ELP3,FADD,FLNA,GABPA,GCLC,GTF2B,IFT74,INPPL1,KANK2,KPNA2,LARP7,LIMD1,LOXL3,MTDH,NAB2,Nelfe,NFKB2,NRBP1,OGT,PCBP2,PFKM,PKN2,PLCB1,POLA1,PPP2R5D,PRKCA,PRRX1,RAF1,RBPJ,RO60,RPL6,SEC61A1,SFR1,SNF8,SORBS3,SPP1,SQSTM1,STAT1,STAT3,STAT5B,TCF12,TLE3,TRIM28,TRIP13,TTC21B,UBE2I,USP47,USP9X,WFS1,ZMPSTE24	70
Cell Death and Survival	Neuronal cell death	0,000112		-1,016	AGA,CDK1,CDK4,CDK6,DNAJC5,DPYSL3,DTYMK,ELP1,ELP3,FADD,FBXO7,GCDH,GCLC,GCLM,GDA,GLRX,HMGCS1,HMOX1,HMOX2,ITPR1,MAT2A,MTSS1,NFS1,NQO1,OGT,PAFAH1B1,PAK1,PRDX2,PRKCA,SERPINE2,SH3GLB1,SQLE,STAT1,STAT3,TRIM2,TRIP10,WFS1	37
Cancer,Organismal Injury and Abnormalities	Tumorigenesis of epithelial neoplasm	1,29E-10		-0,971	AARSD1,ABCD4,ACADS,ACO1,ACO2,ACSF2,ACSL3,ACSL4,ADD1,AGL,AIP,AK3,AKR1B1,AKR1B10,AKR7A2,ALDH2,ALDH4A1,ALDH6A1,ANKRD13A,ANXA4,AP3S2,APPL2,ARFGAP2,ARFGAP3,ARFGEF2,ARHGEF1,ARHGEF12,ARHGEF18,ARMC8,ASRGL1,ATIC,ATP2A2,ATP6AP1,ATXN10,BCAT2,BIRC6,C1QBP,CACYBP,CAMSAP2,CAND1,CANX,CAVIN1,CBX3,CC2D1B,CCDC47,CDIPT,CDK1,CDK4,CDK6,CEP55,CERT1,CNPY2,CRAT,CSNK1A1,CTBP2,CTTNBP2,CUL3,CYB5R1,CYFIP1,DCTN6,DDB1,DDRGK1,DDX5,DGKZ,DGLUCY,DNAJC5,DNMT1,DOCK1,DOCK5,DPY19L1,DPYSL2,DPYSL3,DTYMK,DYNC2H1,EEF1B2,EEF2,EEF2K,EIF2B4,ELOA,ELP1,ELP2,ELP3,EMC7,EMD,EML1,ERLIN2,EXOC2,EXOC4,EXTL3,FAF2,FASN,FBXO7,FDFT1,FLII,FLNA,FLNB,FRMD4A,FUT8,GATM,GCDH,GCLM,GFPT1,GHDC,GLRX3,GLRX5,GMDS,GMPPB,GOT2,GPT2,GRB14,GSTZ1,GTF2B,GTSE1,HAUS7,HDGFL3,HEBP2,HM13,HMMR,HMOX1,HNRNPUL1,HSPA4L,IDH2,IFT74,IKBIP,IMPA1,INPPL1,IRGQ,ISCA2,ITPR1,ITPR3,ITPRID2,KANK1,KPNA2,LARP7,LAYN,LETM1,LIMD1,LOXL3,LPP,LRRC40,LTA4H,MARK2,MEGF10,MKLN1,MMUT,MON2,MTDH,MTSS1,NAA25,NAB2,NAPA,NCLN,NDC80,NEK9,NFKB2,NLE1,NMD3,NPTXR,NQO1,NRBP1,NRDC,NT5C3B,NUP54,OAT,OGT,OSBPL8,OXCT1,PAFAH1B1,PAK1,PAPSS1,PARG,PCBP2,PCK2,PDE4DIP,PDZRN3,PEPD,PFKM,PHLDA1,PHYH,PIGU,PITRM1,PKN2,PLCB1,POLA1,POLD1,POLD3,POLDIP3,PPAT,PPOX,PPP2R5D,PPP4R3A,PRDX2,PRKACB,PRKCA,PRPF19,PRRC2A,PRRX1,PTPN1,QSOX2,RAB32,RAD51,RAF1,RAI14,RANGAP1,RBPJ,RCN2,RER1,RETSAT,RIOK3,RNASEH2B,RPA1,RPL5,RPN1,RPN2,RRM1,RRM2,SAMD4B,SDF4,SEC14L1,SEC24A,SEC24D,SEC61A1,SERPINE2,SLC1A4,SLC9A3R1,SLC9A3R2,SNF8,SNX1,SNX17,SOAT1,SORBS3,SPP1,SQLE,SQSTM1,SRGAP3,STAT1,STAT3,STAT5B,STT3A,SUCLG2,TAX1BP1,TCF12,TDRKH,TKFC,TLN1,TM9SF3,TM9SF4,TMEM115,TMEM43,TNS3,TOM1L2,TPD52,TRIM2,TRIM28,TRIM3,TRIM47,TRIOBP,TRIP10,TRIP12,TRIP13,TRMT2A,UBAP2L,UBE2C,UBE2I,UCKL1,USP24,USP47,USP8,USP9X,VCP,VCPIP1,VPS11,VPS13A,VPS36,WDR12,WDR45B,WDR62,WFS1,WIPF1,XRN1,ZMPSTE24	281
Cancer,Organismal Injury and Abnormalities	Malignant solid organ tumor	0,00000669		-0,927	AARSD1,ABCD4,ACO1,ACO2,ACP2,ACSF2,ACSL3,ACSL4,ADD1,ADI1,AGA,AGFG1,AGL,AKR1B1,AKR1B10,ALDH2,ALDH4A1,ALDH6A1,ALDH9A1,ANAPC7,ANKRD13A,ANKRD46,ANXA4,APPL2,ARFGAP2,ARFGAP3,ARFGEF2,ARHGEF1,ARHGEF12,ARHGEF18,ARPIN/ARPIN-AP3S2,ASRGL1,ATIC,ATP2A2,ATP2B4,ATP6AP1,ATP6V1A,ATPAF2,ATXN10,BCAS1,BCAT2,BIRC6,CAMSAP2,CAND1,CANX,CARMIL1,CAVIN1,CDC37L1,CDK1,CDK4,CEP55,CNEP1R1,CNN2,CNPY2,CRAT,CSNK1A1,CSRP1,CTBP2,CTTNBP2,CUL3,CYB5R1,DDB1,DDRGK1,DDX5,DGKZ,DGLUCY,DNAJC5,DNMT1,DNPEP,DOCK1,DOCK5,DPY19L1,DPYSL2,DPYSL3,DYNC2H1,ECI2,EEF1B2,EEF2,EEF2K,EIF2B4,ELOA,ELP3,EML1,ERLIN2,EXOC2,EXTL3,FADD,FAF2,FASN,FBXL15,FLII,FLNA,FLNB,FRMD4A,FUT8,GATM,GCDH,GCLC,GDA,GMDS,GMPPB,GOT2,GPT2,GRB14,GTSE1,HAUS7,HDGFL3,HMGCL,HMGCS1,HMOX1,HMOX2,HNRNPUL1,IDH2,IKBIP,INPP1,INPPL1,IRGQ,ITPR1,ITPR3,ITPRID2,KANK1,KANK2,KATNB1,KPNA2,LARP7,LAYN,LETM1,LIMD1,LPP,LRRC40,LTA4H,MARK2,MAT2A,MEGF10,MKLN1,MMUT,MON2,MPI,MPST,MTSS1,NAA25,NAB2,NAPA,NAXE,NCLN,NEK9,NFKB2,NFS1,NPTXR,NQO1,NRDC,OAT,OSBPL8,PAK1,PAPSS1,PCBP2,PDE4DIP,PEPD,PGD,PHLDA1,PHYH,PIGU,PITRM1,PLCB1,POLA1,POLD1,POLD3,POLDIP3,PPOX,PPP4R3A,PRKACB,PRKCA,PRPF19,PRRC2A,PRRX1,QSOX2,RAB32,RAD51,RAF1,RAI14,RANGAP1,RASSF4,RBPJ,RIOK3,RPA1,RPL13,RPL3,RPL5,RPN1,RPN2,RRM1,RRM2,SAMD4B,SEC14L1,SEC24A,SEC24D,SEC61A1,SERPINE2,SFR1,SGTA,SLC1A4,SOAT1,SPP1,SQLE,SRGAP3,STARD3NL,STAT1,STAT3,STAT5B,STRN4,STT3A,SUCLG2,TAX1BP1,TCF12,TDRKH,TKFC,TLE3,TLN1,TM9SF4,TMEM43,TNS3,TOM1L2,TRIM2,TRIM28,TRIM3,TRIM47,TRIOBP,TRIP12,TRIP13,TRMT2A,TST,TTC21B,UBAP2L,UBE2C,UBE2O,UBR7,USP24,USP47,USP8,USP9X,VAMP7,VAT1,VCP,VPS11,VPS13A,VPS4B,VWA5A,WDR12,WDR45B,WDR62,WFS1,XRN1	252

Categories	Diseases or Functions Annotation	p-value	Predicted Activation State	Activation z-score	Molecules	# Molecules
Cellular Function and Maintenance	Cellular homeostasis	0,000016		-0,904	ACO1,ACP2,AGA,AGFG1,AKR1B1,APPL2,ATP2A2,ATP2B4,ATP6AP1,ATP6V1A,BCAT2,BIRC6,CAND1,CCDC47,CDK6,CUL3,DDRGK1,DGKZ,DNMT1,EEF2,EEF2K,ELP1,EXOC2,EXOC4,FADD,FBXO7,GABPA,GATM,GCDH,HMOX1,HMOX2,ITPR1,ITPR3,ITPRID2,LAMTOR2,LAMTOR3,LETM1,MARK2,MEGF10,MTDH,MTSS1,NLE1,NMD3,NQO1,PAK1,PAPSS1,PFKM,PPA2,PRDX2,PRKCA,RAF1,RAP2C,RBPJ,RPL7A,SH3GLB1,SLC9A3R1,SNF8,SOAT1,SPP1,SQSTM1,STAM2,STAT1,STAT3,STAT5B,TCF12,TPD52,UBAP2L,UBE2O,VAMP7,VCP,VPS11,VPS13A,VPS36,WFS1,ZMPSTE24	75
Cancer,Organismal Injury and Abnormalities	Formation of solid tumor	1,75E-14		-0,88	AARSD1,ABCD4,ACADS,ACO1,ACO2,ACP2,ACSF2,ACSL3,ACSL4,ADD1,ADI1,AGA,AGFG1,AGL,AIP,AK3,AKR1B1,AKR1B10,AKR7A2,ALDH2,ALDH4A1,ALDH6A1,ALDH9A1,ANAPC7,ANKRD13A,ANXA4,AP3S2,APPL2,ARFGAP2,ARFGAP3,ARFGEF2,ARHGEF1,ARHGEF12,ARHGEF18,ARMC8,ARPIN/ARPIN-AP3S2,ASRGL1,ATIC,ATP2A2,ATP2B4,ATP6AP1,ATP6V1A,ATPAF2,ATXN10,BCAS1,BCAT2,BIRC6,C1QBP,CACYBP,CAMSAP2,CAND1,CANX,CARMIL1,CAVIN1,CBX3,CC2D1B,CCDC47,CCZ1/CCZ1B,CDC42EP4,CDIPT,CDK1,CDK4,CDK6,CEP55,CERT1,CNN2,CNN3,CNPY2,CRAT,CSNK1A1,CSRP1,CTBP2,CTTNBP2,CUL3,CWF19L1,CYB5R1,CYFIP1,DCTN6,DDB1,DDRGK1,DDX5,DGKZ,DGLUCY,DNAJC5,DNMT1,DNPEP,DOCK1,DOCK5,DPY19L1,DPYSL2,DPYSL3,DTYMK,DYNC2H1,EEF1B2,EEF2,EEF2K,EIF2B4,EIF2B5,ELOA,ELP1,ELP2,ELP3,EMC7,EMD,EML1,ERLIN2,EXOC2,EXOC4,EXTL3,FADD,FAF2,FASN,FBXL15,FBXO7,FDFT1,FLII,FLNA,FLNB,FRMD4A,FUT8,GABPA,GATM,GCDH,GCLC,GCLM,GDA,GFPT1,GHDC,GLRX3,GLRX5,GMDS,GMPPB,GOT2,GPT2,GRB14,GSDMD,GSTZ1,GTF2B,GTSE1,HAUS7,HDGFL3,HEBP2,HM13,HMGCS1,HMMR,HMOX1,HNRNPUL1,HSPA4L,IDH2,IFT74,IKBIP,IMPA1,INPPL1,IRGQ,ISCA2,ITPR1,ITPR3,ITPRID2,KANK1,KANK2,KATNB1,KPNA2,LARP7,LAYN,LETM1,LIMD1,LOXL3,LPP,LRRC40,LTA4H,MARK2,MEGF10,MKLN1,MMUT,MON2,MPI,MTDH,MTSS1,NAA25,NAB2,NAPA,NCLN,NDC80,NEK9,NFKB2,NIT2,NLE1,NLN,NMD3,NPTXR,NQO1,NRBP1,NRDC,NT5C3B,NUP54,OAT,OGT,OSBPL8,OXCT1,PAFAH1B1,PAK1,PAPSS1,PARG,PCBP2,PCK2,PDE4DIP,PDZRN3,PEPD,PFKM,PHLDA1,PHYH,PIGU,PIP4P2,PITRM1,PKN2,PLCB1,POLA1,POLD1,POLD3,POLDIP3,PPAT,PPOX,PPP2R5D,PPP4R3A,PRDX2,PRKACB,PRKCA,PRPF19,PRRC2A,PRRX1,PTPN1,QSOX2,RAB32,RABGGTA,RAD51,RAF1,RAI14,RANGAP1,RASSF4,RBPJ,RCN2,RER1,RETSAT,RIOK3,RNASEH2B,RPA1,RPL27,RPL5,RPN1,RPN2,RRM1,RRM2,SAMD4B,SDF4,SEC14L1,SEC24A,SEC24D,SEC61A1,SERPINE2,SH3GLB1,SLC1A4,SLC9A3R1,SLC9A3R2,SNF8,SNX1,SNX17,SOAT1,SORBS3,SPCS3,SPP1,SQLE,SQSTM1,SRGAP3,STARD3NL,STAT1,STAT3,STAT5B,STRN4,STT3A,SUCLG2,TAX1BP1,TCF12,TDRKH,TKFC,TLE3,TLN1,TM9SF3,TM9SF4,TMEM115,TMEM43,TNS3,TOM1L2,TPD52,TRIM2,TRIM28,TRIM3,TRIM47,TRIOBP,TRIP10,TRIP12,TRIP13,TRMT2A,TST,UBAP2L,UBE2C,UBE2I,UBE2O,UBR7,UCKL1,USP24,USP47,USP8,USP9X,VCP,VCPIP1,VPS11,VPS13A,VPS36,VWA5A,WDR12,WDR45B,WDR62,WFS1,WIPF1,XRN1,ZMPSTE24	326
Cell Death and Survival	Cell death of blood cells	0,000288		-0,861	AGA,AIP,ARHGEF1,BIRC6,CDK1,CDK6,CSNK1A1,CUL3,FADD,GSDMD,HMOX1,ITPR1,ITPR3,LAMTOR2,NFKB2,NQO1,OGT,PCBP2,PRKCA,RAF1,RBPJ,SH3GLB1,SOAT1,SPP1,STAM2,STAT1,STAT3,STAT5B,TLN1,TRIM28,USP9X,WIPF1,ZMPSTE24	33
Neurological Disease	Movement Disorders	0,0000024		-0,846	AGA,AGL,ALDH2,ALDH6A1,ARHGEF1,ATP2A2,ATP6V1A,ATXN10,BCAS1,CANX,CWF19L1,DGKZ,DNAJC5,DNMT1,DPYSL3,EEF2,EML1,ERLIN2,FBXO7,FDFT1,FRMD4A,HMOX1,HMOX2,IMPA1,INPP1,ITPR1,KANK1,LOXL3,LPP,MTDH,NAB2,NAPA,NARS1,NDUFAF7,NQO1,OGT,OSBPL8,PAK1,PDE4DIP,PFKM,PHYH,PIP4P2,PITRM1,PLCB1,PRDX2,RAB6A,RAD51,RPL13,RPL3,SEC24A,SGTA,SLC1A4,SPP1,SQSTM1,SRGAP3,STAT1,STRN4,TPD52,USP24,VPS13A,ZMPSTE24	61
Cancer,Organismal Injury and Abnormalities	Frequency of tumor	2,99E-10		-0,823	AARSD1,ABCD4,ACADS,ACO1,ACO2,ACSF2,ACSL3,ACSL4,ADD1,AGL,AIP,AK3,AKR1B1,AKR1B10,AKR7A2,ALDH2,ALDH4A1,ALDH6A1,ANKRD13A,ANXA4,AP3S2,APPL2,ARFGAP2,ARFGAP3,ARFGEF2,ARHGEF1,ARHGEF12,ARHGEF18,ARMC8,ASRGL1,ATIC,ATP2A2,ATP6AP1,ATXN10,BCAT2,BIRC6,C1QBP,CACYBP,CAMSAP2,CAND1,CANX,CAVIN1,CBX3,CC2D1B,CCDC47,CDIPT,CDK1,CDK4,CDK6,CEP55,CERT1,CNN2,CNPY2,CRAT,CSNK1A1,CTBP2,CTTNBP2,CUL3,CYB5R1,CYFIP1,DCTN6,DDB1,DDRGK1,DDX5,DGKZ,DGLUCY,DNAJC5,DNMT1,DOCK1,DOCK5,DPY19L1,DPYSL2,DPYSL3,DTYMK,DYNC2H1,EEF1B2,EEF2,EEF2K,EIF2B4,ELOA,ELP1,ELP2,ELP3,EMC7,EMD,EML1,ERLIN2,EXOC2,EXOC4,EXTL3,FAF2,FASN,FBXO7,FDFT1,FLII,FLNA,FLNB,FRMD4A,FUT8,GATM,GCDH,GCLM,GFPT1,GHDC,GLRX3,GLRX5,GMDS,GMPPB,GOT2,GPT2,GRB14,GSTZ1,GTF2B,GTSE1,HAUS7,HDGFL3,HEBP2,HM13,HMMR,HMOX1,HNRNPUL1,HSPA4L,IDH2,IFT74,IKBIP,IMPA1,INPPL1,IRGQ,ISCA2,ITPR1,ITPR3,ITPRID2,KANK1,KPNA2,LARP7,LAYN,LETM1,LIMD1,LOXL3,LPP,LRRC40,LTA4H,MARK2,MEGF10,MKLN1,MMUT,MON2,MTDH,MTSS1,NAA25,NAB2,NAPA,NCLN,NDC80,NEK9,NFKB2,NLE1,NMD3,NPTXR,NQO1,NRBP1,NRDC,NT5C3B,NUP54,OAT,OGT,OSBPL8,OXCT1,PAFAH1B1,PAK1,PAPSS1,PARG,PCBP2,PCK2,PDE4DIP,PDZRN3,PEPD,PFKM,PHLDA1,PHYH,PIGU,PITRM1,PKN2,PLCB1,POLA1,POLD1,POLD3,POLDIP3,PPAT,PPOX,PPP2R5D,PPP4R3A,PRDX2,PRKACB,PRKCA,PRPF19,PRRC2A,PRRX1,PTPN1,QSOX2,RAB32,RAD51,RAF1,RAI14,RANGAP1,RBPJ,RCN2,RER1,RETSAT,RIOK3,RNASEH2B,RPA1,RPL5,RPN1,RPN2,RRM1,RRM2,SAMD4B,SDF4,SEC14L1,SEC24A,SEC24D,SEC61A1,SERPINE2,SLC1A4,SLC9A3R1,SLC9A3R2,SNF8,SNX1,SNX17,SOAT1,SORBS3,SPP1,SQLE,SQSTM1,SRGAP3,STAT1,STAT3,STAT5B,STT3A,SUCLG2,TAX1BP1,TCF12,TDRKH,TKFC,TLN1,TM9SF3,TM9SF4,TMEM115,TMEM43,TNS3,TOM1L2,TPD52,TRIM2,TRIM28,TRIM3,TRIM47,TRIOBP,TRIP10,TRIP12,TRIP13,TRMT2A,UBAP2L,UBE2C,UBE2I,UCKL1,USP24,USP47,USP8,USP9X,VCP,VCPIP1,VPS11,VPS13A,VPS36,WDR12,WDR45B,WDR62,WFS1,WIPF1,XRN1,ZMPSTE24	282
Molecular Transport,Protein Trafficking	Transport of protein	0,00000686		-0,816	AIP,AP3S2,APPL2,ARFGEF2,CDK1,DDX5,EXOC4,FBXO7,FLNA,HIKESHI,KPNA2,NAPA,NUP54,PITRM1,RAB6A,RANGAP1,SNX1,SQSTM1,STAT3,TRIM28,VAMP7,VPS11	22
Cancer,Organismal Injury and Abnormalities	Adenocarcinoma	6,67E-13		-0,805	AARSD1,ABCD4,ACAA2,ACADS,ACO1,ACO2,ACP2,ACSF2,ACSL3,ACSL4,ADD1,AGA,AGFG1,AGL,AIP,AK3,AKR1B1,AKR1B10,AKR7A2,ALDH2,ALDH4A1,ALDH6A1,ALDH9A1,ANKRD13A,ANXA4,AP3S2,APPL2,ARFGAP2,ARFGAP3,ARFGEF2,ARHGEF1,ARHGEF12,ARHGEF18,ARMC8,ASRGL1,ATIC,ATP2A2,ATP2B4,ATP6AP1,ATXN10,BCAT2,BIRC6,C1QBP,CACYBP,CAMSAP2,CAND1,CANX,CARMIL1,CAVIN1,CBX3,CC2D1B,CCDC47,CDC37L1,CDC42EP4,CDIPT,CDK1,CDK4,CDK6,CEP55,CERT1,CNPY2,CRAT,CSNK1A1,CTBP2,CTTNBP2,CUL3,CWF19L1,CYB5R1,CYFIP1,DCTN6,DDB1,DDRGK1,DDX5,DGKZ,DGLUCY,DNAJC5,DNMT1,DNPEP,DOCK1,DOCK5,DPY19L1,DPYSL2,DPYSL3,DTYMK,DYNC2H1,EEF1B2,EEF2,EEF2K,EIF2B5,ELOA,ELP1,ELP2,ELP3,EMC7,EMD,EML1,ERLIN2,EXOC2,EXOC4,EXTL3,FADD,FADS1,FAF2,FASN,FBXO7,FDFT1,FLII,FLNA,FLNB,FRMD4A,FUT8,GATM,GCDH,GCLC,GCLM,GDA,GFPT1,GHDC,GLRX3,GLRX5,GMDS,GMPPB,GOT2,GPT2,GRB14,GSDMD,GSTZ1,GTF2B,GTSE1,HACD3,HAUS7,HDGFL3,HEBP2,HM13,HMGCL,HMGCS1,HMMR,HMOX1,HMOX2,HNRNPUL1,HSPA4L,IDH2,IFT74,IKBIP,IMPA1,INPPL1,IRGQ,ISCA2,ITPR1,ITPR3,ITPRID2,KANK1,KANK2,KPNA2,LARP7,LAYN,LETM1,LIMD1,LOXL3,LPCAT3,LPP,LRRC40,LRRC59,LTA4H,MARK2,MEGF10,MKLN1,MMUT,MON2,MPI,MPST,MTDH,MTSS1,NAA25,NAB2,NAPA,NCLN,NDC80,NDUFAF7,NEK9,NFKB2,NLE1,NLN,NMD3,NPTXR,NQO1,NRBP1,NRDC,NT5C3B,NUP54,OAT,OGT,OSBPL8,OXCT1,PAFAH1B1,PAK1,PAPSS1,PARG,PCBP2,PCK2,PDE4DIP,PDZRN3,PEPD,PFKM,PHLDA1,PHYH,PIGU,PIP4P2,PITRM1,PKN2,PLCB1,PMPCB,POLA1,POLD1,POLD3,POLDIP3,PPA2,PPAT,PPOX,PPP2R5D,PPP4R3A,PRDX2,PRKACB,PRKCA,PRPF19,PRRC2A,PRRX1,PTPN1,QSOX2,RAB32,RABGGTA,RAD51,RAF1,RAI14,RANGAP1,RAP2C,RASSF4,RBPJ,RCN2,RER1,RETSAT,RIOK3,RNASEH2B,RPA1,RPA2,RPL27,RPL3,RPL5,RPN1,RRM1,RRM2,SAMD4B,SDF4,SEC14L1,SEC24A,SEC24D,SEC61A1,SERPINE2,SGTA,SH3GLB1,SLC1A4,SLC9A3R1,SLC9A3R2,SNF8,SNX1,SNX17,SOAT1,SORBS3,SPP1,SQLE,SQSTM1,SRGAP3,STAT1,STAT3,STAT5B,STRN4,STT3A,SUCLG2,TAX1BP1,TCF12,TDRKH,TKFC,TLE3,TLN1,TM9SF3,TM9SF4,TMEM115,TMEM43,TNS3,TOM1L2,TPD52,TRAPPC4,TRIM2,TRIM28,TRIM3,TRIM47,TRIOBP,TRIP10,TRIP12,TRIP13,TRMT2A,TST,TTC21B,UBAP2L,UBE2C,UBE2O,UCKL1,USP24,USP47,USP8,USP9X,VAT1,VCP,VCPIP1,VPS11,VPS13A,VPS36,VPS4B,VWA5A,WDR12,WDR45B,WDR62,WFS1,WIPF1,XRN1,ZMPSTE24	326
Lipid Metabolism,Molecular Transport,Small Molecule Biochemistry	Transport of lipid	0,0000132		-0,758	ACSL3,ACSL4,ARHGEF12,CANX,CERT1,CRAT,CUL3,FADD,GOT2,LPCAT3,NAXE,OSBPL8,SEC24A,SLC9A3R1,SLC9A3R2,SOAT1,VAMP7,VPS4B	18
Cancer,Organismal Injury and Abnormalities	Breast or pancreatic cancer	0,0000103		-0,747	ABCD4,ACACA,ACADS,ACO1,ACP2,ACSF2,AGL,AK3,AKR1B1,AKR7A2,APPL2,ARFGAP3,ARFGEF2,ARHGEF1,ARHGEF12,ARHGEF18,ASRGL1,ATP2B4,ATXN10,BIRC6,C17orf49,C1QBP,CAND1,CANX,CARMIL1,CAVIN1,CBX3,CC2D1B,CCDC47,CDK1,CDK4,CDK6,CERT1,CNN2,CNPY2,CRAT,CSNK1A1,CTBP2,CTTNBP2,Cyb5r3,DDB1,DDX5,DGKZ,DGLUCY,DNMT1,DOCK5,DPY19L1,DPYSL2,DYNC2H1,EEF1B2,ELOA,ELP1,ELP2,ELP3,EMC7,EMD,EML1,ERLIN2,EXOC4,EXTL3,FADD,FASN,FBXO7,FDFT1,FLII,FLNA,FLNB,GABPA,GCDH,GCLC,GMDS,GMPPB,GOT2,GTSE1,HACD3,HDGFL3,HMGCS1,HMMR,HMOX1,HNRNPUL1,INPPL1,ITPR1,ITPRID2,KPNA2,LETM1,LIMD1,LOXL3,LPP,LTA4H,MARK2,MGST2,MKLN1,MMUT,MTDH,NAA25,NAB2,NCLN,NDC80,NEK9,NFKB2,NPTXR,NQO1,OSBPL8,OXCT1,PAK1,PCK2,PDE4DIP,PEPD,PFKM,PHLDA1,PITRM1,PKN2,PLCB1,POLD1,POLD3,POLDIP3,PPOX,PPP2R5D,PRKACB,PRKCA,PRRX1,PTPN1,RABGGTA,RAD51,RAF1,RANGAP1,RIOK3,RNASEH2B,RPA1,RPL5,RPL6,RPN1,RRM1,RRM2,SAMD4B,SEC14L1,SEC24A,SEC61A1,SERPINE2,SLC9A3R1,SNX17,SPP1,SQLE,SRGAP3,STAT1,STAT3,STAT5B,SUCLG2,TAGLN2,TCF12,TKFC,TLE3,TLN1,TM9SF4,TMEM43,TNS3,TPD52,TRIM28,TRIM3,TRIP10,TRIP12,TRIP13,TRMT2A,UBAP2L,UBE2C,USP24,USP47,USP9X,VCPIP1,VPS13A,VWA5A,WFS1,WIPF1,ZMPSTE24	174
Cell Morphology,Cellular Function and Maintenance	Autophagy	4,8E-08		-0,726	ACP2,AGFG1,BIRC6,CUL3,DDRGK1,EEF2,EEF2K,EXOC2,EXOC4,FADD,FBXO7,HMOX1,ITPR1,LAMTOR2,LAMTOR3,LETM1,MEGF10,MTDH,MTSS1,NMD3,NQO1,PAPSS1,RAF1,RPL7A,SH3GLB1,SNF8,SOAT1,SPP1,SQSTM1,STAM2,STAT1,STAT3,UBE2O,VAMP7,VCP,VPS11,VPS13A,VPS36,ZMPSTE24	39
Cancer,Organismal Injury and Abnormalities	Development of malignant tumor	1,89E-09		-0,717	AARSD1,ABCD4,ACADS,ACO1,ACO2,ACSF2,ACSL3,ACSL4,ADD1,AGL,AK3,AKR1B1,AKR1B10,AKR7A2,ALDH2,ALDH4A1,ALDH6A1,ANKRD13A,ANXA4,AP3S2,APPL2,ARFGAP2,ARFGAP3,ARFGEF2,ARHGEF1,ARHGEF12,ARHGEF18,ARMC8,ASRGL1,ATIC,ATP2A2,ATP6AP1,ATXN10,BCAT2,BIRC6,C1QBP,CACYBP,CAMSAP2,CAND1,CANX,CAVIN1,CBX3,CC2D1B,CCDC47,CDIPT,CDK1,CDK4,CDK6,CEP55,CERT1,CNN2,CNPY2,CRAT,CSNK1A1,CTBP2,CTTNBP2,CUL3,CYB5R1,CYFIP1,DCTN6,DDB1,DDRGK1,DDX5,DGKZ,DGLUCY,DNAJC5,DNMT1,DOCK1,DOCK5,DPY19L1,DPYSL2,DPYSL3,DTYMK,DYNC2H1,EEF1B2,EEF2,EEF2K,EIF2B4,ELOA,ELP1,ELP2,ELP3,EMC7,EMD,EML1,ERLIN2,EXOC2,EXOC4,EXTL3,FAF2,FASN,FBXO7,FDFT1,FLII,FLNA,FLNB,FRMD4A,FUT8,GATM,GCDH,GCLM,GFPT1,GHDC,GLRX3,GLRX5,GMDS,GMPPB,GOT2,GPT2,GRB14,GSTZ1,GTF2B,GTSE1,HAUS7,HDGFL3,HEBP2,HM13,HMMR,HNRNPUL1,HSPA4L,IDH2,IFT74,IKBIP,IMPA1,INPPL1,IRGQ,ISCA2,ITPR1,ITPR3,ITPRID2,KANK1,KPNA2,LARP7,LAYN,LETM1,LIMD1,LOXL3,LPP,LRRC40,LTA4H,MARK2,MEGF10,MKLN1,MMUT,MON2,MTDH,MTSS1,NAA25,NAB2,NAPA,NCLN,NDC80,NEK9,NFKB2,NLE1,NMD3,NPTXR,NQO1,NRBP1,NRDC,NT5C3B,NUP54,OAT,OGT,OSBPL8,OXCT1,PAFAH1B1,PAK1,PAPSS1,PARG,PCBP2,PCK2,PDE4DIP,PDZRN3,PEPD,PFKM,PHLDA1,PHYH,PIGU,PITRM1,PKN2,PLCB1,POLA1,POLD1,POLD3,POLDIP3,PPAT,PPOX,PPP2R5D,PPP4R3A,PRDX2,PRKACB,PRKCA,PRPF19,PRRC2A,PRRX1,PTPN1,QSOX2,RAB32,RAD51,RAF1,RAI14,RANGAP1,RBPJ,RCN2,RER1,RETSAT,RIOK3,RNASEH2B,RPA1,RPL5,RRM1,RRM2,SAMD4B,SDF4,SEC14L1,SEC24A,SEC24D,SEC61A1,SERPINE2,SLC1A4,SLC9A3R1,SLC9A3R2,SNF8,SNX1,SNX17,SOAT1,SORBS3,SPP1,SQLE,SQSTM1,SRGAP3,STAT1,STAT3,STAT5B,STT3A,SUCLG2,TAX1BP1,TCF12,TDRKH,TKFC,TLN1,TM9SF3,TM9SF4,TMEM115,TMEM43,TNS3,TOM1L2,TPD52,TRIM2,TRIM28,TRIM3,TRIM47,TRIOBP,TRIP10,TRIP12,TRIP13,TRMT2A,UBAP2L,UBE2C,UCKL1,USP24,USP47,USP8,USP9X,VCP,VCPIP1,VPS11,VPS13A,VPS36,WDR12,WDR45B,WDR62,WFS1,WIPF1,XRN1,ZMPSTE24	277
Cancer,Organismal Injury and Abnormalities	Development of carcinoma	1,03E-09		-0,706	AARSD1,ABCD4,ACADS,ACO1,ACO2,ACSF2,ACSL3,ACSL4,ADD1,AGL,AK3,AKR1B1,AKR1B10,AKR7A2,ALDH2,ALDH4A1,ALDH6A1,ANKRD13A,ANXA4,AP3S2,APPL2,ARFGAP2,ARFGAP3,ARFGEF2,ARHGEF1,ARHGEF12,ARHGEF18,ARMC8,ASRGL1,ATIC,ATP2A2,ATP6AP1,ATXN10,BCAT2,BIRC6,C1QBP,CACYBP,CAMSAP2,CAND1,CANX,CAVIN1,CBX3,CC2D1B,CCDC47,CDIPT,CDK1,CDK4,CDK6,CEP55,CERT1,CNPY2,CRAT,CSNK1A1,CTBP2,CTTNBP2,CUL3,CYB5R1,CYFIP1,DCTN6,DDB1,DDRGK1,DDX5,DGKZ,DGLUCY,DNAJC5,DNMT1,DOCK1,DOCK5,DPY19L1,DPYSL2,DPYSL3,DTYMK,DYNC2H1,EEF1B2,EEF2,EEF2K,EIF2B4,ELOA,ELP1,ELP2,ELP3,EMC7,EMD,EML1,ERLIN2,EXOC2,EXOC4,EXTL3,FAF2,FASN,FBXO7,FDFT1,FLII,FLNA,FLNB,FRMD4A,FUT8,GATM,GCDH,GCLM,GFPT1,GHDC,GLRX3,GLRX5,GMDS,GMPPB,GOT2,GPT2,GRB14,GSTZ1,GTF2B,GTSE1,HAUS7,HDGFL3,HEBP2,HM13,HMMR,HNRNPUL1,HSPA4L,IDH2,IFT74,IKBIP,IMPA1,INPPL1,IRGQ,ISCA2,ITPR1,ITPR3,ITPRID2,KANK1,KPNA2,LARP7,LAYN,LETM1,LIMD1,LOXL3,LPP,LRRC40,LTA4H,MARK2,MEGF10,MKLN1,MMUT,MON2,MTDH,MTSS1,NAA25,NAB2,NAPA,NCLN,NDC80,NEK9,NFKB2,NLE1,NMD3,NPTXR,NQO1,NRBP1,NRDC,NT5C3B,NUP54,OAT,OGT,OSBPL8,OXCT1,PAFAH1B1,PAK1,PAPSS1,PARG,PCBP2,PCK2,PDE4DIP,PDZRN3,PEPD,PFKM,PHLDA1,PHYH,PIGU,PITRM1,PKN2,PLCB1,POLA1,POLD1,POLD3,POLDIP3,PPAT,PPOX,PPP2R5D,PPP4R3A,PRDX2,PRKACB,PRKCA,PRPF19,PRRC2A,PRRX1,PTPN1,QSOX2,RAB32,RAD51,RAF1,RAI14,RANGAP1,RBPJ,RCN2,RER1,RETSAT,RIOK3,RNASEH2B,RPA1,RPL5,RRM1,RRM2,SAMD4B,SDF4,SEC14L1,SEC24A,SEC24D,SEC61A1,SERPINE2,SLC1A4,SLC9A3R1,SLC9A3R2,SNF8,SNX1,SNX17,SOAT1,SORBS3,SPP1,SQLE,SQSTM1,SRGAP3,STAT1,STAT3,STAT5B,STT3A,SUCLG2,TAX1BP1,TCF12,TDRKH,TKFC,TLN1,TM9SF3,TM9SF4,TMEM115,TMEM43,TNS3,TOM1L2,TPD52,TRIM2,TRIM28,TRIM3,TRIM47,TRIOBP,TRIP10,TRIP12,TRIP13,TRMT2A,UBAP2L,UBE2C,UCKL1,USP24,USP47,USP8,USP9X,VCP,VCPIP1,VPS11,VPS13A,VPS36,WDR12,WDR45B,WDR62,WFS1,WIPF1,XRN1,ZMPSTE24	276
Cellular Development,Cellular Growth and Proliferation	Proliferation of pancreatic cancer cell lines	0,000385		-0,673	EXTL3,OGT,PAK1,PRKACB,PRKCA,RRM1,RRM2,STAT3,STAT5B,TRIM2,UBE2C,USP9X,VCP	13
Cellular Assembly and Organization,Cellular Function and Maintenance	Quantity of cellular protrusions	0,00000897		-0,673	ADI1,CANX,DOCK1,DOCK5,GDA,HMMR,INPPL1,KATNB1,MTSS1,PAK1,SLC9A3R1,SLC9A3R2,STAT3,TLN1,TRIOBP	15

Categories	Diseases or Functions Annotation	p-value	Predicted Activation State	Activation z-score	Molecules	# Molecules
Cancer,Organismal Injury and Abnormalities	Non-hematological solid tumor	1,93E-20		-0,657	AARSD1,ABCD4,ACAA2,ACADS,ACO1,ACO2,ACP2,ACSF2,ACSL3,ACSL4,ADD1,ADI1,ADO,AGA,AGFG1,AGL,AIP,AK3,AKR1B1,AKR1B10,AKR7A2,ALDH2,ALDH4A1,ALDH6A1,ALDH9A1,ANAPC7,ANKRD13A,ANKRD46,ANXA4,AP3S2,APPL2,ARFGAP2,ARFGAP3,ARFGEF2,ARHGEF1,ARHGEF12,ARHGEF18,ARMC8,ARPIN/ARPIN-AP3S2,ASRGL1,ATIC,ATP2A2,ATP2B4,ATP6AP1,ATP6V1A,ATPAF2,ATXN10,BCAS1,BCAT2,BIRC6,C17orf49,C1QBP,CACYBP,CAMSAP2,CAND1,CANX,CARMIL1,CAVIN1,CBX3,CC2D1B,CCDC47,CDC37L1,CDC42EP4,CDIPT,CDK1,CDK4,CDK6,CEP55,CERT1,CNEP1R1,CNN2,CNN3,CNPY2,CRAT,CSNK1A1,CSRP1,CTBP2,CTTNBP2,CUL3,CWF19L1,CYB5R1,CYFIP1,DCTN6,DDB1,DDRGK1,DDX5,DGKZ,DGLUCY,DNAJC5,DNAJC9,DNMT1,DNPEP,DOCK1,DOCK5,DPY19L1,DPYSL2,DPYSL3,DTYMK,DYNC2H1,ECI2,EEF1B2,EEF2,EEF2K,EIF2B4,EIF2B5,ELOA,ELP1,ELP2,ELP3,EMC7,EMD,EML1,ERLIN2,EXOC2,EXOC4,EXTL3,FADD,FADS1,FAF2,FASN,FBXL15,FBXO7,FDFT1,FLII,FLNA,FLNB,FRMD4A,FUT8,GABPA,GATM,GCDH,GCLC,GCLM,GDA,GFPT1,GHDC,GLRX,GLRX3,GLRX5,GMDS,GMPPB,GOT2,GPT2,GRB14,GSDMD,GSTZ1,GTF2B,GTSE1,HACD3,HAUS7,HDGFL3,HEBP2,HIKESHI,HM13,HMGCL,HMGCS1,HMMR,HMOX1,HMOX2,HNRNPUL1,HSPA4L,IDH2,IFT74,IKBIP,IMPA1,INPP1,INPPL1,IRGQ,ISCA2,ITPR1,ITPR3,ITPRID2,KANK1,KANK2,KATNB1,KPNA2,LAMTOR2,LAMTOR3,LARP7,LAYN,LETM1,LIMD1,LOXL3,LPCAT3,LPP,LRRC40,LRRC59,LTA4H,MARK2,MAT2A,MEGF10,MGST2,MKLN1,MMUT,MON2,MPI,MPST,MTDH,MTSS1,NAA25,NAB2,NAPA,NAXE,NCLN,NDC80,NDUFAF7,NEK9,NFKB2,NFS1,NIT2,NLE1,NLN,NMD3,NPTXR,NQO1,NRBP1,NRDC,NT5C3B,NUP54,OAT,OGT,OSBPL8,OXCT1,PAFAH1B1,PAK1,PAPSS1,PARG,PCBP2,PCK2,PDE4DIP,PDZRN3,PEPD,PFKM,PGD,PHLDA1,PHYH,PIGU,PIP4P2,PITRM1,PKN2,PLCB1,PMPCB,POLA1,POLD1,POLD3,POLDIP3,PPA2,PPAT,PPOX,PPP2R5D,PPP4R3A,PRDX2,PRKACB,PRKCA,PRPF19,PRRC2A,PRRX1,PTPN1,QSOX2,RAB32,RAB6A,RABGGTA,RAD51,RAF1,RAI14,RANGAP1,RAP2C,RASSF4,RBPJ,RCN2,RER1,RETSAT,RIOK3,RNASEH2B,RPA1,RPA2,RPL13,RPL27,RPL3,RPL5,RPL6,RPL7A,RPN1,RPN2,RRM1,RRM2,SAMD4B,SDF4,SEC14L1,SEC24A,SEC24D,SEC61A1,SERPINE2,SFR1,SGTA,SH3GLB1,SLC1A4,SLC9A3R1,SLC9A3R2,SNF8,SNX1,SNX17,SOAT1,SORBS3,SPCS3,SPP1,SQLE,SQSTM1,SRGAP3,SRXN1,STAM2,STARD3NL,STAT1,STAT3,STAT5B,STRN4,STT3A,SUCLG2,TAGLN2,TAX1BP1,TCF12,TDRKH,TKFC,TLE3,TLN1,TM9SF3,TM9SF4,TMEM115,TMEM43,TNS3,TOM1L2,TPD52,TRAPPC4,TRIM2,TRIM28,TRIM3,TRIM47,TRIOBP,TRIP10,TRIP12,TRIP13,TRMT2A,TST,TTC21B,UBAP2L,UBE2C,UBE2I,UBE2O,UBR7,UCKL1,USP24,USP47,USP8,USP9X,VAMP7,VAT1,VCP,VCPIP1,VPS11,VPS13A,VPS36,VPS4B,VWA5A,WDR12,WDR45B,WDR62,WFS1,WIPF1,XRN1,ZMPSTE24	370
Cellular Assembly and Organization,Cellular Function and Maintenance,Tissue Development	Formation of actin stress fibers	0,000233		-0,622	ARHGEF1,ARHGEF12,ARHGEF18,CARMIL1,CDK4,CDK6,CUL3,Cyb5r3,KANK2,MARK2,PAK1,PKN2,SORBS3,STAT3,TNS3,TRIP10	16
Cell Death and Survival	Apoptosis of pancreatic cancer cell lines	0,000164		-0,59	ACACA,EXOC2,FADD,GLRX,HMMR,OGT,RRM1,SPP1,STAT1,STAT3,VCP	11
Cellular Development,Cellular Growth and Proliferation	Cell proliferation of colorectal cancer cell lines	0,000191		-0,574	BIRC6,CACYBP,CDK1,DDB1,DDX5,EIF2B5,EXOC2,FASN,NFKB2,PAK1,PGD,PHLDA1,PTPN1,RAD51,RAF1,RRM2,SNX1,STAT3,TCF12,UBE2C,UBE2I	21
Cellular Assembly and Organization	Quantity of actin filaments	0,0000124		-0,555	FLNA,FLNB,PAFAH1B1,PAK1,RAF1,RIOK3,SLC9A3R2,SPP1,STAT3	9
Cancer,Organismal Injury and Abnormalities	Lymphoreticular neoplasm	0,000021		-0,537	ACSL3,ACSL4,ALDH2,ALDH4A1,ALDH6A1,ALDH9A1,ANXA4,ARHGEF1,ATIC,ATP2B4,ATP6AP1,ATP6V1A,BIRC6,CARMIL1,CDK1,CDK4,CDK6,CRAT,CSNK1A1,CTBP2,CTTNBP2,DDB1,DGKZ,DGLUCY,DNAJC5,DNMT1,DOCK1,DYNC2H1,ECI2,EML1,ERLIN2,EXOC4,EXTL3,FADD,FASN,FBXO7,FDFT1,FLII,FLNA,GATM,GMDS,GMPPB,GOT2,GTF2B,HMMR,HMOX1,IDH2,IFT74,IKBIP,ITPR1,ITPR3,KANK2,KPNA2,LAMTOR2,LOXL3,LPP,NAPA,NARS1,NDC80,NFKB2,NQO1,NUP54,PAK1,PDE4DIP,PDZRN3,PIGU,POLA1,POLD1,PPAT,PPOX,PRKACB,PRKCA,PTPN1,RAD51,RAF1,RAI14,RASSF4,RBPJ,RPA1,RPL13,RPL3,RPL5,RPL6,RRM1,RRM2,SEC14L1,SNX1,SQSTM1,STAT1,STAT3,STAT5B,TCF12,TLE3,TLN1,TRIP12,TRIP13,TTC21B,UBE2C,UBE2I,USP8,USP9X,VAMP7,VCP,WDR62,WIPF1,XRN1	106
Cancer,Hematological Disease,Organismal Injury and Abnormalities	Neoplasia of blood cells	0,000118		-0,537	ACSL3,ACSL4,AGL,ALDH2,ALDH4A1,ALDH6A1,ALDH9A1,ANXA4,ARHGEF1,ATIC,ATP2B4,ATP6AP1,ATP6V1A,BIRC6,CARMIL1,CDC37L1,CDK1,CDK4,CDK6,CRAT,CSNK1A1,CTBP2,CTTNBP2,DDB1,DGKZ,DGLUCY,DNAJC5,DNMT1,DOCK1,DOCK5,DYNC2H1,ECI2,EEF2,EML1,ERLIN2,EXOC4,EXTL3,FADD,FASN,FBXO7,FDFT1,FLII,FLNA,GATM,GMDS,GMPPB,GOT2,GTF2B,HMMR,HMOX1,IDH2,IFT74,IKBIP,ITPR1,ITPR3,KANK2,KPNA2,LAMTOR2,LOXL3,LPP,MEGF10,NAPA,NARS1,NDC80,NFKB2,NPTXR,NQO1,NUP54,PAK1,PDE4DIP,PDZRN3,PIGU,POLA1,POLD1,PPAT,PPOX,PRKACB,PRKCA,PRPF19,PTPN1,RAD51,RAF1,RAI14,RASSF4,RBPJ,RPA1,RPL13,RPL3,RPL5,RPL6,RPL7A,RRM1,RRM2,SEC14L1,SEC24D,SNX1,SPP1,SQSTM1,SRGAP3,STAT1,STAT3,STAT5B,TAX1BP1,TCF12,TLE3,TLN1,TRIM3,TRIOBP,TRIP12,TRIP13,TTC21B,UBE2C,UBE2I,UCKL1,USP47,USP8,USP9X,VAMP7,VCP,WDR62,WIPF1,XRN1	122
Cancer,Hematological Disease,Organismal Injury and Abnormalities	Myeloid or lymphoid neoplasm	0,000297		-0,537	ACSL3,ACSL4,AGL,ALDH2,ALDH4A1,ALDH6A1,ALDH9A1,ANXA4,ARHGEF1,ATIC,ATP2B4,ATP6AP1,ATP6V1A,BIRC6,CARMIL1,CDC37L1,CDK1,CDK4,CDK6,CRAT,CSNK1A1,CTBP2,CTTNBP2,DDB1,DGKZ,DGLUCY,DNAJC5,DNMT1,DOCK1,DOCK5,DYNC2H1,ECI2,EEF2,EML1,ERLIN2,EXOC4,EXTL3,FADD,FASN,FBXO7,FDFT1,FLII,FLNA,GATM,GMDS,GMPPB,GOT2,GTF2B,HMMR,HMOX1,IDH2,IFT74,IKBIP,ITPR1,ITPR3,KANK2,KPNA2,LAMTOR2,LOXL3,LPP,MEGF10,NAPA,NARS1,NDC80,NFKB2,NPTXR,NQO1,NUP54,PAK1,PDE4DIP,PDZRN3,PIGU,POLA1,POLD1,PPAT,PPOX,PRKACB,PRKCA,PRPF19,PTPN1,RAD51,RAF1,RAI14,RASSF4,RBPJ,RPA1,RPL13,RPL3,RPL5,RPL6,RPL7A,RRM1,RRM2,SEC14L1,SEC24D,SNX1,SPP1,SQSTM1,SRGAP3,STAT1,STAT3,STAT5B,TAX1BP1,TCF12,TLE3,TLN1,TRIM3,TRIOBP,TRIP12,TRIP13,TTC21B,UBE2C,UBE2I,UCKL1,USP47,USP8,USP9X,VAMP7,VCP,WDR62,WIPF1,XRN1	122
Infectious Diseases	Replication of RNA virus	0,0000193		-0,514	AGFG1,ATP2A2,ATP6AP1,ATP6V1A,CDK4,CUL3,DDB1,DDX5,FASN,GCLC,HMOX1,KATNB1,MARK2,NEK9,NRBP1,NUP54,PCBP2,PRDX2,PRKCA,PTPN1,RAF1,SLC9A3R1,SPP1,SQSTM1,STAT1,STAT3,UBE2I,VCP,XRN1	29
Cancer,Organismal Injury and Abnormalities	Incidence of tumor	1,93E-10		-0,512	AARSD1,ABCD4,ACADS,ACO1,ACO2,ACSF2,ACSL3,ACSL4,ADD1,ADI1,AGL,AIP,AK3,AKR1B1,AKR1B10,AKR7A2,ALDH2,ALDH4A1,ALDH6A1,ANKRD13A,Anp32b,ANXA4,AP3S2,APPL2,ARFGAP2,ARFGAP3,ARFGEF2,ARHGEF1,ARHGEF12,ARHGEF18,ARMC8,ASRGL1,ATIC,ATP2A2,ATP6AP1,ATP6V1A,ATXN10,BCAS1,BCAT2,BIRC6,C1QBP,CACYBP,CAMSAP2,CAND1,CANX,CAVIN1,CBX3,CC2D1B,CCDC47,CCZ1/CCZ1B,CDIPT,CDK1,CDK4,CDK6,CEP55,CERT1,CNN2,CNPY2,CRAT,CSNK1A1,CTBP2,CTTNBP2,CUL3,CYB5R1,CYFIP1,DCTN6,DDB1,DDRGK1,DDX5,DGKZ,DGLUCY,DNAJC5,DNMT1,DOCK1,DOCK5,DPY19L1,DPYSL2,DPYSL3,DTYMK,DYNC2H1,EEF1B2,EEF2,EEF2K,EIF2B4,ELOA,ELP1,ELP2,ELP3,EMC7,EMD,EML1,ERLIN2,EXOC2,EXOC4,EXTL3,FAF2,FASN,FBXO7,FDFT1,FLII,FLNA,FLNB,FRMD4A,FUT8,GATM,GCDH,GCLM,GFPT1,GHDC,GLRX3,GLRX5,GMDS,GMPPB,GOT2,GPT2,GRB14,GSTZ1,GTF2B,GTSE1,HAUS7,HDGFL3,HEBP2,HM13,HMMR,HMOX1,HNRNPUL1,HSPA4L,IDH2,IFT74,IKBIP,IMPA1,INPPL1,IRGQ,ISCA2,ITPR1,ITPR3,ITPRID2,KANK1,KPNA2,LARP7,LAYN,LETM1,LIMD1,LOXL3,LPP,LRRC40,LTA4H,MARK2,MEGF10,MKLN1,MMUT,MON2,MTDH,MTSS1,NAA25,NAB2,NAPA,NCLN,NDC80,NEK9,NFKB2,NLE1,NMD3,NPTXR,NQO1,NRBP1,NRDC,NT5C3B,NUP54,OAT,OGT,OSBPL8,OXCT1,PAFAH1B1,PAK1,PAPSS1,PARG,PCBP2,PCK2,PDE4DIP,PDZRN3,PEPD,PFKM,PHLDA1,PHYH,PIGU,PITRM1,PKN2,PLCB1,POLA1,POLD1,POLD3,POLDIP3,PPAT,PPOX,PPP2R5D,PPP4R3A,PRDX2,PRKACB,PRKCA,PRPF19,PRRC2A,PRRX1,PTPN1,QSOX2,RAB32,RAD51,RAF1,RAI14,RANGAP1,RBPJ,RCN2,RER1,RETSAT,RIOK3,RNASEH2B,RPA1,RPL27,RPL5,RPN1,RPN2,RRM1,RRM2,SAMD4B,SDF4,SEC14L1,SEC24A,SEC24D,SEC61A1,SERPINE2,SLC1A4,SLC9A3R1,SLC9A3R2,SNF8,SNX1,SNX17,SOAT1,SORBS3,SPP1,SQLE,SQSTM1,SRGAP3,STAT1,STAT3,STAT5B,STT3A,SUCLG2,TAX1BP1,TCF12,TDRKH,TKFC,TLN1,TM9SF3,TM9SF4,TMEM115,TMEM43,TNS3,TOM1L2,TPD52,TRIM2,TRIM28,TRIM3,TRIM47,TRIOBP,TRIP10,TRIP12,TRIP13,TRMT2A,TST,UBAP2L,UBE2C,UBE2I,UCKL1,USP24,USP47,USP8,USP9X,VCP,VCPIP1,VPS11,VPS13A,VPS36,WDR12,WDR45B,WDR62,WFS1,WIPF1,XRN1,ZMPSTE24	289
Neurological Disease	Motor dysfunction or movement disorder	0,00000177		-0,474	AGA,AGL,ALDH2,ALDH6A1,ARHGEF1,ATP2A2,ATP6V1A,ATXN10,BCAS1,CANX,CWF19L1,DGKZ,DNAJC5,DNMT1,DPYSL3,EEF2,EML1,ERLIN2,FBXO7,FDFT1,FRMD4A,HMOX1,HMOX2,IMPA1,INPP1,ITPR1,KANK1,LOXL3,LPP,MTDH,NAB2,NAPA,NARS1,NDUFAF7,NQO1,OGT,OSBPL8,PAK1,PDE4DIP,PFKM,PHYH,PIP4P2,PITRM1,PLCB1,PRDX2,RAB6A,RAD51,RPL13,RPL3,SEC24A,SERPINE2,SGTA,SLC1A4,SPP1,SQSTM1,SRGAP3,STAT1,STRN4,TPD52,USP24,VPS13A,ZMPSTE24	62
Cellular Assembly and Organization,Cellular Function and Maintenance	Quantity of lamellipodia	0,0000142		-0,447	DOCK1,DOCK5,PAK1,SLC9A3R2,STAT3	5
Infectious Diseases	Replication of virus	0,00000353		-0,419	AGFG1,ATP2A2,ATP6AP1,ATP6V1A,CDK4,CTBP2,CUL3,DDB1,DDX5,FADD,FASN,GCLC,HMOX1,KATNB1,MARK2,NEK9,NRBP1,NUP54,OGT,PCBP2,POLA1,PRDX2,PRKCA,PTPN1,RAF1,SLC9A3R1,SPP1,SQSTM1,STAT1,STAT3,UBE2I,VCP,XRN1	33
Cancer,Organismal Injury and Abnormalities	Epithelial neoplasm	8,67E-21		-0,408	AARSD1,ABCD4,ACAA2,ACADS,ACO1,ACO2,ACP2,ACSF2,ACSL3,ACSL4,ADD1,ADI1,ADO,AGA,AGFG1,AGL,AIP,AK3,AKR1B1,AKR1B10,AKR7A2,ALDH2,ALDH4A1,ALDH6A1,ALDH9A1,ANAPC7,ANKRD13A,ANXA4,AP3S2,APPL2,ARFGAP2,ARFGAP3,ARFGEF2,ARHGEF1,ARHGEF12,ARHGEF18,ARMC8,ASRGL1,ATIC,ATP2A2,ATP2B4,ATP6AP1,ATP6V1A,ATPAF2,ATXN10,BCAS1,BCAT2,BIRC6,C17orf49,C1QBP,CACYBP,CAMSAP2,CAND1,CANX,CARMIL1,CAVIN1,CBX3,CC2D1B,CCDC47,CDC37L1,CDC42EP4,CDIPT,CDK1,CDK4,CDK6,CEP55,CERT1,CNEP1R1,CNN2,CNN3,CNPY2,CRAT,CSNK1A1,CSRP1,CTBP2,CTTNBP2,CUL3,CWF19L1,CYB5R1,CYFIP1,DCTN6,DDB1,DDRGK1,DDX5,DGKZ,DGLUCY,DNAJC5,DNAJC9,DNMT1,DNPEP,DOCK1,DOCK5,DPY19L1,DPYSL2,DPYSL3,DTYMK,DYNC2H1,ECI2,EEF1B2,EEF2,EEF2K,EIF2B4,EIF2B5,ELOA,ELP1,ELP2,ELP3,EMC7,EMD,EML1,ERLIN2,EXOC2,EXOC4,EXTL3,FADD,FADS1,FAF2,FASN,FBXL15,FBXO7,FDFT1,FLII,FLNA,FLNB,FRMD4A,FUT8,GABPA,GATM,GCDH,GCLC,GCLM,GDA,GFPT1,GHDC,GLRX,GLRX3,GLRX5,GMDS,GMPPB,GOT2,GPT2,GRB14,GSDMD,GSTZ1,GTF2B,GTSE1,HACD3,HAUS7,HDGFL3,HEBP2,HIKESHI,HM13,HMGCL,HMGCS1,HMMR,HMOX1,HMOX2,HNRNPUL1,HSPA4L,IDH2,IFT74,IKBIP,IMPA1,INPP1,INPPL1,IRGQ,ISCA2,ITPR1,ITPR3,ITPRID2,KANK1,KANK2,KATNB1,KPNA2,LAMTOR2,LAMTOR3,LARP7,LAYN,LETM1,LIMD1,LOXL3,LPCAT3,LPP,LRRC40,LRRC59,LTA4H,MARK2,MAT2A,MEGF10,MGST2,MKLN1,MMUT,MON2,MPI,MPST,MTDH,MTSS1,NAA25,NAB2,NAPA,NAXE,NCLN,NDC80,NDUFAF7,NEK9,NFKB2,NFS1,NIT2,NLE1,NLN,NMD3,NPTXR,NQO1,NRBP1,NRDC,NT5C3B,NUP54,OAT,OGT,OSBPL8,OXCT1,PAFAH1B1,PAK1,PAPSS1,PARG,PCBP2,PCK2,PDE4DIP,PDZRN3,PEPD,PFKM,PGD,PHLDA1,PHYH,PIGU,PIP4P2,PITRM1,PKN2,PLCB1,PMPCB,POLA1,POLD1,POLD3,POLDIP3,PPA2,PPAT,PPOX,PPP2R5D,PPP4R3A,PRDX2,PRKACB,PRKCA,PRPF19,PRRC2A,PRRX1,PTPN1,QSOX2,RAB32,RAB6A,RABGGTA,RAD51,RAF1,RAI14,RANGAP1,RAP2C,RASSF4,RBPJ,RCN2,RER1,RETSAT,RIOK3,RNASEH2B,RPA1,RPA2,RPL13,RPL27,RPL3,RPL5,RPL6,RPL7A,RPN1,RPN2,RRM1,RRM2,SAMD4B,SDF4,SEC14L1,SEC24A,SEC24D,SEC61A1,SERPINE2,SFR1,SGTA,SH3GLB1,SLC1A4,SLC9A3R1,SLC9A3R2,SNF8,SNX1,SNX17,SOAT1,SORBS3,SPP1,SQLE,SQSTM1,SRGAP3,SRXN1,STAM2,STARD3NL,STAT1,STAT3,STAT5B,STRN4,STT3A,SUCLG2,TAGLN2,TAX1BP1,TCF12,TDRKH,TKFC,TLE3,TLN1,TM9SF3,TM9SF4,TMEM115,TMEM43,TNS3,TOM1L2,TPD52,TRAPPC4,TRIM2,TRIM28,TRIM3,TRIM47,TRIOBP,TRIP10,TRIP12,TRIP13,TRMT2A,TST,TTC21B,UBAP2L,UBE2C,UBE2I,UBE2O,UBR7,UCKL1,USP24,USP47,USP8,USP9X,VAMP7,VAT1,VCP,VCPIP1,VPS11,VPS13A,VPS36,VPS4B,VWA5A,WDR12,WDR45B,WDR62,WFS1,WIPF1,XRN1,ZMPSTE24	367
Cancer,Organismal Injury and Abnormalities	Non-melanoma solid tumor	5,34E-22		-0,356	AARSD1,ABCD4,ACAA2,ACADS,ACO1,ACO2,ACP2,ACSF2,ACSL3,ACSL4,ADD1,ADI1,ADO,AGA,AGFG1,AGL,AIP,AK3,AKR1B1,AKR1B10,AKR7A2,ALDH2,ALDH4A1,ALDH6A1,ALDH9A1,ANAPC7,ANKRD13A,ANXA4,AP3S2,APPL2,ARFGAP2,ARFGAP3,ARFGEF2,ARHGEF1,ARHGEF12,ARHGEF18,ARMC8,ARPIN/ARPIN-AP3S2,ASRGL1,ATIC,ATP2A2,ATP2B4,ATP6AP1,ATP6V1A,ATPAF2,ATXN10,BCAS1,BCAT2,BIRC6,C17orf49,C1QBP,CACYBP,CAMSAP2,CAND1,CANX,CARMIL1,CAVIN1,CBX3,CC2D1B,CCDC47,CCZ1/CCZ1B,CDC37L1,CDC42EP4,CDIPT,CDK1,CDK4,CDK6,CEP55,CERT1,CNEP1R1,CNN2,CNN3,CNPY2,CRAT,CSNK1A1,CSRP1,CTBP2,CTTNBP2,CUL3,CWF19L1,CYB5R1,CYFIP1,DCTN6,DDB1,DDRGK1,DDX5,DGKZ,DGLUCY,DNAJC5,DNAJC9,DNMT1,DNPEP,DOCK1,DOCK5,DPY19L1,DPYSL2,DPYSL3,DTYMK,DYNC2H1,ECI2,EEF1B2,EEF2,EEF2K,EIF2B4,EIF2B5,ELOA,ELP1,ELP2,ELP3,EMC7,EMD,EML1,ERLIN2,EXOC2,EXOC4,EXTL3,FADD,FADS1,FAF2,FASN,FBXL15,FBXO7,FDFT1,FLII,FLNA,FLNB,FRMD4A,FUT8,GABPA,GATM,GCDH,GCLC,GCLM,GDA,GFPT1,GHDC,GLRX,GLRX3,GLRX5,GMDS,GMPPB,GOT2,GPT2,GRB14,GSDMD,GSTZ1,GTF2B,GTSE1,HACD3,HAUS7,HDGFL3,HEBP2,HIKESHI,HM13,HMGCL,HMGCS1,HMMR,HMOX1,HMOX2,HNRNPUL1,HSPA4L,IDH2,IFT74,IKBIP,IMPA1,INPP1,INPPL1,IRGQ,ISCA2,ITPR1,ITPR3,ITPRID2,KANK1,KANK2,KATNB1,KPNA2,LAMTOR2,LAMTOR3,LARP7,LAYN,LETM1,LIMD1,LOXL3,LPCAT3,LPP,LRRC40,LRRC59,LTA4H,MARK2,MAT2A,MEGF10,MGST2,MKLN1,MMUT,MON2,MPI,MPST,MTDH,MTSS1,NAA25,NAB2,NAPA,NARS1,NAXE,NCLN,NDC80,NDUFAF7,NEK9,NFKB2,NFS1,NIT2,NLE1,NLN,NMD3,NPTXR,NQO1,NRBP1,NRDC,NT5C3B,NUP54,OAT,OGT,OSBPL8,OXCT1,PAFAH1B1,PAK1,PAPSS1,PARG,PCBP2,PCK2,PDE4DIP,PDZRN3,PEPD,PFKM,PGD,PHLDA1,PHYH,PIGU,PIP4P2,PITRM1,PKN2,PLCB1,PMPCB,POLA1,POLD1,POLD3,POLDIP3,PPA2,PPAT,PPOX,PPP2R5D,PPP4R3A,PRDX2,PRKACB,PRKCA,PRPF19,PRRC2A,PRRX1,PTPN1,QSOX2,RAB32,RAB6A,RABGGTA,RAD51,RAF1,RAI14,RANGAP1,RAP2C,RASSF4,RBPJ,RCN2,RER1,RETSAT,RIOK3,RNASEH2B,RPA1,RPA2,RPL13,RPL27,RPL3,RPL5,RPL6,RPL7A,RPN1,RPN2,RRM1,RRM2,SAMD4B,SDF4,SEC14L1,SEC24A,SEC24D,SEC61A1,SERPINE2,SFR1,SGTA,SH3GLB1,SLC1A4,SLC9A3R1,SLC9A3R2,SNF8,SNX1,SNX17,SOAT1,SORBS3,SPCS3,SPP1,SQLE,SQSTM1,SRGAP3,SRXN1,STAM2,STARD3NL,STAT1,STAT3,STAT5B,STRN4,STT3A,SUCLG2,TAGLN2,TAX1BP1,TCF12,TDRKH,TKFC,TLE3,TLN1,TM9SF3,TM9SF4,TMEM115,TMEM43,TNS3,TOM1L2,TPD52,TRAPPC4,TRIM2,TRIM28,TRIM3,TRIM47,TRIOBP,TRIP10,TRIP12,TRIP13,TRMT2A,TST,TTC21B,UBAP2L,UBE2C,UBE2I,UBE2O,UBR7,UCKL1,USP24,USP47,USP8,USP9X,VAMP7,VAT1,VCP,VCPIP1,VPS11,VPS13A,VPS36,VPS4B,VWA5A,WDR12,WDR45B,WDR62,WFS1,WIPF1,XRN1,ZMPSTE24	371

Categories	Diseases or Functions Annotation	p-value	Predicted Activation State	Activation z-score	Molecules	# Molecules
Cancer,Organismal Injury and Abnormalities	Neoplasia of cells	0,000116		-0,288	ACO2,ACSL3,ACSL4,ADD1,ADI1,AGA,AGL,ALDH2,ALDH4A1,ALDH6A1,ALDH9A1,ANXA4,ARHGEF1,ARHGEF12,ASRGL1,ATIC,ATP2A2,ATP2B4,ATP6AP1,ATP6V1A,BCAS1,BIRC6,CACYBP,CAMSAP2,CARMIL1,CAVIN1,CDC37L1,CDK1,CDK4,CDK6,CERT1,CNN2,CNPY2,CRAT,CSNK1A1,CTBP2,CTTNBP2,CUL3,CYFIP1,DDB1,DDX5,DGKZ,DGLUCY,DNAJC5,DNAJC9,DNMT1,DOCK1,DOCK5,DPYSL3,DYNC2H1,ECI2,EEF2,ELP1,EMD,EML1,ERLIN2,EXOC4,EXTL3,FADD,FASN,FBXL15,FBXO7,FDFT1,FLII,FLNA,FUT8,GATM,GCDH,GCLC,GDA,GLRX5,GMDS,GMPPB,GOT2,GPT2,GTF2B,HM13,HMMR,HMOX1,HNRNPUL1,IDH2,IFT74,IKBIP,INPPL1,ITPR1,ITPR3,ITPRID2,KANK1,KANK2,KPNA2,LAMTOR2,LOXL3,LPP,MARK2,MEGF10,MPI,MTDH,MTSS1,NAA25,NAPA,NARS1,NDC80,NFKB2,NPTXR,NQO1,NRDC,NUP54,OAT,OGT,PAFAH1B1,PAK1,PDE4DIP,PDZRN3,PHLDA1,PIGU,PITRM1,PLCB1,PMPCB,POLA1,POLD1,PPAT,PPOX,PPP4R3A,PRDX2,PRKACB,PRKCA,PRPF19,PRRC2A,PTPN1,QSOX2,RAB6A,RAD51,RAF1,RAI14,RASSF4,RBPJ,RETSAT,RIOK3,RPA1,RPL13,RPL3,RPL5,RPL6,RPL7A,RPN2,RRM1,RRM2,SAMD4B,SEC14L1,SEC24D,SLC9A3R2,SNX1,SOAT1,SPP1,SQSTM1,SRGAP3,STAT1,STAT3,STAT5B,TAX1BP1,TCF12,TKFC,TLE3,TLN1,TPD52,TRIM28,TRIM3,TRIM47,TRIOBP,TRIP10,TRIP12,TRIP13,TRMT2A,TTC21B,UBAP2L,UBE2C,UBE2I,UBR7,UCKL1,USP24,USP47,USP8,USP9X,VAMP7,VCP,VPS13A,WDR62,WIPF1,XRN1	189
Post-Translational Modification	Ubiquitination	0,000253		-0,266	BIRC6,CAND1,CUL3,DDB1,FBXL15,FBXO7,GCLC,OGT,PCBP2,PDZRN3,PRPF19,SQSTM1,TRIM3,TRIM47,TRIP12,UBE2C,UBE2O,USP8,USP9X,VCP,VCPIP1,VPS11,WFS1	23
Cell Death and Survival	Cell death of pancreatic cancer cell lines	0,000011		-0,158	ACACA,ATP2A2,EXOC2,FADD,GLRX,HMMR,OGT,RAF1,RRM1,SPP1,STAT1,STAT3,UBE2C,VCP	14
Cancer,Hematological Disease,Organismal Injury and Abnormalities	Hematopoietic neoplasm	0,000276		-0,152	ACSL3,ACSL4,AGA,AGL,ALDH2,ALDH4A1,ALDH6A1,ALDH9A1,ANXA4,ARHGEF1,ATIC,ATP2B4,ATP6AP1,ATP6V1A,BIRC6,CARMIL1,CDC37L1,CDK1,CDK4,CDK6,CRAT,CSNK1A1,CTBP2,CTTNBP2,DDB1,DGKZ,DGLUCY,DNAJC5,DNMT1,DOCK1,DOCK5,DYNC2H1,ECI2,EEF2,EML1,ERLIN2,EXOC4,EXTL3,FADD,FASN,FBXO7,FDFT1,FLII,FLNA,GATM,GMDS,GMPPB,GOT2,GTF2B,HMMR,HMOX1,IDH2,IFT74,IKBIP,ITPR1,ITPR3,KANK2,KPNA2,LAMTOR2,LOXL3,LPP,MEGF10,NAPA,NARS1,NDC80,NFKB2,NPTXR,NQO1,NUP54,PAK1,PDE4DIP,PDZRN3,PIGU,POLA1,POLD1,PPAT,PPOX,PRKACB,PRKCA,PRPF19,PTPN1,RAD51,RAF1,RAI14,RASSF4,RBPJ,RPA1,RPL13,RPL3,RPL5,RPL6,RPL7A,RRM1,RRM2,SEC14L1,SEC24D,SNX1,SPP1,SQSTM1,SRGAP3,STAT1,STAT3,STAT5B,TAX1BP1,TCF12,TLE3,TLN1,TRIM3,TRIOBP,TRIP12,TRIP13,TTC21B,UBE2C,UBE2I,UCKL1,USP47,USP8,USP9X,VAMP7,VCP,WDR62,WIPF1,XRN1	123
Cancer,Organismal Injury and Abnormalities	Nonhematologic malignant neoplasm	1,46E-19		-0,139	AARSD1,ABCD4,ACAA2,ACADS,ACO1,ACO2,ACP2,ACSF2,ACSL3,ACSL4,ADD1,ADI1,ADO,AGA,AGFG1,AGL,AIP,AK3,AKR1B1,AKR1B10,AKR7A2,ALDH2,ALDH4A1,ALDH6A1,ALDH9A1,ANAPC7,ANKRD13A,ANKRD46,ANXA4,AP3S2,APPL2,ARFGAP2,ARFGAP3,ARFGEF2,ARHGEF1,ARHGEF12,ARHGEF18,ARMC8,ARPIN/ARPIN-AP3S2,ASRGL1,ATIC,ATP2A2,ATP2B4,ATP6AP1,ATP6V1A,ATPAF2,ATXN10,BCAS1,BCAT2,BIRC6,C17orf49,C1QBP,CACYBP,CAMSAP2,CAND1,CANX,CARMIL1,CAVIN1,CBX3,CC2D1B,CCDC47,CDC37L1,CDC42EP4,CDIPT,CDK1,CDK4,CDK6,CEP55,CERT1,CNEP1R1,CNN2,CNN3,CNPY2,CRAT,CSNK1A1,CSRP1,CTBP2,CTTNBP2,CUL3,CWF19L1,CYB5R1,CYFIP1,DCTN6,DDB1,DDRGK1,DDX5,DGKZ,DGLUCY,DNAJC5,DNAJC9,DNMT1,DNPEP,DOCK1,DOCK5,DPY19L1,DPYSL2,DPYSL3,DTYMK,DYNC2H1,ECI2,EEF1B2,EEF2,EEF2K,EIF2B4,EIF2B5,ELOA,ELP1,ELP2,ELP3,EMC7,EMD,EML1,ERLIN2,EXOC2,EXOC4,EXTL3,FADD,FADS1,FAF2,FASN,FBXL15,FBXO7,FDFT1,FLII,FLNA,FLNB,FRMD4A,FUT8,GABPA,GATM,GCDH,GCLC,GCLM,GDA,GFPT1,GHDC,GLRX3,GLRX5,GMDS,GMPPB,GOT2,GPT2,GRB14,GSDMD,GSTZ1,GTF2B,GTSE1,HACD3,HAUS7,HDGFL3,HEBP2,HIKESHI,HM13,HMGCL,HMGCS1,HMMR,HMOX1,HMOX2,HNRNPUL1,HSPA4L,IDH2,IFT74,IKBIP,IMPA1,INPP1,INPPL1,IRGQ,ISCA2,ITPR1,ITPR3,ITPRID2,KANK1,KANK2,KATNB1,KPNA2,LAMTOR2,LAMTOR3,LARP7,LAYN,LETM1,LIMD1,LOXL3,LPCAT3,LPP,LRRC40,LRRC59,LTA4H,MARK2,MAT2A,MEGF10,MGST2,MKLN1,MMUT,MON2,MPI,MPST,MTDH,MTSS1,NAA25,NAB2,NAPA,NAXE,NCLN,NDC80,NDUFAF7,NEK9,NFKB2,NFS1,NIT2,NLE1,NLN,NMD3,NPTXR,NQO1,NRBP1,NRDC,NT5C3B,NUP54,OAT,OGT,OSBPL8,OXCT1,PAFAH1B1,PAK1,PAPSS1,PARG,PCBP2,PCK2,PDE4DIP,PDZRN3,PEPD,PFKM,PGD,PHLDA1,PHYH,PIGU,PIP4P2,PITRM1,PKN2,PLCB1,PMPCB,POLA1,POLD1,POLD3,POLDIP3,PPA2,PPAT,PPOX,PPP2R5D,PPP4R3A,PRDX2,PRKACB,PRKCA,PRPF19,PRRC2A,PRRX1,PTPN1,QSOX2,RAB32,RAB6A,RABGGTA,RAD51,RAF1,RAI14,RANGAP1,RAP2C,RASSF4,RBPJ,RCN2,RER1,RETSAT,RIOK3,RNASEH2B,RPA1,RPA2,RPL13,RPL27,RPL3,RPL5,RPL6,RPL7A,RPN1,RPN2,RRM1,RRM2,SAMD4B,SDF4,SEC14L1,SEC24A,SEC24D,SEC61A1,SERPINE2,SFR1,SGTA,SH3GLB1,SLC1A4,SLC9A3R1,SLC9A3R2,SNF8,SNX1,SNX17,SOAT1,SORBS3,SPCS3,SPP1,SQLE,SQSTM1,SRGAP3,SRXN1,STAM2,STARD3NL,STAT1,STAT3,STAT5B,STRN4,STT3A,SUCLG2,TAGLN2,TAX1BP1,TCF12,TDRKH,TKFC,TLE3,TLN1,TM9SF3,TM9SF4,TMEM115,TMEM43,TNS3,TOM1L2,TPD52,TRAPPC4,TRIM2,TRIM28,TRIM3,TRIM47,TRIOBP,TRIP10,TRIP12,TRIP13,TRMT2A,TST,TTC21B,UBAP2L,UBE2C,UBE2O,UBR7,UCKL1,USP24,USP47,USP8,USP9X,VAMP7,VAT1,VCP,VCPIP1,VPS11,VPS13A,VPS36,VPS4B,VWA5A,WDR12,WDR45B,WDR62,WFS1,WIPF1,XRN1,ZMPSTE24	368
Protein Synthesis	Metabolism of protein	0,00000381		-0,121	ACO1,ADI1,ANAPC7,ARHGEF1,CANX,CCDC47,CDK4,CUL3,CYFIP1,DDB1,DDRGK1,DNMT1,EEF1B2,EEF2,EEF2K,EIF2B4,EIF2B5,FADD,FBXO7,FLNA,GTSE1,INPPL1,LTA4H,NCLN,NDC80,NLN,NRDC,OGT,PITRM1,POLDIP3,PPAT,PRKCA,RPL13,RPL27,RPL3,RPL5,RPL6,RPL7A,SERPINE2,SH3GLB1,SNF8,SNX1,SPCS3,SPP1,SQSTM1,STAT3,TRIP12,TST,UBE2C,UBE2I,USP8,USP9X,VCP,VPS11,WFS1,XRN1,ZMPSTE24	57
Cancer,Hematological Disease,Organismal Injury and Abnormalities	Hematologic cancer	0,000208		-0,115	ACSL3,ACSL4,AGL,ALDH2,ALDH4A1,ALDH6A1,ALDH9A1,ANXA4,ARHGEF1,ATIC,ATP2B4,ATP6AP1,ATP6V1A,BIRC6,CARMIL1,CDC37L1,CDK1,CDK4,CDK6,CRAT,CSNK1A1,CTBP2,CTTNBP2,DDB1,DGKZ,DGLUCY,DNAJC5,DNMT1,DOCK1,DOCK5,DYNC2H1,ECI2,EEF2,EML1,ERLIN2,EXOC4,EXTL3,FADD,FASN,FBXO7,FDFT1,FLII,FLNA,GATM,GMDS,GMPPB,GOT2,GTF2B,HMMR,HMOX1,IDH2,IFT74,IKBIP,ITPR1,ITPR3,KANK2,KPNA2,LAMTOR2,LOXL3,LPP,MEGF10,NAPA,NARS1,NDC80,NFKB2,NPTXR,NQO1,NUP54,PAK1,PDE4DIP,PDZRN3,PIGU,POLA1,POLD1,PPAT,PPOX,PRKACB,PRKCA,PRPF19,PTPN1,RAD51,RAF1,RAI14,RASSF4,RBPJ,RPA1,RPL13,RPL3,RPL5,RPL6,RPL7A,RRM1,RRM2,SEC14L1,SEC24D,SNX1,SPP1,SQSTM1,SRGAP3,STAT1,STAT3,STAT5B,TAX1BP1,TCF12,TLE3,TLN1,TRIM3,TRIOBP,TRIP12,TRIP13,TTC21B,UBE2C,UBE2I,UCKL1,USP47,USP8,USP9X,VAMP7,VCP,WDR62,WIPF1,XRN1	122
Cell-To-Cell Signaling and Interaction	Response of tumor cell lines	0,000168		-0,095	APPL2,ATP2A2,CYFIP1,DGKZ,FADD,HMOX1,LAMTOR2,MEGF10,NQO1,POLA1,STAT3,STT3A,TLE3,TLN1,USP9X,VCP	16
Cell Death and Survival	Apoptosis of cervical cancer cell lines	0,0000732		-0,08	CDK1,EXOC2,FADD,FLNB,GTSE1,ITPR1,NAPA,NDC80,PAK1,PCBP2,PRPF19,RAB32,RAF1,RPA1,SDF4,STAT1,STAT3,TNFAIP8,UBE2I	19
Cellular Assembly and Organization,Cellular Function and Maintenance,Tissue Development	Formation of actin filaments	0,000229		-0,037	ADD1,ARHGEF1,ARHGEF12,ARHGEF18,CARMIL1,CDK4,CDK6,CUL3,Cyb5r3,KANK1,KANK2,MARK2,MTSS1,PAK1,PKN2,SORBS3,STAT3,TNS3,TRIP10	19
Carbohydrate Metabolism	Metabolism of carbohydrate	0,000235		0	ACSL3,AGL,AKR1B1,AKR1B10,AKR7A2,ALDH2,CDIPT,CTBP2,DGKZ,EXTL3,FUT8,GCLC,GFPT1,GMDS,GMPPB,HMMR,IMPA1,INPP1,INPPL1,MPI,NQO1,OGT,PAK1,PFKM,PGD,PIGU,PLCB1,PPAT,PRDX2,PTPN1,SH3GLB1,SLC9A3R1,SPP1,STAT3,Tpi1 (includes others)	35
Cancer,Organismal Injury and Abnormalities	Head and neck cancer	4,37E-21		0	AARSD1,ABCD4,ACAA2,ACADS,ACO1,ACO2,ACP2,ACSF2,ACSL3,ACSL4,ADD1,ADI1,ADO,AGA,AGFG1,AGL,AIP,AK3,AKR1B1,AKR1B10,AKR7A2,ALDH2,ALDH4A1,ALDH6A1,ALDH9A1,ANAPC7,ANKRD13A,APPL2,ARFGAP2,ARFGAP3,ARFGEF2,ARHGEF1,ARHGEF12,ARHGEF18,ARMC8,ARPIN/ARPIN-AP3S2,ASRGL1,ATIC,ATP2A2,ATP2B4,ATP6AP1,ATP6V1A,ATPAF2,ATXN10,BCAS1,BCAT2,BIRC6,C17orf49,C1QBP,CAMSAP2,CAND1,CANX,CARMIL1,CAVIN1,CBX3,CC2D1B,CCDC47,CDC37L1,CDC42EP4,CDIPT,CDK1,CDK4,CDK6,CEP55,CNEP1R1,CNN2,CNN3,CRAT,CSNK1A1,CSRP1,CTBP2,CTTNBP2,CUL3,CWF19L1,CYB5R1,CYFIP1,DDB1,DDRGK1,DDX5,DGKZ,DGLUCY,DNAJC9,DNMT1,DNPEP,DOCK1,DOCK5,DPY19L1,DPYSL2,DPYSL3,DTYMK,DYNC2H1,EEF1B2,EEF2,EEF2K,EIF2B4,ELOA,ELP1,ELP2,ELP3,EMD,EML1,EXOC2,EXOC4,FADD,FADS1,FASN,FBXL15,FBXO7,FDFT1,FLII,FLNA,FLNB,FRMD4A,FUT8,GABPA,GATM,GCDH,GCLC,GCLM,GDA,GFPT1,GHDC,GLRX3,GLRX5,GMDS,GMPPB,GOT2,GPT2,GSDMD,GTF2B,GTSE1,HACD3,HDGFL3,HEBP2,HM13,HMGCL,HMGCS1,HMMR,HMOX1,HMOX2,HNRNPUL1,HSPA4L,IDH2,IFT74,INPPL1,IRGQ,ITPR1,ITPR3,ITPRID2,KANK1,KANK2,KATNB1,KPNA2,LAMTOR3,LARP7,LETM1,LIMD1,LOXL3,LPCAT3,LPP,LRRC40,LRRC59,MARK2,MAT2A,MEGF10,MGST2,MKLN1,MMUT,MON2,MPI,MPST,MTDH,MTSS1,NAA25,NAB2,NAPA,NAXE,NCLN,NDC80,NDUFAF7,NEK9,NFKB2,NFS1,NIT2,NLE1,NLN,NPTXR,NQO1,NRBP1,NRDC,NT5C3B,NUP54,OAT,OGT,OSBPL8,OXCT1,PAFAH1B1,PAK1,PAPSS1,PARG,PCBP2,PCK2,PDE4DIP,PDZRN3,PEPD,PFKM,PGD,PHLDA1,PHYH,PIGU,PITRM1,PKN2,PLCB1,PMPCB,POLA1,POLD1,POLD3,POLDIP3,PPA2,PPAT,PPOX,PPP2R5D,PPP4R3A,PRDX2,PRKACB,PRKCA,PRPF19,PRRC2A,PTPN1,QSOX2,RAB32,RAB6A,RABGGTA,RAD51,RAF1,RAI14,RANGAP1,RASSF4,RBPJ,RCN2,RETSAT,RIOK3,RNASEH2B,RPA1,RPA2,RPL13,RPL3,RPL6,RPL7A,RPN1,RPN2,RRM1,RRM2,SAMD4B,SEC14L1,SEC24A,SEC24D,SERPINE2,SFR1,SGTA,SH3GLB1,SLC1A4,SLC9A3R1,SLC9A3R2,SNF8,SNX1,SNX17,SOAT1,SORBS3,SPP1,SQLE,SQSTM1,SRGAP3,SRXN1,STAM2,STAT1,STAT3,STAT5B,STRN4,STT3A,SUCLG2,TAGLN2,TAX1BP1,TCF12,TKFC,TLE3,TLN1,TM9SF3,TM9SF4,TMEM115,TMEM43,TNS3,TOM1L2,TPD52,TRAPPC4,TRIM2,TRIM28,TRIM3,TRIM47,TRIOBP,TRIP10,TRIP12,TRIP13,TRMT2A,TST,TTC21B,UBAP2L,UBE2C,UBE2I,UBE2O,UBR7,UCKL1,USP24,USP47,USP8,USP9X,VAMP7,VAT1,VCP,VPS11,VPS13A,VPS36,VWA5A,WDR12,WDR45B,WDR62,WFS1,WIPF1,XRN1,ZMPSTE24	330
Cancer,Organismal Injury and Abnormalities	Head and neck tumor	1,75E-20		0	AARSD1,ABCD4,ACAA2,ACADS,ACO1,ACO2,ACP2,ACSF2,ACSL3,ACSL4,ADD1,ADI1,ADO,AGA,AGFG1,AGL,AIP,AK3,AKR1B1,AKR1B10,AKR7A2,ALDH2,ALDH4A1,ALDH6A1,ALDH9A1,ANAPC7,ANKRD13A,AP3S2,APPL2,ARFGAP2,ARFGAP3,ARFGEF2,ARHGEF1,ARHGEF12,ARHGEF18,ARMC8,ARPIN/ARPIN-AP3S2,ASRGL1,ATIC,ATP2A2,ATP2B4,ATP6AP1,ATP6V1A,ATPAF2,ATXN10,BCAS1,BCAT2,BIRC6,C17orf49,C1QBP,CACYBP,CAMSAP2,CAND1,CANX,CARMIL1,CAVIN1,CBX3,CC2D1B,CCDC47,CDC37L1,CDC42EP4,CDIPT,CDK1,CDK4,CDK6,CEP55,CNEP1R1,CNN2,CNN3,CRAT,CSNK1A1,CSRP1,CTBP2,CTTNBP2,CUL3,CWF19L1,CYB5R1,CYFIP1,DDB1,DDRGK1,DDX5,DGKZ,DGLUCY,DNAJC9,DNMT1,DNPEP,DOCK1,DOCK5,DPY19L1,DPYSL2,DPYSL3,DTYMK,DYNC2H1,EEF1B2,EEF2,EEF2K,EIF2B4,EIF2B5,ELOA,ELP1,ELP2,ELP3,EMD,EML1,ERLIN2,EXOC2,EXOC4,FADD,FADS1,FASN,FBXL15,FBXO7,FDFT1,FLII,FLNA,FLNB,FRMD4A,FUT8,GABPA,GATM,GCDH,GCLC,GCLM,GDA,GFPT1,GHDC,GLRX3,GLRX5,GMDS,GMPPB,GOT2,GPT2,GRB14,GSDMD,GTF2B,GTSE1,HACD3,HDGFL3,HEBP2,HM13,HMGCL,HMGCS1,HMMR,HMOX1,HMOX2,HNRNPUL1,HSPA4L,IDH2,IFT74,INPPL1,IRGQ,ITPR1,ITPR3,ITPRID2,KANK1,KANK2,KATNB1,KPNA2,LAMTOR3,LARP7,LETM1,LIMD1,LOXL3,LPCAT3,LPP,LRRC40,LRRC59,MARK2,MAT2A,MEGF10,MGST2,MKLN1,MMUT,MON2,MPI,MPST,MTDH,MTSS1,NAA25,NAB2,NAPA,NAXE,NCLN,NDC80,NDUFAF7,NEK9,NFKB2,NFS1,NIT2,NLE1,NLN,NPTXR,NQO1,NRBP1,NRDC,NT5C3B,NUP54,OAT,OGT,OSBPL8,OXCT1,PAFAH1B1,PAK1,PAPSS1,PARG,PCBP2,PCK2,PDE4DIP,PDZRN3,PEPD,PFKM,PGD,PHLDA1,PHYH,PIGU,PIP4P2,PITRM1,PKN2,PLCB1,PMPCB,POLA1,POLD1,POLD3,POLDIP3,PPA2,PPAT,PPOX,PPP2R5D,PPP4R3A,PRDX2,PRKACB,PRKCA,PRPF19,PRRC2A,PTPN1,QSOX2,RAB32,RAB6A,RABGGTA,RAD51,RAF1,RAI14,RANGAP1,RASSF4,RBPJ,RCN2,RETSAT,RIOK3,RNASEH2B,RPA1,RPA2,RPL13,RPL3,RPL5,RPL6,RPL7A,RPN1,RPN2,RRM1,RRM2,SAMD4B,SDF4,SEC14L1,SEC24A,SEC24D,SERPINE2,SFR1,SGTA,SH3GLB1,SLC1A4,SLC9A3R1,SLC9A3R2,SNF8,SNX1,SNX17,SOAT1,SORBS3,SPCS3,SPP1,SQLE,SQSTM1,SRGAP3,SRXN1,STAM2,STARD3NL,STAT1,STAT3,STAT5B,STRN4,STT3A,SUCLG2,TAGLN2,TAX1BP1,TCF12,TDRKH,TKFC,TLE3,TLN1,TM9SF3,TM9SF4,TMEM115,TMEM43,TNS3,TOM1L2,TPD52,TRAPPC4,TRIM2,TRIM28,TRIM3,TRIM47,TRIOBP,TRIP10,TRIP12,TRIP13,TRMT2A,TST,TTC21B,UBAP2L,UBE2C,UBE2I,UBE2O,UBR7,UCKL1,USP24,USP47,USP8,USP9X,VAMP7,VAT1,VCP,VCPIP1,VPS11,VPS13A,VPS36,VWA5A,WDR12,WDR45B,WDR62,WFS1,WIPF1,XRN1,ZMPSTE24	342
Cancer,Organismal Injury and Abnormalities	Head and neck carcinoma	1,01E-19		0	AARSD1,ACAA2,ACADS,ACO1,ACO2,ACP2,ACSF2,ACSL3,ACSL4,ADD1,ADI1,ADO,AGA,AGFG1,AGL,AIP,AK3,AKR1B1,AKR7A2,ALDH2,ALDH4A1,ALDH6A1,ALDH9A1,ANAPC7,ANKRD13A,APPL2,ARFGAP2,ARFGAP3,ARFGEF2,ARHGEF1,ARHGEF12,ARHGEF18,ARMC8,ASRGL1,ATIC,ATP2A2,ATP2B4,ATP6AP1,ATP6V1A,ATXN10,BCAS1,BCAT2,BIRC6,C17orf49,C1QBP,CAMSAP2,CAND1,CANX,CARMIL1,CAVIN1,CBX3,CC2D1B,CCDC47,CDC37L1,CDC42EP4,CDIPT,CDK1,CDK4,CDK6,CEP55,CNEP1R1,CNN2,CNN3,CRAT,CSNK1A1,CSRP1,CTBP2,CTTNBP2,CUL3,CWF19L1,CYB5R1,CYFIP1,DDB1,DDRGK1,DDX5,DGKZ,DGLUCY,DNAJC9,DNMT1,DNPEP,DOCK1,DOCK5,DPY19L1,DPYSL2,DPYSL3,DTYMK,DYNC2H1,EEF1B2,EEF2,EEF2K,EIF2B4,ELOA,ELP1,ELP2,ELP3,EMD,EML1,EXOC2,EXOC4,FADD,FADS1,FASN,FBXL15,FDFT1,FLII,FLNA,FLNB,FRMD4A,GABPA,GATM,GCDH,GCLC,GCLM,GDA,GFPT1,GHDC,GLRX3,GLRX5,GMDS,GMPPB,GOT2,GPT2,GSDMD,GTF2B,GTSE1,HACD3,HDGFL3,HM13,HMGCL,HMGCS1,HMMR,HMOX1,HMOX2,HNRNPUL1,HSPA4L,IDH2,IFT74,INPPL1,IRGQ,ITPR1,ITPR3,ITPRID2,KANK1,KANK2,KATNB1,KPNA2,LAMTOR3,LARP7,LETM1,LIMD1,LOXL3,LPCAT3,LPP,LRRC40,LRRC59,MARK2,MAT2A,MEGF10,MGST2,MKLN1,MMUT,MON2,MPI,MPST,MTDH,MTSS1,NAA25,NAB2,NAPA,NAXE,NCLN,NDC80,NDUFAF7,NEK9,NFKB2,NFS1,NIT2,NLE1,NLN,NPTXR,NQO1,NRBP1,NRDC,NUP54,OAT,OGT,OSBPL8,OXCT1,PAFAH1B1,PAK1,PAPSS1,PARG,PCBP2,PCK2,PDE4DIP,PDZRN3,PEPD,PFKM,PGD,PHLDA1,PHYH,PIGU,PITRM1,PKN2,PLCB1,PMPCB,POLA1,POLD1,POLD3,POLDIP3,PPA2,PPAT,PPOX,PPP2R5D,PPP4R3A,PRDX2,PRKACB,PRKCA,PRPF19,PRRC2A,PTPN1,QSOX2,RAB32,RAB6A,RABGGTA,RAD51,RAF1,RAI14,RANGAP1,RASSF4,RBPJ,RCN2,RETSAT,RIOK3,RNASEH2B,RPA1,RPA2,RPL13,RPL3,RPL6,RPL7A,RPN1,RPN2,RRM1,RRM2,SAMD4B,SEC14L1,SEC24A,SEC24D,SERPINE2,SFR1,SGTA,SH3GLB1,SLC1A4,SLC9A3R1,SLC9A3R2,SNF8,SNX1,SNX17,SOAT1,SORBS3,SPP1,SQLE,SQSTM1,SRGAP3,SRXN1,STAM2,STAT1,STAT3,STAT5B,STRN4,STT3A,SUCLG2,TAGLN2,TAX1BP1,TCF12,TKFC,TLE3,TLN1,TM9SF3,TM9SF4,TMEM115,TMEM43,TNS3,TOM1L2,TPD52,TRAPPC4,TRIM2,TRIM28,TRIM3,TRIM47,TRIOBP,TRIP10,TRIP12,TRIP13,TRMT2A,TST,TTC21B,UBAP2L,UBE2C,UBE2O,UBR7,UCKL1,USP24,USP47,USP8,USP9X,VAMP7,VAT1,VCP,VPS11,VPS13A,VWA5A,WDR12,WDR45B,WDR62,WFS1,WIPF1,XRN1,ZMPSTE24	320
Cancer,Hematological Disease,Immunological Disease,Organismal Injury and Abnormalities	Lymphoma	0,0000216		0	ALDH2,ALDH9A1,ANXA4,ARHGEF1,ATIC,BIRC6,CARMIL1,CDK1,CDK4,CDK6,CSNK1A1,CTBP2,CTTNBP2,DDB1,DGKZ,DNMT1,DOCK1,ECI2,EXOC4,FADD,FASN,FDFT1,GATM,GMPPB,GOT2,GTF2B,HMMR,HMOX1,IDH2,IKBIP,ITPR1,ITPR3,KANK2,KPNA2,LPP,NAPA,NARS1,NDC80,NFKB2,NQO1,PAK1,PDZRN3,POLA1,POLD1,PPAT,PPOX,PRKCA,PTPN1,RAD51,RAF1,RASSF4,RBPJ,RPA1,RPL13,RPL3,RPL5,RRM1,RRM2,SEC14L1,SNX1,STAT1,STAT3,STAT5B,TRIP12,TRIP13,TTC21B,UBE2C,USP8,VCP,WIPF1	70
DNA Replication, Recombination, and Repair	DNA damage response of cervical cancer cell lines	0,0000443		0	CDK1,DDB1,POLD1,RPA1	4

Categories	Diseases or Functions Annotation	p-value	Predicted Activation State	Activation z-score	Molecules	# Molecules
Cancer,Hematological Disease,Immunological Disease,Organismal Injury and Abnormalities	Tumorigenesis of lymphocytes	0,000261		0	AGL,ALDH2,ALDH9A1,ANXA4,ARHGEF1,ATIC,BIRC6,CARMIL1,CDC37L1,CDK1,CDK4,CDK6,CSNK1A1,CTBP2,CTTNBP2,DDB1,DGKZ,DNMT1,DOCK1,DOCK5,ECI2,ERLIN2,EXOC4,FADD,FASN,FDFT1,FLNA,GATM,GMPPB,GOT2,GTF2B,HMMR,HMOX1,IDH2,IKBIP,ITPR1,ITPR3,KANK2,KPNA2,LPP,MEGF10,NAPA,NARS1,NDC80,NFKB2,NPTXR,NQO1,PAK1,PDE4DIP,PDZRN3,PIGU,POLA1,POLD1,PPAT,PPOX,PRKCA,PRPF19,PTPN1,RAD51,RAF1,RASSF4,RBPJ,RPA1,RPL13,RPL3,RPL5,RPL7A,RRM1,RRM2,SEC14L1,SEC24D,SNX1,SPP1,SRGAP3,STAT1,STAT3,STAT5B,TRIM3,TRIOBP,TRIP12,TRIP13,TTC21B,UBE2C,UCKL1,USP47,USP8,USP9X,VCP,WIPF1	89
Cancer,Hematological Disease,Organismal Injury and Abnormalities	Hematologic cancer of cells	0,0004		0	AGL,ALDH2,ALDH9A1,ANXA4,ARHGEF1,ATIC,BIRC6,CARMIL1,CDC37L1,CDK1,CDK4,CDK6,CSNK1A1,CTBP2,CTTNBP2,DDB1,DGKZ,DNMT1,DOCK1,DOCK5,ECI2,ERLIN2,EXOC4,FADD,FASN,FDFT1,GATM,GMPPB,GOT2,GTF2B,HMMR,HMOX1,IDH2,IKBIP,ITPR1,ITPR3,KANK2,KPNA2,LPP,MEGF10,NAPA,NARS1,NDC80,NFKB2,NPTXR,NQO1,PAK1,PDZRN3,PIGU,POLA1,POLD1,PPAT,PPOX,PRKCA,PTPN1,RAD51,RAF1,RASSF4,RBPJ,RPA1,RPL13,RPL3,RPL5,RPL7A,RRM1,RRM2,SEC14L1,SEC24D,SNX1,SRGAP3,STAT1,STAT3,STAT5B,TAX1BP1,TRIM3,TRIOBP,TRIP12,TRIP13,TTC21B,UBE2C,UCKL1,USP47,USP8,USP9X,VCP,WIPF1	86
Cancer,Organismal Injury and Abnormalities	Carcinoma	1,25E-19		0,005	AARSD1,ABCD4,ACAA2,ACADS,ACO1,ACO2,ACP2,ACSF2,ACSL3,ACSL4,ADD1,ADI1,ADO,AGA,AGFG1,AGL,AIP,AK3,AKR1B1,AKR1B10,AKR7A2,ALDH2,ALDH4A1,ALDH6A1,ALDH9A1,ANAPC7,ANKRD13A,ANXA4,AP3S2,APPL2,ARFGAP2,ARFGAP3,ARFGEF2,ARHGEF1,ARHGEF12,ARHGEF18,ARMC8,ASRGL1,ATIC,ATP2A2,ATP2B4,ATP6AP1,ATP6V1A,ATPAF2,ATXN10,BCAS1,BCAT2,BIRC6,C17orf49,C1QBP,CACYBP,CAMSAP2,CAND1,CANX,CARMIL1,CAVIN1,CBX3,CC2D1B,CCDC47,CDC37L1,CDC42EP4,CDIPT,CDK1,CDK4,CDK6,CEP55,CERT1,CNEP1R1,CNN2,CNN3,CNPY2,CRAT,CSNK1A1,CSRP1,CTBP2,CTTNBP2,CUL3,CWF19L1,CYB5R1,CYFIP1,DCTN6,DDB1,DDRGK1,DDX5,DGKZ,DGLUCY,DNAJC5,DNAJC9,DNMT1,DNPEP,DOCK1,DOCK5,DPY19L1,DPYSL2,DPYSL3,DTYMK,DYNC2H1,ECI2,EEF1B2,EEF2,EEF2K,EIF2B4,EIF2B5,ELOA,ELP1,ELP2,ELP3,EMC7,EMD,EML1,ERLIN2,EXOC2,EXOC4,EXTL3,FADD,FADS1,FAF2,FASN,FBXL15,FBXO7,FDFT1,FLII,FLNA,FLNB,FRMD4A,FUT8,GABPA,GATM,GCDH,GCLC,GCLM,GDA,GFPT1,GHDC,GLRX3,GLRX5,GMDS,GMPPB,GOT2,GPT2,GRB14,GSDMD,GSTZ1,GTF2B,GTSE1,HACD3,HAUS7,HDGFL3,HEBP2,HIKESHI,HM13,HMGCL,HMGCS1,HMMR,HMOX1,HMOX2,HNRNPUL1,HSPA4L,IDH2,IFT74,IKBIP,IMPA1,INPP1,INPPL1,IRGQ,ISCA2,ITPR1,ITPR3,ITPRID2,KANK1,KANK2,KATNB1,KPNA2,LAMTOR2,LAMTOR3,LARP7,LAYN,LETM1,LIMD1,LOXL3,LPCAT3,LPP,LRRC40,LRRC59,LTA4H,MARK2,MAT2A,MEGF10,MGST2,MKLN1,MMUT,MON2,MPI,MPST,MTDH,MTSS1,NAA25,NAB2,NAPA,NAXE,NCLN,NDC80,NDUFAF7,NEK9,NFKB2,NFS1,NIT2,NLE1,NLN,NMD3,NPTXR,NQO1,NRBP1,NRDC,NT5C3B,NUP54,OAT,OGT,OSBPL8,OXCT1,PAFAH1B1,PAK1,PAPSS1,PARG,PCBP2,PCK2,PDE4DIP,PDZRN3,PEPD,PFKM,PGD,PHLDA1,PHYH,PIGU,PIP4P2,PITRM1,PKN2,PLCB1,PMPCB,POLA1,POLD1,POLD3,POLDIP3,PPA2,PPAT,PPOX,PPP2R5D,PPP4R3A,PRDX2,PRKACB,PRKCA,PRPF19,PRRC2A,PRRX1,PTPN1,QSOX2,RAB32,RAB6A,RABGGTA,RAD51,RAF1,RAI14,RANGAP1,RAP2C,RASSF4,RBPJ,RCN2,RER1,RETSAT,RIOK3,RNASEH2B,RPA1,RPA2,RPL13,RPL27,RPL3,RPL5,RPL6,RPL7A,RPN1,RPN2,RRM1,RRM2,SAMD4B,SDF4,SEC14L1,SEC24A,SEC24D,SEC61A1,SERPINE2,SFR1,SGTA,SH3GLB1,SLC1A4,SLC9A3R1,SLC9A3R2,SNF8,SNX1,SNX17,SOAT1,SORBS3,SPP1,SQLE,SQSTM1,SRGAP3,SRXN1,STAM2,STARD3NL,STAT1,STAT3,STAT5B,STRN4,STT3A,SUCLG2,TAGLN2,TAX1BP1,TCF12,TDRKH,TKFC,TLE3,TLN1,TM9SF3,TM9SF4,TMEM115,TMEM43,TNS3,TOM1L2,TPD52,TRAPPC4,TRIM2,TRIM28,TRIM3,TRIM47,TRIOBP,TRIP10,TRIP12,TRIP13,TRMT2A,TST,TTC21B,UBAP2L,UBE2C,UBE2O,UBR7,UCKL1,USP24,USP47,USP8,USP9X,VAMP7,VAT1,VCP,VCPIP1,VPS11,VPS13A,VPS36,VPS4B,VWA5A,WDR12,WDR45B,WDR62,WFS1,WIPF1,XRN1,ZMPSTE24	365
Cell Death and Survival	Cell death of cervical cancer cell lines	1,06E-07		0,024	ATP2A2,CDK1,CYB5R1,EXOC2,FADD,FLNB,GTSE1,ITPR1,LETM1,NAPA,NDC80,PAK1,PCBP2,PRDX2,PRKCA,PRPF19,RAB32,RAF1,RNASEH2B,RPA1,SDF4,SPP1,SQSTM1,STAT1,STAT3,TNFAIP8,UBE2I,VCP	28
Protein Degradation,Protein Synthesis	Catabolism of protein	0,00005		0,036	ADI1,ANAPC7,CANX,CCDC47,CUL3,DDB1,DDRGK1,FADD,FBXO7,FLNA,GTSE1,NCLN,NDC80,NLN,NRDC,OGT,PITRM1,RPL5,SERPINE2,SH3GLB1,SNF8,SNX1,SPCS3,SQSTM1,TRIP12,UBE2C,UBE2I,USP8,USP9X,VCP,VPS11,WFS1,ZMPSTE24	33
Cell Death and Survival	Apoptosis of colorectal cancer cell lines	6,32E-08		0,048	AKR1B10,CBX3,CSNK1A1,CTBP2,DTYMK,EIF2B5,EXOC2,FADD,FASN,GMDS,ITPR3,MTSS1,NAPA,POLA1,RAF1,RPA2,SLC9A3R1,SNX1,SPP1,SQSTM1,STAT1,TM9SF4,VCP	23
Cell Death and Survival	Cell death of colorectal cancer cell lines	4,15E-08		0,165	AKR1B10,CBX3,CDK1,CSNK1A1,CTBP2,DTYMK,EIF2B5,EXOC2,FADD,FASN,GMDS,HMOX1,ITPR3,MTSS1,NAPA,POLA1,RAF1,RPA2,SLC9A3R1,SNX1,SPP1,SQSTM1,STAT1,TM9SF4,UBE2C,VCP	26
DNA Replication, Recombination, and Repair	DNA damage response of cells	0,000329		0,2	CBX3,CDK1,DDB1,PAK1,PARG,POLD1,RAD51,RPA1,RPA2,TRIP12,USP47,USP9X,VCP,VCPIP1,ZMPSTE24	15
Cellular Response to Therapeutics	Drug resistance of breast cancer cell lines	0,0000897		0,218	CUL3,ELOA,LAMTOR2,MARK2,PAPSS1,STAT5B,VCP	7
Cancer,Cellular Response to Therapeutics,Organismal Injury and Abnormalities,Reproductive System Disease	Chemotherapy resistance of breast cancer cell lines	0,000295		0,218	CUL3,ELOA,LAMTOR2,MARK2,PAPSS1,VCP	6
Cellular Response to Therapeutics	Drug resistance of tumor cell lines	0,00000938		0,31	BIRC6,CUL3,ELOA,HMMR,LAMTOR2,MARK2,PAPSS1,RAF1,STAT3,STAT5B,TRIM28,TRIP12,VCP,VPS11	14
Gastrointestinal Disease,Hepatic System Disease,Metabolic Disease,Organismal Injury and Abnormalities	Hepatic steatosis	0,000131		0,443	ACAA2,ACACA,ACADS,ACSL4,ATP2A2,CNPY2,FASN,FDFT1,GRB14,GSTZ1,HMOX1,ITPR1,LPCAT3,LTA4H,MMUT,PAFAH1B1,PHYH,PPP4R3A,RBPJ,SOAT1,SPP1,STAT3	22
Cell Death and Survival	Cell death of adenocarcinoma cell lines	0,000284		0,49	ADI1,FADS1,MTDH,NQO1,SLC9A3R1,SQSTM1,UBE2C	7
Cancer,Organismal Injury and Abnormalities	Solid tumor	2,44E-20		0,526	AARSD1,ABCD4,ACAA2,ACACA,ACADS,ACO1,ACO2,ACP2,ACSF2,ACSL3,ACSL4,ADD1,ADI1,ADO,AGA,AGFG1,AGL,AIP,AK3,AKR1B1,AKR1B10,AKR7A2,ALDH2,ALDH4A1,ALDH6A1,ALDH9A1,ANAPC7,ANKRD13A,ANKRD46,ANXA4,AP3S2,APPL2,ARFGAP2,ARFGAP3,ARFGEF2,ARHGEF1,ARHGEF12,ARHGEF18,ARMC8,ARPIN/ARPIN-AP3S2,ASRGL1,ATIC,ATP2A2,ATP2B4,ATP6AP1,ATP6V1A,ATPAF2,ATXN10,BCAS1,BCAT2,BIRC6,C17orf49,C1QBP,CACYBP,CAMSAP2,CAND1,CANX,CARMIL1,CAVIN1,CBX3,CC2D1B,CCDC47,CCZ1/CCZ1B,CDC37L1,CDC42EP4,CDIPT,CDK1,CDK4,CDK6,CEP55,CERT1,CNEP1R1,CNN2,CNN3,CNPY2,CRAT,CSNK1A1,CSRP1,CTBP2,CTTNBP2,CUL3,CWF19L1,CYB5R1,Cyb5r3,CYFIP1,DCTN6,DDB1,DDRGK1,DDX5,DGKZ,DGLUCY,DNAJC5,DNAJC9,DNMT1,DNPEP,DOCK1,DOCK5,DPY19L1,DPYSL2,DPYSL3,DTYMK,DYNC2H1,ECI2,EEF1B2,EEF2,EEF2K,EIF2B4,EIF2B5,ELOA,ELP1,ELP2,ELP3,EMC7,EMD,EML1,ERLIN2,EXOC2,EXOC4,EXTL3,FADD,FADS1,FAF2,FASN,FBXL15,FBXO7,FDFT1,FLII,FLNA,FLNB,FRMD4A,FUT8,GABPA,GATM,GCDH,GCLC,GCLM,GDA,GFPT1,GHDC,GLRX,GLRX3,GLRX5,GMDS,GMPPB,GOT2,GPT2,GRB14,GSDMD,GSTZ1,GTF2B,GTSE1,HACD3,HAUS7,HDGFL3,HEBP2,HIKESHI,HM13,HMGCL,HMGCS1,HMMR,HMOX1,HMOX2,HNRNPUL1,HSPA4L,IDH2,IFT74,IKBIP,IMPA1,INPP1,INPPL1,IRGQ,ISCA2,ITPR1,ITPR3,ITPRID2,KANK1,KANK2,KATNB1,KPNA2,LAMTOR2,LAMTOR3,LARP7,LAYN,LETM1,LIMD1,LOXL3,LPCAT3,LPP,LRRC40,LRRC59,LTA4H,MARK2,MAT2A,MEGF10,MGST2,MKLN1,MMUT,MON2,MPI,MPST,MTDH,MTSS1,NAA25,NAB2,NAPA,NARS1,NAXE,NCLN,NDC80,NDUFAF7,NEK9,NFKB2,NFS1,NIT2,NLE1,NLN,NMD3,NPTXR,NQO1,NRBP1,NRDC,NT5C3B,NUP54,OAT,OGT,OSBPL8,OXCT1,PAFAH1B1,PAK1,PAPSS1,PARG,PCBP2,PCK2,PDE4DIP,PDZRN3,PEPD,PFKM,PGD,PHLDA1,PHYH,PIGU,PIP4P2,PITRM1,PKN2,PLCB1,PMPCB,POLA1,POLD1,POLD3,POLDIP3,PPA2,PPAT,PPOX,PPP2R5D,PPP4R3A,PRDX2,PRKACB,PRKCA,PRPF19,PRRC2A,PRRX1,PTPN1,QSOX2,RAB32,RAB6A,RABGGTA,RAD51,RAF1,RAI14,RANGAP1,RAP2C,RASSF4,RBPJ,RCN2,RER1,RETSAT,RIOK3,RNASEH2B,RPA1,RPA2,RPL13,RPL27,RPL3,RPL5,RPL6,RPL7A,RPN1,RPN2,RRM1,RRM2,SAMD4B,SDF4,SEC14L1,SEC24A,SEC24D,SEC61A1,SERPINE2,SFR1,SGTA,SH3GLB1,SLC1A4,SLC9A3R1,SLC9A3R2,SNF8,SNX1,SNX17,SOAT1,SORBS3,SPCS3,SPP1,SQLE,SQSTM1,SRGAP3,SRXN1,STAM2,STARD3NL,STAT1,STAT3,STAT5B,STRN4,STT3A,SUCLG2,TAGLN2,TAX1BP1,TCF12,TDRKH,TKFC,TLE3,TLN1,TM9SF3,TM9SF4,TMEM115,TMEM43,TNS3,TOM1L2,TPD52,TRAPPC4,TRIM2,TRIM28,TRIM3,TRIM47,TRIOBP,TRIP10,TRIP12,TRIP13,TRMT2A,TST,TTC21B,UBAP2L,UBE2C,UBE2I,UBE2O,UBR7,UCKL1,USP24,USP47,USP8,USP9X,VAMP7,VAT1,VCP,VCPIP1,VPS11,VPS13A,VPS36,VPS4B,VWA5A,WDR12,WDR45B,WDR62,WFS1,WIPF1,XRN1,ZMPSTE24	374
Cancer,Gastrointestinal Disease,Organismal Injury and Abnormalities	Development of digestive organ tumor	0,0000226		0,557	AARSD1,ACO1,ACO2,ACSF2,ACSL3,ACSL4,ADD1,AGL,AK3,AKR1B1,AKR1B10,AKR7A2,ALDH2,ALDH6A1,ANKRD13A,ANXA4,APPL2,ARFGAP2,ARFGAP3,ARHGEF1,ARHGEF18,ARMC8,ATIC,ATP2A2,ATXN10,BCAT2,BIRC6,C1QBP,CACYBP,CAMSAP2,CANX,CAVIN1,CBX3,CDK4,CDK6,CEP55,CNPY2,CRAT,CSNK1A1,CTBP2,CTTNBP2,CYB5R1,CYFIP1,DCTN6,DDB1,DDX5,DGKZ,DNAJC5,DNMT1,DOCK1,DOCK5,DPY19L1,DPYSL3,DYNC2H1,EEF1B2,EEF2,EEF2K,EIF2B4,ELOA,ELP1,EMC7,EML1,ERLIN2,EXOC2,EXOC4,EXTL3,FAF2,FASN,FLII,FLNA,FLNB,FRMD4A,GCLM,GHDC,GMPPB,GRB14,GSTZ1,GTF2B,GTSE1,HDGFL3,HEBP2,HM13,HMMR,HNRNPUL1,HSPA4L,IDH2,IMPA1,INPPL1,IRGQ,ISCA2,ITPR1,ITPR3,ITPRID2,KANK1,KPNA2,LARP7,LAYN,LIMD1,LOXL3,LPP,MARK2,MEGF10,MON2,MTDH,MTSS1,NAA25,NAB2,NCLN,NEK9,NLE1,NQO1,NRBP1,NRDC,NT5C3B,NUP54,OXCT1,PAK1,PAPSS1,PARG,PCBP2,PCK2,PDE4DIP,PDZRN3,PEPD,PHLDA1,PIGU,PKN2,PLCB1,POLA1,POLD1,POLD3,POLDIP3,PPP2R5D,PPP4R3A,PRKACB,PRKCA,PRRC2A,PRRX1,PTPN1,RAD51,RAF1,RBPJ,RETSAT,RIOK3,RNASEH2B,RPA1,RPL5,RRM1,SAMD4B,SDF4,SEC14L1,SEC24A,SEC24D,SERPINE2,SLC1A4,SLC9A3R2,SNX1,SNX17,SOAT1,SORBS3,SQLE,SQSTM1,SRGAP3,STAT1,STAT3,STAT5B,STT3A,SUCLG2,TAX1BP1,TCF12,TDRKH,TLN1,TM9SF3,TM9SF4,TMEM115,TMEM43,TNS3,TPD52,TRIM28,TRIM3,TRIM47,TRIOBP,TRIP10,TRIP12,TRIP13,UBAP2L,UBE2C,UCKL1,USP24,USP47,USP9X,VCP,VCPIP1,VPS11,VPS13A,VPS36,WDR12,WDR45B,WDR62,WFS1,WIPF1,XRN1,ZMPSTE24	203
Cell Death and Survival	Apoptosis of carcinoma cell lines	0,0000215		0,581	ACO2,ADI1,AKR1B10,DNMT1,FADD,FASN,HMOX1,MTDH,OGT,PARG,PCK2,POLA1,PRKCA,PTPN1,RABGGTA,RPA2,RRM1,RRM2,SLC9A3R1,SPP1,SQSTM1,STAT3	22
Cancer,Gastrointestinal Disease,Organismal Injury and Abnormalities	Digestive system cancer	1,28E-13		0,67	AARSD1,ABCD4,ACAA2,ACADS,ACO1,ACO2,ACP2,ACSF2,ACSL3,ACSL4,ADD1,AGA,AGFG1,AGL,AIP,AK3,AKR1B1,AKR1B10,AKR7A2,ALDH2,ALDH4A1,ALDH6A1,ALDH9A1,ANKRD13A,ANXA4,APPL2,ARFGAP2,ARFGAP3,ARFGEF2,ARHGEF1,ARHGEF12,ARHGEF18,ARMC8,ASRGL1,ATIC,ATP2A2,ATP2B4,ATP6AP1,ATP6V1A,ATXN10,BCAS1,BCAT2,BIRC6,C1QBP,CACYBP,CAMSAP2,CAND1,CANX,CARMIL1,CAVIN1,CBX3,CC2D1B,CCDC47,CDC37L1,CDC42EP4,CDIPT,CDK1,CDK4,CDK6,CEP55,CNPY2,CRAT,CSNK1A1,CTBP2,CTTNBP2,CWF19L1,CYB5R1,CYFIP1,DCTN6,DDB1,DDX5,DGKZ,DGLUCY,DNAJC5,DNAJC9,DNMT1,DNPEP,DOCK1,DOCK5,DPY19L1,DPYSL2,DPYSL3,DYNC2H1,ECI2,EEF1B2,EEF2,EEF2K,EIF2B4,EIF2B5,ELOA,ELP1,ELP2,ELP3,EMC7,EMD,EML1,ERLIN2,EXOC2,EXOC4,EXTL3,FADS1,FAF2,FASN,FBXL15,FBXO7,FDFT1,FLII,FLNA,FLNB,FRMD4A,FUT8,GCDH,GCLC,GCLM,GDA,GFPT1,GHDC,GLRX,GLRX3,GLRX5,GMDS,GMPPB,GOT2,GPT2,GRB14,GSDMD,GSTZ1,GTF2B,GTSE1,HACD3,HAUS7,HDGFL3,HEBP2,HM13,HMGCL,HMGCS1,HMMR,HMOX1,HMOX2,HNRNPUL1,HSPA4L,IDH2,IFT74,IKBIP,IMPA1,INPP1,INPPL1,IRGQ,ISCA2,ITPR1,ITPR3,ITPRID2,KANK1,KANK2,KPNA2,LAMTOR2,LARP7,LAYN,LETM1,LIMD1,LOXL3,LPCAT3,LPP,LRRC59,LTA4H,MARK2,MAT2A,MEGF10,MKLN1,MMUT,MON2,MPI,MPST,MTDH,MTSS1,NAA25,NAB2,NAPA,NCLN,NDUFAF7,NEK9,NFKB2,NLE1,NLN,NPTXR,NQO1,NRBP1,NRDC,NT5C3B,NUP54,OAT,OGT,OSBPL8,OXCT1,PAFAH1B1,PAK1,PAPSS1,PARG,PCBP2,PCK2,PDE4DIP,PDZRN3,PEPD,PFKM,PHLDA1,PHYH,PIGU,PIP4P2,PITRM1,PKN2,PLCB1,PMPCB,POLA1,POLD1,POLD3,POLDIP3,PPA2,PPOX,PPP2R5D,PPP4R3A,PRKACB,PRKCA,PRRC2A,PRRX1,PTPN1,QSOX2,RAB32,RABGGTA,RAD51,RAF1,RAI14,RANGAP1,RAP2C,RASSF4,RBPJ,RCN2,RER1,RETSAT,RIOK3,RNASEH2B,RPA1,RPA2,RPL27,RPL3,RPL5,RPL6,RPL7A,RPN1,RPN2,RRM1,RRM2,SAMD4B,SDF4,SEC14L1,SEC24A,SEC24D,SEC61A1,SERPINE2,SH3GLB1,SLC1A4,SLC9A3R1,SLC9A3R2,SNF8,SNX1,SNX17,SOAT1,SORBS3,SPP1,SQLE,SQSTM1,SRGAP3,SRXN1,STAM2,STAT1,STAT3,STAT5B,STRN4,STT3A,SUCLG2,TAGLN2,TAX1BP1,TCF12,TDRKH,TKFC,TLE3,TLN1,TM9SF3,TM9SF4,TMEM115,TMEM43,TNS3,TOM1L2,TPD52,TRAPPC4,TRIM2,TRIM28,TRIM3,TRIM47,TRIOBP,TRIP10,TRIP12,TRIP13,TRMT2A,TST,TTC21B,UBAP2L,UBE2C,UBE2O,UBR7,UCKL1,USP24,USP47,USP8,USP9X,VAT1,VCP,VCPIP1,VPS11,VPS13A,VPS36,VPS4B,VWA5A,WDR12,WDR45B,WDR62,WFS1,WIPF1,XRN1,ZMPSTE24	329
Cancer,Gastrointestinal Disease,Organismal Injury and Abnormalities	Digestive organ tumor	3,14E-14		0,683	AARSD1,ABCD4,ACAA2,ACADS,ACO1,ACO2,ACP2,ACSF2,ACSL3,ACSL4,ADD1,AGA,AGFG1,AGL,AIP,AK3,AKR1B1,AKR1B10,AKR7A2,ALDH2,ALDH4A1,ALDH6A1,ALDH9A1,ANKRD13A,ANKRD46,ANXA4,APPL2,ARFGAP2,ARFGAP3,ARFGEF2,ARHGEF1,ARHGEF12,ARHGEF18,ARMC8,ASRGL1,ATIC,ATP2A2,ATP2B4,ATP6AP1,ATP6V1A,ATXN10,BCAS1,BCAT2,BIRC6,C1QBP,CACYBP,CAMSAP2,CAND1,CANX,CARMIL1,CAVIN1,CBX3,CC2D1B,CCDC47,CDC37L1,CDC42EP4,CDIPT,CDK1,CDK4,CDK6,CEP55,CNPY2,CRAT,CSNK1A1,CTBP2,CTTNBP2,CWF19L1,CYB5R1,CYFIP1,DCTN6,DDB1,DDX5,DGKZ,DGLUCY,DNAJC5,DNAJC9,DNMT1,DNPEP,DOCK1,DOCK5,DPY19L1,DPYSL2,DPYSL3,DYNC2H1,ECI2,EEF1B2,EEF2,EEF2K,EIF2B4,EIF2B5,ELOA,ELP1,ELP2,ELP3,EMC7,EMD,EML1,ERLIN2,EXOC2,EXOC4,EXTL3,FADS1,FAF2,FASN,FBXL15,FBXO7,FDFT1,FLII,FLNA,FLNB,FRMD4A,FUT8,GCDH,GCLC,GCLM,GDA,GFPT1,GHDC,GLRX,GLRX3,GLRX5,GMDS,GMPPB,GOT2,GPT2,GRB14,GSDMD,GSTZ1,GTF2B,GTSE1,HACD3,HAUS7,HDGFL3,HEBP2,HIKESHI,HM13,HMGCL,HMGCS1,HMMR,HMOX1,HMOX2,HNRNPUL1,HSPA4L,IDH2,IFT74,IKBIP,IMPA1,INPP1,INPPL1,IRGQ,ISCA2,ITPR1,ITPR3,ITPRID2,KANK1,KANK2,KATNB1,KPNA2,LAMTOR2,LARP7,LAYN,LETM1,LIMD1,LOXL3,LPCAT3,LPP,LRRC59,LTA4H,MARK2,MAT2A,MEGF10,MKLN1,MMUT,MON2,MPI,MPST,MTDH,MTSS1,NAA25,NAB2,NAPA,NCLN,NDUFAF7,NEK9,NFKB2,NLE1,NLN,NPTXR,NQO1,NRBP1,NRDC,NT5C3B,NUP54,OAT,OGT,OSBPL8,OXCT1,PAFAH1B1,PAK1,PAPSS1,PARG,PCBP2,PCK2,PDE4DIP,PDZRN3,PEPD,PFKM,PHLDA1,PHYH,PIGU,PIP4P2,PITRM1,PKN2,PLCB1,PMPCB,POLA1,POLD1,POLD3,POLDIP3,PPA2,PPOX,PPP2R5D,PPP4R3A,PRDX2,PRKACB,PRKCA,PRRC2A,PRRX1,PTPN1,QSOX2,RAB32,RAB6A,RABGGTA,RAD51,RAF1,RAI14,RANGAP1,RAP2C,RASSF4,RBPJ,RCN2,RER1,RETSAT,RIOK3,RNASEH2B,RPA1,RPA2,RPL27,RPL3,RPL5,RPL6,RPL7A,RPN1,RPN2,RRM1,RRM2,SAMD4B,SDF4,SEC14L1,SEC24A,SEC24D,SEC61A1,SERPINE2,SH3GLB1,SLC1A4,SLC9A3R1,SLC9A3R2,SNF8,SNX1,SNX17,SOAT1,SORBS3,SPP1,SQLE,SQSTM1,SRGAP3,SRXN1,STAM2,STAT1,STAT3,STAT5B,STRN4,STT3A,SUCLG2,TAGLN2,TAX1BP1,TCF12,TDRKH,TKFC,TLE3,TLN1,TM9SF3,TM9SF4,TMEM115,TMEM43,TNS3,TOM1L2,TPD52,TRAPPC4,TRIM2,TRIM28,TRIM3,TRIM47,TRIOBP,TRIP10,TRIP12,TRIP13,TRMT2A,TST,TTC21B,UBAP2L,UBE2C,UBE2O,UBR7,UCKL1,USP24,USP47,USP8,USP9X,VAT1,VCP,VCPIP1,VPS11,VPS13A,VPS36,VPS4B,VWA5A,WDR12,WDR45B,WDR62,WFS1,WIPF1,XRN1,ZMPSTE24	334
Cell-To-Cell Signaling and Interaction	Interaction of tumor cell lines	0,000174		0,8	ADD1,ARHGEF1,C1QBP,EXTL3,FASN,FLNA,GLRX,HMMR,INPPL1,MON2,NRDC,PAK1,PHLDA1,PRKCA,RAF1,SNX1,SNX17,SPP1,STAT1,STAT3,TCF12,TLN1,UBE2I,VAMP7	24

Categories	Diseases or Functions Annotation	p-value	Predicted Activation State	Activation z-score	Molecules	# Molecules
Cancer,Organismal Injury and Abnormalities	Malignant solid tumor	2,32E-20		0,889	AARSD1,ABCD4,ACAA2,ACADS,ACO1,ACO2,ACP2,ACSF2,ACSL3,ACSL4,ADD1,ADI1,ADO,AGA,AGFG1,AGL,AIP,AK3,AKR1B1,AKR1B10,AKR7A2,ALDH2,ALDH4A1,ALDH6A1,ALDH9A1,ANAPC7,ANKRD13A,ANKRD46,ANXA4,AP3S2,APPL2,ARFGAP2,ARFGAP3,ARFGEF2,ARHGEF1,ARHGEF12,ARHGEF18,ARMC8,ARPIN/ARPIN-AP3S2,ASRGL1,ATIC,ATP2A2,ATP2B4,ATP6AP1,ATP6V1A,ATPAF2,ATXN10,BCAS1,BCAT2,BIRC6,C17orf49,C1QBP,CACYBP,CAMSAP2,CAND1,CANX,CARMIL1,CAVIN1,CBX3,CC2D1B,CCDC47,CCZ1/CCZ1B,CDC37L1,CDC42EP4,CDIPT,CDK1,CDK4,CDK6,CEP55,CERT1,CNEP1R1,CNN2,CNN3,CNPY2,CRAT,CSNK1A1,CSRP1,CTBP2,CTTNBP2,CUL3,CWF19L1,CYB5R1,Cyb5r3,CYFIP1,DCTN6,DDB1,DDRGK1,DDX5,DGKZ,DGLUCY,DNAJC5,DNAJC9,DNMT1,DNPEP,DOCK1,DOCK5,DPY19L1,DPYSL2,DPYSL3,DTYMK,DYNC2H1,ECI2,EEF1B2,EEF2,EEF2K,EIF2B4,EIF2B5,ELOA,ELP1,ELP2,ELP3,EMC7,EMD,EML1,ERLIN2,EXOC2,EXOC4,EXTL3,FADD,FADS1,FAF2,FASN,FBXL15,FBXO7,FDFT1,FLII,FLNA,FLNB,FRMD4A,FUT8,GABPA,GATM,GCDH,GCLC,GCLM,GDA,GFPT1,GHDC,GLRX,GLRX3,GLRX5,GMDS,GMPPB,GOT2,GPT2,GRB14,GSDMD,GSTZ1,GTF2B,GTSE1,HACD3,HAUS7,HDGFL3,HEBP2,HIKESHI,HM13,HMGCL,HMGCS1,HMMR,HMOX1,HMOX2,HNRNPUL1,HSPA4L,IDH2,IFT74,IKBIP,IMPA1,INPP1,INPPL1,IRGQ,ISCA2,ITPR1,ITPR3,ITPRID2,KANK1,KANK2,KATNB1,KPNA2,LAMTOR2,LAMTOR3,LARP7,LAYN,LETM1,LIMD1,LOXL3,LPCAT3,LPP,LRRC40,LRRC59,LTA4H,MARK2,MAT2A,MEGF10,MGST2,MKLN1,MMUT,MON2,MPI,MPST,MTDH,MTSS1,NAA25,NAB2,NAPA,NARS1,NAXE,NCLN,NDC80,NDUFAF7,NEK9,NFKB2,NFS1,NIT2,NLE1,NLN,NMD3,NPTXR,NQO1,NRBP1,NRDC,NT5C3B,NUP54,OAT,OGT,OSBPL8,OXCT1,PAFAH1B1,PAK1,PAPSS1,PARG,PCBP2,PCK2,PDE4DIP,PDZRN3,PEPD,PFKM,PGD,PHLDA1,PHYH,PIGU,PIP4P2,PITRM1,PKN2,PLCB1,PMPCB,POLA1,POLD1,POLD3,POLDIP3,PPA2,PPAT,PPOX,PPP2R5D,PPP4R3A,PRDX2,PRKACB,PRKCA,PRPF19,PRRC2A,PRRX1,PTPN1,QSOX2,RAB32,RAB6A,RABGGTA,RAD51,RAF1,RAI14,RANGAP1,RAP2C,RASSF4,RBPJ,RCN2,RER1,RETSAT,RIOK3,RNASEH2B,RPA1,RPA2,RPL13,RPL27,RPL3,RPL5,RPL6,RPL7A,RPN1,RPN2,RRM1,RRM2,SAMD4B,SDF4,SEC14L1,SEC24A,SEC24D,SEC61A1,SERPINE2,SFR1,SGTA,SH3GLB1,SLC1A4,SLC9A3R1,SLC9A3R2,SNF8,SNX1,SNX17,SOAT1,SORBS3,SPCS3,SPP1,SQLE,SQSTM1,SRGAP3,SRXN1,STAM2,STARD3NL,STAT1,STAT3,STAT5B,STRN4,STT3A,SUCLG2,TAGLN2,TAX1BP1,TCF12,TDRKH,TKFC,TLE3,TLN1,TM9SF3,TM9SF4,TMEM115,TMEM43,TNS3,TOM1L2,TPD52,TRAPPC4,TRIM2,TRIM28,TRIM3,TRIM47,TRIOBP,TRIP10,TRIP12,TRIP13,TRMT2A,TST,TTC21B,UBAP2L,UBE2C,UBE2I,UBE2O,UBR7,UCKL1,USP24,USP47,USP8,USP9X,VAMP7,VAT1,VCP,VCPIP1,VPS11,VPS13A,VPS36,VPS4B,VWA5A,WDR12,WDR45B,WDR62,WFS1,WIPF1,XRN1,ZMPSTE24	373
Cell Death and Survival	Cell death of carcinoma cell lines	0,00000144		0,928	ACO2,ADI1,AKR1B10,DNMT1,FADD,FADS1,FASN,HMOX1,MTDH,NQO1,OGT,PARG,PCK2,POLA1,PRKCA,PTPN1,RABGGTA,RAF1,RPA2,RRM1,RRM2,SLC9A3R1,SPP1,SQSTM1,STAT1,STAT3,UBE2C	27
Cancer,Organismal Injury and Abnormalities	Malignant genitourinary solid tumor	4,24E-10		0,928	AARSD1,ABCD4,ACADS,ACO1,ACP2,ACSF2,ACSL3,ACSL4,ADD1,ADI1,AGL,AIP,AK3,AKR1B1,AKR7A2,ALDH2,ALDH4A1,ALDH9A1,ANKRD13A,AP3S2,APPL2,ARFGAP2,ARFGAP3,ARFGEF2,ARHGEF1,ARHGEF12,ARHGEF18,ASRGL1,ATIC,ATP2B4,ATP6AP1,ATP6V1A,ATPAF2,BCAS1,BIRC6,C17orf49,C1QBP,CAMSAP2,CAND1,CANX,CARMIL1,CAVIN1,CBX3,CC2D1B,CCDC47,CCZ1/CCZ1B,CDC42EP4,CDIPT,CDK1,CDK4,CDK6,CEP55,CERT1,CNN2,CNPY2,CRAT,CSNK1A1,CTBP2,CTTNBP2,CUL3,Cyb5r3,DDB1,DDRGK1,DDX5,DGKZ,DGLUCY,DNMT1,DOCK1,DOCK5,DPY19L1,DPYSL2,DTYMK,DYNC2H1,EEF1B2,EEF2,EEF2K,ELOA,ELP1,ELP2,ELP3,EMC7,EMD,EML1,ERLIN2,EXOC2,EXOC4,FADD,FAF2,FASN,FBXO7,FDFT1,FLII,FLNA,FLNB,FRMD4A,FUT8,GABPA,GATM,GCDH,GDA,GFPT1,GHDC,GLRX3,GLRX5,GMDS,GMPPB,GOT2,GPT2,GRB14,GSDMD,GTSE1,HAUS7,HDGFL3,HEBP2,HIKESHI,HMGCS1,HMMR,HMOX1,HNRNPUL1,HSPA4L,IDH2,IFT74,IKBIP,INPPL1,IRGQ,ITPR1,ITPR3,KANK1,KATNB1,KPNA2,LARP7,LAYN,LETM1,LIMD1,LOXL3,LPP,LRRC40,LTA4H,MARK2,MAT2A,MEGF10,MGST2,MKLN1,MMUT,MON2,MPST,MTDH,MTSS1,NAA25,NAB2,NAPA,NCLN,NDC80,NEK9,NFKB2,NLE1,NMD3,NPTXR,NQO1,NRDC,NUP54,OAT,OGT,OSBPL8,OXCT1,PAFAH1B1,PAK1,PAPSS1,PARG,PCBP2,PCK2,PDE4DIP,PDZRN3,PEPD,PFKM,PHLDA1,PHYH,PIGU,PIP4P2,PITRM1,PKN2,PLCB1,POLA1,POLD1,POLD3,POLDIP3,PPAT,PPOX,PRDX2,PRKACB,PRKCA,PRPF19,PRRC2A,PRRX1,PTPN1,RAB32,RAD51,RAF1,RAI14,RANGAP1,RAP2C,RCN2,RER1,RETSAT,RIOK3,RNASEH2B,RPA1,RPA2,RPL5,RPL6,RRM1,RRM2,SAMD4B,SDF4,SEC14L1,SEC24A,SEC24D,SEC61A1,SERPINE2,SGTA,SH3GLB1,SLC1A4,SLC9A3R1,SNF8,SNX1,SNX17,SOAT1,SORBS3,SPP1,SQLE,SQSTM1,STAT1,STAT3,STAT5B,STT3A,SUCLG2,TAGLN2,TAX1BP1,TCF12,TKFC,TLE3,TLN1,TMEM43,TNS3,TOM1L2,TPD52,TRAPPC4,TRIM28,TRIM3,TRIOBP,TRIP10,TRIP12,TRIP13,TRMT2A,TST,UBAP2L,UBE2C,UBE2O,UCKL1,USP24,USP47,USP8,USP9X,VCPIP1,VPS11,VPS13A,VPS36,VWA5A,WDR12,WDR62,WFS1,WIPF1,XRN1,ZMPSTE24	274
Cancer,Organismal Injury and Abnormalities	Abdominal carcinoma	3,55E-15		0,933	AARSD1,ABCD4,ACAA2,ACADS,ACO1,ACO2,ACP2,ACSF2,ACSL3,ACSL4,ADD1,AGA,AGFG1,AGL,AIP,AK3,AKR1B1,AKR1B10,AKR7A2,ALDH2,ALDH4A1,ALDH6A1,ALDH9A1,ANKRD13A,ANXA4,AP3S2,APPL2,ARFGAP2,ARFGAP3,ARFGEF2,ARHGEF1,ARHGEF12,ARHGEF18,ARMC8,ASRGL1,ATIC,ATP2A2,ATP2B4,ATP6AP1,ATP6V1A,ATPAF2,ATXN10,BCAS1,BCAT2,BIRC6,C1QBP,CACYBP,CAMSAP2,CAND1,CANX,CARMIL1,CAVIN1,CBX3,CC2D1B,CCDC47,CDC37L1,CDC42EP4,CDIPT,CDK1,CDK4,CDK6,CEP55,CNPY2,CRAT,CSNK1A1,CTBP2,CTTNBP2,CUL3,CWF19L1,CYB5R1,CYFIP1,DCTN6,DDB1,DDRGK1,DDX5,DGKZ,DGLUCY,DNAJC5,DNAJC9,DNMT1,DNPEP,DOCK1,DOCK5,DPY19L1,DPYSL2,DPYSL3,DTYMK,DYNC2H1,ECI2,EEF1B2,EEF2,EEF2K,EIF2B4,EIF2B5,ELOA,ELP1,ELP2,ELP3,EMC7,EMD,EML1,ERLIN2,EXOC2,EXOC4,EXTL3,FADD,FADS1,FAF2,FASN,FBXL15,FBXO7,FDFT1,FLII,FLNA,FLNB,FRMD4A,FUT8,GATM,GCDH,GCLC,GCLM,GDA,GFPT1,GHDC,GLRX3,GLRX5,GMDS,GMPPB,GOT2,GPT2,GRB14,GSDMD,GSTZ1,GTF2B,GTSE1,HACD3,HAUS7,HDGFL3,HEBP2,HIKESHI,HM13,HMGCL,HMGCS1,HMMR,HMOX1,HMOX2,HNRNPUL1,HSPA4L,IDH2,IFT74,IKBIP,IMPA1,INPP1,INPPL1,IRGQ,ISCA2,ITPR1,ITPR3,ITPRID2,KANK1,KANK2,KATNB1,KPNA2,LAMTOR2,LARP7,LAYN,LETM1,LIMD1,LOXL3,LPCAT3,LPP,LRRC40,LRRC59,LTA4H,MARK2,MAT2A,MEGF10,MKLN1,MMUT,MON2,MPI,MPST,MTDH,MTSS1,NAA25,NAB2,NAPA,NCLN,NDUFAF7,NEK9,NFKB2,NLE1,NLN,NPTXR,NQO1,NRBP1,NRDC,NT5C3B,NUP54,OAT,OGT,OSBPL8,OXCT1,PAFAH1B1,PAK1,PAPSS1,PARG,PCBP2,PCK2,PDE4DIP,PDZRN3,PEPD,PFKM,PHLDA1,PHYH,PIGU,PIP4P2,PITRM1,PKN2,PLCB1,PMPCB,POLA1,POLD1,POLD3,POLDIP3,PPA2,PPAT,PPOX,PPP2R5D,PPP4R3A,PRDX2,PRKACB,PRKCA,PRPF19,PRRC2A,PRRX1,PTPN1,QSOX2,RAB32,RABGGTA,RAD51,RAF1,RAI14,RANGAP1,RAP2C,RASSF4,RBPJ,RCN2,RER1,RETSAT,RIOK3,RNASEH2B,RPA1,RPA2,RPL27,RPL3,RPL5,RPL6,RPL7A,RPN1,RPN2,RRM1,RRM2,SAMD4B,SDF4,SEC14L1,SEC24A,SEC24D,SEC61A1,SERPINE2,SGTA,SH3GLB1,SLC1A4,SLC9A3R1,SLC9A3R2,SNF8,SNX1,SNX17,SOAT1,SORBS3,SPP1,SQLE,SQSTM1,SRGAP3,SRXN1,STAM2,STAT1,STAT3,STAT5B,STRN4,STT3A,SUCLG2,TAX1BP1,TCF12,TDRKH,TKFC,TLE3,TLN1,TM9SF3,TM9SF4,TMEM115,TMEM43,TNS3,TOM1L2,TPD52,TRAPPC4,TRIM2,TRIM28,TRIM3,TRIM47,TRIOBP,TRIP10,TRIP12,TRIP13,TRMT2A,TST,TTC21B,UBAP2L,UBE2C,UBE2O,UCKL1,USP24,USP47,USP8,USP9X,VCP,VCPIP1,VPS11,VPS13A,VPS36,VPS4B,VWA5A,WDR12,WDR45B,WDR62,WFS1,WIPF1,XRN1,ZMPSTE24	339
Cancer,Organismal Injury and Abnormalities,Renal and Urological Disease	Urinary tract cancer	0,000238		1	ACADS,ACSL3,AGL,AIP,AK3,ALDH2,ARFGAP2,ARHGEF1,ARHGEF18,ASRGL1,ATIC,ATP6AP1,ATPAF2,BIRC6,CAND1,CARMIL1,CAVIN1,CC2D1B,CDK4,CTTNBP2,CUL3,DDRGK1,DDX5,DGKZ,DOCK1,DOCK5,DPY19L1,DPYSL2,EEF1B2,EEF2K,ELOA,ERLIN2,FADD,FBXO7,FDFT1,FLII,FLNA,FRMD4A,GATM,GLRX5,GMPPB,GOT2,GRB14,HAUS7,HIKESHI,HMOX1,HNRNPUL1,IFT74,ITPR3,KATNB1,LTA4H,MARK2,MAT2A,MMUT,MON2,MTDH,NAA25,NQO1,NRDC,OGT,PAFAH1B1,PAK1,PCK2,PDE4DIP,PDZRN3,PHLDA1,PITRM1,PLCB1,PPAT,PRDX2,PRRC2A,RAB32,RAF1,RAI14,RCN2,RPL5,RPL6,RRM1,RRM2,SEC24A,SLC1A4,SLC9A3R1,SNF8,SNX17,SORBS3,SPP1,STAT3,TCF12,TKFC,TLN1,TNS3,TRIM28,TRIOBP,TRMT2A,UBAP2L,UBE2C,UCKL1,USP8,USP9X,VPS13A,WDR62	101
Cancer,Organismal Injury and Abnormalities,Renal and Urological Disease	Urinary tract tumor	0,000271		1	ACADS,ACSL3,AGL,AIP,AK3,ALDH2,ARFGAP2,ARHGEF1,ARHGEF18,ASRGL1,ATIC,ATP6AP1,ATPAF2,BIRC6,CAND1,CARMIL1,CAVIN1,CC2D1B,CDK4,CTTNBP2,CUL3,DDRGK1,DDX5,DGKZ,DOCK1,DOCK5,DPY19L1,DPYSL2,EEF1B2,EEF2K,ELOA,ERLIN2,FADD,FBXO7,FDFT1,FLII,FLNA,FRMD4A,GATM,GLRX5,GMPPB,GOT2,GRB14,HAUS7,HIKESHI,HMOX1,HNRNPUL1,IFT74,ITPR3,KATNB1,LTA4H,MARK2,MAT2A,MMUT,MON2,MTDH,NAA25,NQO1,NRDC,OGT,PAFAH1B1,PAK1,PCK2,PDE4DIP,PDZRN3,PHLDA1,PITRM1,PLCB1,PPAT,PRDX2,PRKCA,PRRC2A,RAB32,RAF1,RAI14,RCN2,RPL5,RPL6,RRM1,RRM2,SEC24A,SLC1A4,SLC9A3R1,SNF8,SNX17,SORBS3,SPP1,STAT3,TCF12,TKFC,TLN1,TNS3,TRIM28,TRIOBP,TRMT2A,UBAP2L,UBE2C,UCKL1,USP8,USP9X,VPS13A,WDR62	102
Cancer,Organismal Injury and Abnormalities	Extracranial solid tumor	4,8E-20		1,015	AARSD1,ABCD4,ACAA2,ACACA,ACADS,ACO1,ACO2,ACP2,ACSF2,ACSL3,ACSL4,ADD1,ADI1,ADO,AGA,AGFG1,AGL,AIP,AK3,AKR1B1,AKR1B10,AKR7A2,ALDH2,ALDH4A1,ALDH6A1,ALDH9A1,ANAPC7,ANKRD13A,ANKRD46,ANXA4,AP3S2,APPL2,ARFGAP2,ARFGAP3,ARFGEF2,ARHGEF1,ARHGEF12,ARHGEF18,ARMC8,ARPIN/ARPIN-AP3S2,ASRGL1,ATIC,ATP2A2,ATP2B4,ATP6AP1,ATP6V1A,ATPAF2,ATXN10,BCAS1,BCAT2,BIRC6,C17orf49,C1QBP,CACYBP,CAMSAP2,CAND1,CANX,CARMIL1,CAVIN1,CBX3,CC2D1B,CCDC47,CCZ1/CCZ1B,CDC37L1,CDC42EP4,CDIPT,CDK1,CDK4,CDK6,CEP55,CERT1,CNEP1R1,CNN2,CNN3,CNPY2,CRAT,CSNK1A1,CSRP1,CTBP2,CTTNBP2,CUL3,CWF19L1,CYB5R1,Cyb5r3,CYFIP1,DCTN6,DDB1,DDRGK1,DDX5,DGKZ,DGLUCY,DNAJC5,DNAJC9,DNMT1,DNPEP,DOCK1,DOCK5,DPY19L1,DPYSL2,DPYSL3,DTYMK,DYNC2H1,ECI2,EEF1B2,EEF2,EEF2K,EIF2B4,EIF2B5,ELOA,ELP1,ELP2,ELP3,EMC7,EMD,EML1,ERLIN2,EXOC2,EXOC4,EXTL3,FADD,FADS1,FAF2,FASN,FBXL15,FBXO7,FDFT1,FLII,FLNA,FLNB,FRMD4A,FUT8,GABPA,GATM,GCDH,GCLC,GCLM,GDA,GFPT1,GHDC,GLRX,GLRX3,GLRX5,GMDS,GMPPB,GOT2,GPT2,GRB14,GSDMD,GSTZ1,GTF2B,GTSE1,HACD3,HAUS7,HDGFL3,HEBP2,HIKESHI,HM13,HMGCL,HMGCS1,HMMR,HMOX1,HMOX2,HNRNPUL1,HSPA4L,IDH2,IFT74,IKBIP,IMPA1,INPP1,INPPL1,IRGQ,ISCA2,ITPR1,ITPR3,ITPRID2,KANK1,KANK2,KATNB1,KPNA2,LAMTOR2,LAMTOR3,LARP7,LAYN,LETM1,LIMD1,LOXL3,LPCAT3,LPP,LRRC40,LRRC59,LTA4H,MARK2,MAT2A,MEGF10,MGST2,MKLN1,MMUT,MON2,MPI,MPST,MTDH,MTSS1,NAA25,NAB2,NAPA,NAXE,NCLN,NDC80,NDUFAF7,NEK9,NFKB2,NFS1,NIT2,NLE1,NLN,NMD3,NPTXR,NQO1,NRBP1,NRDC,NT5C3B,NUP54,OAT,OGT,OSBPL8,OXCT1,PAFAH1B1,PAK1,PAPSS1,PARG,PCBP2,PCK2,PDE4DIP,PDZRN3,PEPD,PFKM,PGD,PHLDA1,PHYH,PIGU,PIP4P2,PITRM1,PKN2,PLCB1,PMPCB,POLA1,POLD1,POLD3,POLDIP3,PPA2,PPAT,PPOX,PPP2R5D,PPP4R3A,PRDX2,PRKACB,PRKCA,PRPF19,PRRC2A,PRRX1,PTPN1,QSOX2,RAB32,RAB6A,RABGGTA,RAD51,RAF1,RAI14,RANGAP1,RAP2C,RASSF4,RBPJ,RCN2,RER1,RETSAT,RIOK3,RNASEH2B,RPA1,RPA2,RPL13,RPL27,RPL3,RPL5,RPL6,RPL7A,RPN1,RPN2,RRM1,RRM2,SAMD4B,SDF4,SEC14L1,SEC24A,SEC24D,SEC61A1,SERPINE2,SFR1,SGTA,SH3GLB1,SLC1A4,SLC9A3R1,SLC9A3R2,SNF8,SNX1,SNX17,SOAT1,SORBS3,SPP1,SQLE,SQSTM1,SRGAP3,SRXN1,STAM2,STARD3NL,STAT1,STAT3,STAT5B,STRN4,STT3A,SUCLG2,TAGLN2,TAX1BP1,TCF12,TDRKH,TKFC,TLE3,TLN1,TM9SF3,TM9SF4,TMEM115,TMEM43,TNS3,TOM1L2,TPD52,TRAPPC4,TRIM2,TRIM28,TRIM3,TRIM47,TRIOBP,TRIP10,TRIP12,TRIP13,TRMT2A,TST,TTC21B,UBAP2L,UBE2C,UBE2I,UBE2O,UBR7,UCKL1,USP24,USP47,USP8,USP9X,VAMP7,VAT1,VCP,VCPIP1,VPS11,VPS13A,VPS36,VPS4B,VWA5A,WDR12,WDR45B,WDR62,WFS1,WIPF1,XRN1,ZMPSTE24	372
Cell Cycle	M phase	0,000351		1,04	BIRC6,CDK1,CEP55,CUL3,FADD,FLNA,HMMR,NDC80,NEK9,PKN2,RAD51,SH3GLB1,TRIP13,UBAP2L,UBE2C	15
Cancer,Organismal Injury and Abnormalities	Metastatic solid tumor	0,0000387		1,054	ATIC,CDK4,CDK6,CSNK1A1,DPYSL2,DPYSL3,ELP3,FADD,FASN,FDFT1,FLNA,GLRX,HMMR,HMOX1,ITPR3,NQO1,PAFAH1B1,PAK1,PDZRN3,POLA1,POLD1,PPAT,PRDX2,PRRX1,RAF1,RIOK3,RPL27,RRM1,RRM2,SH3GLB1,SPP1,SQSTM1,STAT1,STAT3,TCF12	35
Cancer,Organismal Injury and Abnormalities	Advanced malignant solid tumor	0,0000733		1,054	ATIC,CDK4,CDK6,CSNK1A1,DNMT1,DPYSL2,DPYSL3,ELP3,FADD,FASN,FDFT1,FLII,FLNA,GLRX,HMMR,HMOX1,ITPR3,NQO1,PAFAH1B1,PAK1,PDZRN3,POLA1,POLD1,PPAT,PRDX2,PRRX1,RAF1,RIOK3,RPL27,RRM1,RRM2,SERPINE2,SH3GLB1,SPP1,SQSTM1,STAT1,STAT3,TCF12	38
Cancer,Gastrointestinal Disease,Organismal Injury and Abnormalities	Hepato-pancreato-biliary cancer	2,09E-07		1,076	ABCD4,ACAA2,ACO1,ACO2,ACSF2,ACSL3,ACSL4,ADD1,AGA,AGFG1,AGL,AKR1B10,ALDH2,ALDH4A1,ALDH6A1,ALDH9A1,APPL2,ARFGEF2,ARHGEF12,ASRGL1,ATIC,ATP2A2,ATP2B4,ATXN10,BIRC6,C1QBP,CACYBP,CAND1,CANX,CARMIL1,CAVIN1,CBX3,CC2D1B,CDC37L1,CDK1,CDK4,CDK6,CNPY2,CTBP2,CTTNBP2,DDB1,DGLUCY,DNAJC9,DOCK1,DOCK5,DPY19L1,DPYSL3,DYNC2H1,ECI2,EEF2,EEF2K,ELOA,EMC7,EML1,ERLIN2,EXOC2,EXOC4,EXTL3,FADS1,FASN,FBXL15,FLNA,FLNB,FRMD4A,GCLC,GMPPB,GPT2,GRB14,GSDMD,GSTZ1,GTF2B,GTSE1,HACD3,HDGFL3,HMGCS1,HMMR,HMOX1,HNRNPUL1,IDH2,IFT74,INPP1,INPPL1,IRGQ,ITPR1,ITPR3,ITPRID2,KANK1,KANK2,KPNA2,LAMTOR2,LARP7,LETM1,LIMD1,LOXL3,LPP,LRRC59,LTA4H,MARK2,MAT2A,MKLN1,MMUT,MON2,MPI,MTSS1,NAA25,NAB2,NAPA,NCLN,NQO1,NRDC,OGT,OSBPL8,OXCT1,PAK1,PARG,PCBP2,PCK2,PDE4DIP,PDZRN3,PFKM,PHLDA1,PIGU,PIP4P2,PITRM1,PKN2,PLCB1,POLA1,POLD1,POLD3,POLDIP3,PPA2,PPOX,PPP2R5D,PRKACB,PRKCA,PRRC2A,PTPN1,RABGGTA,RAF1,RANGAP1,RBPJ,RCN2,RER1,RETSAT,RIOK3,RPA1,RPL6,RPL7A,RPN1,RPN2,RRM1,RRM2,SEC14L1,SEC24A,SEC24D,SERPINE2,SH3GLB1,SLC1A4,SLC9A3R1,SOAT1,SPP1,SQLE,SQSTM1,SRGAP3,SRXN1,STAM2,STAT1,STAT3,STAT5B,TAX1BP1,TCF12,TKFC,TLE3,TLN1,TM9SF3,TM9SF4,TNS3,TPD52,TRAPPC4,TRIM2,TRIM28,TRIM3,TRIOBP,TRIP10,TRMT2A,TST,TTC21B,UBE2C,USP24,USP47,USP9X,VCP,VCPIP1,VPS13A,VPS36,VPS4B,WDR12,WDR62,WFS1,WIPF1,XRN1	201
Cancer,Organismal Injury and Abnormalities,Respiratory Disease	Advanced lung cancer	0,000382		1,091	ATIC,CDK4,CDK6,DPYSL3,ELP3,FADD,FDFT1,PRDX2,RAF1,RIOK3,RPL27,RRM1,RRM2,SPP1,SQSTM1,STAT3	16

Categories	Diseases or Functions Annotation	p-value	Predicted Activation State	Activation z-score	Molecules	# Molecules
Cancer,Organismal Injury and Abnormalities	Extrapancreatic malignant tumor	7,04E-20		1,097	AARSD1,ABCD4,ACAA2,ACACA,ACADS,ACO1,ACO2,ACP2,ACSF2,ACSL3,ACSL4,ADD1,ADI1,AGA,AGFG1,AGL,AIP,AK3,AKR1B1,AKR1B10,AKR7A2,ALDH2,ALDH4A1,ALDH6A1,ALDH9A1,ANAPC7,ANKRD13A,ANXA4,AP3S2,APPL2,ARFGAP2,ARFGAP3,ARFGEF2,ARHGEF1,ARHGEF12,ARHGEF18,ARMC8,ARPIN/ARPIN-AP3S2,ASRGL1,ATIC,ATP2A2,ATP2B4,ATP6AP1,ATP6V1A,ATPAF2,ATXN10,BCAS1,BCAT2,BIRC6,C17orf49,C1QBP,CACYBP,CAMSAP2,CAND1,CANX,CARMIL1,CAVIN1,CBX3,CC2D1B,CCDC47,CCZ1/CCZ1B,CDC37L1,CDC42EP4,CDIPT,CDK1,CDK4,CDK6,CEP55,CERT1,CNN2,CNN3,CNPY2,CRAT,CSNK1A1,CSRP1,CTBP2,CTTNBP2,CUL3,CWF19L1,CYB5R1,Cyb5r3,CYFIP1,DCTN6,DDB1,DDRGK1,DDX5,DGKZ,DGLUCY,DNAJC5,DNAJC9,DNMT1,DNPEP,DOCK1,DOCK5,DPY19L1,DPYSL2,DPYSL3,DTYMK,DYNC2H1,ECI2,EEF1B2,EEF2,EEF2K,EIF2B4,EIF2B5,ELOA,ELP1,ELP2,ELP3,EMC7,EMD,EML1,ERLIN2,EXOC2,EXOC4,EXTL3,FADD,FADS1,FAF2,FASN,FBXL15,FBXO7,FDFT1,FLII,FLNA,FLNB,FRMD4A,FUT8,GABPA,GATM,GCDH,GCLC,GCLM,GDA,GFPT1,GHDC,GLRX,GLRX3,GLRX5,GMDS,GMPPB,GOT2,GPT2,GRB14,GSDMD,GSTZ1,GTF2B,GTSE1,HACD3,HAUS7,HDGFL3,HEBP2,HIKESHI,HM13,HMGCL,HMGCS1,HMMR,HMOX1,HMOX2,HNRNPUL1,HSPA4L,IDH2,IFT74,IKBIP,IMPA1,INPP1,INPPL1,IRGQ,ISCA2,ITPR1,ITPR3,ITPRID2,KANK1,KANK2,KATNB1,KPNA2,LAMTOR2,LARP7,LAYN,LETM1,LIMD1,LOXL3,LPCAT3,LPP,LRRC40,LRRC59,LTA4H,MARK2,MAT2A,MEGF10,MGST2,MKLN1,MMUT,MON2,MPI,MPST,MTDH,MTSS1,NAA25,NAB2,NAPA,NARS1,NCLN,NDC80,NDUFAF7,NEK9,NFKB2,NIT2,NLE1,NLN,NMD3,NPTXR,NQO1,NRBP1,NRDC,NT5C3B,NUP54,OAT,OGT,OSBPL8,OXCT1,PAFAH1B1,PAK1,PAPSS1,PARG,PCBP2,PCK2,PDE4DIP,PDZRN3,PEPD,PFKM,PHLDA1,PHYH,PIGU,PIP4P2,PITRM1,PKN2,PLCB1,PMPCB,POLA1,POLD1,POLD3,POLDIP3,PPA2,PPAT,PPOX,PPP2R5D,PPP4R3A,PRDX2,PRKACB,PRKCA,PRPF19,PRRC2A,PRRX1,PTPN1,QSOX2,RAB32,RABGGTA,RAD51,RAF1,RAI14,RANGAP1,RAP2C,RASSF4,RBPJ,RCN2,RER1,RETSAT,RIOK3,RNASEH2B,RPA1,RPA2,RPL13,RPL27,RPL3,RPL5,RPL6,RPL7A,RPN1,RPN2,RRM1,RRM2,SAMD4B,SDF4,SEC14L1,SEC24A,SEC24D,SEC61A1,SERPINE2,SGTA,SH3GLB1,SLC1A4,SLC9A3R1,SLC9A3R2,SNF8,SNX1,SNX17,SOAT1,SORBS3,SPCS3,SPP1,SQLE,SQSTM1,SRGAP3,SRXN1,STAM2,STARD3NL,STAT1,STAT3,STAT5B,STRN4,STT3A,SUCLG2,TAGLN2,TAX1BP1,TCF12,TDRKH,TKFC,TLE3,TLN1,TM9SF3,TM9SF4,TMEM115,TMEM43,TNS3,TOM1L2,TPD52,TRAPPC4,TRIM2,TRIM28,TRIM3,TRIM47,TRIOBP,TRIP10,TRIP12,TRIP13,TRMT2A,TST,TTC21B,UBAP2L,UBE2C,UBE2I,UBE2O,UBR7,UCKL1,USP24,USP47,USP8,USP9X,VAMP7,VAT1,VCP,VCPIP1,VPS11,VPS13A,VPS36,VPS4B,VWA5A,WDR12,WDR45B,WDR62,WFS1,WIPF1,XRN1,ZMPSTE24	365
Cancer,Organismal Injury and Abnormalities	Abdominal neoplasm	1,49E-14		1,124	AARSD1,ABCD4,ACAA2,ACADS,ACO1,ACO2,ACP2,ACSF2,ACSL3,ACSL4,ADD1,ADI1,AGA,AGFG1,AGL,AIP,AK3,AKR1B1,AKR1B10,AKR7A2,ALDH2,ALDH4A1,ALDH6A1,ALDH9A1,ANKRD13A,ANKRD46,ANXA4,AP3S2,APPL2,ARFGAP2,ARFGAP3,ARFGEF2,ARHGEF1,ARHGEF12,ARHGEF18,ARMC8,ASRGL1,ATIC,ATP2A2,ATP2B4,ATP6AP1,ATP6V1A,ATPAF2,ATXN10,BCAS1,BCAT2,BIRC6,C1QBP,CACYBP,CAMSAP2,CAND1,CANX,CARMIL1,CAVIN1,CBX3,CC2D1B,CCDC47,CCZ1/CCZ1B,CDC37L1,CDC42EP4,CDIPT,CDK1,CDK4,CDK6,CEP55,CNPY2,CRAT,CSNK1A1,CTBP2,CTTNBP2,CUL3,CWF19L1,CYB5R1,CYFIP1,DCTN6,DDB1,DDRGK1,DDX5,DGKZ,DGLUCY,DNAJC5,DNAJC9,DNMT1,DNPEP,DOCK1,DOCK5,DPY19L1,DPYSL2,DPYSL3,DTYMK,DYNC2H1,ECI2,EEF1B2,EEF2,EEF2K,EIF2B4,EIF2B5,ELOA,ELP1,ELP2,ELP3,EMC7,EMD,EML1,ERLIN2,EXOC2,EXOC4,EXTL3,FADD,FADS1,FAF2,FASN,FBXL15,FBXO7,FDFT1,FLII,FLNA,FLNB,FRMD4A,FUT8,GATM,GCDH,GCLC,GCLM,GDA,GFPT1,GHDC,GLRX,GLRX3,GLRX5,GMDS,GMPPB,GOT2,GPT2,GRB14,GSDMD,GSTZ1,GTF2B,GTSE1,HACD3,HAUS7,HDGFL3,HEBP2,HIKESHI,HM13,HMGCL,HMGCS1,HMMR,HMOX1,HMOX2,HNRNPUL1,HSPA4L,IDH2,IFT74,IKBIP,IMPA1,INPP1,INPPL1,IRGQ,ISCA2,ITPR1,ITPR3,ITPRID2,KANK1,KANK2,KATNB1,KPNA2,LAMTOR2,LARP7,LAYN,LETM1,LIMD1,LOXL3,LPCAT3,LPP,LRRC40,LRRC59,LTA4H,MARK2,MAT2A,MEGF10,MKLN1,MMUT,MON2,MPI,MPST,MTDH,MTSS1,NAA25,NAB2,NAPA,NCLN,NDUFAF7,NEK9,NFKB2,NLE1,NLN,NMD3,NPTXR,NQO1,NRBP1,NRDC,NT5C3B,NUP54,OAT,OGT,OSBPL8,OXCT1,PAFAH1B1,PAK1,PAPSS1,PARG,PCBP2,PCK2,PDE4DIP,PDZRN3,PEPD,PFKM,PHLDA1,PHYH,PIGU,PIP4P2,PITRM1,PKN2,PLCB1,PMPCB,POLA1,POLD1,POLD3,POLDIP3,PPA2,PPAT,PPOX,PPP2R5D,PPP4R3A,PRDX2,PRKACB,PRKCA,PRPF19,PRRC2A,PRRX1,PTPN1,QSOX2,RAB32,RAB6A,RABGGTA,RAD51,RAF1,RAI14,RANGAP1,RAP2C,RASSF4,RBPJ,RCN2,RER1,RETSAT,RIOK3,RNASEH2B,RPA1,RPA2,RPL27,RPL3,RPL5,RPL6,RPL7A,RPN1,RPN2,RRM1,RRM2,SAMD4B,SDF4,SEC14L1,SEC24A,SEC24D,SEC61A1,SERPINE2,SGTA,SH3GLB1,SLC1A4,SLC9A3R1,SLC9A3R2,SNF8,SNX1,SNX17,SOAT1,SORBS3,SPP1,SQLE,SQSTM1,SRGAP3,SRXN1,STAM2,STAT1,STAT3,STAT5B,STRN4,STT3A,SUCLG2,TAGLN2,TAX1BP1,TCF12,TDRKH,TKFC,TLE3,TLN1,TM9SF3,TM9SF4,TMEM115,TMEM43,TNS3,TOM1L2,TPD52,TRAPPC4,TRIM2,TRIM28,TRIM3,TRIM47,TRIOBP,TRIP10,TRIP12,TRIP13,TRMT2A,TST,TTC21B,UBAP2L,UBE2C,UBE2I,UBE2O,UCKL1,USP24,USP47,USP8,USP9X,VCP,VCPIP1,VPS11,VPS13A,VPS36,VPS4B,VWA5A,WDR12,WDR45B,WDR62,WFS1,WIPF1,XRN1,ZMPSTE24	347
Cancer,Cell Death and Survival,Organismal Injury and Abnormalities,Tumor Morphology	Cell death of osteosarcoma cells	0,00000246		1,155	NAPA,NUP54,RPA2,RPL13,RPL27,RPL3,RPL5,RPL6,RPL7A,RRM2,UBE2I,VCP	12
Cell Death and Survival	Apoptosis of prostate cancer cell lines	0,000193		1,208	ACACA,ADI1,BIRC6,FADD,FASN,NAPA,PAK1,PRKCA,RABGGTA,RAF1,STAT3,TLN1,TPD52	13
Cancer,Organismal Injury and Abnormalities	Metastasis	0,0000209		1,249	ADI1,ARHGEF1,ATIC,C1QBP,CDK4,CDK6,CSNK1A1,DNMT1,DOCK5,DPYSL2,DPYSL3,ELP3,FADD,FASN,FBXO7,FDFT1,FLNA,FUT8,GLRX,GPT2,HMMR,HMOX1,IDH2,ITPR3,MTDH,NQO1,PAFAH1B1,PAK1,PDZRN3,POLA1,POLD1,PPAT,PRDX2,PRKCA,PRRX1,RAD51,RAF1,RBPJ,RIOK3,RPL27,RPL3,RRM1,RRM2,SH3GLB1,SLC9A3R2,SPP1,SQSTM1,STAT1,STAT3,TCF12,TLN1,USP9X	52
Cancer,Organismal Injury and Abnormalities	Advanced malignant tumor	0,0000539		1,249	ADI1,ARHGEF1,ATIC,C1QBP,CDK4,CDK6,CSNK1A1,DNMT1,DOCK5,DPYSL2,DPYSL3,ELP3,FADD,FASN,FBXO7,FDFT1,FLII,FLNA,FUT8,GLRX,GPT2,HMMR,HMOX1,IDH2,ITPR3,MTDH,NQO1,PAFAH1B1,PAK1,PDZRN3,POLA1,POLD1,PPAT,PRDX2,PRKCA,PRRX1,RAD51,RAF1,RBPJ,RIOK3,RPL27,RPL3,RRM1,RRM2,SERPINE2,SH3GLB1,SLC9A3R2,SPP1,SQSTM1,STAT1,STAT3,TCF12,TLN1,USP9X	54
Cancer,Organismal Injury and Abnormalities	Invasive tumor	0,000144		1,249	ADI1,ARHGEF1,ATIC,C1QBP,CAVIN1,CDK4,CDK6,CSNK1A1,DNMT1,DOCK5,DPYSL2,DPYSL3,ELP3,FADD,FASN,FBXO7,FDFT1,FLNA,FUT8,GLRX,GPT2,HMMR,HMOX1,IDH2,ITPR3,MTDH,NQO1,PAFAH1B1,PAK1,PDZRN3,POLA1,POLD1,PPAT,PRDX2,PRKCA,PRRX1,PTPN1,RAD51,RAF1,RBPJ,RIOK3,RPL27,RPL3,RRM1,RRM2,SH3GLB1,SLC9A3R2,SPP1,SQSTM1,STAT1,STAT3,TCF12,TLE3,TLN1,USP9X,ZMPSTE24	56
Cancer,Organismal Injury and Abnormalities	Genitourinary tumor	1,22E-10		1,27	AARSD1,ABCD4,ACACA,ACADS,ACO1,ACP2,ACSF2,ACSL3,ACSL4,ADD1,ADI1,AGL,AIP,AK3,AKR1B1,AKR7A2,ALDH2,ALDH4A1,ALDH9A1,ANKRD13A,AP3S2,APPL2,ARFGAP2,ARFGAP3,ARFGEF2,ARHGEF1,ARHGEF12,ARHGEF18,ASRGL1,ATIC,ATP2B4,ATP6AP1,ATP6V1A,ATPAF2,BCAS1,BIRC6,C17orf49,C1QBP,CAMSAP2,CAND1,CANX,CARMIL1,CAVIN1,CBX3,CC2D1B,CCDC47,CCZ1/CCZ1B,CDC42EP4,CDIPT,CDK1,CDK4,CDK6,CEP55,CERT1,CNN2,CNPY2,CRAT,CSNK1A1,CTBP2,CTTNBP2,CUL3,Cyb5r3,DDB1,DDRGK1,DDX5,DGKZ,DGLUCY,DNMT1,DOCK1,DOCK5,DPY19L1,DPYSL2,DPYSL3,DTYMK,DYNC2H1,EEF1B2,EEF2,EEF2K,ELOA,ELP1,ELP2,ELP3,EMC7,EMD,EML1,ERLIN2,EXOC2,EXOC4,FADD,FAF2,FASN,FBXO7,FDFT1,FLII,FLNA,FLNB,FRMD4A,FUT8,GABPA,GATM,GCDH,GDA,GFPT1,GHDC,GLRX3,GLRX5,GMDS,GMPPB,GOT2,GPT2,GRB14,GSDMD,GTSE1,HAUS7,HDGFL3,HEBP2,HIKESHI,HMGCS1,HMMR,HMOX1,HNRNPUL1,HSPA4L,IDH2,IFT74,IKBIP,INPPL1,IRGQ,ITPR1,ITPR3,KANK1,KATNB1,KPNA2,LARP7,LAYN,LETM1,LIMD1,LOXL3,LPP,LRRC40,LTA4H,MARK2,MAT2A,MEGF10,MGST2,MKLN1,MMUT,MON2,MPST,MTDH,MTSS1,NAA25,NAB2,NAPA,NCLN,NDC80,NEK9,NFKB2,NLE1,NMD3,NPTXR,NQO1,NRDC,NUP54,OAT,OGT,OSBPL8,OXCT1,PAFAH1B1,PAK1,PAPSS1,PARG,PCBP2,PCK2,PDE4DIP,PDZRN3,PEPD,PFKM,PHLDA1,PHYH,PIGU,PIP4P2,PITRM1,PKN2,PLCB1,POLA1,POLD1,POLD3,POLDIP3,PPAT,PPOX,PRDX2,PRKACB,PRKCA,PRPF19,PRRC2A,PRRX1,PTPN1,RAB32,RAD51,RAF1,RAI14,RANGAP1,RAP2C,RCN2,RER1,RETSAT,RIOK3,RNASEH2B,RPA1,RPA2,RPL27,RPL5,RPL6,RPN1,RRM1,RRM2,SAMD4B,SDF4,SEC14L1,SEC24A,SEC24D,SEC61A1,SERPINE2,SGTA,SH3GLB1,SLC1A4,SLC9A3R1,SNF8,SNX1,SNX17,SOAT1,SORBS3,SPP1,SQLE,SQSTM1,STAT1,STAT3,STAT5B,STT3A,SUCLG2,TAGLN2,TAX1BP1,TCF12,TKFC,TLE3,TLN1,TMEM43,TNS3,TOM1L2,TPD52,TRAPPC4,TRIM28,TRIM3,TRIOBP,TRIP10,TRIP12,TRIP13,TRMT2A,TST,UBAP2L,UBE2C,UBE2O,UCKL1,USP24,USP47,USP8,USP9X,VCP,VCPIP1,VPS11,VPS13A,VPS36,VWA5A,WDR12,WDR62,WFS1,WIPF1,XRN1,ZMPSTE24	279
Cell Death and Survival	Apoptosis	4,45E-11		1,306	ACACA,ACO2,ACSL4,ADI1,AGA,AIP,AKR1B1,AKR1B10,ALDH2,Anp32b,ANXA4,ARHGEF1,ARHGEF12,ARHGEF18,ATP2B4,BIRC6,C1QBP,CANX,CBX3,CCDC47,CDK1,CDK4,CDK6,CERT1,CNN2,CSNK1A1,CTBP2,CUL3,DDB1,DDRGK1,DDX5,DNAJC5,DNMT1,DTYMK,EEF2K,EIF2B5,ELOA,ELP1,ELP3,EMD,EXOC2,FADD,FASN,FLNA,FLNB,GCDH,GCLC,GCLM,GFPT1,GLRX,GMDS,GOT2,GSTZ1,GTSE1,HEBP2,HMMR,HMOX1,HMOX2,INPPL1,ITPR1,ITPR3,KANK2,KPNA2,LAMTOR2,MAT2A,MEGF10,MTDH,MTSS1,NAPA,NDC80,NFKB2,NLE1,NQO1,OGT,OSBPL8,PAFAH1B1,PAK1,PARG,PCBP2,PCK2,PHLDA1,PKN2,POLA1,PRDX2,PRKACB,PRKCA,PRPF19,PTPN1,RAB32,RABGGTA,RAD51,RAF1,RAI14,RASSF4,RBPJ,RPA1,RPA2,RRM1,RRM2,SDF4,Serbp1,SERPINE2,SH3GLB1,SLC9A3R1,SNX1,SOAT1,SPP1,SQSTM1,STAT1,STAT3,STAT5B,TAGLN2,TAX1BP1,TCF12,TLN1,TM9SF4,TNFAIP8,TPD52,TRIM2,TRIM28,UBE2I,USP47,VCP,VPS13A,WFS1,ZMPSTE24	126
Cell Death and Survival	Necrosis of prostate cancer cell lines	0,0000163		1,35	ACACA,ADI1,BIRC6,FADD,FASN,FDFT1,NAPA,NFKB2,PAK1,PRKCA,RABGGTA,RAF1,SH3GLB1,STAT3,TLN1,TPD52	16
Cancer,Gastrointestinal Disease,Organismal Injury and Abnormalities	Hepatobiliary carcinoma	0,00000332		1,404	ACAA2,ACO1,ACO2,ACSL3,ACSL4,ADD1,AGA,AGFG1,AKR1B10,ALDH2,ALDH4A1,ALDH6A1,ALDH9A1,APPL2,ARFGEF2,ARHGEF12,ASRGL1,ATIC,ATP2A2,BIRC6,CACYBP,CAND1,CANX,CARMIL1,CAVIN1,CBX3,CDC37L1,CDK1,CDK4,CDK6,CNPY2,CTBP2,CTTNBP2,DGLUCY,DNAJC9,DOCK1,DPYSL3,DYNC2H1,ECI2,EEF2,EEF2K,ERLIN2,EXOC2,EXOC4,FADS1,FASN,FBXL15,FLNA,FLNB,FRMD4A,GCLC,GPT2,GRB14,GSDMD,GSTZ1,GTF2B,GTSE1,HDGFL3,HMGCS1,HMMR,HMOX1,HNRNPUL1,IDH2,IFT74,INPP1,IRGQ,ITPR1,ITPR3,ITPRID2,KANK1,KANK2,KPNA2,LAMTOR2,LARP7,LOXL3,LPP,LRRC59,MARK2,MMUT,MON2,MPI,MTSS1,NAA25,NAPA,NCLN,NQO1,NRDC,OGT,OSBPL8,OXCT1,PAK1,PARG,PCBP2,PCK2,PDE4DIP,PDZRN3,PHLDA1,PIGU,PIP4P2,PITRM1,PKN2,PLCB1,POLA1,POLD1,POLD3,POLDIP3,PPA2,PPOX,PPP2R5D,PRRC2A,PTPN1,RAF1,RANGAP1,RBPJ,RCN2,RER1,RETSAT,RIOK3,RPL6,RPL7A,RPN1,RPN2,RRM1,RRM2,SEC24A,SEC24D,SH3GLB1,SLC1A4,SOAT1,SPP1,SQLE,SQSTM1,SRXN1,STAM2,STAT1,STAT3,STAT5B,TAX1BP1,TCF12,TM9SF3,TM9SF4,TNS3,TPD52,TRAPPC4,TRIM2,TRIOBP,TRIP10,TRMT2A,TST,TTC21B,UBE2C,USP24,USP47,VCP,VCPIP1,VPS13A,VPS36,VPS4B,WDR12,WDR62,WIPF1,XRN1	162
Metabolic Disease,Organismal Injury and Abnormalities,Renal and Urological Disease	Organic aciduria	0,00000729		1,432	ABCD4,CDK4,GCDH,GSTZ1,IDH2,MMUT,OAT,SUCLG2	8
Cell Death and Survival	Necrosis	5,92E-20		1,433	ACACA,ACO2,ACSL4,ADI1,AGA,AIP,AKR1B1,AKR1B10,ALDH2,ARHGEF1,ATIC,ATP2A2,ATP2B4,BIRC6,CBX3,CCDC47,CDK1,CDK4,CDK6,CERT1,CNN2,CNPY2,CSNK1A1,CTBP2,CUL3,CYB5R1,DNAJC5,DNMT1,DOCK1,DPYSL3,DTYMK,EEF2,EEF2K,EIF2B4,EIF2B5,ELOA,ELP1,ELP3,EMD,EXOC2,FADD,FADS1,FASN,FBXO7,FDFT1,FLNA,FLNB,GCDH,GCLC,GCLM,GDA,GFPT1,GLRX,GMDS,GOT2,GSDMD,GSTZ1,GTSE1,HMGCS1,HMMR,HMOX1,HMOX2,IDH2,IFT74,INPPL1,ITPR1,ITPR3,KANK2,KPNA2,LAMTOR2,LETM1,MAT2A,MPI,MTDH,MTSS1,NAPA,NDC80,NFKB2,NFS1,NQO1,NUP54,OGT,OSBPL8,PAFAH1B1,PAK1,PARG,PCBP2,PCK2,PFKM,PHLDA1,PKN2,PLCB1,POLA1,POLD1,PPAT,PRDX2,PRKACB,PRKCA,PRPF19,PTPN1,RAB32,RABGGTA,RAD51,RAF1,RAI14,RASSF4,RBPJ,RNASEH2B,RPA1,RPA2,RPL13,RPL27,RPL3,RPL5,RPL6,RPL7A,RRM1,RRM2,SDF4,SERPINE2,SFR1,SH3GLB1,SLC9A3R1,SLC9A3R2,SNX1,SOAT1,SPP1,SQLE,SQSTM1,STAM2,STAT1,STAT3,STAT5B,TAGLN2,TAX1BP1,TCF12,TLN1,TM9SF4,TNFAIP8,TPD52,TRIM2,TRIM28,TRIP10,UBE2C,UBE2I,USP47,USP9X,VCP,VPS13A,WFS1,WIPF1,ZMPSTE24	152
Cancer,Gastrointestinal Disease,Hepatic System Disease,Organismal Injury and Abnormalities	Hepatobiliary system cancer	0,00000331		1,456	ACAA2,ACO1,ACO2,ACSL3,ACSL4,ADD1,AGA,AGFG1,AKR1B10,ALDH2,ALDH4A1,ALDH6A1,ALDH9A1,APPL2,ARFGEF2,ARHGEF12,ASRGL1,ATIC,ATP2A2,BIRC6,CACYBP,CAND1,CANX,CARMIL1,CAVIN1,CBX3,CDC37L1,CDK1,CDK4,CDK6,CNPY2,CTBP2,CTTNBP2,DGLUCY,DNAJC9,DOCK1,DPYSL3,DYNC2H1,ECI2,EEF2,EEF2K,ERLIN2,EXOC2,EXOC4,FADS1,FASN,FBXL15,FLNA,FLNB,FRMD4A,GCLC,GPT2,GRB14,GSDMD,GSTZ1,GTF2B,GTSE1,HDGFL3,HMGCS1,HMMR,HMOX1,HNRNPUL1,IDH2,IFT74,INPP1,IRGQ,ITPR1,ITPR3,ITPRID2,KANK1,KANK2,KPNA2,LAMTOR2,LARP7,LETM1,LOXL3,LPP,LRRC59,MARK2,MAT2A,MMUT,MON2,MPI,MTSS1,NAA25,NAPA,NCLN,NQO1,NRDC,OGT,OSBPL8,OXCT1,PAK1,PARG,PCBP2,PCK2,PDE4DIP,PDZRN3,PHLDA1,PIGU,PIP4P2,PITRM1,PKN2,PLCB1,POLA1,POLD1,POLD3,POLDIP3,PPA2,PPOX,PPP2R5D,PRRC2A,PTPN1,RAF1,RANGAP1,RBPJ,RCN2,RER1,RETSAT,RIOK3,RPL6,RPL7A,RPN1,RPN2,RRM1,RRM2,SEC24A,SEC24D,SH3GLB1,SLC1A4,SOAT1,SPP1,SQLE,SQSTM1,SRXN1,STAM2,STAT1,STAT3,STAT5B,TAX1BP1,TCF12,TM9SF3,TM9SF4,TNS3,TPD52,TRAPPC4,TRIM2,TRIM28,TRIOBP,TRIP10,TRMT2A,TST,TTC21B,UBE2C,USP24,USP47,USP9X,VCP,VCPIP1,VPS13A,VPS36,VPS4B,WDR12,WDR62,WIPF1,XRN1	166
Cell Death and Survival	Apoptosis of tumor cell lines	3,4E-10		1,54	ACACA,ACO2,ACSL4,ADI1,AGA,AKR1B1,AKR1B10,BIRC6,CBX3,CDK1,CDK4,CDK6,CNN2,CSNK1A1,CTBP2,DNMT1,DTYMK,EEF2K,EIF2B5,EXOC2,FADD,FASN,FLNB,GLRX,GMDS,GOT2,GTSE1,HMMR,HMOX1,ITPR1,ITPR3,KANK2,KPNA2,MAT2A,MTDH,MTSS1,NAPA,NDC80,NFKB2,NQO1,OGT,OSBPL8,PAK1,PARG,PCBP2,PCK2,POLA1,PRKACB,PRKCA,PRPF19,PTPN1,RAB32,RABGGTA,RAF1,RPA1,RPA2,RRM1,RRM2,SDF4,SLC9A3R1,SNX1,SPP1,SQSTM1,STAT1,STAT3,STAT5B,TAGLN2,TLN1,TM9SF4,TNFAIP8,TPD52,TRIM28,UBE2I,VCP,VPS13A	75

Categories	Diseases or Functions Annotation	p-value	Predicted Activation State	Activation z-score	Molecules	# Molecules
Cancer,Organismal Injury and Abnormalities	Intraabdominal organ tumor	2,45E-14		1,568	AARSD1,ABCD4,ACAA2,ACADS,ACO1,ACO2,ACP2,ACSF2,ACSL3,ACSL4,ADD1,ADI1,AGA,AGFG1,AGL,AIP,AK3,AKR1B1,AKR1B10,AKR7A2,ALDH2,ALDH4A1,ALDH6A1,ALDH9A1,ANKRD13A,ANKRD46,ANXA4,AP3S2,APPL2,ARFGAP2,ARFGAP3,ARFGEF2,ARHGEF1,ARHGEF12,ARHGEF18,ARMC8,ASRGL1,ATIC,ATP2A2,ATP2B4,ATP6AP1,ATP6V1A,ATPAF2,ATXN10,BCAS1,BCAT2,BIRC6,C1QBP,CACYBP,CAMSAP2,CAND1,CANX,CARMIL1,CAVIN1,CBX3,CC2D1B,CCDC47,CCZ1/CCZ1B,CDC37L1,CDC42EP4,CDIPT,CDK1,CDK4,CDK6,CEP55,CNPY2,CRAT,CSNK1A1,CTBP2,CTTNBP2,CUL3,CWF19L1,CYB5R1,CYFIP1,DCTN6,DDB1,DDRGK1,DDX5,DGKZ,DGLUCY,DNAJC5,DNAJC9,DNMT1,DNPEP,DOCK1,DOCK5,DPY19L1,DPYSL2,DPYSL3,DTYMK,DYNC2H1,ECI2,EEF1B2,EEF2,EEF2K,EIF2B4,EIF2B5,ELOA,ELP1,ELP2,ELP3,EMC7,EMD,EML1,ERLIN2,EXOC2,EXOC4,EXTL3,FADD,FADS1,FAF2,FASN,FBXL15,FBXO7,FDFT1,FLII,FLNA,FLNB,FRMD4A,FUT8,GATM,GCDH,GCLC,GCLM,GDA,GFPT1,GHDC,GLRX,GLRX3,GLRX5,GMDS,GMPPB,GOT2,GPT2,GRB14,GSDMD,GSTZ1,GTF2B,GTSE1,HACD3,HAUS7,HDGFL3,HEBP2,HIKESHI,HM13,HMGCL,HMGCS1,HMMR,HMOX1,HMOX2,HNRNPUL1,HSPA4L,IDH2,IFT74,IKBIP,IMPA1,INPP1,INPPL1,IRGQ,ISCA2,ITPR1,ITPR3,ITPRID2,KANK1,KANK2,KATNB1,KPNA2,LAMTOR2,LARP7,LAYN,LETM1,LIMD1,LOXL3,LPCAT3,LPP,LRRC40,LRRC59,LTA4H,MARK2,MAT2A,MEGF10,MKLN1,MMUT,MON2,MPI,MPST,MTDH,MTSS1,NAA25,NAB2,NAPA,NCLN,NDUFAF7,NEK9,NFKB2,NLE1,NLN,NPTXR,NQO1,NRBP1,NRDC,NT5C3B,NUP54,OAT,OGT,OSBPL8,OXCT1,PAFAH1B1,PAK1,PAPSS1,PARG,PCBP2,PCK2,PDE4DIP,PDZRN3,PEPD,PFKM,PHLDA1,PHYH,PIGU,PIP4P2,PITRM1,PKN2,PLCB1,PMPCB,POLA1,POLD1,POLD3,POLDIP3,PPA2,PPAT,PPOX,PPP2R5D,PPP4R3A,PRDX2,PRKACB,PRKCA,PRPF19,PRRC2A,PRRX1,PTPN1,QSOX2,RAB32,RAB6A,RABGGTA,RAD51,RAF1,RAI14,RANGAP1,RAP2C,RASSF4,RBPJ,RCN2,RER1,RETSAT,RIOK3,RNASEH2B,RPA1,RPA2,RPL27,RPL3,RPL5,RPL6,RPL7A,RPN1,RRM1,RRM2,SAMD4B,SDF4,SEC14L1,SEC24A,SEC24D,SEC61A1,SERPINE2,SH3GLB1,SLC1A4,SLC9A3R1,SLC9A3R2,SNF8,SNX1,SNX17,SOAT1,SORBS3,SPP1,SQLE,SQSTM1,SRGAP3,SRXN1,STAM2,STAT1,STAT3,STAT5B,STRN4,STT3A,SUCLG2,TAGLN2,TAX1BP1,TCF12,TDRKH,TKFC,TLE3,TLN1,TM9SF3,TM9SF4,TMEM115,TMEM43,TNS3,TOM1L2,TPD52,TRAPPC4,TRIM2,TRIM28,TRIM3,TRIM47,TRIOBP,TRIP10,TRIP12,TRIP13,TRMT2A,TST,TTC21B,UBAP2L,UBE2C,UBE2O,UCKL1,USP24,USP47,USP8,USP9X,VCP,VCPIP1,VPS11,VPS13A,VPS36,VPS4B,VWA5A,WDR12,WDR45B,WDR62,WFS1,WIPF1,XRN1,ZMPSTE24	343
Cardiovascular Disease,Hematological Disease,Organismal Injury and Abnormalities	Anemia	0,00000288		1,576	ADD1,ALDH2,C1QBP,CDK4,CDK6,DNMT1,FADD,GCLC,GLRX5,HMOX1,IDH2,MMUT,NFKB2,NQO1,POLA1,POLD1,PPAT,PRDX2,PRKCA,RAD51,RAF1,RPL27,RPL5,RRM1,RRM2,STAT3,STAT5B,TLE3,TRIM28	29
Cancer,Gastrointestinal Disease,Hepatic System Disease,Organismal Injury and Abnormalities	Liver cancer	0,0000285		1,584	ACAA2,ACO1,ACO2,ACSL3,ACSL4,ADD1,AGFG1,AKR1B10,ALDH2,ALDH4A1,ALDH9A1,APPL2,ARFGEF2,ARHGEF12,ASRGL1,ATIC,ATP2A2,BIRC6,CACYBP,CARMIL1,CAVIN1,CBX3,CDC37L1,CDK1,CDK4,CDK6,CNPY2,CTTNBP2,DGLUCY,DNAJC9,DOCK1,DPYSL3,DYNC2H1,ECI2,EEF2,EEF2K,ERLIN2,EXOC2,FADS1,FASN,FBXL15,FLNA,FLNB,FRMD4A,GCLC,GPT2,GRB14,GSDMD,GSTZ1,GTF2B,GTSE1,HMGCS1,HMMR,HMOX1,HNRNPUL1,IDH2,IFT74,INPP1,IRGQ,ITPR1,ITPR3,ITPRID2,KANK1,KANK2,LAMTOR2,LETM1,LOXL3,LPP,LRRC59,MARK2,MAT2A,MMUT,MON2,MPI,MTSS1,NAA25,NAPA,NCLN,NQO1,NRDC,OGT,OSBPL8,OXCT1,PAK1,PARG,PCBP2,PCK2,PDE4DIP,PDZRN3,PHLDA1,PIP4P2,PITRM1,PKN2,PLCB1,POLA1,POLD1,POLD3,POLDIP3,PPA2,PPOX,PPP2R5D,PRRC2A,PTPN1,RAF1,RANGAP1,RBPJ,RCN2,RER1,RETSAT,RIOK3,RPL6,RPL7A,RPN1,RRM1,RRM2,SEC24A,SH3GLB1,SLC1A4,SOAT1,SPP1,SQLE,SQSTM1,SRXN1,STAM2,STAT1,STAT3,STAT5B,TAX1BP1,TCF12,TM9SF3,TM9SF4,TNS3,TPD52,TRAPPC4,TRIM2,TRIM28,TRIOBP,TRIP10,TRMT2A,TST,TTC21B,UBE2C,USP24,USP47,USP9X,VCP,VCPIP1,VPS13A,VPS36,VPS4B,WDR12,WDR62,WIPF1,XRN1	154
Gastrointestinal Disease,Hepatic System Disease,Organismal Injury and Abnormalities	Liver lesion	0,0000103		1,638	ACAA2,ACO1,ACO2,ACSL3,ACSL4,ADD1,AGA,AGFG1,AKR1B10,ALDH2,ALDH4A1,ALDH9A1,ANKRD46,APPL2,ARFGEF2,ARHGEF12,ARHGEF18,ASRGL1,ATIC,ATP2A2,BIRC6,CACYBP,CARMIL1,CAVIN1,CBX3,CDC37L1,CDK1,CDK4,CDK6,CNPY2,CTBP2,CTTNBP2,DDX5,DGLUCY,DNAJC9,DOCK1,DPY19L1,DPYSL3,DYNC2H1,ECI2,EEF2,EEF2K,ERLIN2,EXOC2,FADD,FADS1,FASN,FBXL15,FLII,FLNA,FLNB,FRMD4A,GCLC,GCLM,GOT2,GPT2,GRB14,GSDMD,GSTZ1,GTF2B,GTSE1,HDGFL3,HIKESHI,HMGCS1,HMMR,HMOX1,HNRNPUL1,IDH2,IFT74,INPP1,IRGQ,ITPR1,ITPR3,ITPRID2,KANK1,KANK2,KATNB1,LAMTOR2,LETM1,LOXL3,LPP,LRRC59,MARK2,MAT2A,MMUT,MON2,MPI,MTDH,MTSS1,NAA25,NAPA,NCLN,NQO1,NRDC,OGT,OSBPL8,OXCT1,PAFAH1B1,PAK1,PARG,PCBP2,PCK2,PDE4DIP,PDZRN3,PHLDA1,PHYH,PIP4P2,PITRM1,PKN2,PLCB1,POLA1,POLD1,POLD3,POLDIP3,PPA2,PPAT,PPOX,PPP2R5D,PPP4R3A,PRDX2,PRKACB,PRRC2A,PTPN1,RAB6A,RAD51,RAF1,RANGAP1,RBPJ,RCN2,RER1,RETSAT,RIOK3,RPL5,RPL6,RPL7A,RPN1,RRM1,RRM2,SAMD4B,SEC24A,SEC24D,SERPINE2,SH3GLB1,SLC1A4,SOAT1,SPP1,SQLE,SQSTM1,SRXN1,STAM2,STAT1,STAT3,STAT5B,TAX1BP1,TCF12,TM9SF3,TM9SF4,TNS3,TPD52,TRAPPC4,TRIM2,TRIM28,TRIOBP,TRIP10,TRMT2A,TST,TTC21B,UBE2C,UBE2O,USP24,USP47,USP9X,VCP,VCPIP1,VPS13A,VPS36,VPS4B,VWA5A,WDR12,WDR62,WIPF1,XRN1	182
Cancer,Organismal Injury and Abnormalities	Cancer	2,03E-20		1,652	AARSD1,ABCD4,ACAA2,ACACA,ACADS,ACO1,ACO2,ACP2,ACSF2,ACSL3,ACSL4,ADD1,ADI1,ADO,AGA,AGFG1,AGL,AIP,AK3,AKR1B1,AKR1B10,AKR7A2,ALDH2,ALDH4A1,ALDH6A1,ALDH9A1,ANAPC7,ANKRD13A,ANKRD46,ANXA4,AP3S2,APPL2,ARFGAP2,ARFGAP3,ARFGEF2,ARHGEF1,ARHGEF12,ARHGEF18,ARMC8,ARPIN/ARPIN-AP3S2,ASRGL1,ATIC,ATP2A2,ATP2B4,ATP6AP1,ATP6V1A,ATPAF2,ATXN10,BCAS1,BCAT2,BIRC6,C17orf49,C1QBP,CACYBP,CAMSAP2,CAND1,CANX,CARMIL1,CAVIN1,CBX3,CC2D1B,CCDC47,CCZ1/CCZ1B,CDC37L1,CDC42EP4,CDIPT,CDK1,CDK4,CDK6,CEP55,CERT1,CNEP1R1,CNN2,CNN3,CNPY2,CRAT,CSNK1A1,CSRP1,CTBP2,CTTNBP2,CUL3,CWF19L1,CYB5R1,Cyb5r3,CYFIP1,DCTN6,DDB1,DDRGK1,DDX5,DGKZ,DGLUCY,DNAJC5,DNAJC9,DNMT1,DNPEP,DOCK1,DOCK5,DPY19L1,DPYSL2,DPYSL3,DTYMK,DYNC2H1,ECI2,EEF1B2,EEF2,EEF2K,EIF2B4,EIF2B5,ELOA,ELP1,ELP2,ELP3,EMC7,EMD,EML1,ERLIN2,EXOC2,EXOC4,EXTL3,FADD,FADS1,FAF2,FASN,FBXL15,FBXO7,FDFT1,FLII,FLNA,FLNB,FRMD4A,FUT8,GABPA,GATM,GCDH,GCLC,GCLM,GDA,GFPT1,GHDC,GLRX,GLRX3,GLRX5,GMDS,GMPPB,GOT2,GPT2,GRB14,GSDMD,GSTZ1,GTF2B,GTSE1,HACD3,HAUS7,HDGFL3,HEBP2,HIKESHI,HM13,HMGCL,HMGCS1,HMMR,HMOX1,HMOX2,HNRNPUL1,HSPA4L,IDH2,IFT74,IKBIP,IMPA1,INPP1,INPPL1,IRGQ,ISCA2,ITPR1,ITPR3,ITPRID2,KANK1,KANK2,KATNB1,KPNA2,LAMTOR2,LAMTOR3,LARP7,LAYN,LETM1,LIMD1,LOXL3,LPCAT3,LPP,LRRC40,LRRC59,LTA4H,MARK2,MAT2A,MEGF10,MGST2,MKLN1,MMUT,MON2,MPI,MPST,MTDH,MTSS1,NAA25,NAB2,NAPA,NARS1,NAXE,NCLN,NDC80,NDUFAF7,NEK9,NFKB2,NFS1,NIT2,NLE1,NLN,NMD3,NPTXR,NQO1,NRBP1,NRDC,NT5C3B,NUP54,OAT,OGT,OSBPL8,OXCT1,PAFAH1B1,PAK1,PAPSS1,PARG,PCBP2,PCK2,PDE4DIP,PDZRN3,PEPD,PFKM,PGD,PHLDA1,PHYH,PIGU,PIP4P2,PITRM1,PKN2,PLCB1,PMPCB,POLA1,POLD1,POLD3,POLDIP3,PPA2,PPAT,PPOX,PPP2R5D,PPP4R3A,PRDX2,PRKACB,PRKCA,PRPF19,PRRC2A,PRRX1,PTPN1,QSOX2,RAB32,RAB6A,RABGGTA,RAD51,RAF1,RAI14,RANGAP1,RAP2C,RASSF4,RBPJ,RCN2,RER1,RETSAT,RIOK3,RNASEH2B,RPA1,RPA2,RPL13,RPL27,RPL3,RPL5,RPL6,RPL7A,RPN1,RPN2,RRM1,RRM2,SAMD4B,SDF4,SEC14L1,SEC24A,SEC24D,SEC61A1,SERPINE2,SFR1,SGTA,SH3GLB1,SLC1A4,SLC9A3R1,SLC9A3R2,SNF8,SNX1,SNX17,SOAT1,SORBS3,SPCS3,SPP1,SQLE,SQSTM1,SRGAP3,SRXN1,STAM2,STARD3NL,STAT1,STAT3,STAT5B,STRN4,STT3A,SUCLG2,TAGLN2,TAX1BP1,TCF12,TDRKH,TKFC,TLE3,TLN1,TM9SF3,TM9SF4,TMEM115,TMEM43,TNS3,TOM1L2,TPD52,TRAPPC4,TRIM2,TRIM28,TRIM3,TRIM47,TRIOBP,TRIP10,TRIP12,TRIP13,TRMT2A,TST,TTC21B,UBAP2L,UBE2C,UBE2I,UBE2O,UBR7,UCKL1,USP24,USP47,USP8,USP9X,VAMP7,VAT1,VCP,VCPIP1,VPS11,VPS13A,VPS36,VPS4B,VWA5A,WDR12,WDR45B,WDR62,WFS1,WIPF1,XRN1,ZMPSTE24	374
Cancer,Organismal Injury and Abnormalities	Abdominal cancer	2,61E-14		1,697	AARSD1,ABCD4,ACAA2,ACADS,ACO1,ACO2,ACP2,ACSF2,ACSL3,ACSL4,ADD1,ADI1,AGA,AGFG1,AGL,AIP,AK3,AKR1B1,AKR1B10,AKR7A2,ALDH2,ALDH4A1,ALDH6A1,ALDH9A1,ANKRD13A,ANXA4,AP3S2,APPL2,ARFGAP2,ARFGAP3,ARFGEF2,ARHGEF1,ARHGEF12,ARHGEF18,ARMC8,ASRGL1,ATIC,ATP2A2,ATP2B4,ATP6AP1,ATP6V1A,ATPAF2,ATXN10,BCAS1,BCAT2,BIRC6,C1QBP,CACYBP,CAMSAP2,CAND1,CANX,CARMIL1,CAVIN1,CBX3,CC2D1B,CCDC47,CCZ1/CCZ1B,CDC37L1,CDC42EP4,CDIPT,CDK1,CDK4,CDK6,CEP55,CNPY2,CRAT,CSNK1A1,CTBP2,CTTNBP2,CUL3,CWF19L1,CYB5R1,CYFIP1,DCTN6,DDB1,DDRGK1,DDX5,DGKZ,DGLUCY,DNAJC5,DNAJC9,DNMT1,DNPEP,DOCK1,DOCK5,DPY19L1,DPYSL2,DPYSL3,DTYMK,DYNC2H1,ECI2,EEF1B2,EEF2,EEF2K,EIF2B4,EIF2B5,ELOA,ELP1,ELP2,ELP3,EMC7,EMD,EML1,ERLIN2,EXOC2,EXOC4,EXTL3,FADD,FADS1,FAF2,FASN,FBXL15,FBXO7,FDFT1,FLII,FLNA,FLNB,FRMD4A,FUT8,GATM,GCDH,GCLC,GCLM,GDA,GFPT1,GHDC,GLRX,GLRX3,GLRX5,GMDS,GMPPB,GOT2,GPT2,GRB14,GSDMD,GSTZ1,GTF2B,GTSE1,HACD3,HAUS7,HDGFL3,HEBP2,HIKESHI,HM13,HMGCL,HMGCS1,HMMR,HMOX1,HMOX2,HNRNPUL1,HSPA4L,IDH2,IFT74,IKBIP,IMPA1,INPP1,INPPL1,IRGQ,ISCA2,ITPR1,ITPR3,ITPRID2,KANK1,KANK2,KATNB1,KPNA2,LAMTOR2,LARP7,LAYN,LETM1,LIMD1,LOXL3,LPCAT3,LPP,LRRC40,LRRC59,LTA4H,MARK2,MAT2A,MEGF10,MKLN1,MMUT,MON2,MPI,MPST,MTDH,MTSS1,NAA25,NAB2,NAPA,NCLN,NDUFAF7,NEK9,NFKB2,NLE1,NLN,NMD3,NPTXR,NQO1,NRBP1,NRDC,NT5C3B,NUP54,OAT,OGT,OSBPL8,OXCT1,PAFAH1B1,PAK1,PAPSS1,PARG,PCBP2,PCK2,PDE4DIP,PDZRN3,PEPD,PFKM,PHLDA1,PHYH,PIGU,PIP4P2,PITRM1,PKN2,PLCB1,PMPCB,POLA1,POLD1,POLD3,POLDIP3,PPA2,PPAT,PPOX,PPP2R5D,PPP4R3A,PRDX2,PRKACB,PRKCA,PRPF19,PRRC2A,PRRX1,PTPN1,QSOX2,RAB32,RABGGTA,RAD51,RAF1,RAI14,RANGAP1,RAP2C,RASSF4,RBPJ,RCN2,RER1,RETSAT,RIOK3,RNASEH2B,RPA1,RPA2,RPL27,RPL3,RPL5,RPL6,RPL7A,RPN1,RPN2,RRM1,RRM2,SAMD4B,SDF4,SEC14L1,SEC24A,SEC24D,SEC61A1,SERPINE2,SGTA,SH3GLB1,SLC1A4,SLC9A3R1,SLC9A3R2,SNF8,SNX1,SNX17,SOAT1,SORBS3,SPP1,SQLE,SQSTM1,SRGAP3,SRXN1,STAM2,STAT1,STAT3,STAT5B,STRN4,STT3A,SUCLG2,TAGLN2,TAX1BP1,TCF12,TDRKH,TKFC,TLE3,TLN1,TM9SF3,TM9SF4,TMEM115,TMEM43,TNS3,TOM1L2,TPD52,TRAPPC4,TRIM2,TRIM28,TRIM3,TRIM47,TRIOBP,TRIP10,TRIP12,TRIP13,TRMT2A,TST,TTC21B,UBAP2L,UBE2C,UBE2O,UCKL1,USP24,USP47,USP8,USP9X,VCP,VCPIP1,VPS11,VPS13A,VPS36,VPS4B,VWA5A,WDR12,WDR45B,WDR62,WFS1,WIPF1,XRN1,ZMPSTE24	344
Cancer,Gastrointestinal Disease,Hepatic System Disease,Organismal Injury and Abnormalities	Liver carcinoma	0,0000273		1,698	ACAA2,ACO1,ACO2,ACSL3,ACSL4,ADD1,AGFG1,AKR1B10,ALDH2,ALDH4A1,ALDH9A1,APPL2,ARFGEF2,ARHGEF12,ASRGL1,ATIC,ATP2A2,BIRC6,CACYBP,CARMIL1,CAVIN1,CBX3,CDC37L1,CDK1,CDK4,CDK6,CNPY2,CTTNBP2,DGLUCY,DNAJC9,DOCK1,DPYSL3,DYNC2H1,ECI2,EEF2,EEF2K,ERLIN2,EXOC2,FADS1,FASN,FBXL15,FLNA,FLNB,FRMD4A,GCLC,GPT2,GRB14,GSDMD,GSTZ1,GTF2B,GTSE1,HMGCS1,HMMR,HMOX1,HNRNPUL1,IDH2,IFT74,INPP1,IRGQ,ITPR1,ITPR3,ITPRID2,KANK1,KANK2,LAMTOR2,LOXL3,LPP,LRRC59,MARK2,MMUT,MON2,MPI,MTSS1,NAA25,NAPA,NCLN,NQO1,NRDC,OGT,OSBPL8,OXCT1,PAK1,PARG,PCBP2,PCK2,PDE4DIP,PDZRN3,PHLDA1,PIP4P2,PITRM1,PKN2,PLCB1,POLA1,POLD1,POLD3,POLDIP3,PPA2,PPOX,PPP2R5D,PRRC2A,PTPN1,RAF1,RANGAP1,RBPJ,RCN2,RER1,RETSAT,RIOK3,RPL6,RPL7A,RPN1,RRM1,RRM2,SEC24A,SH3GLB1,SLC1A4,SOAT1,SPP1,SQLE,SQSTM1,SRXN1,STAM2,STAT1,STAT3,STAT5B,TAX1BP1,TCF12,TM9SF3,TM9SF4,TNS3,TPD52,TRAPPC4,TRIM2,TRIOBP,TRIP10,TRMT2A,TST,TTC21B,UBE2C,USP24,USP47,VCP,VCPIP1,VPS13A,VPS36,VPS4B,WDR12,WDR62,WIPF1,XRN1	150
Cancer,Gastrointestinal Disease,Organismal Injury and Abnormalities	Hepatobiliary neoplasm	0,00000569		1,72	ACAA2,ACO1,ACO2,ACSL3,ACSL4,ADD1,AGA,AGFG1,AKR1B10,ALDH2,ALDH4A1,ALDH6A1,ALDH9A1,ANKRD46,APPL2,ARFGEF2,ARHGEF12,ARHGEF18,ASRGL1,ATIC,ATP2A2,BIRC6,CACYBP,CAND1,CANX,CARMIL1,CAVIN1,CBX3,CDC37L1,CDK1,CDK4,CDK6,CNPY2,CTBP2,CTTNBP2,DGLUCY,DNAJC9,DOCK1,DPY19L1,DPYSL3,DYNC2H1,ECI2,EEF2,EEF2K,ERLIN2,EXOC2,EXOC4,FADS1,FASN,FBXL15,FLII,FLNA,FLNB,FRMD4A,GCLC,GPT2,GRB14,GSDMD,GSTZ1,GTF2B,GTSE1,HDGFL3,HIKESHI,HMGCS1,HMMR,HMOX1,HNRNPUL1,IDH2,IFT74,INPP1,IRGQ,ITPR1,ITPR3,ITPRID2,KANK1,KANK2,KATNB1,KPNA2,LAMTOR2,LARP7,LETM1,LOXL3,LPP,LRRC59,MARK2,MAT2A,MMUT,MON2,MPI,MTDH,MTSS1,NAA25,NAPA,NCLN,NQO1,NRDC,OGT,OSBPL8,OXCT1,PAFAH1B1,PAK1,PARG,PCBP2,PCK2,PDE4DIP,PDZRN3,PHLDA1,PIGU,PIP4P2,PITRM1,PKN2,PLCB1,POLA1,POLD1,POLD3,POLDIP3,PPA2,PPOX,PPP2R5D,PRDX2,PRKACB,PRRC2A,PTPN1,RAB6A,RAD51,RAF1,RANGAP1,RBPJ,RCN2,RER1,RETSAT,RIOK3,RPL5,RPL6,RPL7A,RPN1,RPN2,RRM1,RRM2,SAMD4B,SEC24A,SEC24D,SERPINE2,SH3GLB1,SLC1A4,SOAT1,SPP1,SQLE,SQSTM1,SRXN1,STAM2,STAT1,STAT3,STAT5B,TAX1BP1,TCF12,TM9SF3,TM9SF4,TNS3,TPD52,TRAPPC4,TRIM2,TRIM28,TRIOBP,TRIP10,TRMT2A,TST,TTC21B,UBE2C,UBE2O,USP24,USP47,USP9X,VCP,VCPIP1,VPS13A,VPS36,VPS4B,VWA5A,WDR12,WDR62,WIPF1,XRN1	183
Cell Death and Survival	Cell death of tumor cell lines	1,91E-13		1,728	ACACA,ACO2,ACSL4,ADI1,AGA,AKR1B1,AKR1B10,ATP2A2,BIRC6,CBX3,CDK1,CDK4,CDK6,CNN2,CNPY2,CSNK1A1,CTBP2,CYB5R1,DNMT1,DOCK1,DTYMK,EEF2,EEF2K,EIF2B4,EIF2B5,EXOC2,FADD,FADS1,FASN,FDFT1,FLNB,GLRX,GMDS,GOT2,GTSE1,HMGCS1,HMMR,HMOX1,ITPR1,ITPR3,KANK2,KPNA2,LETM1,MAT2A,MPI,MTDH,MTSS1,NAPA,NDC80,NFKB2,NQO1,OGT,OSBPL8,PAK1,PARG,PCBP2,PCK2,POLA1,POLD1,PPAT,PRDX2,PRKACB,PRKCA,PRPF19,PTPN1,RAB32,RABGGTA,RAD51,RAF1,RNASEH2B,RPA1,RPA2,RRM1,RRM2,SDF4,SFR1,SH3GLB1,SLC9A3R1,SLC9A3R2,SNX1,SPP1,SQSTM1,STAT1,STAT3,STAT5B,TAGLN2,TLN1,TM9SF4,TNFAIP8,TPD52,TRIM28,UBE2C,UBE2I,USP47,VCP,VPS13A	96
Cancer,Gastrointestinal Disease,Hepatic System Disease,Organismal Injury and Abnormalities	Liver tumor	0,0000219		1,759	ACAA2,ACO1,ACO2,ACSL3,ACSL4,ADD1,AGFG1,AKR1B10,ALDH2,ALDH4A1,ALDH9A1,ANKRD46,APPL2,ARFGEF2,ARHGEF12,ARHGEF18,ASRGL1,ATIC,ATP2A2,BIRC6,CACYBP,CARMIL1,CAVIN1,CBX3,CDC37L1,CDK1,CDK4,CDK6,CNPY2,CTBP2,CTTNBP2,DGLUCY,DNAJC9,DOCK1,DPY19L1,DPYSL3,DYNC2H1,ECI2,EEF2,EEF2K,ERLIN2,EXOC2,FADS1,FASN,FBXL15,FLII,FLNA,FLNB,FRMD4A,GCLC,GPT2,GRB14,GSDMD,GSTZ1,GTF2B,GTSE1,HDGFL3,HIKESHI,HMGCS1,HMMR,HMOX1,HNRNPUL1,IDH2,IFT74,INPP1,IRGQ,ITPR1,ITPR3,ITPRID2,KANK1,KANK2,KATNB1,LAMTOR2,LETM1,LOXL3,LPP,LRRC59,MARK2,MAT2A,MMUT,MON2,MPI,MTDH,MTSS1,NAA25,NAPA,NCLN,NQO1,NRDC,OGT,OSBPL8,OXCT1,PAFAH1B1,PAK1,PARG,PCBP2,PCK2,PDE4DIP,PDZRN3,PHLDA1,PIP4P2,PITRM1,PKN2,PLCB1,POLA1,POLD1,POLD3,POLDIP3,PPA2,PPOX,PPP2R5D,PRDX2,PRKACB,PRRC2A,PTPN1,RAB6A,RAD51,RAF1,RANGAP1,RBPJ,RCN2,RER1,RETSAT,RIOK3,RPL5,RPL6,RPL7A,RPN1,RRM1,RRM2,SAMD4B,SEC24A,SEC24D,SERPINE2,SH3GLB1,SLC1A4,SOAT1,SPP1,SQLE,SQSTM1,SRXN1,STAM2,STAT1,STAT3,STAT5B,TAX1BP1,TCF12,TM9SF3,TM9SF4,TNS3,TPD52,TRAPPC4,TRIM2,TRIM28,TRIOBP,TRIP10,TRMT2A,TST,TTC21B,UBE2C,UBE2O,USP24,USP47,USP9X,VCP,VCPIP1,VPS13A,VPS36,VPS4B,VWA5A,WDR12,WDR62,WIPF1,XRN1	174
DNA Replication, Recombination, and Repair	DNA damage	0,00000214		1,919	ACO2,ALDH2,CDK1,DNMT1,GCLC,NQO1,OGT,POLD1,PRRX1,RAD51,RAF1,RNASEH2B,RPA1,RRM1,RRM2,SH3GLB1,SQSTM1,STAT3,USP9X,ZMPSTE24	20
Cellular Response to Therapeutics	Sensitivity of tumor cell lines	1,69E-11		1,985	ACSL4,AIP,ATIC,BIRC6,CANX,CDK4,CDK6,CUL3,DNMT1,ELOA,EMC7,HMMR,INPPL1,MPI,NFS1,OGT,POLA1,PRPF19,RER1,SEC14L1,TAGLN2,TOM1L2,TRIM28,TRIP10,TRIP12,UBAP2L,UBR7,USP24,VCP,VCPIP1,VPS11	31
Cellular Response to Therapeutics	Sensitivity of lung cancer cell lines	0,0000885	Increased	2,121	ATIC,INPPL1,MPI,OGT,POLA1,TAGLN2,TOM1L2,TRIP10	8
Cellular Response to Therapeutics	Sensitivity of carcinoma cell lines	0,00019	Increased	2,138	ATIC,INPPL1,MPI,OGT,POLA1,TAGLN2,TOM1L2,TRIP10	8
Developmental Disorder,Neurological Disease	Congenital neurological disorder	0,00000428	Increased	2,215	ARFGEF2,CCDC47,CEP55,CNN3,DNAJC5,DYNC2H1,EML1,EXOC4,EXTL3,FADD,FLNA,FRMD4A,GATM,GFPT1,GMPPB,HMOX1,IFT74,ITPR1,KATNB1,KIFBP,LARP7,NARS1,NCLN,NEK9,PAFAH1B1,PAK1,PHYH,POLA1,POLD1,PRKACB,PRKCA,PRRX1,RAD51,RAF1,RPL6,SLC1A4,SNF8,SNX1,SOAT1,STRN4,SUCLG2,TRAPPC4,TTC21B,VARS1,VCP,WDR62	46

Categories	Diseases or Functions Annotation	p-value	Predicted Activation State	Activation z-score	Molecules	# Molecules
Cancer,Cell Death and Survival,Organismal Injury and Abnormalities,Tumor Morphology	Cell death of cancer cells	0,0000261	Increased	2,237	CDK1,CDK6,EXOC2,FADD,FASN,HMOX1,IDH2,NAPA,NUP54,PHLDA1,RAF1,RBPJ,RPA2,RPL13,RPL27,RPL3,RPL5,RPL6,RPL7A,RRM2,STAT3,UBE2I,VCP	23
Developmental Disorder	Growth failure or short stature	0,00000293	Increased	2,285	ACACA,BIRC6,C1QBP,CAMSAP2,DDRGK1,DNAJC5,DYNC2H1,ELOA,ELP1,FADD,FDFT1,FLNA,FLNB,FUT8,GCLC,HMOX1,IFT74,INPPL1,ITPR1,ITPR3,LAMTOR2,LARP7,MARK2,MMUT,NFKB2,POLA1,PRRX1,RAF1,RBPJ,RNASEH2B,RPL13,SNX1,STAT1,STAT3,STAT5B,TCF12,TRIP12,TTC21B,USP8,ZMPSTE24	40
Developmental Disorder	Growth Failure	0,000098	Increased	2,285	ACACA,BIRC6,C1QBP,CAMSAP2,DNAJC5,ELOA,ELP1,FADD,FDFT1,FLNB,FUT8,GCLC,HMOX1,ITPR1,ITPR3,LAMTOR2,MARK2,MMUT,NFKB2,PRRX1,RAF1,RBPJ,RNASEH2B,RPL13,SNX1,STAT1,STAT3,TCF12,TRIP12,USP8,ZMPSTE24	31
Cellular Response to Therapeutics	Sensitivity of cells	7,86E-11	Increased	2,334	ACSL4,AIP,ATIC,BIRC6,CANX,CDK4,CDK6,CUL3,DNMT1,ELOA,EMC7,GCLM,GLRX,HMMR,INPPL1,MPI,NFS1,OGT,POLA1,PRDX2,PRPF19,RAD51,RER1,RO60,SEC14L1,TAGLN2,TOM1L2,TRIM28,TRIP10,TRIP12,UBAP2L,UBR7,USP24,VCP,VCPIP1,VPS11	36
Developmental Disorder,Hereditary Disorder,Organismal Injury and Abnormalities	Familial congenital malformation	6,67E-07	Increased	2,433	ACAA2,AIP,ARFGEF2,ATP6V1A,CAVIN1,CCDC47,CDK4,CDK6,CEP55,DDRGK1,DDX5,DNMT1,DPYSL3,DYNC2H1,ECI2,EML1,EXOC4,EXTL3,FADD,FDFT1,FLNA,FLNB,FRMD4A,GABPA,GFPT1,GMPPB,HMOX1,IFT74,INPPL1,ITPR1,KATNB1,KIFBP,LARP7,LETM1,LOXL3,MEGF10,NARS1,NCLN,NEK9,NFKB2,OXCT1,PAFAH1B1,PAK1,PCK2,PHYH,PITRM1,PLCB1,POLA1,POLD1,PRKACB,PRKCA,PRRX1,RAF1,RBPJ,RPL13,RPL6,RRM1,RRM2,SEC24D,SLC1A4,SPP1,SRGAP3,STRN4,TCF12,TMEM120A,TRAPPC4,TRIP12,TTC21B,VARS1,WDR62,WFS1,ZMPSTE24	72
Organismal Survival	Organismal death	1,56E-09	Increased	4,264	ACACA,ADD1,AGA,AGL,AIP,Anp32b,ARHGEF12,ATP2A2,ATP6AP1,BIRC6,C1QBP,CANX,CCDC47,CDK1,CDK4,CDK6,CERT1,CNN3,CNPY2,CSNK1A1,CSRP1,CUL3,DDB1,DDX5,DNAJC5,DNMT1,DOCK1,DYNC2H1,EEF2K,ELOA,ELP1,EMC7,EML1,EXTL3,FADD,FASN,FDFT1,FLII,FLNA,FLNB,FUT8,GABPA,GCLC,GCLM,GSTZ1,HMGCL,HMMR,HMOX1,HMOX2,HSPA4L,IDH2,IKBIP,IMPA1,ITPR1,ITPR3,LAMTOR2,LARP7,LOXL3,MARK2,MMUT,MPI,MTDH,MTSS1,NAB2,NDC80,NFKB2,NLE1,NQO1,NRBP1,NRDC,OAT,PAFAH1B1,PARG,PCBP2,PCK2,PDE4DIP,PFKM,PLCB1,POLD1,PRKACB,PRKCA,PRPF19,PRRX1,PTPN1,RAB6A,RAD51,RAF1,RBPJ,RNASEH2B,RO60,RPL5,RPL6,SGTA,SLC9A3R1,SNX1,SPP1,SQSTM1,SRGAP3,SRXN1,STAM2,STAT1,STAT3,STAT5B,TAX1BP1,TCF12,TLN1,TRIM28,TRIP12,TRIP13,UBE2C,UBE2I,USP8,VCP,WIPF1,ZMPSTE24	115
Cancer,Organismal Injury and Abnormalities	Neck neoplasm	1,4E-23			AARSD1,ABCD4,ACAA2,ACADS,ACO1,ACO2,ACP2,ACSF2,ACSL3,ACSL4,ADD1,ADI1,ADO,AGA,AGFG1,AGL,AIP,AK3,AKR1B1,AKR7A2,ALDH2,ALDH4A1,ALDH6A1,ALDH9A1,ANAPC7,ANKRD13A,APPL2,ARFGAP2,ARFGAP3,ARFGEF2,ARHGEF1,ARHGEF12,ARHGEF18,ARMC8,ASRGL1,ATIC,ATP2A2,ATP2B4,ATP6AP1,ATP6V1A,ATXN10,BCAS1,BCAT2,BIRC6,C17orf49,C1QBP,CAMSAP2,CAND1,CANX,CARMIL1,CAVIN1,CBX3,CC2D1B,CCDC47,CDC37L1,CDC42EP4,CDIPT,CDK1,CDK4,CDK6,CEP55,CNEP1R1,CNN2,CNN3,CRAT,CSNK1A1,CSRP1,CTBP2,CTTNBP2,CUL3,CWF19L1,CYB5R1,CYFIP1,DDB1,DDRGK1,DDX5,DGKZ,DGLUCY,DNAJC9,DNMT1,DNPEP,DOCK1,DOCK5,DPY19L1,DPYSL2,DPYSL3,DTYMK,DYNC2H1,EEF1B2,EEF2,EEF2K,EIF2B4,ELOA,ELP1,ELP2,ELP3,EMD,EML1,EXOC2,EXOC4,FADD,FADS1,FASN,FBXL15,FDFT1,FLII,FLNA,FLNB,FRMD4A,GABPA,GATM,GCDH,GCLM,GDA,GFPT1,GHDC,GLRX3,GLRX5,GMDS,GMPPB,GOT2,GPT2,GSDMD,GTF2B,GTSE1,HACD3,HDGFL3,HM13,HMGCL,HMGCS1,HMMR,HMOX1,HMOX2,HNRNPUL1,HSPA4L,IDH2,IFT74,INPPL1,IRGQ,ITPR1,ITPR3,ITPRID2,KANK1,KANK2,KATNB1,KPNA2,LAMTOR3,LARP7,LETM1,LIMD1,LOXL3,LPCAT3,LPP,LRRC40,LRRC59,MARK2,MAT2A,MEGF10,MGST2,MKLN1,MMUT,MON2,MPI,MPST,MTSS1,NAA25,NAB2,NAPA,NAXE,NCLN,NDC80,NDUFAF7,NEK9,NFKB2,NFS1,NIT2,NLE1,NLN,NPTXR,NQO1,NRBP1,NRDC,NUP54,OAT,OGT,OSBPL8,OXCT1,PAFAH1B1,PAK1,PAPSS1,PARG,PCBP2,PCK2,PDE4DIP,PDZRN3,PEPD,PFKM,PGD,PHLDA1,PHYH,PIGU,PITRM1,PKN2,PLCB1,PMPCB,POLA1,POLD1,POLD3,POLDIP3,PPA2,PPAT,PPOX,PPP2R5D,PPP4R3A,PRDX2,PRKACB,PRKCA,PRPF19,PRRC2A,PTPN1,QSOX2,RAB32,RAB6A,RABGGTA,RAD51,RAF1,RAI14,RANGAP1,RASSF4,RBPJ,RCN2,RETSAT,RIOK3,RNASEH2B,RPA1,RPA2,RPL13,RPL3,RPL6,RPL7A,RPN1,RPN2,RRM1,RRM2,SAMD4B,SEC14L1,SEC24A,SEC24D,SERPINE2,SFR1,SGTA,SH3GLB1,SLC1A4,SLC9A3R1,SLC9A3R2,SNF8,SNX1,SNX17,SOAT1,SORBS3,SPP1,SQLE,SQSTM1,SRGAP3,SRXN1,STAM2,STAT1,STAT3,STAT5B,STRN4,STT3A,SUCLG2,TAGLN2,TAX1BP1,TCF12,TKFC,TLE3,TLN1,TM9SF3,TM9SF4,TMEM115,TMEM43,TNS3,TOM1L2,TPD52,TRAPPC4,TRIM2,TRIM28,TRIM3,TRIM47,TRIOBP,TRIP10,TRIP12,TRIP13,TRMT2A,TST,TTC21B,UBAP2L,UBE2C,UBE2O,UBR7,UCKL1,USP24,USP47,USP8,USP9X,VAMP7,VAT1,VCP,VPS11,VPS13A,VWA5A,WDR12,WDR45B,WDR62,WFS1,WIPF1,XRN1,ZMPSTE24	319
Cancer,Endocrine System Disorders,Organismal Injury and Abnormalities	Thyroid gland tumor	1,67E-23			AARSD1,ABCD4,ACAA2,ACADS,ACO1,ACO2,ACP2,ACSF2,ACSL3,ACSL4,ADD1,ADI1,ADO,AGA,AGFG1,AGL,AIP,AK3,AKR1B1,AKR7A2,ALDH2,ALDH4A1,ALDH6A1,ALDH9A1,ANAPC7,ANKRD13A,APPL2,ARFGAP2,ARFGAP3,ARFGEF2,ARHGEF1,ARHGEF12,ARHGEF18,ARMC8,ASRGL1,ATIC,ATP2A2,ATP2B4,ATP6AP1,ATP6V1A,ATXN10,BCAS1,BCAT2,BIRC6,C17orf49,C1QBP,CAMSAP2,CAND1,CANX,CARMIL1,CAVIN1,CBX3,CC2D1B,CCDC47,CDC37L1,CDC42EP4,CDIPT,CDK1,CDK4,CDK6,CEP55,CNEP1R1,CNN2,CNN3,CRAT,CSNK1A1,CSRP1,CTBP2,CTTNBP2,CUL3,CWF19L1,CYB5R1,CYFIP1,DDB1,DDRGK1,DDX5,DGKZ,DGLUCY,DNAJC9,DNMT1,DNPEP,DOCK1,DOCK5,DPY19L1,DPYSL2,DPYSL3,DTYMK,DYNC2H1,EEF1B2,EEF2,EEF2K,EIF2B4,ELOA,ELP1,ELP2,ELP3,EMD,EML1,EXOC2,EXOC4,FADS1,FASN,FBXL15,FDFT1,FLII,FLNA,FLNB,FRMD4A,GABPA,GATM,GCDH,GCLM,GDA,GFPT1,GHDC,GLRX3,GLRX5,GMDS,GMPPB,GOT2,GPT2,GSDMD,GTSE1,HACD3,HDGFL3,HM13,HMGCL,HMGCS1,HMMR,HMOX1,HMOX2,HNRNPUL1,HSPA4L,IDH2,IFT74,INPPL1,IRGQ,ITPR1,ITPR3,ITPRID2,KANK1,KANK2,KATNB1,KPNA2,LAMTOR3,LARP7,LETM1,LIMD1,LOXL3,LPCAT3,LPP,LRRC40,LRRC59,MARK2,MAT2A,MEGF10,MGST2,MKLN1,MMUT,MON2,MPI,MPST,MTSS1,NAA25,NAB2,NAPA,NAXE,NCLN,NDC80,NDUFAF7,NEK9,NFKB2,NFS1,NIT2,NLE1,NLN,NPTXR,NQO1,NRBP1,NRDC,NUP54,OAT,OGT,OSBPL8,OXCT1,PAFAH1B1,PAK1,PAPSS1,PARG,PCBP2,PCK2,PDE4DIP,PDZRN3,PEPD,PFKM,PGD,PHLDA1,PHYH,PIGU,PITRM1,PKN2,PLCB1,PMPCB,POLA1,POLD1,POLD3,POLDIP3,PPA2,PPAT,PPOX,PPP2R5D,PPP4R3A,PRDX2,PRKACB,PRKCA,PRPF19,PRRC2A,PTPN1,QSOX2,RAB32,RAB6A,RABGGTA,RAD51,RAF1,RAI14,RANGAP1,RASSF4,RBPJ,RCN2,RETSAT,RIOK3,RNASEH2B,RPA1,RPA2,RPL13,RPL3,RPL6,RPL7A,RPN1,RPN2,RRM1,RRM2,SAMD4B,SEC14L1,SEC24A,SEC24D,SERPINE2,SFR1,SGTA,SH3GLB1,SLC1A4,SLC9A3R1,SLC9A3R2,SNF8,SNX1,SNX17,SOAT1,SORBS3,SPP1,SQLE,SQSTM1,SRGAP3,SRXN1,STAM2,STAT1,STAT3,STAT5B,STRN4,STT3A,SUCLG2,TAGLN2,TAX1BP1,TCF12,TKFC,TLE3,TLN1,TM9SF3,TM9SF4,TMEM115,TMEM43,TNS3,TOM1L2,TPD52,TRAPPC4,TRIM2,TRIM28,TRIM3,TRIM47,TRIOBP,TRIP10,TRIP12,TRIP13,TRMT2A,TST,TTC21B,UBAP2L,UBE2C,UBE2O,UBR7,UCKL1,USP24,USP47,USP8,USP9X,VAMP7,VAT1,VCP,VPS11,VPS13A,VWA5A,WDR12,WDR45B,WDR62,WFS1,WIPF1,XRN1,ZMPSTE24	317
Cancer,Endocrine System Disorders,Organismal Injury and Abnormalities	Thyroid carcinoma	6,08E-23			AARSD1,ACAA2,ACADS,ACO1,ACO2,ACP2,ACSF2,ACSL3,ACSL4,ADD1,ADI1,ADO,AGA,AGFG1,AGL,AIP,AK3,AKR1B1,AKR7A2,ALDH2,ALDH4A1,ALDH6A1,ALDH9A1,ANAPC7,ANKRD13A,APPL2,ARFGAP2,ARFGAP3,ARFGEF2,ARHGEF1,ARHGEF12,ARHGEF18,ARMC8,ASRGL1,ATIC,ATP2A2,ATP2B4,ATP6AP1,ATP6V1A,ATXN10,BCAS1,BCAT2,BIRC6,C17orf49,C1QBP,CAMSAP2,CAND1,CANX,CARMIL1,CAVIN1,CBX3,CC2D1B,CCDC47,CDC37L1,CDC42EP4,CDIPT,CDK1,CDK4,CDK6,CEP55,CNEP1R1,CNN2,CNN3,CRAT,CSNK1A1,CSRP1,CTBP2,CTTNBP2,CUL3,CWF19L1,CYB5R1,CYFIP1,DDB1,DDRGK1,DDX5,DGKZ,DGLUCY,DNAJC9,DNMT1,DNPEP,DOCK1,DOCK5,DPY19L1,DPYSL2,DPYSL3,DTYMK,DYNC2H1,EEF1B2,EEF2,EEF2K,EIF2B4,ELOA,ELP1,ELP2,ELP3,EMD,EML1,EXOC2,EXOC4,FADS1,FASN,FBXL15,FDFT1,FLII,FLNA,FLNB,FRMD4A,GABPA,GATM,GCDH,GCLM,GDA,GFPT1,GHDC,GLRX3,GLRX5,GMDS,GMPPB,GOT2,GPT2,GSDMD,GTSE1,HACD3,HDGFL3,HM13,HMGCL,HMGCS1,HMMR,HMOX1,HMOX2,HSPA4L,IDH2,IFT74,INPPL1,IRGQ,ITPR1,ITPR3,ITPRID2,KANK1,KANK2,KATNB1,KPNA2,LAMTOR3,LARP7,LETM1,LIMD1,LOXL3,LPCAT3,LPP,LRRC40,LRRC59,MARK2,MAT2A,MEGF10,MGST2,MKLN1,MMUT,MON2,MPI,MPST,MTSS1,NAA25,NAB2,NAPA,NAXE,NCLN,NDC80,NDUFAF7,NEK9,NFKB2,NFS1,NIT2,NLE1,NLN,NPTXR,NQO1,NRBP1,NRDC,NUP54,OAT,OGT,OSBPL8,OXCT1,PAFAH1B1,PAK1,PAPSS1,PARG,PCBP2,PCK2,PDE4DIP,PDZRN3,PEPD,PFKM,PGD,PHLDA1,PHYH,PIGU,PITRM1,PKN2,PLCB1,PMPCB,POLA1,POLD1,POLD3,POLDIP3,PPA2,PPAT,PPOX,PPP2R5D,PPP4R3A,PRDX2,PRKACB,PRKCA,PRPF19,PRRC2A,PTPN1,QSOX2,RAB32,RAB6A,RABGGTA,RAD51,RAF1,RAI14,RANGAP1,RASSF4,RBPJ,RCN2,RETSAT,RIOK3,RNASEH2B,RPA1,RPA2,RPL13,RPL3,RPL6,RPL7A,RPN1,RPN2,RRM1,RRM2,SAMD4B,SEC14L1,SEC24A,SEC24D,SERPINE2,SFR1,SGTA,SH3GLB1,SLC1A4,SLC9A3R1,SLC9A3R2,SNF8,SNX1,SNX17,SOAT1,SORBS3,SPP1,SQLE,SQSTM1,SRGAP3,SRXN1,STAM2,STAT1,STAT3,STAT5B,STRN4,STT3A,SUCLG2,TAGLN2,TAX1BP1,TCF12,TKFC,TLE3,TLN1,TM9SF3,TM9SF4,TMEM115,TMEM43,TNS3,TOM1L2,TPD52,TRAPPC4,TRIM2,TRIM28,TRIM3,TRIM47,TRIOBP,TRIP10,TRIP12,TRIP13,TRMT2A,TST,TTC21B,UBAP2L,UBE2C,UBE2O,UBR7,UCKL1,USP24,USP47,USP8,USP9X,VAMP7,VAT1,VCP,VPS11,VPS13A,VWA5A,WDR12,WDR45B,WDR62,WFS1,WIPF1,XRN1,ZMPSTE24	315
Cancer,Endocrine System Disorders,Organismal Injury and Abnormalities	Nonpituitary endocrine tumor	1,15E-22			AARSD1,ABCD4,ACAA2,ACADS,ACO1,ACO2,ACP2,ACSF2,ACSL3,ACSL4,ADD1,ADI1,ADO,AGA,AGFG1,AGL,AIP,AK3,AKR1B1,AKR7A2,ALDH2,ALDH4A1,ALDH6A1,ALDH9A1,ANAPC7,ANKRD13A,APPL2,ARFGAP2,ARFGAP3,ARFGEF2,ARHGEF1,ARHGEF12,ARHGEF18,ARMC8,ASRGL1,ATIC,ATP2A2,ATP2B4,ATP6AP1,ATP6V1A,ATXN10,BCAS1,BCAT2,BIRC6,C17orf49,C1QBP,CAMSAP2,CAND1,CANX,CARMIL1,CAVIN1,CBX3,CC2D1B,CCDC47,CDC37L1,CDC42EP4,CDIPT,CDK1,CDK4,CDK6,CEP55,CNEP1R1,CNN2,CNN3,CRAT,CSNK1A1,CSRP1,CTBP2,CTTNBP2,CUL3,CWF19L1,CYB5R1,CYFIP1,DDB1,DDRGK1,DDX5,DGKZ,DGLUCY,DNAJC9,DNMT1,DNPEP,DOCK1,DOCK5,DPY19L1,DPYSL2,DPYSL3,DTYMK,DYNC2H1,EEF1B2,EEF2,EEF2K,EIF2B4,ELOA,ELP1,ELP2,ELP3,EMD,EML1,EXOC2,EXOC4,FADS1,FASN,FBXL15,FDFT1,FLII,FLNA,FLNB,FRMD4A,GABPA,GATM,GCDH,GCLM,GDA,GFPT1,GHDC,GLRX3,GLRX5,GMDS,GMPPB,GOT2,GPT2,GSDMD,GTSE1,HACD3,HDGFL3,HM13,HMGCL,HMGCS1,HMMR,HMOX1,HMOX2,HNRNPUL1,HSPA4L,IDH2,IFT74,INPPL1,IRGQ,ITPR1,ITPR3,ITPRID2,KANK1,KANK2,KATNB1,KPNA2,LAMTOR3,LARP7,LETM1,LIMD1,LOXL3,LPCAT3,LPP,LRRC40,LRRC59,MARK2,MAT2A,MEGF10,MGST2,MKLN1,MMUT,MON2,MPI,MPST,MTDH,MTSS1,NAA25,NAB2,NAPA,NAXE,NCLN,NDC80,NDUFAF7,NEK9,NFKB2,NFS1,NIT2,NLE1,NLN,NPTXR,NQO1,NRBP1,NRDC,NUP54,OAT,OGT,OSBPL8,OXCT1,PAFAH1B1,PAK1,PAPSS1,PARG,PCBP2,PCK2,PDE4DIP,PDZRN3,PEPD,PFKM,PGD,PHLDA1,PHYH,PIGU,PITRM1,PKN2,PLCB1,PMPCB,POLA1,POLD1,POLD3,POLDIP3,PPA2,PPAT,PPOX,PPP2R5D,PPP4R3A,PRDX2,PRKACB,PRKCA,PRPF19,PRRC2A,PTPN1,QSOX2,RAB32,RAB6A,RABGGTA,RAD51,RAF1,RAI14,RANGAP1,RASSF4,RBPJ,RCN2,RETSAT,RIOK3,RNASEH2B,RPA1,RPA2,RPL13,RPL3,RPL6,RPL7A,RPN1,RPN2,RRM1,RRM2,SAMD4B,SEC14L1,SEC24A,SEC24D,SERPINE2,SFR1,SGTA,SH3GLB1,SLC1A4,SLC9A3R1,SLC9A3R2,SNF8,SNX1,SNX17,SOAT1,SORBS3,SPP1,SQLE,SQSTM1,SRGAP3,SRXN1,STAM2,STAT1,STAT3,STAT5B,STRN4,STT3A,SUCLG2,TAGLN2,TAX1BP1,TCF12,TKFC,TLE3,TLN1,TM9SF3,TM9SF4,TMEM115,TMEM43,TNS3,TOM1L2,TPD52,TRAPPC4,TRIM2,TRIM28,TRIM3,TRIM47,TRIOBP,TRIP10,TRIP12,TRIP13,TRMT2A,TST,TTC21B,UBAP2L,UBE2C,UBE2I,UBE2O,UBR7,UCKL1,USP24,USP47,USP8,USP9X,VAMP7,VAT1,VCP,VPS11,VPS13A,VWA5A,WDR12,WDR45B,WDR62,WFS1,WIPF1,XRN1,ZMPSTE24	319
Cancer,Endocrine System Disorders,Organismal Injury and Abnormalities	Endocrine carcinoma	1,88E-21			AARSD1,ABCD4,ACAA2,ACADS,ACO1,ACO2,ACP2,ACSF2,ACSL3,ACSL4,ADD1,ADI1,ADO,AGA,AGFG1,AGL,AIP,AK3,AKR1B1,AKR7A2,ALDH2,ALDH4A1,ALDH6A1,ALDH9A1,ANAPC7,ANKRD13A,APPL2,ARFGAP2,ARFGAP3,ARFGEF2,ARHGEF1,ARHGEF12,ARHGEF18,ARMC8,ASRGL1,ATIC,ATP2A2,ATP2B4,ATP6AP1,ATP6V1A,ATXN10,BCAS1,BCAT2,BIRC6,C17orf49,C1QBP,CAMSAP2,CAND1,CANX,CARMIL1,CAVIN1,CBX3,CC2D1B,CCDC47,CDC37L1,CDC42EP4,CDIPT,CDK1,CDK4,CDK6,CEP55,CNEP1R1,CNN2,CNN3,CRAT,CSNK1A1,CSRP1,CTBP2,CTTNBP2,CUL3,CWF19L1,CYB5R1,CYFIP1,DDB1,DDRGK1,DDX5,DGKZ,DGLUCY,DNAJC9,DNMT1,DNPEP,DOCK1,DOCK5,DPY19L1,DPYSL2,DPYSL3,DTYMK,DYNC2H1,EEF1B2,EEF2,EEF2K,EIF2B4,ELOA,ELP1,ELP2,ELP3,EMD,EML1,EXOC2,EXOC4,FADS1,FASN,FBXL15,FDFT1,FLII,FLNA,FLNB,FRMD4A,GABPA,GATM,GCDH,GCLM,GDA,GFPT1,GHDC,GLRX3,GLRX5,GMDS,GMPPB,GOT2,GPT2,GSDMD,GTSE1,HACD3,HDGFL3,HM13,HMGCL,HMGCS1,HMMR,HMOX1,HMOX2,HSPA4L,IDH2,IFT74,INPPL1,IRGQ,ITPR1,ITPR3,ITPRID2,KANK1,KANK2,KATNB1,KPNA2,LAMTOR3,LARP7,LETM1,LIMD1,LOXL3,LPCAT3,LPP,LRRC40,LRRC59,MARK2,MAT2A,MEGF10,MGST2,MKLN1,MMUT,MON2,MPI,MPST,MTDH,MTSS1,NAA25,NAB2,NAPA,NAXE,NCLN,NDC80,NDUFAF7,NEK9,NFKB2,NFS1,NIT2,NLE1,NLN,NPTXR,NQO1,NRBP1,NRDC,NUP54,OAT,OGT,OSBPL8,OXCT1,PAFAH1B1,PAK1,PAPSS1,PARG,PCBP2,PCK2,PDE4DIP,PDZRN3,PEPD,PFKM,PGD,PHLDA1,PHYH,PIGU,PITRM1,PKN2,PLCB1,PMPCB,POLA1,POLD1,POLD3,POLDIP3,PPA2,PPAT,PPOX,PPP2R5D,PPP4R3A,PRDX2,PRKACB,PRKCA,PRPF19,PRRC2A,PRRX1,PTPN1,QSOX2,RAB32,RAB6A,RABGGTA,RAD51,RAF1,RAI14,RANGAP1,RASSF4,RBPJ,RCN2,RER1,RETSAT,RIOK3,RNASEH2B,RPA1,RPA2,RPL13,RPL27,RPL3,RPL5,RPL6,RPL7A,RPN1,RPN2,RRM1,RRM2,SAMD4B,SEC14L1,SEC24A,SEC24D,SERPINE2,SFR1,SGTA,SH3GLB1,SLC1A4,SLC9A3R1,SLC9A3R2,SNF8,SNX1,SNX17,SOAT1,SORBS3,SPP1,SQLE,SQSTM1,SRGAP3,SRXN1,STAM2,STAT1,STAT3,STAT5B,STRN4,STT3A,SUCLG2,TAGLN2,TAX1BP1,TCF12,TKFC,TLE3,TLN1,TM9SF3,TM9SF4,TMEM115,TMEM43,TNS3,TOM1L2,TPD52,TRAPPC4,TRIM2,TRIM28,TRIM3,TRIM47,TRIOBP,TRIP10,TRIP12,TRIP13,TRMT2A,TST,TTC21B,UBAP2L,UBE2C,UBE2O,UBR7,UCKL1,USP24,USP47,USP8,USP9X,VAMP7,VAT1,VCP,VPS11,VPS13A,VWA5A,WDR12,WDR45B,WDR62,WFS1,WIPF1,XRN1,ZMPSTE24	321

Categories	Diseases or Functions Annotation	p-value	Predicted Activation State	Activation z-score	Molecules	# Molecules
Cancer,Endocrine System Disorders,Organismal Injury and Abnormalities	Endocrine gland tumor	5,24E-21			AARSD1,ABCD4,ACAA2,ACADS,ACO1,ACO2,ACP2,ACSF2,ACSL3,ACSL4,ADD1,ADI1,ADO,AGA,AGFG1,AGL,AIP,AK3,AKR1B1,AKR7A2,ALDH2,ALDH4A1,ALDH6A1,ALDH9A1,ANAPC7,ANKRD13A,APPL2,ARFGAP2,ARFGAP3,ARFGEF2,ARHGEF1,ARHGEF12,ARHGEF18,ARMC8,ASRGL1,ATIC,ATP2A2,ATP2B4,ATP6AP1,ATP6V1A,ATXN10,BCAS1,BCAT2,BIRC6,C17orf49,C1QBP,CAMSAP2,CAND1,CANX,CARMIL1,CAVIN1,CBX3,CC2D1B,CCDC47,CDC37L1,CDC42EP4,CDIPT,CDK1,CDK4,CDK6,CEP55,CNEP1R1,CNN2,CNN3,CRAT,CSNK1A1,CSRP1,CTBP2,CTTNBP2,CUL3,CWF19L1,CYB5R1,CYFIP1,DDB1,DDRGK1,DDX5,DGKZ,DGLUCY,DNAJC9,DNMT1,DNPEP,DOCK1,DOCK5,DPY19L1,DPYSL2,DPYSL3,DTYMK,DYNC2H1,EEF1B2,EEF2,EEF2K,EIF2B4,ELOA,ELP1,ELP2,ELP3,EMD,EML1,EXOC2,EXOC4,FADS1,FASN,FBXL15,FDFT1,FLII,FLNA,FLNB,FRMD4A,GABPA,GATM,GCDH,GCLM,GDA,GFPT1,GHDC,GLRX3,GLRX5,GMDS,GMPPB,GOT2,GPT2,GSDMD,GTSE1,HACD3,HDGFL3,HM13,HMGCL,HMGCS1,HMMR,HMOX1,HMOX2,HNRNPUL1,HSPA4L,IDH2,IFT74,INPPL1,IRGQ,ITPR1,ITPR3,ITPRID2,KANK1,KANK2,KATNB1,KPNA2,LAMTOR3,LARP7,LETM1,LIMD1,LOXL3,LPCAT3,LPP,LRRC40,LRRC59,MARK2,MAT2A,MEGF10,MGST2,MKLN1,MMUT,MON2,MPI,MPST,MTDH,MTSS1,NAA25,NAB2,NAPA,NAXE,NCLN,NDC80,NDUFAF7,NEK9,NFKB2,NFS1,NIT2,NLE1,NLN,NPTXR,NQO1,NRBP1,NRDC,NUP54,OAT,OGT,OSBPL8,OXCT1,PAFAH1B1,PAK1,PAPSS1,PARG,PCBP2,PCK2,PDE4DIP,PDZRN3,PEPD,PFKM,PGD,PHLDA1,PHYH,PIGU,PITRM1,PKN2,PLCB1,PMPCB,POLA1,POLD1,POLD3,POLDIP3,PPA2,PPAT,PPOX,PPP2R5D,PPP4R3A,PRDX2,PRKACB,PRKCA,PRPF19,PRRC2A,PRRX1,PTPN1,QSOX2,RAB32,RAB6A,RABGGTA,RAD51,RAF1,RAI14,RANGAP1,RASSF4,RBPJ,RCN2,RER1,RETSAT,RIOK3,RNASEH2B,RPA1,RPA2,RPL13,RPL27,RPL3,RPL5,RPL6,RPL7A,RPN1,RPN2,RRM1,RRM2,SAMD4B,SEC14L1,SEC24A,SEC24D,SERPINE2,SFR1,SGTA,SH3GLB1,SLC1A4,SLC9A3R1,SLC9A3R2,SNF8,SNX1,SNX17,SOAT1,SORBS3,SPP1,SQLE,SQSTM1,SRGAP3,SRXN1,STAM2,STAT1,STAT3,STAT5B,STRN4,STT3A,SUCLG2,TAGLN2,TAX1BP1,TCF12,TKFC,TLE3,TLN1,TM9SF3,TM9SF4,TMEM115,TMEM43,TNS3,TOM1L2,TPD52,TRAPPC4,TRIM2,TRIM28,TRIM3,TRIM47,TRIOBP,TRIP10,TRIP12,TRIP13,TRMT2A,TST,TTC21B,UBAP2L,UBE2C,UBE2I,UBE2O,UBR7,UCKL1,USP24,USP47,USP8,USP9X,VAMP7,VAT1,VCP,VPS11,VPS13A,VWA5A,WDR12,WDR45B,WDR62,WFS1,WIPF1,XRN1,ZMPSTE24	323
Cancer,Organismal Injury and Abnormalities	Cancer of secretory structure	3,12E-18			AARSD1,ABCD4,ACAA2,ACADS,ACO1,ACO2,ACP2,ACSF2,ACSL3,ACSL4,ADD1,ADI1,ADO,AGA,AGFG1,AGL,AIP,AK3,AKR1B1,AKR7A2,ALDH2,ALDH4A1,ALDH6A1,ALDH9A1,ANAPC7,ANKRD13A,APPL2,ARFGAP2,ARFGAP3,ARFGEF2,ARHGEF1,ARHGEF12,ARHGEF18,ARMC8,ASRGL1,ATIC,ATP2A2,ATP2B4,ATP6AP1,ATP6V1A,ATXN10,BCAS1,BCAT2,BIRC6,C17orf49,C1QBP,CAMSAP2,CAND1,CANX,CARMIL1,CAVIN1,CBX3,CC2D1B,CCDC47,CDC37L1,CDC42EP4,CDIPT,CDK1,CDK4,CDK6,CEP55,CNEP1R1,CNN2,CNN3,CRAT,CSNK1A1,CSRP1,CTBP2,CTTNBP2,CUL3,CWF19L1,CYB5R1,CYFIP1,DDB1,DDRGK1,DDX5,DGKZ,DGLUCY,DNAJC9,DNMT1,DNPEP,DOCK1,DOCK5,DPY19L1,DPYSL2,DPYSL3,DTYMK,DYNC2H1,EEF1B2,EEF2,EEF2K,EIF2B4,ELOA,ELP1,ELP2,ELP3,EMC7,EMD,EML1,EXOC2,EXOC4,EXTL3,FADS1,FASN,FBXL15,FDFT1,FLII,FLNA,FLNB,FRMD4A,GABPA,GATM,GCDH,GCLC,GCLM,GDA,GFPT1,GHDC,GLRX3,GLRX5,GMDS,GMPPB,GOT2,GPT2,GSDMD,GTSE1,HACD3,HDGFL3,HEBP2,HM13,HMGCL,HMGCS1,HMMR,HMOX1,HMOX2,HNRNPUL1,HSPA4L,IDH2,IFT74,INPPL1,IRGQ,ITPR1,ITPR3,ITPRID2,KANK1,KANK2,KATNB1,KPNA2,LAMTOR3,LARP7,LAYN,LETM1,LIMD1,LOXL3,LPCAT3,LPP,LRRC40,LRRC59,LTA4H,MARK2,MAT2A,MEGF10,MGST2,MKLN1,MMUT,MON2,MPI,MPST,MTSS1,NAA25,NAB2,NAPA,NAXE,NCLN,NDC80,NDUFAF7,NEK9,NFKB2,NFS1,NIT2,NLE1,NLN,NMD3,NPTXR,NQO1,NRBP1,NRDC,NUP54,OAT,OGT,OSBPL8,OXCT1,PAFAH1B1,PAK1,PAPSS1,PARG,PCBP2,PCK2,PDE4DIP,PDZRN3,PEPD,PFKM,PGD,PHLDA1,PHYH,PIGU,PIP4P2,PITRM1,PKN2,PLCB1,PMPCB,POLA1,POLD1,POLD3,POLDIP3,PPA2,PPAT,PPOX,PPP2R5D,PPP4R3A,PRDX2,PRKACB,PRKCA,PRPF19,PRRC2A,PTPN1,QSOX2,RAB32,RAB6A,RABGGTA,RAD51,RAF1,RAI14,RANGAP1,RASSF4,RBPJ,RCN2,RETSAT,RIOK3,RNASEH2B,RPA1,RPA2,RPL13,RPL3,RPL6,RPL7A,RPN1,RPN2,RRM1,RRM2,SAMD4B,SEC14L1,SEC24A,SEC24D,SERPINE2,SFR1,SGTA,SH3GLB1,SLC1A4,SLC9A3R1,SLC9A3R2,SNF8,SNX1,SNX17,SOAT1,SORBS3,SPP1,SQLE,SQSTM1,SRGAP3,SRXN1,STAM2,STAT1,STAT3,STAT5B,STRN4,STT3A,SUCLG2,TAGLN2,TAX1BP1,TCF12,TKFC,TLE3,TLN1,TM9SF3,TM9SF4,TMEM115,TMEM43,TNS3,TOM1L2,TPD52,TRAPPC4,TRIM2,TRIM28,TRIM3,TRIM47,TRIOBP,TRIP10,TRIP12,TRIP13,TRMT2A,TST,TTC21B,UBAP2L,UBE2C,UBE2O,UBR7,UCKL1,USP24,USP47,USP8,USP9X,VAMP7,VAT1,VCP,VPS11,VPS13A,VPS36,VWA5A,WDR12,WDR45B,WDR62,WFS1,WIPF1,XRN1,ZMPSTE24	326
Hereditary Disorder,Neurological Disease,Organismal Injury and Abnormalities	Familial neurological disorder	5E-14			ACO2,ACSL4,ALDH2,ALDH6A1,ARFGEF2,ARHGEF1,ATP2A2,ATP6V1A,ATXN10,CANX,CCDC47,CEP55,CERT1,CRAT,CUL3,CWF19L1,DNAJC5,DNMT1,DPYSL3,DYNC2H1,EEF1B2,EEF2,EIF2B4,EIF2B5,ELP1,ELP2,EML1,ERLIN2,EXOC4,EXTL3,FADD,FASN,FBXO7,FDFT1,FLNA,FRMD4A,GATM,GFPT1,GMPPB,GOT2,GPT2,HIKESHI,HMGCS1,HMOX1,IDH2,IFT74,IMPA1,INPP1,ITPR1,KANK1,KATNB1,KIFBP,LARP7,LOXL3,LPP,MTDH,NAPA,NARS1,NAXE,NCLN,NDUFAF7,NEK9,NFKB2,OGT,OSBPL8,PAFAH1B1,PAK1,PDE4DIP,PFKM,PHYH,PIGU,PITRM1,PLCB1,POLA1,POLD1,PPAT,PPP2R5D,PRDX2,PRKCA,RAB6A,RAD51,RAF1,RNASEH2B,RPL6,RRM1,RRM2,SEC24A,SERPINE2,SGTA,SLC1A4,SOAT1,SPP1,SQSTM1,STAT1,STRN4,SUCLG2,TDRKH,TPD52,TRAPPC4,TRIM2,TRIOBP,TRIP12,TTC21B,UBR7,USP8,USP9X,VARS1,VCP,VPS11,VPS13A,WDR45B,WDR62,WFS1	113
Cancer,Gastrointestinal Disease,Organismal Injury and Abnormalities	Gastrointestinal carcinoma	1,01E-13			AARSD1,ACAA2,ACO1,ACO2,ACP2,ACSF2,ACSL3,ACSL4,ADD1,AGA,AGFG1,AGL,AIP,AK3,AKR1B1,AKR1B10,AKR7A2,ALDH2,ALDH4A1,ALDH6A1,ALDH9A1,ANKRD13A,ANXA4,APPL2,ARFGAP2,ARFGAP3,ARFGEF2,ARHGEF1,ARHGEF12,ARHGEF18,ARMC8,ASRGL1,ATIC,ATP2A2,ATP2B4,ATP6AP1,ATP6V1A,ATXN10,BCAT2,BIRC6,C1QBP,CACYBP,CAMSAP2,CAND1,CANX,CARMIL1,CAVIN1,CBX3,CC2D1B,CCDC47,CDC37L1,CDC42EP4,CDIPT,CDK1,CDK4,CDK6,CEP55,CNPY2,CRAT,CSNK1A1,CTBP2,CTTNBP2,CWF19L1,CYB5R1,CYFIP1,DCTN6,DDB1,DDX5,DGKZ,DGLUCY,DNAJC5,DNAJC9,DNMT1,DNPEP,DOCK1,DOCK5,DPY19L1,DPYSL2,DPYSL3,DYNC2H1,EEF1B2,EEF2,EEF2K,EIF2B4,EIF2B5,ELOA,ELP1,ELP2,ELP3,EMC7,EMD,EML1,ERLIN2,EXOC2,EXOC4,EXTL3,FADS1,FAF2,FASN,FBXO7,FDFT1,FLII,FLNA,FLNB,FRMD4A,FUT8,GCDH,GCLC,GCLM,GDA,GFPT1,GHDC,GLRX5,GMDS,GMPPB,GOT2,GPT2,GRB14,GSDMD,GSTZ1,GTF2B,GTSE1,HAUS7,HDGFL3,HEBP2,HM13,HMGCL,HMMR,HMOX1,HMOX2,HNRNPUL1,HSPA4L,IDH2,IFT74,IKBIP,IMPA1,INPPL1,IRGQ,ISCA2,ITPR1,ITPR3,ITPRID2,KANK1,KANK2,KPNA2,LARP7,LAYN,LETM1,LIMD1,LOXL3,LPCAT3,LPP,LRRC59,LTA4H,MARK2,MEGF10,MKLN1,MMUT,MON2,MPI,MPST,MTDH,MTSS1,NAA25,NAB2,NCLN,NDUFAF7,NEK9,NFKB2,NLE1,NLN,NPTXR,NQO1,NRBP1,NRDC,NT5C3B,NUP54,OAT,OGT,OSBPL8,OXCT1,PAFAH1B1,PAK1,PAPSS1,PCBP2,PCK2,PDE4DIP,PDZRN3,PEPD,PFKM,PHLDA1,PHYH,PIGU,PIP4P2,PITRM1,PKN2,PLCB1,PMPCB,POLA1,POLD1,POLD3,POLDIP3,PPA2,PPOX,PPP2R5D,PPP4R3A,PRKACB,PRKCA,PRRC2A,PRRX1,PTPN1,QSOX2,RAB32,RAD51,RAF1,RAI14,RANGAP1,RAP2C,RASSF4,RBPJ,RETSAT,RIOK3,RNASEH2B,RPA1,RPA2,RPL27,RPL3,RPL5,RPN1,RRM1,RRM2,SAMD4B,SDF4,SEC14L1,SEC24A,SEC24D,SEC61A1,SERPINE2,SH3GLB1,SLC1A4,SLC9A3R2,SNF8,SNX1,SNX17,SOAT1,SORBS3,SPP1,SQLE,SQSTM1,SRGAP3,STAT1,STAT3,STAT5B,STRN4,STT3A,SUCLG2,TAX1BP1,TCF12,TDRKH,TKFC,TLE3,TLN1,TM9SF3,TM9SF4,TMEM115,TMEM43,TNS3,TOM1L2,TPD52,TRAPPC4,TRIM2,TRIM28,TRIM3,TRIM47,TRIOBP,TRIP10,TRIP12,TRIP13,TRMT2A,TST,TTC21B,UBAP2L,UBE2C,UBE2O,UBR7,UCKL1,USP24,USP47,USP8,USP9X,VAT1,VCP,VCPIP1,VPS11,VPS13A,VPS36,VPS4B,VWA5A,WDR12,WDR45B,WDR62,WFS1,WIPF1,XRN1,ZMPSTE24	305
Hereditary Disorder,Neurological Disease,Organismal Injury and Abnormalities	Autosomal recessive neurological disorder	1,19E-13			ACO2,ARFGEF2,CANX,CCDC47,CEP55,CRAT,CWF19L1,EEF1B2,EIF2B4,EIF2B5,ELP1,ELP2,EML1,ERLIN2,EXOC4,EXTL3,FADD,FASN,FBXO7,FLNA,FRMD4A,GATM,GFPT1,GMPPB,GOT2,GPT2,HIKESHI,IFT74,IMPA1,ITPR1,KATNB1,LARP7,NARS1,NAXE,NEK9,PHYH,PIGU,PITRM1,PLCB1,RNASEH2B,SLC1A4,SPP1,SQSTM1,STRN4,SUCLG2,TRAPPC4,TRIM2,TRIOBP,TTC21B,UBR7,VARS1,VCP,VPS11,VPS13A,WDR45B,WDR62,WFS1	57
Cancer,Gastrointestinal Disease,Organismal Injury and Abnormalities	Gastrointestinal adenocarcinoma	1,37E-13			AARSD1,ACAA2,ACO1,ACO2,ACP2,ACSF2,ACSL3,ACSL4,ADD1,AGA,AGFG1,AGL,AIP,AK3,AKR1B1,AKR1B10,AKR7A2,ALDH2,ALDH4A1,ALDH6A1,ALDH9A1,ANKRD13A,ANXA4,APPL2,ARFGAP2,ARFGAP3,ARFGEF2,ARHGEF1,ARHGEF12,ARHGEF18,ARMC8,ASRGL1,ATIC,ATP2A2,ATP2B4,ATP6AP1,ATXN10,BCAT2,BIRC6,C1QBP,CACYBP,CAMSAP2,CAND1,CANX,CARMIL1,CAVIN1,CBX3,CC2D1B,CCDC47,CDC37L1,CDC42EP4,CDIPT,CDK1,CDK4,CDK6,CEP55,CNPY2,CRAT,CSNK1A1,CTBP2,CTTNBP2,CWF19L1,CYB5R1,CYFIP1,DCTN6,DDB1,DDX5,DGKZ,DGLUCY,DNAJC5,DNMT1,DNPEP,DOCK1,DOCK5,DPY19L1,DPYSL2,DPYSL3,DYNC2H1,EEF1B2,EEF2,EEF2K,EIF2B5,ELOA,ELP1,ELP2,ELP3,EMC7,EML1,ERLIN2,EXOC2,EXOC4,EXTL3,FADS1,FAF2,FASN,FBXO7,FDFT1,FLII,FLNA,FLNB,FRMD4A,FUT8,GCDH,GCLC,GCLM,GDA,GFPT1,GHDC,GLRX5,GMDS,GMPPB,GOT2,GPT2,GRB14,GSDMD,GSTZ1,GTF2B,GTSE1,HAUS7,HDGFL3,HEBP2,HM13,HMGCL,HMMR,HMOX1,HMOX2,HNRNPUL1,HSPA4L,IDH2,IFT74,IKBIP,IMPA1,INPPL1,IRGQ,ISCA2,ITPR1,ITPR3,ITPRID2,KANK1,KANK2,KPNA2,LARP7,LAYN,LETM1,LIMD1,LOXL3,LPCAT3,LRRC59,LTA4H,MARK2,MEGF10,MKLN1,MMUT,MON2,MPI,MPST,MTDH,MTSS1,NAA25,NAB2,NCLN,NDUFAF7,NEK9,NFKB2,NLE1,NLN,NPTXR,NQO1,NRBP1,NRDC,NT5C3B,NUP54,OAT,OGT,OSBPL8,OXCT1,PAFAH1B1,PAK1,PCBP2,PCK2,PDE4DIP,PDZRN3,PEPD,PFKM,PHLDA1,PHYH,PIGU,PIP4P2,PITRM1,PKN2,PLCB1,PMPCB,POLA1,POLD1,POLD3,POLDIP3,PPA2,PPOX,PPP2R5D,PPP4R3A,PRKACB,PRKCA,PRRC2A,PRRX1,PTPN1,QSOX2,RAB32,RAD51,RAF1,RAI14,RANGAP1,RAP2C,RASSF4,RBPJ,RETSAT,RIOK3,RNASEH2B,RPA1,RPA2,RPL27,RPL3,RPL5,RPN1,RRM1,SAMD4B,SDF4,SEC14L1,SEC24A,SEC24D,SEC61A1,SERPINE2,SH3GLB1,SLC1A4,SLC9A3R2,SNF8,SNX1,SNX17,SOAT1,SORBS3,SPP1,SQLE,SQSTM1,SRGAP3,STAT1,STAT3,STAT5B,STRN4,STT3A,TAX1BP1,TCF12,TDRKH,TKFC,TLE3,TLN1,TM9SF3,TM9SF4,TMEM115,TMEM43,TNS3,TOM1L2,TRAPPC4,TRIM2,TRIM28,TRIM3,TRIM47,TRIOBP,TRIP10,TRIP12,TRIP13,TRMT2A,TST,TTC21B,UBAP2L,UBE2C,UBE2O,UCKL1,USP24,USP47,USP8,USP9X,VAT1,VCP,VCPIP1,VPS11,VPS13A,VPS36,VPS4B,VWA5A,WDR45B,WDR62,WFS1,WIPF1,XRN1,ZMPSTE24	294
Cancer,Gastrointestinal Disease,Organismal Injury and Abnormalities	Gastrointestinal tract cancer	5,29E-13			AARSD1,ACAA2,ACADS,ACO1,ACO2,ACP2,ACSF2,ACSL3,ACSL4,ADD1,AGA,AGFG1,AGL,AIP,AK3,AKR1B1,AKR1B10,AKR7A2,ALDH2,ALDH4A1,ALDH6A1,ALDH9A1,ANKRD13A,ANXA4,APPL2,ARFGAP2,ARFGAP3,ARFGEF2,ARHGEF1,ARHGEF12,ARHGEF18,ARMC8,ASRGL1,ATIC,ATP2A2,ATP2B4,ATP6AP1,ATP6V1A,ATXN10,BCAS1,BCAT2,BIRC6,C1QBP,CACYBP,CAMSAP2,CAND1,CANX,CARMIL1,CAVIN1,CBX3,CC2D1B,CCDC47,CDC37L1,CDC42EP4,CDIPT,CDK1,CDK4,CDK6,CEP55,CNPY2,CRAT,CSNK1A1,CTBP2,CTTNBP2,CWF19L1,CYB5R1,CYFIP1,DCTN6,DDB1,DDX5,DGKZ,DGLUCY,DNAJC5,DNAJC9,DNMT1,DNPEP,DOCK1,DOCK5,DPY19L1,DPYSL2,DPYSL3,DYNC2H1,EEF1B2,EEF2,EEF2K,EIF2B4,EIF2B5,ELOA,ELP1,ELP2,ELP3,EMC7,EMD,EML1,ERLIN2,EXOC2,EXOC4,EXTL3,FADS1,FAF2,FASN,FBXO7,FDFT1,FLII,FLNA,FLNB,FRMD4A,FUT8,GCDH,GCLC,GCLM,GDA,GFPT1,GHDC,GLRX,GLRX3,GLRX5,GMDS,GMPPB,GOT2,GPT2,GRB14,GSDMD,GSTZ1,GTF2B,GTSE1,HAUS7,HDGFL3,HEBP2,HM13,HMGCL,HMMR,HMOX1,HMOX2,HNRNPUL1,HSPA4L,IDH2,IFT74,IKBIP,IMPA1,INPPL1,IRGQ,ISCA2,ITPR1,ITPR3,ITPRID2,KANK1,KANK2,KPNA2,LARP7,LAYN,LETM1,LIMD1,LOXL3,LPCAT3,LPP,LRRC59,LTA4H,MARK2,MAT2A,MEGF10,MKLN1,MMUT,MON2,MPI,MPST,MTDH,MTSS1,NAA25,NAB2,NCLN,NDUFAF7,NEK9,NFKB2,NLE1,NLN,NPTXR,NQO1,NRBP1,NRDC,NT5C3B,NUP54,OAT,OGT,OSBPL8,OXCT1,PAFAH1B1,PAK1,PAPSS1,PCBP2,PCK2,PDE4DIP,PDZRN3,PEPD,PFKM,PHLDA1,PHYH,PIGU,PIP4P2,PITRM1,PKN2,PLCB1,PMPCB,POLA1,POLD1,POLD3,POLDIP3,PPA2,PPOX,PPP2R5D,PPP4R3A,PRKACB,PRKCA,PRRC2A,PRRX1,PTPN1,QSOX2,RAB32,RAD51,RAF1,RAI14,RANGAP1,RAP2C,RASSF4,RBPJ,RETSAT,RIOK3,RNASEH2B,RPA1,RPA2,RPL27,RPL3,RPL5,RPN1,RRM1,RRM2,SAMD4B,SDF4,SEC14L1,SEC24A,SEC24D,SEC61A1,SERPINE2,SH3GLB1,SLC1A4,SLC9A3R2,SNF8,SNX1,SNX17,SOAT1,SORBS3,SPP1,SQLE,SQSTM1,SRGAP3,STAT1,STAT3,STAT5B,STRN4,STT3A,SUCLG2,TAGLN2,TAX1BP1,TCF12,TDRKH,TKFC,TLE3,TLN1,TM9SF3,TM9SF4,TMEM115,TMEM43,TNS3,TOM1L2,TPD52,TRAPPC4,TRIM2,TRIM28,TRIM3,TRIM47,TRIOBP,TRIP10,TRIP12,TRIP13,TRMT2A,TST,TTC21B,UBAP2L,UBE2C,UBE2O,UBR7,UCKL1,USP24,USP47,USP8,USP9X,VAT1,VCP,VCPIP1,VPS11,VPS13A,VPS36,VPS4B,VWA5A,WDR12,WDR45B,WDR62,WFS1,WIPF1,XRN1,ZMPSTE24	311
Cancer,Gastrointestinal Disease,Organismal Injury and Abnormalities	Large intestine adenocarcinoma	9,84E-13			AARSD1,ACAA2,ACO1,ACO2,ACP2,ACSL4,ADD1,AGA,AGFG1,AGL,AIP,AK3,AKR1B1,AKR1B10,AKR7A2,ALDH2,ALDH4A1,ALDH6A1,ALDH9A1,ANKRD13A,ANXA4,APPL2,ARFGAP2,ARFGAP3,ARFGEF2,ARHGEF1,ARHGEF12,ARHGEF18,ARMC8,ASRGL1,ATIC,ATP2A2,ATP2B4,ATP6AP1,ATXN10,BCAT2,BIRC6,C1QBP,CACYBP,CAMSAP2,CAND1,CANX,CARMIL1,CAVIN1,CBX3,CC2D1B,CCDC47,CDC37L1,CDC42EP4,CDIPT,CDK1,CDK4,CDK6,CEP55,CNPY2,CRAT,CSNK1A1,CTBP2,CTTNBP2,CWF19L1,CYB5R1,CYFIP1,DCTN6,DDB1,DDX5,DGKZ,DGLUCY,DNAJC5,DNMT1,DNPEP,DOCK1,DOCK5,DPY19L1,DPYSL2,DPYSL3,DYNC2H1,EEF1B2,EEF2,EEF2K,EIF2B5,ELOA,ELP1,ELP2,ELP3,EMC7,ERLIN2,EXOC2,EXOC4,EXTL3,FADS1,FAF2,FASN,FBXO7,FDFT1,FLII,FLNA,FLNB,FRMD4A,FUT8,GCDH,GCLC,GCLM,GDA,GFPT1,GHDC,GLRX5,GMDS,GMPPB,GOT2,GPT2,GRB14,GSDMD,GSTZ1,GTF2B,GTSE1,HAUS7,HDGFL3,HEBP2,HM13,HMGCL,HMMR,HMOX1,HMOX2,HNRNPUL1,HSPA4L,IDH2,IFT74,IKBIP,IMPA1,INPPL1,IRGQ,ISCA2,ITPR1,ITPR3,ITPRID2,KANK1,KANK2,KPNA2,LARP7,LAYN,LETM1,LIMD1,LOXL3,LPCAT3,LRRC59,LTA4H,MARK2,MEGF10,MKLN1,MMUT,MON2,MPI,MPST,MTDH,MTSS1,NAA25,NAB2,NCLN,NDUFAF7,NEK9,NFKB2,NLE1,NLN,NPTXR,NRBP1,NRDC,NT5C3B,NUP54,OAT,OGT,OSBPL8,PAFAH1B1,PAK1,PCBP2,PDE4DIP,PDZRN3,PEPD,PFKM,PHLDA1,PHYH,PIGU,PIP4P2,PITRM1,PKN2,PLCB1,PMPCB,POLA1,POLD1,POLD3,POLDIP3,PPA2,PPOX,PPP2R5D,PPP4R3A,PRKACB,PRKCA,PRRC2A,PRRX1,PTPN1,QSOX2,RAB32,RAF1,RAI14,RANGAP1,RAP2C,RASSF4,RBPJ,RETSAT,RIOK3,RNASEH2B,RPA1,RPA2,RPL27,RPL3,RPL5,RPN1,RRM1,SAMD4B,SDF4,SEC14L1,SEC24A,SEC24D,SEC61A1,SERPINE2,SH3GLB1,SLC1A4,SLC9A3R2,SNF8,SNX1,SNX17,SOAT1,SORBS3,SQLE,SQSTM1,SRGAP3,STAT1,STAT3,STAT5B,STRN4,TAX1BP1,TCF12,TDRKH,TKFC,TLE3,TLN1,TM9SF3,TM9SF4,TMEM115,TMEM43,TNS3,TOM1L2,TRAPPC4,TRIM2,TRIM28,TRIM3,TRIM47,TRIOBP,TRIP10,TRIP12,TRIP13,TRMT2A,TST,TTC21B,UBAP2L,UBE2C,UBE2O,UCKL1,USP24,USP47,USP8,USP9X,VCP,VCPIP1,VPS11,VPS13A,VPS36,VPS4B,VWA5A,WDR45B,WDR62,WFS1,WIPF1,XRN1,ZMPSTE24	284

Categories	Diseases or Functions Annotation	p-value	Predicted Activation State	Activation z-score	Molecules	# Molecules
Cancer,Gastrointestinal Disease,Organismal Injury and Abnormalities	Malignant neoplasm of large intestine	1,02E-12			AARSD1,ACAA2,ACADS,ACO1,ACO2,ACP2,ACSL4,ADD1,AGA,AGFG1,AGL,AIP,AK3,AKR1B1,AKR1B10,AKR7A2,ALDH2,ALDH4A1,ALDH6A1,ALDH9A1,ANKRD13A,ANXA4,APPL2,ARFGAP2,ARFGAP3,ARFGEF2,ARHGEF1,ARHGEF12,ARHGEF18,ARMC8,ASRGL1,ATIC,ATP2A2,ATP2B4,ATP6AP1,ATXN10,BCAS1,BCAT2,BIRC6,C1QBP,CACYBP,CAMSAP2,CAND1,CANX,CARMIL1,CAVIN1,CBX3,CC2D1B,CCDC47,CDC37L1,CDC42EP4,CDIPT,CDK1,CDK4,CDK6,CEP55,CNPY2,CRAT,CSNK1A1,CTBP2,CTTNBP2,CWF19L1,CYB5R1,CYFIP1,DCTN6,DDB1,DDX5,DGKZ,DGLUCY,DNAJC5,DNMT1,DNPEP,DOCK1,DOCK5,DPY19L1,DPYSL2,DPYSL3,DYNC2H1,EEF1B2,EEF2,EEF2K,EIF2B5,ELOA,ELP1,ELP2,ELP3,EMC7,ERLIN2,EXOC2,EXOC4,EXTL3,FADS1,FAF2,FASN,FBXO7,FDFT1,FLII,FLNA,FLNB,FRMD4A,FUT8,GCDH,GCLC,GCLM,GDA,GFPT1,GHDC,GLRX,GLRX3,GLRX5,GMDS,GMPPB,GOT2,GPT2,GRB14,GSDMD,GSTZ1,GTF2B,GTSE1,HAUS7,HDGFL3,HEBP2,HM13,HMGCL,HMMR,HMOX1,HMOX2,HNRNPUL1,HSPA4L,IDH2,IFT74,IKBIP,IMPA1,INPPL1,IRGQ,ISCA2,ITPR1,ITPR3,ITPRID2,KANK1,KANK2,KPNA2,LARP7,LAYN,LETM1,LIMD1,LOXL3,LPCAT3,LRRC59,LTA4H,MARK2,MAT2A,MEGF10,MKLN1,MMUT,MON2,MPI,MPST,MTDH,MTSS1,NAA25,NAB2,NCLN,NDUFAF7,NEK9,NFKB2,NLE1,NLN,NPTXR,NQO1,NRBP1,NRDC,NT5C3B,NUP54,OAT,OGT,OSBPL8,PAFAH1B1,PAK1,PAPSS1,PCBP2,PDE4DIP,PDZRN3,PEPD,PFKM,PHLDA1,PHYH,PIGU,PIP4P2,PITRM1,PKN2,PLCB1,PMPCB,POLA1,POLD1,POLD3,POLDIP3,PPA2,PPOX,PPP2R5D,PPP4R3A,PRKACB,PRKCA,PRRC2A,PRRX1,PTPN1,QSOX2,RAB32,RAD51,RAF1,RAI14,RANGAP1,RAP2C,RASSF4,RBPJ,RETSAT,RIOK3,RNASEH2B,RPA1,RPA2,RPL27,RPL3,RPL5,RPN1,RRM1,RRM2,SAMD4B,SDF4,SEC14L1,SEC24A,SEC24D,SEC61A1,SERPINE2,SH3GLB1,SLC1A4,SLC9A3R2,SNF8,SNX1,SNX17,SOAT1,SORBS3,SPP1,SQLE,SQSTM1,SRGAP3,STAT1,STAT3,STAT5B,STRN4,TAGLN2,TAX1BP1,TCF12,TDRKH,TKFC,TLE3,TLN1,TM9SF3,TM9SF4,TMEM115,TMEM43,TNS3,TOM1L2,TRAPPC4,TRIM2,TRIM28,TRIM3,TRIM47,TRIOBP,TRIP10,TRIP12,TRIP13,TRMT2A,TST,TTC21B,UBAP2L,UBE2C,UBE2O,UCKL1,USP24,USP47,USP8,USP9X,VCP,VCPIP1,VPS11,VPS13A,VPS36,VPS4B,VWA5A,WDR45B,WDR62,WFS1,WIPF1,XRN1,ZMPSTE24	295
Cancer,Gastrointestinal Disease,Organismal Injury and Abnormalities	Large intestine carcinoma	1,73E-12			AARSD1,ACAA2,ACO1,ACO2,ACP2,ACSL4,ADD1,AGA,AGFG1,AGL,AIP,AK3,AKR1B1,AKR1B10,AKR7A2,ALDH2,ALDH4A1,ALDH6A1,ALDH9A1,ANKRD13A,ANXA4,APPL2,ARFGAP2,ARFGAP3,ARFGEF2,ARHGEF1,ARHGEF12,ARHGEF18,ARMC8,ASRGL1,ATIC,ATP2A2,ATP2B4,ATP6AP1,ATXN10,BCAT2,BIRC6,C1QBP,CACYBP,CAMSAP2,CAND1,CANX,CARMIL1,CAVIN1,CBX3,CC2D1B,CCDC47,CDC37L1,CDC42EP4,CDIPT,CDK1,CDK4,CDK6,CEP55,CNPY2,CRAT,CSNK1A1,CTBP2,CTTNBP2,CWF19L1,CYB5R1,CYFIP1,DCTN6,DDB1,DDX5,DGKZ,DGLUCY,DNAJC5,DNMT1,DNPEP,DOCK1,DOCK5,DPY19L1,DPYSL2,DPYSL3,DYNC2H1,EEF1B2,EEF2,EEF2K,EIF2B5,ELOA,ELP1,ELP2,ELP3,EMC7,ERLIN2,EXOC2,EXOC4,EXTL3,FADS1,FAF2,FASN,FBXO7,FDFT1,FLII,FLNA,FLNB,FRMD4A,FUT8,GCDH,GCLC,GCLM,GDA,GFPT1,GHDC,GLRX5,GMDS,GMPPB,GOT2,GPT2,GRB14,GSDMD,GSTZ1,GTF2B,GTSE1,HAUS7,HDGFL3,HEBP2,HM13,HMGCL,HMMR,HMOX1,HMOX2,HNRNPUL1,HSPA4L,IDH2,IFT74,IKBIP,IMPA1,INPPL1,IRGQ,ISCA2,ITPR1,ITPR3,ITPRID2,KANK1,KANK2,KPNA2,LARP7,LAYN,LETM1,LIMD1,LOXL3,LPCAT3,LRRC59,LTA4H,MARK2,MEGF10,MKLN1,MMUT,MON2,MPI,MPST,MTDH,MTSS1,NAA25,NAB2,NCLN,NDUFAF7,NEK9,NFKB2,NLE1,NLN,NPTXR,NQO1,NRBP1,NRDC,NT5C3B,NUP54,OAT,OGT,OSBPL8,PAFAH1B1,PAK1,PCBP2,PDE4DIP,PDZRN3,PEPD,PFKM,PHLDA1,PHYH,PIGU,PIP4P2,PITRM1,PKN2,PLCB1,PMPCB,POLA1,POLD1,POLD3,POLDIP3,PPA2,PPOX,PPP2R5D,PPP4R3A,PRKACB,PRKCA,PRRC2A,PRRX1,PTPN1,QSOX2,RAB32,RAF1,RAI14,RANGAP1,RAP2C,RASSF4,RBPJ,RETSAT,RIOK3,RNASEH2B,RPA1,RPA2,RPL27,RPL3,RPL5,RPN1,RRM1,SAMD4B,SDF4,SEC14L1,SEC24A,SEC24D,SEC61A1,SERPINE2,SH3GLB1,SLC1A4,SLC9A3R2,SNF8,SNX1,SNX17,SOAT1,SORBS3,SQLE,SQSTM1,SRGAP3,STAT1,STAT3,STAT5B,STRN4,TAX1BP1,TCF12,TDRKH,TKFC,TLE3,TLN1,TM9SF3,TM9SF4,TMEM115,TMEM43,TNS3,TOM1L2,TRAPPC4,TRIM2,TRIM28,TRIM3,TRIM47,TRIOBP,TRIP10,TRIP12,TRIP13,TRMT2A,TST,TTC21B,UBAP2L,UBE2C,UBE2O,UCKL1,USP24,USP47,USP8,USP9X,VCP,VCPIP1,VPS11,VPS13A,VPS36,VPS4B,VWA5A,WDR45B,WDR62,WFS1,WIPF1,XRN1,ZMPSTE24	285
Hereditary Disorder,Neurological Disease,Organismal Injury and Abnormalities	Familial encephalopathy	2,3E-12			ACO2,ACSL4,ALDH2,ALDH6A1,ARFGEF2,ARHGEF1,ATP2A2,ATP6V1A,ATXN10,CEP55,CERT1,CRAT,CUL3,CWF19L1,DNMT1,DPYSL3,EEF1B2,EEF2,EIF2B4,EIF2B5,ELP2,EML1,ERLIN2,FADD,FASN,FBXO7,FDFT1,FLNA,FRMD4A,GATM,GMPPB,GOT2,GPT2,HIKESHI,HMGCS1,HMOX1,IFT74,IMPA1,INPP1,ITPR1,KANK1,KATNB1,KIFBP,LARP7,LOXL3,LPP,MTDH,NAPA,NARS1,NAXE,NDUFAF7,NFKB2,OGT,OSBPL8,PAFAH1B1,PAK1,PDE4DIP,PFKM,PHYH,PIGU,PITRM1,PLCB1,POLA1,PPAT,PPP2R5D,PRDX2,PRKCA,RAB6A,RAF1,RNASEH2B,SEC24A,SERPINE2,SGTA,SLC1A4,SOAT1,SQSTM1,STAT1,STRN4,SUCLG2,TPD52,TRAPPC4,TRIP12,TTC21B,UBR7,USP8,USP9X,VARS1,VCP,VPS11,VPS13A,WDR45B,WDR62	92
Cancer,Organismal Injury and Abnormalities	Abdominal adenocarcinoma	4,9E-12			AARSD1,ABCD4,ACAA2,ACADS,ACO1,ACO2,ACP2,ACSF2,ACSL3,ACSL4,ADD1,AGA,AGFG1,AGL,AIP,AK3,AKR1B1,AKR1B10,AKR7A2,ALDH2,ALDH4A1,ALDH6A1,ALDH9A1,ANKRD13A,ANXA4,AP3S2,APPL2,ARFGAP2,ARFGAP3,ARFGEF2,ARHGEF1,ARHGEF12,ARHGEF18,ARMC8,ASRGL1,ATIC,ATP2A2,ATP2B4,ATP6AP1,ATXN10,BCAT2,BIRC6,C1QBP,CACYBP,CAMSAP2,CAND1,CANX,CARMIL1,CAVIN1,CBX3,CC2D1B,CCDC47,CDC37L1,CDC42EP4,CDIPT,CDK1,CDK4,CDK6,CEP55,CNPY2,CRAT,CSNK1A1,CTBP2,CTTNBP2,CUL3,CWF19L1,CYB5R1,CYFIP1,DCTN6,DDB1,DDRGK1,DDX5,DGKZ,DGLUCY,DNAJC5,DNMT1,DNPEP,DOCK1,DOCK5,DPY19L1,DPYSL2,DPYSL3,DTYMK,DYNC2H1,EEF1B2,EEF2,EEF2K,EIF2B5,ELOA,ELP1,ELP2,ELP3,EMC7,EMD,EML1,ERLIN2,EXOC2,EXOC4,EXTL3,FADS1,FAF2,FASN,FBXO7,FDFT1,FLII,FLNA,FLNB,FRMD4A,FUT8,GATM,GCDH,GCLC,GCLM,GDA,GFPT1,GHDC,GLRX3,GLRX5,GMDS,GMPPB,GOT2,GPT2,GRB14,GSDMD,GSTZ1,GTF2B,GTSE1,HACD3,HAUS7,HDGFL3,HEBP2,HM13,HMGCL,HMGCS1,HMMR,HMOX1,HMOX2,HNRNPUL1,HSPA4L,IDH2,IFT74,IKBIP,IMPA1,INPPL1,IRGQ,ISCA2,ITPR1,ITPR3,ITPRID2,KANK1,KANK2,KPNA2,LARP7,LAYN,LETM1,LIMD1,LOXL3,LPCAT3,LPP,LRRC40,LRRC59,LTA4H,MARK2,MEGF10,MKLN1,MMUT,MON2,MPI,MPST,MTDH,MTSS1,NAA25,NAB2,NAPA,NCLN,NDUFAF7,NEK9,NFKB2,NLE1,NLN,NPTXR,NQO1,NRBP1,NRDC,NT5C3B,NUP54,OAT,OGT,OSBPL8,OXCT1,PAFAH1B1,PAK1,PAPSS1,PARG,PCBP2,PCK2,PDE4DIP,PDZRN3,PEPD,PFKM,PHLDA1,PHYH,PIGU,PIP4P2,PITRM1,PKN2,PLCB1,PMPCB,POLA1,POLD1,POLD3,POLDIP3,PPA2,PPAT,PPOX,PPP2R5D,PPP4R3A,PRDX2,PRKACB,PRKCA,PRPF19,PRRC2A,PRRX1,PTPN1,QSOX2,RAB32,RABGGTA,RAD51,RAF1,RAI14,RANGAP1,RAP2C,RASSF4,RBPJ,RER1,RETSAT,RIOK3,RNASEH2B,RPA1,RPA2,RPL27,RPL3,RPL5,RPN1,RRM1,RRM2,SAMD4B,SDF4,SEC14L1,SEC24A,SEC24D,SEC61A1,SERPINE2,SGTA,SH3GLB1,SLC1A4,SLC9A3R1,SLC9A3R2,SNF8,SNX1,SNX17,SOAT1,SORBS3,SPP1,SQLE,SQSTM1,SRGAP3,STAT1,STAT3,STAT5B,STRN4,STT3A,SUCLG2,TAX1BP1,TCF12,TDRKH,TKFC,TLE3,TLN1,TM9SF3,TM9SF4,TMEM115,TMEM43,TNS3,TOM1L2,TRAPPC4,TRIM2,TRIM28,TRIM3,TRIM47,TRIOBP,TRIP10,TRIP12,TRIP13,TRMT2A,TST,TTC21B,UBAP2L,UBE2C,UBE2O,UCKL1,USP24,USP47,USP8,USP9X,VCP,VCPIP1,VPS11,VPS13A,VPS36,VPS4B,VWA5A,WDR12,WDR45B,WDR62,WFS1,WIPF1,XRN1,ZMPSTE24	319
Cancer,Organismal Injury and Abnormalities	Anogenital cancer	1,63E-10			AARSD1,ABCD4,ACACA,ACADS,ACO1,ACP2,ACSF2,ACSL3,ACSL4,ADD1,ADI1,AGL,AK3,AKR1B1,AKR7A2,ALDH2,ALDH4A1,ALDH9A1,ANKRD13A,AP3S2,APPL2,ARFGAP2,ARFGAP3,ARFGEF2,ARHGEF1,ARHGEF12,ARHGEF18,ASRGL1,ATIC,ATP2B4,ATP6AP1,ATP6V1A,BCAS1,BIRC6,C17orf49,C1QBP,CAMSAP2,CAND1,CANX,CARMIL1,CAVIN1,CBX3,CC2D1B,CCDC47,CCZ1/CCZ1B,CDC42EP4,CDIPT,CDK1,CDK4,CDK6,CEP55,CERT1,CNN2,CNPY2,CRAT,CSNK1A1,CTBP2,CTTNBP2,CUL3,Cyb5r3,DDB1,DDRGK1,DDX5,DGKZ,DGLUCY,DNMT1,DOCK1,DOCK5,DPYSL2,DTYMK,DYNC2H1,EEF1B2,EEF2,EEF2K,ELOA,ELP1,ELP2,ELP3,EMC7,EMD,EML1,ERLIN2,EXOC2,EXOC4,FADD,FAF2,FASN,FBXO7,FDFT1,FLII,FLNA,FLNB,FRMD4A,FUT8,GABPA,GATM,GCDH,GDA,GFPT1,GHDC,GLRX3,GMDS,GMPPB,GOT2,GPT2,GSDMD,GTSE1,HAUS7,HDGFL3,HEBP2,HMGCS1,HMMR,HMOX1,HNRNPUL1,HSPA4L,IDH2,IFT74,IKBIP,INPPL1,IRGQ,ITPR1,ITPR3,KANK1,KPNA2,LARP7,LAYN,LETM1,LIMD1,LOXL3,LPP,LRRC40,MARK2,MEGF10,MGST2,MKLN1,MMUT,MON2,MPST,MTDH,MTSS1,NAA25,NAB2,NAPA,NCLN,NDC80,NEK9,NFKB2,NLE1,NMD3,NPTXR,NQO1,NRDC,NUP54,OAT,OGT,OSBPL8,OXCT1,PAFAH1B1,PAK1,PAPSS1,PARG,PCBP2,PCK2,PDE4DIP,PDZRN3,PEPD,PFKM,PHLDA1,PHYH,PIGU,PIP4P2,PITRM1,PKN2,PLCB1,POLA1,POLD1,POLD3,POLDIP3,PPAT,PPOX,PRKACB,PRKCA,PRPF19,PRRC2A,PRRX1,PTPN1,RAD51,RAF1,RANGAP1,RAP2C,RER1,RETSAT,RIOK3,RNASEH2B,RPA1,RPA2,RPL5,RPL6,RPN1,RRM1,RRM2,SAMD4B,SDF4,SEC14L1,SEC24D,SEC61A1,SERPINE2,SGTA,SH3GLB1,SLC1A4,SLC9A3R1,SNF8,SNX1,SNX17,SOAT1,SORBS3,SPP1,SQLE,SQSTM1,STAT1,STAT3,STAT5B,STT3A,SUCLG2,TAGLN2,TAX1BP1,TCF12,TKFC,TLE3,TLN1,TMEM43,TNS3,TOM1L2,TPD52,TRAPPC4,TRIM28,TRIM3,TRIOBP,TRIP10,TRIP12,TRIP13,TRMT2A,TST,TTC21B,UBAP2L,UBE2C,UBE2O,USP24,USP47,USP8,USP9X,VCPIP1,VPS11,VPS13A,VPS36,VWA5A,WDR12,WFS1,WIPF1,XRN1,ZMPSTE24	261
Amino Acid Metabolism,Small Molecule Biochemistry	Metabolism of amino acids	3,66E-09			ALDH4A1,ALDH6A1,ASRGL1,ATP2B4,BCAT2,DGLUCY,GATM,GCDH,GCLC,GCLM,GOT2,GPT2,GSTZ1,HMGCL,MMUT,MPST,NIT2,OAT,PEPD,PPAT,TST	21
Connective Tissue Disorders,Hereditary Disorder,Organismal Injury and Abnormalities	Hereditary connective tissue disorder	6,26E-09			AKR1B10,ARFGEF2,ATP6V1A,CAVIN1,CDK6,DDRGK1,DYNC2H1,EXTL3,FDFT1,FLNA,FLNB,FRMD4A,IFT74,INPPL1,KATNB1,KIFBP,LARP7,LOXL3,MEGF10,NARS1,NEK9,NFKB2,PAK1,PHYH,PIGU,POLA1,POLD1,PRKACB,RAF1,RPL13,RPL6,RRM1,RRM2,SEC24D,SEC61A1,SLC1A4,SLC9A3R1,SQSTM1,TCF12,TLE3,TRAPPC4,TRIP12,TTC21B,VARS1,VCP,WDR62,ZMPSTE24	47
Cancer,Organismal Injury and Abnormalities	Genitourinary adenocarcinoma	1,19E-08			AARSD1,ABCD4,ACADS,ACSF2,ACSL3,ACSL4,ADD1,AGL,AK3,AKR1B1,ALDH2,ALDH4A1,ANKRD13A,AP3S2,APPL2,ARFGAP2,ARFGAP3,ARFGEF2,ARHGEF1,ARHGEF12,ARHGEF18,ASRGL1,ATIC,ATP2B4,ATP6AP1,BIRC6,CAMSAP2,CAND1,CARMIL1,CAVIN1,CBX3,CC2D1B,CCDC47,CDC42EP4,CDIPT,CDK1,CDK4,CDK6,CEP55,CRAT,CSNK1A1,CTBP2,CTTNBP2,CUL3,DDB1,DDRGK1,DGKZ,DGLUCY,DNMT1,DOCK1,DOCK5,DPY19L1,DPYSL2,DTYMK,DYNC2H1,EEF1B2,EEF2,EEF2K,ELOA,ELP1,ELP2,ELP3,EMC7,EMD,EML1,ERLIN2,EXOC2,EXOC4,FADD,FAF2,FASN,FBXO7,FDFT1,FLII,FLNA,FLNB,FRMD4A,FUT8,GATM,GCDH,GDA,GFPT1,GHDC,GLRX3,GLRX5,GMDS,GMPPB,GOT2,GPT2,GSDMD,GTSE1,HAUS7,HDGFL3,HMMR,HNRNPUL1,HSPA4L,IDH2,IFT74,IKBIP,INPPL1,ITPR1,ITPR3,KANK1,KPNA2,LARP7,LAYN,LETM1,LPP,LRRC40,LTA4H,MEGF10,MKLN1,MMUT,MON2,MPST,MTDH,MTSS1,NAA25,NAB2,NAPA,NCLN,NDC80,NEK9,NFKB2,NLE1,NPTXR,NQO1,NRDC,NUP54,OAT,OGT,OSBPL8,OXCT1,PAFAH1B1,PAK1,PAPSS1,PARG,PCBP2,PCK2,PDE4DIP,PDZRN3,PEPD,PFKM,PHLDA1,PHYH,PIGU,PIP4P2,PITRM1,PKN2,PLCB1,POLA1,POLD1,POLD3,POLDIP3,PPAT,PPOX,PRDX2,PRKACB,PRKCA,PRPF19,PRRC2A,PTPN1,RAB32,RAD51,RAF1,RAI14,RANGAP1,RER1,RETSAT,RIOK3,RNASEH2B,RPA1,RPA2,RPL5,RRM1,RRM2,SAMD4B,SEC14L1,SEC24A,SEC24D,SEC61A1,SERPINE2,SGTA,SH3GLB1,SLC1A4,SLC9A3R1,SNF8,SNX17,SOAT1,SORBS3,SPP1,SQSTM1,STAT1,STAT3,STAT5B,STT3A,SUCLG2,TAX1BP1,TCF12,TKFC,TLE3,TLN1,TMEM43,TNS3,TOM1L2,TPD52,TRAPPC4,TRIM28,TRIM3,TRIOBP,TRIP12,TRIP13,TRMT2A,UBAP2L,UBE2C,UBE2O,USP24,USP47,USP8,USP9X,VPS11,VPS13A,VPS36,VWA5A,WDR12,WDR62,WFS1,WIPF1,XRN1,ZMPSTE24	230
Cancer,Organismal Injury and Abnormalities,Reproductive System Disease	Genital tumor	2,25E-08			AARSD1,ABCD4,ACADS,ACO1,ACSF2,ACSL3,ACSL4,ADD1,ADI1,AGL,AK3,AKR1B1,AKR7A2,ALDH2,ALDH4A1,ALDH9A1,ANKRD13A,AP3S2,APPL2,ARFGAP2,ARFGAP3,ARFGEF2,ARHGEF1,ARHGEF12,ARHGEF18,ASRGL1,ATIC,ATP2B4,ATP6AP1,ATP6V1A,BCAS1,BIRC6,CAMSAP2,CAND1,CARMIL1,CAVIN1,CBX3,CC2D1B,CCDC47,CCZ1/CCZ1B,CDC42EP4,CDIPT,CDK1,CDK4,CDK6,CEP55,CRAT,CTTNBP2,CUL3,DDB1,DDRGK1,DGKZ,DGLUCY,DNMT1,DOCK1,DOCK5,DPYSL2,DPYSL3,DTYMK,DYNC2H1,EEF1B2,EEF2,EEF2K,ELOA,ELP1,ELP2,ELP3,EMC7,EMD,EML1,ERLIN2,EXOC2,EXOC4,FAF2,FASN,FBXO7,FDFT1,FLII,FLNA,FLNB,FRMD4A,FUT8,GATM,GCDH,GDA,GFPT1,GHDC,GLRX3,GMDS,GMPPB,GOT2,GPT2,GSDMD,HAUS7,HDGFL3,HEBP2,HMMR,HMOX1,HNRNPUL1,HSPA4L,IDH2,IFT74,IKBIP,INPPL1,IRGQ,ITPR1,ITPR3,KANK1,KPNA2,LARP7,LAYN,LETM1,LIMD1,LPP,LRRC40,MEGF10,MKLN1,MMUT,MON2,MPST,MTDH,MTSS1,NAA25,NAB2,NAPA,NCLN,NEK9,NFKB2,NLE1,NMD3,NPTXR,NQO1,NRDC,NUP54,OAT,OGT,OSBPL8,OXCT1,PAFAH1B1,PAK1,PAPSS1,PARG,PCBP2,PCK2,PDE4DIP,PDZRN3,PEPD,PFKM,PHLDA1,PHYH,PIGU,PIP4P2,PITRM1,PKN2,PLCB1,POLA1,POLD1,POLD3,POLDIP3,PPAT,PPOX,PRKACB,PRKCA,PRPF19,PRRC2A,PTPN1,RAD51,RAF1,RANGAP1,RAP2C,RER1,RETSAT,RIOK3,RNASEH2B,RPA1,RPA2,RPL27,RPL5,RRM1,RRM2,SAMD4B,SDF4,SEC14L1,SEC24D,SEC61A1,SERPINE2,SGTA,SH3GLB1,SLC1A4,SNF8,SNX1,SNX17,SOAT1,SORBS3,SPP1,SQSTM1,STAT1,STAT3,STAT5B,STT3A,SUCLG2,TAX1BP1,TCF12,TKFC,TLE3,TLN1,TMEM43,TNS3,TOM1L2,TPD52,TRAPPC4,TRIM28,TRIM3,TRIOBP,TRIP12,TRIP13,TRMT2A,TST,UBAP2L,UBE2C,UBE2O,USP24,USP47,USP8,USP9X,VCP,VCPIP1,VPS11,VPS13A,VPS36,VWA5A,WDR12,WFS1,WIPF1,XRN1,ZMPSTE24	236
Cancer,Organismal Injury and Abnormalities	Genitourinary carcinoma	2,34E-08			AARSD1,ABCD4,ACADS,ACP2,ACSF2,ACSL3,ACSL4,ADD1,AGL,AIP,AK3,AKR1B1,ALDH2,ALDH4A1,ANKRD13A,AP3S2,APPL2,ARFGAP2,ARFGAP3,ARFGEF2,ARHGEF1,ARHGEF12,ARHGEF18,ASRGL1,ATIC,ATP2B4,ATP6AP1,ATP6V1A,ATPAF2,BCAS1,BIRC6,C17orf49,C1QBP,CAMSAP2,CAND1,CANX,CARMIL1,CAVIN1,CBX3,CC2D1B,CCDC47,CDC42EP4,CDIPT,CDK1,CDK4,CDK6,CEP55,CNN2,CNPY2,CRAT,CSNK1A1,CTBP2,CTTNBP2,CUL3,DDB1,DDRGK1,DGKZ,DGLUCY,DNMT1,DOCK1,DOCK5,DPY19L1,DPYSL2,DTYMK,DYNC2H1,EEF1B2,EEF2,EEF2K,ELOA,ELP1,ELP2,ELP3,EMC7,EMD,EML1,ERLIN2,EXOC2,EXOC4,FADD,FAF2,FASN,FBXO7,FDFT1,FLII,FLNA,FLNB,FRMD4A,FUT8,GABPA,GATM,GCDH,GDA,GFPT1,GHDC,GLRX3,GLRX5,GMDS,GMPPB,GOT2,GPT2,GRB14,GSDMD,GTSE1,HAUS7,HDGFL3,HIKESHI,HMGCS1,HMMR,HNRNPUL1,HSPA4L,IDH2,IFT74,IKBIP,INPPL1,ITPR1,ITPR3,KANK1,KATNB1,KPNA2,LARP7,LAYN,LETM1,LOXL3,LPP,LRRC40,LTA4H,MARK2,MAT2A,MEGF10,MKLN1,MMUT,MON2,MPST,MTDH,MTSS1,NAA25,NAB2,NAPA,NCLN,NDC80,NEK9,NFKB2,NLE1,NPTXR,NQO1,NRDC,NUP54,OAT,OGT,OSBPL8,OXCT1,PAFAH1B1,PAK1,PAPSS1,PARG,PCBP2,PCK2,PDE4DIP,PDZRN3,PEPD,PFKM,PHLDA1,PHYH,PIGU,PIP4P2,PITRM1,PKN2,PLCB1,POLA1,POLD1,POLD3,POLDIP3,PPAT,PPOX,PRDX2,PRKACB,PRKCA,PRPF19,PRRC2A,PRRX1,PTPN1,RAB32,RAD51,RAF1,RAI14,RANGAP1,RAP2C,RCN2,RER1,RETSAT,RIOK3,RNASEH2B,RPA1,RPA2,RPL5,RRM1,RRM2,SAMD4B,SDF4,SEC14L1,SEC24A,SEC24D,SEC61A1,SERPINE2,SGTA,SH3GLB1,SLC1A4,SLC9A3R1,SNF8,SNX17,SOAT1,SORBS3,SPP1,SQSTM1,STAT1,STAT3,STAT5B,STT3A,SUCLG2,TAX1BP1,TCF12,TKFC,TLE3,TLN1,TMEM43,TNS3,TOM1L2,TPD52,TRAPPC4,TRIM28,TRIM3,TRIOBP,TRIP10,TRIP12,TRIP13,TRMT2A,UBAP2L,UBE2C,UBE2O,UCKL1,USP24,USP47,USP8,USP9X,VCPIP1,VPS11,VPS13A,VPS36,VWA5A,WDR12,WDR62,WFS1,WIPF1,XRN1,ZMPSTE24	255

Categories	Diseases or Functions Annotation	p-value	Predicted Activation State	Activation z-score	Molecules	# Molecules
Cancer,Organismal Injury and Abnormalities,Reproductive System Disease	Genital tract cancer	2,54E-08			AARSD1,ABCD4,ACADS,ACO1,ACSF2,ACSL3,ACSL4,ADD1,ADI1,AGL,AK3,AKR1B1,AKR7A2,ALDH2,ALDH4A1,ALDH9A1,ANKRD13A,AP3S2,APPL2,ARFGAP2,ARFGAP3,ARFGEF2,ARHGEF1,ARHGEF12,ARHGEF18,ASRGL1,ATIC,ATP2B4,ATP6AP1,ATP6V1A,BCAS1,BIRC6,CAMSAP2,CAND1,CARMIL1,CAVIN1,CBX3,CC2D1B,CCDC47,CCZ1/CCZ1B,CDC42EP4,CDIPT,CDK1,CDK4,CDK6,CEP55,CRAT,CTTNBP2,CUL3,DDB1,DDRGK1,DGKZ,DGLUCY,DNMT1,DOCK1,DOCK5,DPYSL2,DTYMK,DYNC2H1,EEF1B2,EEF2,EEF2K,ELOA,ELP1,ELP2,ELP3,EMC7,EMD,EML1,ERLIN2,EXOC2,EXOC4,FAF2,FASN,FBXO7,FDFT1,FLII,FLNA,FLNB,FRMD4A,FUT8,GATM,GCDH,GDA,GFPT1,GHDC,GLRX3,GMDS,GMPPB,GOT2,GPT2,GSDMD,HAUS7,HDGFL3,HEBP2,HMMR,HMOX1,HNRNPUL1,HSPA4L,IDH2,IFT74,IKBIP,INPPL1,IRGQ,ITPR1,ITPR3,KANK1,KPNA2,LARP7,LAYN,LETM1,LIMD1,LPP,LRRC40,MEGF10,MKLN1,MMUT,MON2,MPST,MTDH,MTSS1,NAA25,NAB2,NAPA,NCLN,NEK9,NFKB2,NLE1,NMD3,NPTXR,NQO1,NRDC,NUP54,OAT,OGT,OSBPL8,OXCT1,PAFAH1B1,PAK1,PAPSS1,PARG,PCBP2,PCK2,PDE4DIP,PDZRN3,PEPD,PFKM,PHLDA1,PHYH,PIGU,PIP4P2,PITRM1,PKN2,PLCB1,POLA1,POLD1,POLD3,POLDIP3,PPAT,PPOX,PRKACB,PRKCA,PRPF19,PRRC2A,PTPN1,RAD51,RAF1,RANGAP1,RAP2C,RER1,RETSAT,RIOK3,RNASEH2B,RPA1,RPA2,RPL5,RRM1,RRM2,SAMD4B,SDF4,SEC14L1,SEC24D,SEC61A1,SERPINE2,SGTA,SH3GLB1,SLC1A4,SNF8,SNX1,SNX17,SOAT1,SORBS3,SPP1,SQSTM1,STAT1,STAT3,STAT5B,STT3A,SUCLG2,TAX1BP1,TCF12,TKFC,TLE3,TLN1,TMEM43,TNS3,TOM1L2,TPD52,TRAPPC4,TRIM28,TRIM3,TRIOBP,TRIP12,TRIP13,TRMT2A,TST,UBAP2L,UBE2C,UBE2O,USP24,USP47,USP8,USP9X,VCPIP1,VPS11,VPS13A,VPS36,VWA5A,WDR12,WFS1,WIPF1,XRN1,ZMPSTE24	233
Cancer,Organismal Injury and Abnormalities	Pelvic tumor	3,2E-08			AARSD1,ABCD4,ACADS,ACO1,ACSF2,ACSL3,ACSL4,ADD1,ADI1,AGL,AIP,AK3,AKR1B1,AKR7A2,ALDH2,ALDH4A1,ALDH9A1,ANKRD13A,AP3S2,APPL2,ARFGAP2,ARFGAP3,ARFGEF2,ARHGEF1,ARHGEF12,ARHGEF18,ARMC8,ASRGL1,ATIC,ATP2B4,ATP6AP1,ATP6V1A,ATPAF2,BCAS1,BIRC6,CAMSAP2,CAND1,CARMIL1,CAVIN1,CBX3,CC2D1B,CCDC47,CCZ1/CCZ1B,CDC42EP4,CDIPT,CDK1,CDK4,CDK6,CEP55,CRAT,CTBP2,CTTNBP2,CUL3,DDB1,DDRGK1,DGKZ,DGLUCY,DNMT1,DOCK1,DOCK5,DPYSL2,DPYSL3,DTYMK,DYNC2H1,EEF1B2,EEF2,EEF2K,ELOA,ELP1,ELP2,ELP3,EMC7,EMD,EML1,ERLIN2,EXOC2,EXOC4,FADD,FAF2,FASN,FBXO7,FDFT1,FLII,FLNA,FLNB,FRMD4A,FUT8,GATM,GCDH,GDA,GFPT1,GHDC,GLRX3,GMDS,GMPPB,GOT2,GPT2,GRB14,GSDMD,HAUS7,HDGFL3,HEBP2,HIKESHI,HMMR,HMOX1,HNRNPUL1,HSPA4L,IDH2,IFT74,IKBIP,INPPL1,IRGQ,ITPR1,ITPR3,KANK1,KATNB1,KPNA2,LARP7,LAYN,LETM1,LIMD1,LPP,LRRC40,MARK2,MAT2A,MEGF10,MKLN1,MMUT,MON2,MPST,MTDH,MTSS1,NAA25,NAB2,NAPA,NCLN,NEK9,NFKB2,NLE1,NMD3,NPTXR,NQO1,NRDC,NUP54,OAT,OGT,OSBPL8,OXCT1,PAFAH1B1,PAK1,PAPSS1,PARG,PCBP2,PCK2,PDE4DIP,PDZRN3,PEPD,PFKM,PHLDA1,PHYH,PIGU,PIP4P2,PITRM1,PKN2,PLCB1,POLA1,POLD1,POLD3,POLDIP3,PPAT,PPOX,PRKACB,PRKCA,PRPF19,PRRC2A,PTPN1,RAD51,RAF1,RANGAP1,RCN2,RER1,RETSAT,RIOK3,RNASEH2B,RPA1,RPA2,RPL27,RPL5,RRM1,RRM2,SAMD4B,SDF4,SEC14L1,SEC24D,SEC61A1,SERPINE2,SGTA,SH3GLB1,SLC1A4,SLC9A3R1,SNF8,SNX1,SNX17,SOAT1,SORBS3,SPP1,SQSTM1,STAT1,STAT3,STAT5B,STT3A,SUCLG2,TAX1BP1,TCF12,TKFC,TLN1,TMEM43,TNS3,TOM1L2,TPD52,TRAPPC4,TRIM28,TRIM3,TRIOBP,TRIP12,TRIP13,TRMT2A,TST,TTC21B,UBAP2L,UBE2C,UBE2O,UCKL1,USP24,USP47,USP8,USP9X,VCP,VCPIP1,VPS11,VPS13A,VPS36,VWA5A,WDR12,WFS1,WIPF1,XRN1,ZMPSTE24	248
Cancer,Organismal Injury and Abnormalities	Pelvic cancer	4,12E-08			AARSD1,ABCD4,ACADS,ACO1,ACSF2,ACSL3,ACSL4,ADD1,ADI1,AGL,AIP,AK3,AKR1B1,AKR7A2,ALDH2,ALDH4A1,ALDH9A1,ANKRD13A,AP3S2,APPL2,ARFGAP2,ARFGAP3,ARFGEF2,ARHGEF1,ARHGEF12,ARHGEF18,ARMC8,ASRGL1,ATIC,ATP2B4,ATP6AP1,ATP6V1A,ATPAF2,BCAS1,BIRC6,CAMSAP2,CAND1,CARMIL1,CAVIN1,CBX3,CC2D1B,CCDC47,CCZ1/CCZ1B,CDC42EP4,CDIPT,CDK1,CDK4,CDK6,CEP55,CRAT,CTBP2,CTTNBP2,CUL3,DDB1,DDRGK1,DGKZ,DGLUCY,DNMT1,DOCK1,DOCK5,DPYSL2,DTYMK,DYNC2H1,EEF1B2,EEF2,EEF2K,ELOA,ELP1,ELP2,ELP3,EMC7,EMD,EML1,ERLIN2,EXOC2,EXOC4,FADD,FAF2,FASN,FBXO7,FDFT1,FLII,FLNA,FLNB,FRMD4A,FUT8,GATM,GCDH,GDA,GFPT1,GHDC,GLRX3,GMDS,GMPPB,GOT2,GPT2,GRB14,GSDMD,HAUS7,HDGFL3,HEBP2,HIKESHI,HMMR,HMOX1,HNRNPUL1,HSPA4L,IDH2,IFT74,IKBIP,INPPL1,IRGQ,ITPR1,ITPR3,KANK1,KATNB1,KPNA2,LARP7,LAYN,LETM1,LIMD1,LPP,LRRC40,MARK2,MAT2A,MEGF10,MKLN1,MMUT,MON2,MPST,MTDH,MTSS1,NAA25,NAB2,NAPA,NCLN,NEK9,NFKB2,NLE1,NMD3,NPTXR,NQO1,NRDC,NUP54,OAT,OGT,OSBPL8,OXCT1,PAFAH1B1,PAK1,PAPSS1,PARG,PCBP2,PCK2,PDE4DIP,PDZRN3,PEPD,PFKM,PHLDA1,PHYH,PIGU,PIP4P2,PITRM1,PKN2,PLCB1,POLA1,POLD1,POLD3,POLDIP3,PPAT,PPOX,PRKACB,PRKCA,PRPF19,PRRC2A,PTPN1,RAD51,RAF1,RANGAP1,RCN2,RER1,RETSAT,RIOK3,RNASEH2B,RPA1,RPA2,RPL5,RRM1,RRM2,SAMD4B,SDF4,SEC14L1,SEC24D,SEC61A1,SERPINE2,SGTA,SH3GLB1,SLC1A4,SLC9A3R1,SNF8,SNX1,SNX17,SOAT1,SORBS3,SPP1,SQSTM1,STAT1,STAT3,STAT5B,STT3A,SUCLG2,TAX1BP1,TCF12,TKFC,TLN1,TMEM43,TNS3,TOM1L2,TPD52,TRAPPC4,TRIM28,TRIM3,TRIOBP,TRIP12,TRIP13,TRMT2A,TST,TTC21B,UBAP2L,UBE2C,UBE2O,UCKL1,USP24,USP47,USP8,USP9X,VCPIP1,VPS11,VPS13A,VPS36,VWA5A,WDR12,WFS1,WIPF1,XRN1,ZMPSTE24	245
Cancer,Organismal Injury and Abnormalities,Reproductive System Disease	Breast or gynecological cancer	1,02E-07			ABCD4,ACACA,ACADS,ACP2,ACSL3,ACSL4,ADD1,ADI1,AGL,AK3,AKR1B1,AKR7A2,ALDH2,ALDH4A1,AP3S2,APPL2,ARFGAP3,ARFGEF2,ARHGEF1,ARHGEF12,ARHGEF18,ASRGL1,ATIC,ATP6AP1,ATP6V1A,BCAS1,BIRC6,C17orf49,C1QBP,CAMSAP2,CAND1,CANX,CAVIN1,CBX3,CCDC47,CCZ1/CCZ1B,CDIPT,CDK1,CDK4,CDK6,CEP55,CERT1,CNN2,CNPY2,CRAT,CSNK1A1,CTBP2,CTTNBP2,Cyb5r3,DDB1,DDRGK1,DDX5,DGKZ,DGLUCY,DNMT1,DOCK1,DOCK5,DPYSL2,DTYMK,DYNC2H1,EEF1B2,EEF2,ELOA,ELP1,ELP2,ELP3,EMC7,EMD,ERLIN2,EXOC2,EXOC4,FADD,FAF2,FASN,FBXO7,FDFT1,FLII,FLNA,FLNB,FRMD4A,FUT8,GABPA,GATM,GCDH,GFPT1,GHDC,GLRX3,GMDS,GMPPB,GOT2,GPT2,GTSE1,HAUS7,HDGFL3,HMGCS1,HMMR,HMOX1,HNRNPUL1,HSPA4L,IDH2,IFT74,IKBIP,INPPL1,ITPR1,ITPR3,KPNA2,LARP7,LAYN,LETM1,LIMD1,LOXL3,LPP,LRRC40,MARK2,MEGF10,MGST2,MKLN1,MMUT,MON2,MTDH,MTSS1,NAA25,NAB2,NAPA,NCLN,NDC80,NEK9,NFKB2,NPTXR,NQO1,NRDC,NUP54,OAT,OGT,OSBPL8,OXCT1,PAK1,PARG,PCBP2,PCK2,PDE4DIP,PDZRN3,PEPD,PFKM,PHLDA1,PHYH,PIGU,PITRM1,PKN2,PLCB1,POLA1,POLD1,POLD3,POLDIP3,PPAT,PPOX,PRKCA,PRPF19,PRRC2A,PRRX1,PTPN1,RAD51,RAF1,RANGAP1,RER1,RETSAT,RIOK3,RNASEH2B,RPL5,RPL6,RPN1,RRM1,RRM2,SAMD4B,SDF4,SEC14L1,SEC61A1,SLC9A3R1,SNF8,SNX1,SNX17,SOAT1,SORBS3,SPP1,SQLE,STAT1,STAT3,STAT5B,STT3A,SUCLG2,TAGLN2,TAX1BP1,TCF12,TKFC,TLE3,TLN1,TMEM43,TNS3,TOM1L2,TPD52,TRIM28,TRIM3,TRIOBP,TRIP10,TRIP12,TRIP13,TRMT2A,TST,UBAP2L,UBE2C,USP24,USP47,USP8,USP9X,VCPIP1,VPS11,VPS13A,VWA5A,WFS1,WIPF1,XRN1,ZMPSTE24	222
Cancer,Cardiovascular Disease,Hematological Disease,Organismal Injury and Abnormalities	Refractory anemia with excess of blasts type 2	1,46E-07			DNMT1,IDH2,POLA1,POLD1,PRKCA,RRM1,RRM2	7
Cancer,Organismal Injury and Abnormalities	Pelvic adenocarcinoma	1,53E-07			AARSD1,ABCD4,ACADS,ACSF2,ACSL3,ACSL4,ADD1,AGL,AK3,AKR1B1,ALDH2,ALDH4A1,ANKRD13A,AP3S2,APPL2,ARFGAP2,ARFGAP3,ARFGEF2,ARHGEF1,ARHGEF12,ARHGEF18,ARMC8,ASRGL1,ATIC,ATP2B4,ATP6AP1,BIRC6,CAMSAP2,CAND1,CARMIL1,CAVIN1,CBX3,CC2D1B,CCDC47,CDC42EP4,CDIPT,CDK4,CDK6,CEP55,CRAT,CTBP2,CTTNBP2,CUL3,DDB1,DGKZ,DGLUCY,DNMT1,DOCK1,DOCK5,DPYSL2,DTYMK,DYNC2H1,EEF1B2,EEF2,EEF2K,ELOA,ELP1,ELP2,ELP3,EMC7,EMD,EML1,ERLIN2,EXOC2,EXOC4,FAF2,FASN,FBXO7,FDFT1,FLII,FLNA,FLNB,FRMD4A,FUT8,GATM,GCDH,GDA,GFPT1,GHDC,GLRX3,GMDS,GMPPB,GOT2,GPT2,GSDMD,HAUS7,HDGFL3,HMMR,HNRNPUL1,HSPA4L,IDH2,IFT74,IKBIP,INPPL1,ITPR1,ITPR3,KANK1,KPNA2,LARP7,LAYN,LETM1,LPP,LRRC40,MEGF10,MKLN1,MMUT,MON2,MPST,MTDH,MTSS1,NAA25,NAB2,NAPA,NCLN,NEK9,NFKB2,NLE1,NPTXR,NQO1,NRDC,NUP54,OAT,OGT,OSBPL8,OXCT1,PAK1,PAPSS1,PARG,PCBP2,PCK2,PDE4DIP,PDZRN3,PEPD,PFKM,PHLDA1,PHYH,PIGU,PIP4P2,PITRM1,PKN2,PLCB1,POLA1,POLD1,POLD3,POLDIP3,PPAT,PPOX,PRKACB,PRKCA,PRPF19,PRRC2A,PTPN1,RAD51,RAF1,RANGAP1,RER1,RETSAT,RIOK3,RNASEH2B,RPA1,RPA2,RPL5,RRM1,RRM2,SAMD4B,SEC14L1,SEC24D,SEC61A1,SERPINE2,SGTA,SH3GLB1,SLC1A4,SNF8,SNX17,SOAT1,SORBS3,SPP1,SQSTM1,STAT1,STAT3,STAT5B,STT3A,SUCLG2,TAX1BP1,TCF12,TKFC,TLN1,TMEM43,TNS3,TOM1L2,TRAPPC4,TRIM28,TRIM3,TRIOBP,TRIP12,TRIP13,TRMT2A,UBAP2L,UBE2C,UBE2O,USP24,USP47,USP8,USP9X,VPS11,VPS13A,VPS36,VWA5A,WDR12,WFS1,WIPF1,XRN1,ZMPSTE24	213
Developmental Disorder,Hereditary Disorder,Neurological Disease,Organismal Injury and Abnormalities	Autosomal recessive neurodevelopmental syndrome	1,66E-07			ELP1,EXTL3,GOT2,NARS1,PIGU,PLCB1,SLC1A4,TRAPPC4,UBR7,VARS1,WDR45B,WDR62	12
Hereditary Disorder,Organismal Injury and Abnormalities,Psychological Disorders	Familial psychiatric disease	2,43E-07			ACO2,ALDH2,ALDH6A1,ARHGEF1,ATP2A2,ATP6V1A,CRAT,CUL3,DNMT1,DPYSL3,EEF2,EIF2B4,EIF2B5,EML1,FBXO7,FDFT1,FRMD4A,GATM,HIKESHI,HMGCS1,HMOX1,IMPA1,INPP1,ITPR1,KANK1,LOXL3,LPP,MTDH,NAPA,NARS1,NDUFAF7,NFKB2,OSBPL8,PAK1,PDE4DIP,PFKM,PHYH,PLCB1,PRDX2,RAB6A,RNASEH2B,SEC24A,SGTA,SLC1A4,SOAT1,SQSTM1,STAT1,STRN4,TPD52,TRAPPC4,VARS1,VCP,VPS11,VPS13A	54
Cancer,Hematological Disease,Immunological Disease,Organismal Injury and Abnormalities	Therapy-related leukemia	3,91E-07			DNMT1,IDH2,NQO1,POLA1,POLD1,RAD51,RPL3,RRM1,RRM2	9
Cancer,Hematological Disease,Immunological Disease,Organismal Injury and Abnormalities	Secondary leukemia	4,91E-07			DNMT1,IDH2,NQO1,POLA1,POLD1,PPAT,PRKCA,RAD51,RPL3,RRM1,RRM2,STAT3	12
Cancer,Gastrointestinal Disease,Hepatic System Disease,Organismal Injury and Abnormalities	Hepatitis B virus-related hepatocellular carcinoma	5,39E-07			ACSL3,ACSL4,ALDH4A1,CDK1,CDK4,EEF2,FASN,POLA1,POLD1,SOAT1,TST,VCP	12
Cancer,Hematological Disease,Immunological Disease,Organismal Injury and Abnormalities	Newly diagnosed acute myeloid leukemia	0,00000061			DNMT1,IDH2,POLA1,POLD1,PPAT,PRKCA,RPL3,RRM1,RRM2	9
Neurological Disease	Progressive neurological disorder	7,37E-07			ALDH2,ATIC,BCAS1,CANX,CDK1,CDK4,DPYSL2,DPYSL3,EEF2,EEF2K,ELP3,FADD,FASN,FBXO7,FDFT1,GATM,GLRX,GSDMD,HMGCL,HMGCS1,HMOX1,HMOX2,LOXL3,MEGF10,MMUT,NAXE,NFKB2,NFS1,NQO1,OGT,PAK1,PIP4P2,PMPCB,POLA1,POLD1,PPAT,PRDX2,PRKACB,PRKCA,PRRC2A,RAB6A,RAF1,RPL13,RPL3,RPL5,RRM1,RRM2,SERPINE2,SLC1A4,SOAT1,SPP1,SQLE,SQSTM1,STAT3,TRAPPC4,USP24,VCP,WFS1	58
Cancer,Organismal Injury and Abnormalities,Reproductive System Disease	Genital carcinoma	9,39E-07			AARSD1,ABCD4,ACADS,ACSF2,ACSL3,ACSL4,ADD1,AGL,AK3,AKR1B1,ALDH2,ALDH4A1,ANKRD13A,AP3S2,APPL2,ARFGAP2,ARFGAP3,ARFGEF2,ARHGEF1,ARHGEF12,ARHGEF18,ASRGL1,ATIC,ATP2B4,ATP6AP1,ATP6V1A,BCAS1,BIRC6,CAMSAP2,CAND1,CARMIL1,CAVIN1,CBX3,CC2D1B,CCDC47,CDC42EP4,CDIPT,CDK4,CDK6,CEP55,CRAT,CTTNBP2,CUL3,DDB1,DGKZ,DGLUCY,DNMT1,DOCK1,DOCK5,DPYSL2,DTYMK,DYNC2H1,EEF1B2,EEF2,EEF2K,ELOA,ELP1,ELP2,ELP3,EMC7,EMD,EML1,ERLIN2,EXOC2,EXOC4,FAF2,FASN,FBXO7,FDFT1,FLII,FLNA,FLNB,FRMD4A,FUT8,GATM,GCDH,GDA,GFPT1,GHDC,GLRX3,GMDS,GMPPB,GOT2,GPT2,GSDMD,HAUS7,HDGFL3,HMMR,HNRNPUL1,HSPA4L,IDH2,IFT74,IKBIP,INPPL1,ITPR1,ITPR3,KANK1,KPNA2,LARP7,LAYN,LETM1,LPP,LRRC40,MEGF10,MKLN1,MMUT,MON2,MPST,MTDH,MTSS1,NAA25,NAB2,NAPA,NCLN,NFKB2,NLE1,NPTXR,NQO1,NRDC,NUP54,OAT,OGT,OSBPL8,OXCT1,PAK1,PAPSS1,PARG,PCBP2,PCK2,PDE4DIP,PDZRN3,PEPD,PFKM,PHLDA1,PHYH,PIGU,PIP4P2,PITRM1,PKN2,PLCB1,POLA1,POLD1,POLD3,POLDIP3,PPAT,PPOX,PRKACB,PRKCA,PRPF19,PRRC2A,PTPN1,RAD51,RAF1,RANGAP1,RAP2C,RER1,RETSAT,RIOK3,RNASEH2B,RPA1,RPA2,RPL5,RRM1,RRM2,SAMD4B,SDF4,SEC14L1,SEC24D,SEC61A1,SERPINE2,SGTA,SH3GLB1,SLC1A4,SNF8,SNX17,SOAT1,SORBS3,SPP1,SQSTM1,STAT1,STAT3,STAT5B,STT3A,SUCLG2,TAX1BP1,TCF12,TKFC,TLN1,TMEM43,TNS3,TOM1L2,TRAPPC4,TRIM28,TRIM3,TRIOBP,TRIP12,TRIP13,TRMT2A,UBAP2L,UBE2C,UBE2O,USP24,USP47,USP8,USP9X,VPS11,VPS13A,VPS36,VWA5A,WDR12,WFS1,WIPF1,XRN1,ZMPSTE24	214
Cancer,Hematological Disease,Immunological Disease,Organismal Injury and Abnormalities	Newly diagnosed secondary acute myeloid leukemia	0,00000102			DNMT1,POLA1,POLD1,RPL3	4
Cancer,Organismal Injury and Abnormalities	Pelvic carcinoma	0,00000118			AARSD1,ABCD4,ACADS,ACSF2,ACSL3,ACSL4,ADD1,AGL,AIP,AK3,AKR1B1,ALDH2,ALDH4A1,ANKRD13A,AP3S2,APPL2,ARFGAP2,ARFGAP3,ARFGEF2,ARHGEF1,ARHGEF12,ARHGEF18,ARMC8,ASRGL1,ATIC,ATP2B4,ATP6AP1,ATP6V1A,ATPAF2,BCAS1,BIRC6,CAMSAP2,CAND1,CARMIL1,CAVIN1,CBX3,CC2D1B,CCDC47,CDC42EP4,CDIPT,CDK4,CDK6,CEP55,CRAT,CTBP2,CTTNBP2,CUL3,DDB1,DGKZ,DGLUCY,DNMT1,DOCK1,DOCK5,DPYSL2,DTYMK,DYNC2H1,EEF1B2,EEF2,EEF2K,ELOA,ELP1,ELP2,ELP3,EMC7,EMD,EML1,ERLIN2,EXOC2,EXOC4,FADD,FAF2,FASN,FBXO7,FDFT1,FLII,FLNA,FLNB,FRMD4A,FUT8,GATM,GCDH,GDA,GFPT1,GHDC,GLRX3,GMDS,GMPPB,GOT2,GPT2,GRB14,GSDMD,HAUS7,HDGFL3,HIKESHI,HMMR,HNRNPUL1,HSPA4L,IDH2,IFT74,IKBIP,INPPL1,ITPR1,ITPR3,KANK1,KATNB1,KPNA2,LARP7,LAYN,LETM1,LPP,LRRC40,MARK2,MAT2A,MEGF10,MKLN1,MMUT,MON2,MPST,MTDH,MTSS1,NAA25,NAB2,NAPA,NCLN,NEK9,NFKB2,NLE1,NPTXR,NQO1,NRDC,NUP54,OAT,OGT,OSBPL8,OXCT1,PAK1,PAPSS1,PARG,PCBP2,PCK2,PDE4DIP,PDZRN3,PEPD,PFKM,PHLDA1,PHYH,PIGU,PIP4P2,PITRM1,PKN2,PLCB1,POLA1,POLD1,POLD3,POLDIP3,PPAT,PPOX,PRKACB,PRKCA,PRPF19,PRRC2A,PTPN1,RAD51,RAF1,RANGAP1,RCN2,RER1,RETSAT,RIOK3,RNASEH2B,RPA1,RPA2,RPL5,RRM1,RRM2,SAMD4B,SDF4,SEC14L1,SEC24D,SEC61A1,SERPINE2,SGTA,SH3GLB1,SLC1A4,SLC9A3R1,SNF8,SNX17,SOAT1,SORBS3,SPP1,SQSTM1,STAT1,STAT3,STAT5B,STT3A,SUCLG2,TAX1BP1,TCF12,TKFC,TLN1,TMEM43,TNS3,TOM1L2,TRAPPC4,TRIM28,TRIM3,TRIOBP,TRIP12,TRIP13,TRMT2A,TTC21B,UBAP2L,UBE2C,UBE2O,UCKL1,USP24,USP47,USP8,USP9X,VPS11,VPS13A,VPS36,VWA5A,WDR12,WFS1,WIPF1,XRN1,ZMPSTE24	228
Neurological Disease,Skeletal and Muscular Disorders	Neuromuscular disease	0,00000152			ALDH2,ALDH6A1,ARHGEF1,ATP2A2,ATP6V1A,BCAS1,CANX,DPYSL3,EMD,EML1,FBXO7,FDFT1,FRMD4A,GFPT1,GSDMD,HMOX1,HMOX2,IMPA1,INPP1,ITPR1,LOXL3,LPP,MMUT,MTDH,NAPA,NAXE,NDUFAF7,OSBPL8,PAK1,PDE4DIP,PFKM,PIP4P2,PLCB1,POLA1,PPAT,PRDX2,PRRC2A,RAB6A,RPL13,RPL3,RPL5,RRM1,RRM2,SEC24A,SGTA,SLC1A4,SPP1,SQLE,SQSTM1,STAT1,STRN4,TPD52,TRAPPC4,USP24,VCP	55
Cancer,Hematological Disease,Organismal Injury and Abnormalities	Myelodysplastic syndrome	0,00000162			CDK1,CRAT,CSNK1A1,DNMT1,DYNC2H1,IDH2,IFT74,PIGU,POLA1,POLD1,PPAT,PRKACB,PRKCA,RAD51,RPL13,RPL3,RPL5,RPL6,RRM1,RRM2,STAT3,STAT5B,TLE3	23
Cancer,Hematological Disease,Immunological Disease,Organismal Injury and Abnormalities	MYC mutation positive B-cell lymphoma	0,00000192			CDK1,HMMR,KPNA2,NDC80,RAD51,RPA1,RRM1,RRM2,TRIP13,UBE2C	10
Cancer,Hematological Disease,Immunological Disease,Organismal Injury and Abnormalities	Leukemic transformation of myelodysplastic syndrome	0,00000237			DNMT1,IDH2,POLA1,POLD1,PPAT,RRM1,RRM2	7
Cancer,Organismal Injury and Abnormalities	BRAF-mutation positive non-melanoma solid tumor	0,00000251			ATIC,CDK4,CDK6,POLA1,POLD1,RAF1,RRM1,RRM2	8
Cancer,Hematological Disease,Immunological Disease,Organismal Injury and Abnormalities	Treatment-related acute myeloid leukemia	0,0000029			DNMT1,IDH2,POLA1,POLD1,RAD51,RPL3,RRM1,RRM2	8
Cancer,Hematological Disease,Organismal Injury and Abnormalities	De novo myelodysplastic syndrome	0,00000316			DNMT1,POLA1,POLD1,PPAT,RRM1,RRM2	6

Categories	Diseases or Functions Annotation	p-value	Predicted Activation State	Activation z-score	Molecules	# Molecules
Cancer,Neurological Disease,Organismal Injury and Abnormalities	Brain tumor	0,00000349			ABCD4,ACADS,ACP2,ACSF2,ADD1,AGA,AGFG1,AIP,AKR1B1,AKR1B10,ALDH2,ALDH9A1,ANAPC7,AP3S2,APPL2,ARFGAP2,ARFGAP3,ARFGEF2,ARHGEF1,ARHGEF18,ARPIN/ARPIN-AP3S2,ASRGL1,ATIC,ATP2A2,ATP2B4,ATP6AP1,ATP6V1A,ATPAF2,ATXN10,BCAS1,BCAT2,BIRC6,CACYBP,CAMSAP2,CAND1,CANX,CARMIL1,CC2D1B,CCDC47,CDC42EP4,CDK1,CDK4,CDK6,CEP55,CNN2,CNN3,CRAT,CTBP2,CTTNBP2,CUL3,CWF19L1,CYFIP1,DDB1,DDRGK1,DDX5,DNMT1,DNPEP,DOCK1,DOCK5,DPY19L1,DYNC2H1,EEF2K,EIF2B4,EIF2B5,ELP1,ELP2,ELP3,ERLIN2,EXOC4,FADD,FASN,FBXL15,FBXO7,FLII,FLNA,FLNB,FUT8,GABPA,GCLC,GCLM,GDA,GFPT1,GMDS,GMPPB,GPT2,GRB14,GSDMD,GTSE1,HDGFL3,HEBP2,HMGCS1,HMMR,HMOX1,IDH2,INPPL1,ITPR1,ITPR3,ITPRID2,KANK1,KANK2,KATNB1,KPNA2,LETM1,LPP,LRRC40,MARK2,MEGF10,MKLN1,MMUT,MON2,MPI,MTDH,MTSS1,NAA25,NCLN,NFKB2,NIT2,NLE1,NLN,NRBP1,NRDC,NT5C3B,OGT,OXCT1,PAFAH1B1,PAK1,PAPSS1,PCBP2,PCK2,PDE4DIP,PDZRN3,PEPD,PFKM,PHLDA1,PHYH,PIP4P2,PITRM1,PKN2,PLCB1,POLA1,POLD1,PPAT,PPP4R3A,PRKCA,PRRC2A,PTPN1,QSOX2,RAB32,RABGGTA,RAF1,RANGAP1,RASSF4,RBPJ,RPA1,RPL5,RPN1,RPN2,RRM1,RRM2,SAMD4B,SDF4,SEC14L1,SEC24A,SEC24D,SH3GLB1,SLC1A4,SNX1,SNX17,SOAT1,SORBS3,SPCS3,SPP1,SQSTM1,SRGAP3,STARD3NL,STAT1,STAT3,STAT5B,STRN4,STT3A,TAX1BP1,TCF12,TDRKH,TLE3,TLN1,TM9SF4,TMEM43,TNS3,TOM1L2,TPD52,TRIM2,TRIM28,TRIOBP,TRIP10,TRIP12,UBE2C,UBE2O,UBR7,USP24,USP47,USP8,USP9X,VCP,VCPIP1,VPS13A,VPS36,VWA5A,WDR62,WFS1,WIPF1,XRN1	211
Cancer,Hematological Disease,Immunological Disease,Organismal Injury and Abnormalities	Secondary acute myeloid leukemia	0,00000384			DNMT1,IDH2,POLA1,POLD1,PPAT,PRKCA,RAD51,RPL3,RRM1,RRM2	10
Cancer,Cardiovascular Disease,Hematological Disease,Immunological Disease,Organismal Injury and Abnormalities	Refractory anemia with excess blasts in transformation	0,00000401			DNMT1,POLA1,POLD1,PPAT,RRM1,RRM2	6
Cancer,Hematological Disease,Immunological Disease,Organismal Injury and Abnormalities	Acute myeloid leukemia with myelodysplasia-related changes	0,00000423			DNMT1,POLA1,POLD1,RRM1,RRM2	5
Amino Acid Metabolism,Small Molecule Biochemistry	Catabolism of amino acids	0,00000484			ALDH4A1,ALDH6A1,ASRGL1,BCAT2,GCDH,GOT2,GSTZ1,HMGCL,MPST,PPAT,TST	11
Cancer,Neurological Disease,Organismal Injury and Abnormalities	Brain glioma	0,00000578			ABCD4,ACADS,ACP2,ACSF2,ADD1,AGA,AGFG1,AIP,AKR1B1,AKR1B10,ALDH2,ALDH9A1,ANAPC7,AP3S2,APPL2,ARFGAP2,ARFGAP3,ARFGEF2,ARHGEF1,ARHGEF18,ARPIN/ARPIN-AP3S2,ASRGL1,ATIC,ATP2A2,ATP2B4,ATP6AP1,ATP6V1A,ATPAF2,ATXN10,BCAS1,BCAT2,BIRC6,CACYBP,CAMSAP2,CAND1,CANX,CARMIL1,CC2D1B,CCDC47,CDC42EP4,CDK1,CDK6,CEP55,CNN2,CNN3,CRAT,CTBP2,CTTNBP2,CUL3,CWF19L1,CYFIP1,DDB1,DDRGK1,DDX5,DNMT1,DNPEP,DOCK1,DOCK5,DPY19L1,DYNC2H1,EEF2K,EIF2B4,EIF2B5,ELP1,ELP2,ELP3,ERLIN2,EXOC4,FADD,FASN,FBXL15,FBXO7,FLII,FLNA,FLNB,FUT8,GABPA,GCLC,GCLM,GDA,GFPT1,GMDS,GMPPB,GPT2,GRB14,GSDMD,GTSE1,HDGFL3,HEBP2,HMGCS1,HMMR,HMOX1,IDH2,INPPL1,ITPR1,ITPR3,ITPRID2,KANK1,KANK2,KATNB1,KPNA2,LETM1,LPP,LRRC40,MARK2,MEGF10,MKLN1,MMUT,MON2,MPI,MTDH,MTSS1,NAA25,NCLN,NFKB2,NIT2,NLE1,NLN,NRBP1,NRDC,NT5C3B,OGT,OXCT1,PAFAH1B1,PAK1,PAPSS1,PCBP2,PCK2,PDE4DIP,PDZRN3,PEPD,PFKM,PHLDA1,PHYH,PIP4P2,PITRM1,PKN2,PLCB1,POLA1,POLD1,PPAT,PPP4R3A,PRRC2A,PTPN1,QSOX2,RAB32,RABGGTA,RAF1,RANGAP1,RASSF4,RBPJ,RPA1,RPL5,RPN1,RPN2,RRM1,RRM2,SAMD4B,SDF4,SEC14L1,SEC24A,SEC24D,SH3GLB1,SLC1A4,SNX1,SNX17,SOAT1,SORBS3,SPCS3,SPP1,SQSTM1,SRGAP3,STARD3NL,STAT1,STAT5B,STRN4,STT3A,TAX1BP1,TCF12,TDRKH,TLE3,TLN1,TM9SF4,TMEM43,TNS3,TOM1L2,TPD52,TRIM2,TRIM28,TRIOBP,TRIP10,TRIP12,UBE2C,UBE2O,UBR7,USP24,USP47,USP8,USP9X,VCP,VCPIP1,VPS13A,VPS36,VWA5A,WDR62,WFS1,WIPF1,XRN1	208
Cancer,Hematological Disease,Immunological Disease,Organismal Injury and Abnormalities	Primary refractory acute myeloid leukemia	0,00000592			IDH2,POLA1,POLD1,RRM1,RRM2	5
Cancer,Hematological Disease,Immunological Disease,Organismal Injury and Abnormalities	Recurrent acute myeloid leukemia	0,00000644			DNMT1,IDH2,POLA1,POLD1,RAF1,RPL3,RRM1,RRM2	8
Cancer,Cardiovascular Disease,Hematological Disease,Organismal Injury and Abnormalities	Refractory anemia with excess blasts	0,00000644			DNMT1,IDH2,POLA1,POLD1,PPAT,PRKCA,RRM1,RRM2	8
Cancer,Hematological Disease,Immunological Disease,Organismal Injury and Abnormalities	Waldenström macroglobulinemia	0,00000656			ANXA4,CDK1,CTBP2,DNMT1,ECI2,EXOC4,FADD,FASN,GTF2B,HMOX1,KPNA2,LPP,POLA1,PRKCA,PTPN1,RRM1,RRM2,SNX1,STAT3	19
Cancer,Dermatological Diseases and Conditions,Organismal Injury and Abnormalities	Skin cancer	0,00000736			AARSD1,ABCD4,ACO1,ACO2,ACP2,ACSF2,ACSL3,ACSL4,ADD1,ADI1,AGA,AGFG1,AGL,AKR1B1,AKR1B10,ALDH2,ALDH4A1,ALDH6A1,ALDH9A1,ANAPC7,ANKRD13A,ANKRD46,ANXA4,APPL2,ARFGAP2,ARFGAP3,ARFGEF2,ARHGEF1,ARHGEF12,ARHGEF18,ARPIN/ARPIN-AP3S2,ASRGL1,ATIC,ATP2A2,ATP2B4,ATP6AP1,ATP6V1A,ATPAF2,ATXN10,BCAS1,BCAT2,BIRC6,CAMSAP2,CAND1,CANX,CARMIL1,CAVIN1,CDC37L1,CDK1,CDK4,CEP55,CNEP1R1,CNN2,CNPY2,CRAT,CSNK1A1,CSRP1,CTBP2,CTTNBP2,CUL3,CYB5R1,DDB1,DDRGK1,DDX5,DGKZ,DGLUCY,DNAJC5,DNMT1,DNPEP,DOCK1,DOCK5,DPY19L1,DPYSL2,DPYSL3,DYNC2H1,ECI2,EEF1B2,EEF2,EEF2K,EIF2B4,ELOA,ELP3,EML1,ERLIN2,EXOC2,EXTL3,FADD,FAF2,FASN,FBXL15,FLII,FLNA,FLNB,FRMD4A,FUT8,GATM,GCDH,GCLC,GDA,GMDS,GMPPB,GOT2,GPT2,GRB14,GTSE1,HAUS7,HDGFL3,HMGCL,HMGCS1,HMOX2,HNRNPUL1,IDH2,IKBIP,INPP1,INPPL1,IRGQ,ITPR1,ITPR3,ITPRID2,KANK1,KANK2,KATNB1,KPNA2,LARP7,LAYN,LETM1,LIMD1,LPP,LRRC40,LTA4H,MARK2,MAT2A,MEGF10,MKLN1,MMUT,MON2,MPI,MPST,MTSS1,NAA25,NAB2,NAPA,NAXE,NCLN,NEK9,NFKB2,NFS1,NPTXR,NQO1,NRDC,OAT,OSBPL8,PAK1,PAPSS1,PCBP2,PDE4DIP,PEPD,PGD,PHLDA1,PHYH,PIGU,PITRM1,PLCB1,POLA1,POLD1,POLD3,POLDIP3,PPOX,PPP4R3A,PRKACB,PRKCA,PRPF19,PRRC2A,PRRX1,QSOX2,RAB32,RAD51,RAF1,RAI14,RANGAP1,RASSF4,RBPJ,RIOK3,RPA1,RPL13,RPL3,RPL5,RPN1,RPN2,RRM1,RRM2,SAMD4B,SEC14L1,SEC24A,SEC24D,SEC61A1,SERPINE2,SFR1,SGTA,SLC1A4,SOAT1,SPP1,SQLE,SRGAP3,STARD3NL,STAT1,STAT3,STAT5B,STRN4,STT3A,SUCLG2,TAX1BP1,TCF12,TDRKH,TKFC,TLE3,TLN1,TM9SF4,TMEM43,TNS3,TOM1L2,TRIM2,TRIM28,TRIM3,TRIM47,TRIOBP,TRIP12,TRIP13,TRMT2A,TST,TTC21B,UBAP2L,UBE2O,UBR7,USP24,USP47,USP8,USP9X,VAMP7,VAT1,VCP,VPS11,VPS13A,VPS4B,VWA5A,WDR12,WDR45B,WDR62,WFS1,XRN1	250
Cancer,Organismal Injury and Abnormalities,Reproductive System Disease	Development of genital tumor	0,00000749			ABCD4,ACADS,ACSL3,ACSL4,ADD1,ADI1,AGL,AK3,AKR1B1,AKR7A2,ALDH2,ALDH4A1,AP3S2,APPL2,ARFGEF2,ARHGEF1,ARHGEF12,ARHGEF18,ASRGL1,ATIC,ATP6AP1,ATP6V1A,BCAS1,BIRC6,CAMSAP2,CAND1,CAVIN1,CBX3,CCDC47,CCZ1/CCZ1B,CDIPT,CDK4,CEP55,CTTNBP2,DDB1,DDRGK1,DGKZ,DGLUCY,DNMT1,DOCK1,DOCK5,DPYSL2,DTYMK,DYNC2H1,EEF1B2,EEF2,ELOA,ELP2,ELP3,EMC7,EMD,ERLIN2,EXOC2,EXOC4,FAF2,FBXO7,FDFT1,FLII,FLNA,FLNB,FRMD4A,FUT8,GATM,GCDH,GFPT1,GHDC,GLRX3,GMDS,GMPPB,GOT2,GPT2,HAUS7,HDGFL3,HMMR,HMOX1,HSPA4L,IDH2,IFT74,IKBIP,INPPL1,ITPR1,ITPR3,KPNA2,LARP7,LAYN,LETM1,LIMD1,LPP,LRRC40,MEGF10,MKLN1,MMUT,MON2,MTDH,MTSS1,NAA25,NAPA,NCLN,NFKB2,NPTXR,NQO1,NRDC,NUP54,OAT,OGT,OSBPL8,OXCT1,PAK1,PARG,PCBP2,PCK2,PDE4DIP,PDZRN3,PEPD,PFKM,PHLDA1,PHYH,PIGU,PITRM1,PKN2,PLCB1,POLA1,POLD1,POLD3,POLDIP3,PPAT,PPOX,PRKCA,PRPF19,PRRC2A,PTPN1,RAD51,RAF1,RANGAP1,RER1,RETSAT,RIOK3,RNASEH2B,RPL27,RPL5,RRM1,RRM2,SAMD4B,SDF4,SEC14L1,SEC61A1,SNF8,SNX1,SNX17,SOAT1,SORBS3,SPP1,STAT1,STAT3,STAT5B,STT3A,SUCLG2,TAX1BP1,TCF12,TKFC,TLN1,TMEM43,TNS3,TOM1L2,TRIM28,TRIM3,TRIOBP,TRIP12,TRIP13,TRMT2A,TST,UBE2C,USP24,USP8,USP9X,VCP,VCPIP1,VPS11,VPS13A,WFS1,WIPF1,XRN1,ZMPSTE24	183
Cancer,Neurological Disease,Organismal Injury and Abnormalities	Glioma cancer	0,00000761			ABCD4,ACADS,ACP2,ACSF2,ADD1,AGA,AGFG1,AIP,AKR1B1,AKR1B10,ALDH2,ALDH9A1,ANAPC7,AP3S2,APPL2,ARFGAP2,ARFGAP3,ARFGEF2,ARHGEF1,ARHGEF18,ARPIN/ARPIN-AP3S2,ASRGL1,ATIC,ATP2A2,ATP2B4,ATP6AP1,ATP6V1A,ATPAF2,ATXN10,BCAS1,BCAT2,BIRC6,CACYBP,CAMSAP2,CAND1,CANX,CARMIL1,CC2D1B,CCDC47,CDC42EP4,CDK4,CDK6,CEP55,CNN2,CNN3,CRAT,CSRP1,CTBP2,CTTNBP2,CUL3,CWF19L1,CYFIP1,DDB1,DDRGK1,DDX5,DNMT1,DNPEP,DOCK1,DOCK5,DPY19L1,DYNC2H1,EEF2K,EIF2B4,EIF2B5,ELP1,ELP2,ELP3,ERLIN2,EXOC4,FADD,FASN,FBXL15,FBXO7,FLII,FLNA,FLNB,FUT8,GABPA,GCLC,GCLM,GDA,GFPT1,GMDS,GMPPB,GPT2,GRB14,GSDMD,GTSE1,HDGFL3,HEBP2,HMGCS1,HMMR,HMOX1,IDH2,INPPL1,ITPR1,ITPR3,ITPRID2,KANK1,KANK2,KATNB1,KPNA2,LETM1,LPP,LRRC40,MARK2,MEGF10,MKLN1,MMUT,MON2,MPI,MTDH,MTSS1,NAA25,NCLN,NFKB2,NIT2,NLE1,NLN,NRBP1,NRDC,NT5C3B,OGT,OXCT1,PAFAH1B1,PAK1,PAPSS1,PCBP2,PCK2,PDE4DIP,PDZRN3,PEPD,PFKM,PHLDA1,PHYH,PIP4P2,PITRM1,PKN2,PLCB1,POLA1,POLD1,PPAT,PPP4R3A,PRRC2A,PTPN1,QSOX2,RAB32,RABGGTA,RAF1,RANGAP1,RASSF4,RBPJ,RPA1,RPL5,RPN1,RPN2,RRM1,RRM2,SAMD4B,SDF4,SEC14L1,SEC24A,SEC24D,SH3GLB1,SLC9A3R1,SNX1,SNX17,SOAT1,SORBS3,SPCS3,SPP1,SQSTM1,SRGAP3,STARD3NL,STAT1,STAT3,STAT5B,STRN4,STT3A,TAX1BP1,TCF12,TDRKH,TLE3,TLN1,TM9SF4,TMEM43,TNS3,TOM1L2,TPD52,TRIM2,TRIM28,TRIOBP,TRIP10,TRIP12,UBE2C,UBE2O,UBR7,USP24,USP47,USP8,USP9X,VCP,VCPIP1,VPS13A,VPS36,VWA5A,WDR62,WFS1,WIPF1,XRN1	210
Cancer,Organismal Injury and Abnormalities,Reproductive System Disease	Breast or ovarian cancer	0,00000762			ACACA,ACADS,ACP2,AGL,AK3,AKR1B1,AKR7A2,ARFGAP3,ARHGEF1,ARHGEF12,ARHGEF18,ASRGL1,BIRC6,C17orf49,C1QBP,CAND1,CANX,CAVIN1,CBX3,CCDC47,CDK1,CDK4,CDK6,CERT1,CNN2,CNPY2,CRAT,CSNK1A1,CTBP2,Cyb5r3,DDB1,DDX5,DGKZ,DGLUCY,DNMT1,DOCK5,DPYSL2,DYNC2H1,EEF1B2,ELOA,ELP1,ELP2,ELP3,EMD,ERLIN2,EXOC4,FADD,FASN,FBXO7,FDFT1,FLII,FLNA,FLNB,GABPA,GCDH,GMDS,GMPPB,GOT2,GTSE1,HDGFL3,HMGCS1,HMMR,HMOX1,HNRNPUL1,INPPL1,ITPR1,KPNA2,LETM1,LOXL3,MARK2,MGST2,MMUT,MTDH,NAA25,NAB2,NCLN,NDC80,NEK9,NFKB2,NPTXR,NQO1,OSBPL8,OXCT1,PAK1,PCK2,PDE4DIP,PEPD,PHLDA1,PITRM1,PKN2,PLCB1,POLD1,POLDIP3,PPOX,PRKCA,PRRX1,PTPN1,RAD51,RAF1,RANGAP1,RIOK3,RNASEH2B,RPL5,RPL6,RPN1,RRM1,RRM2,SAMD4B,SEC14L1,SEC61A1,SLC9A3R1,SNX17,SPP1,SQLE,STAT1,STAT3,STAT5B,SUCLG2,TAGLN2,TLE3,TLN1,TMEM43,TNS3,TPD52,TRIM28,TRIM3,TRIP10,TRIP12,TRIP13,TRMT2A,UBAP2L,UBE2C,USP24,USP47,USP9X,VCPIP1,VPS13A,VWA5A,WFS1,WIPF1,ZMPSTE24	141
Developmental Disorder,Hereditary Disorder,Neurological Disease,Organismal Injury and Abnormalities	Recessive mental retardation	0,00000762			EEF1B2,ELP2,ERLIN2,FASN,GATM,GMPPB,GPT2,IMPA1,LARP7,NARS1,OGT,POLA1,STRN4,UBR7,USP9X,WDR45B,WDR62	17
Cancer,Hematological Disease,Immunological Disease,Organismal Injury and Abnormalities	De novo unfavorable cytogenetic risk acute myeloid leukemia	0,00000808			DNMT1,POLA1,POLD1,RRM1,RRM2	5
Cancer,Hematological Disease,Immunological Disease,Organismal Injury and Abnormalities	Favorable-risk acute myeloid leukemia	0,00000808			IDH2,POLA1,POLD1,RRM1,RRM2	5
Cancer,Hematological Disease,Immunological Disease,Organismal Injury and Abnormalities	PML-RARA negative acute promyelocytic leukemia	0,00000822			DNMT1,POLA1,POLD1,RRM1	4
Cardiovascular Disease,Hematological Disease,Organismal Injury and Abnormalities	Sideroblastic anemia	0,00000824			DNMT1,GLRX5,IDH2,POLA1,POLD1,PPAT,RRM1,RRM2	8
Cancer,Hematological Disease,Immunological Disease,Organismal Injury and Abnormalities	FLT3 internal tandem duplication positive acute myeloid leukemia	0,00000899			DNMT1,POLA1,POLD1,PRKCA,RAF1,RRM1,RRM2	7
Cancer,Hematological Disease,Immunological Disease,Organismal Injury and Abnormalities	Type M0 acute myeloid leukemia	0,00000943			IDH2,POLA1,POLD1,PPAT,RRM1,RRM2	6
Cancer,Hematological Disease,Immunological Disease,Organismal Injury and Abnormalities	C-MYC rearrangement positive B-cell lymphoma	0,0000104			ARHGEF1,DNMT1,POLA1,POLD1,RRM1,RRM2,STAT3	7
Cancer,Neurological Disease,Organismal Injury and Abnormalities	Central nervous system cancer	0,0000117			ABCD4,ACADS,ACP2,ACSF2,ADD1,AGA,AGFG1,AIP,AKR1B1,AKR1B10,ALDH2,ALDH9A1,ANAPC7,AP3S2,APPL2,ARFGAP2,ARFGAP3,ARFGEF2,ARHGEF1,ARHGEF18,ARPIN/ARPIN-AP3S2,ASRGL1,ATIC,ATP2A2,ATP2B4,ATP6AP1,ATP6V1A,ATPAF2,ATXN10,BCAS1,BCAT2,BIRC6,CACYBP,CAMSAP2,CAND1,CANX,CARMIL1,CC2D1B,CCDC47,CDC42EP4,CDK4,CDK6,CEP55,CNN2,CNN3,CRAT,CSRP1,CTBP2,CTTNBP2,CUL3,CWF19L1,CYFIP1,DDB1,DDRGK1,DDX5,DGKZ,DNMT1,DNPEP,DOCK1,DOCK5,DPY19L1,DYNC2H1,EEF2K,EIF2B4,EIF2B5,ELP1,ELP2,ELP3,ERLIN2,EXOC4,FADD,FASN,FBXL15,FBXO7,FLII,FLNA,FLNB,FUT8,GABPA,GCLC,GCLM,GDA,GFPT1,GMDS,GMPPB,GOT2,GPT2,GRB14,GSDMD,GTSE1,HDGFL3,HEBP2,HMGCS1,HMMR,HMOX1,IDH2,INPPL1,ITPR1,ITPR3,ITPRID2,KANK1,KANK2,KATNB1,KPNA2,LETM1,LPP,LRRC40,MARK2,MEGF10,MKLN1,MMUT,MON2,MPI,MTDH,MTSS1,NAA25,NCLN,NFKB2,NIT2,NLE1,NLN,NRBP1,NRDC,NT5C3B,OGT,OXCT1,PAFAH1B1,PAK1,PAPSS1,PCBP2,PCK2,PDE4DIP,PDZRN3,PEPD,PFKM,PHLDA1,PHYH,PIP4P2,PITRM1,PKN2,PLCB1,POLA1,POLD1,PPAT,PPP4R3A,PRRC2A,PTPN1,QSOX2,RAB32,RABGGTA,RAF1,RANGAP1,RASSF4,RBPJ,RPA1,RPL5,RPN1,RPN2,RRM1,RRM2,SAMD4B,SDF4,SEC14L1,SEC24A,SEC24D,SH3GLB1,SLC9A3R1,SNX1,SNX17,SOAT1,SORBS3,SPCS3,SPP1,SQSTM1,SRGAP3,STARD3NL,STAT1,STAT3,STAT5B,STRN4,STT3A,TAX1BP1,TCF12,TDRKH,TLE3,TLN1,TM9SF4,TMEM43,TNS3,TOM1L2,TPD52,TRIM2,TRIM28,TRIOBP,TRIP10,TRIP12,UBE2C,UBE2O,UBR7,USP24,USP47,USP8,USP9X,VCP,VCPIP1,VPS13A,VPS36,VWA5A,WDR62,WFS1,WIPF1,XRN1	212
Cancer,Hematological Disease,Immunological Disease,Organismal Injury and Abnormalities	Refractory acute myeloid leukemia	0,0000124			DNMT1,IDH2,POLA1,POLD1,RAF1,RPL3,RPL6,RRM1,RRM2	9
Cancer,Organismal Injury and Abnormalities,Reproductive System Disease	Female genital tract cancer	0,0000133			ABCD4,ACADS,ACSL3,ACSL4,ADD1,ADI1,AGL,AK3,AKR1B1,AKR7A2,ALDH2,ALDH4A1,AP3S2,APPL2,ARFGEF2,ARHGEF1,ARHGEF12,ARHGEF18,ASRGL1,ATIC,ATP6AP1,ATP6V1A,BCAS1,BIRC6,CAMSAP2,CAND1,CAVIN1,CBX3,CCDC47,CCZ1/CCZ1B,CDIPT,CDK1,CDK4,CEP55,CTTNBP2,DDB1,DDRGK1,DGKZ,DGLUCY,DNMT1,DOCK1,DOCK5,DPYSL2,DTYMK,DYNC2H1,EEF1B2,EEF2,ELOA,ELP2,ELP3,EMC7,EMD,ERLIN2,EXOC2,EXOC4,FAF2,FBXO7,FDFT1,FLII,FLNA,FLNB,FRMD4A,FUT8,GATM,GCDH,GFPT1,GHDC,GLRX3,GMDS,GMPPB,GOT2,GPT2,HAUS7,HDGFL3,HMMR,HMOX1,HSPA4L,IDH2,IFT74,IKBIP,INPPL1,ITPR1,ITPR3,KPNA2,LARP7,LAYN,LETM1,LIMD1,LPP,LRRC40,MEGF10,MKLN1,MMUT,MON2,MTDH,MTSS1,NAA25,NAPA,NCLN,NFKB2,NPTXR,NQO1,NRDC,NUP54,OAT,OGT,OSBPL8,OXCT1,PARG,PCBP2,PCK2,PDE4DIP,PDZRN3,PEPD,PFKM,PHLDA1,PHYH,PIGU,PITRM1,PKN2,PLCB1,POLA1,POLD1,POLD3,POLDIP3,PPAT,PPOX,PRKCA,PRPF19,PRRC2A,PTPN1,RAD51,RAF1,RANGAP1,RER1,RETSAT,RIOK3,RNASEH2B,RPL5,RRM1,RRM2,SAMD4B,SDF4,SEC14L1,SEC61A1,SNF8,SNX1,SNX17,SOAT1,SORBS3,SPP1,STAT1,STAT3,STAT5B,STT3A,SUCLG2,TAX1BP1,TCF12,TKFC,TLN1,TMEM43,TNS3,TOM1L2,TPD52,TRIM28,TRIM3,TRIOBP,TRIP12,TRIP13,TRMT2A,TST,UBE2C,USP24,USP8,USP9X,VCPIP1,VPS11,VPS13A,WFS1,WIPF1,XRN1,ZMPSTE24	182
Cancer,Hematological Disease,Immunological Disease,Organismal Injury and Abnormalities	Mixed phenotype T cell/myeloid acute leukemia	0,0000135			DNMT1,POLA1,POLD1,RPL3	4
Cancer,Hematological Disease,Immunological Disease,Organismal Injury and Abnormalities	P53 mutation positive acute myeloid leukemia	0,0000135			POLA1,POLD1,RRM1,RRM2	4
Carbohydrate Metabolism	Metabolism of glycosides	0,0000138			AKR1B1,AKR1B10,AKR7A2,FUT8,GFPT1,GMDS	6

Categories	Diseases or Functions Annotation	p-value	Predicted Activation State	Activation z-score	Molecules	# Molecules
Lipid Metabolism,Nucleic Acid Metabolism,Small Molecule Biochemistry	Metabolism of acyl-coenzyme A	0,0000138			ACAA2,ACACA,ACSF2,FASN,GCDH,HMGCL,SUCLG2	7
Cancer,Hematological Disease,Immunological Disease,Organismal Injury and Abnormalities	5q deletion acute myeloid leukemia	0,0000142			DNMT1,POLA1,POLD1,RRM1,RRM2	5
Cancer,Organismal Injury and Abnormalities,Reproductive System Disease	Female genital tract adenocarcinoma	0,0000146			ABCD4,ACADS,ACSL3,ACSL4,ADD1,AGL,AK3,AKR1B1,ALDH2,ALDH4A1,AP3S2,APPL2,ARFGEF2,ARHGEF1,ARHGEF12,ARHGEF18,ASRGL1,ATIC,ATP6AP1,BIRC6,CAMSAP2,CAND1,CAVIN1,CBX3,CCDC47,CDIPT,CEP55,CTTNBP2,DDB1,DGKZ,DGLUCY,DNMT1,DOCK1,DOCK5,DPYSL2,DTYMK,DYNC2H1,EEF1B2,EEF2,ELOA,ELP2,ELP3,EMC7,EMD,ERLIN2,EXOC2,EXOC4,FAF2,FBXO7,FDFT1,FLII,FLNA,FLNB,FRMD4A,FUT8,GATM,GCDH,GFPT1,GLRX3,GMDS,GMPPB,GOT2,GPT2,HAUS7,HDGFL3,HMMR,HSPA4L,IDH2,IFT74,IKBIP,INPPL1,ITPR1,ITPR3,KPNA2,LARP7,LETM1,LPP,LRRC40,MEGF10,MKLN1,MMUT,MON2,MTDH,MTSS1,NAA25,NAPA,NCLN,NFKB2,NPTXR,NQO1,NRDC,NUP54,OAT,OGT,OSBPL8,OXCT1,PARG,PCBP2,PCK2,PDE4DIP,PDZRN3,PEPD,PFKM,PHLDA1,PHYH,PIGU,PITRM1,PKN2,PLCB1,POLA1,POLD1,POLD3,POLDIP3,PPAT,PPOX,PRKCA,PRPF19,PRRC2A,PTPN1,RAD51,RAF1,RANGAP1,RER1,RETSAT,RIOK3,RNASEH2B,RPL5,RRM1,RRM2,SAMD4B,SEC14L1,SEC61A1,SNF8,SNX17,SOAT1,SORBS3,SPP1,STAT1,STAT3,STT3A,SUCLG2,TAX1BP1,TCF12,TKFC,TLN1,TMEM43,TNS3,TOM1L2,TRIM28,TRIM3,TRIOBP,TRIP12,TRIP13,TRMT2A,UBE2C,USP24,USP8,USP9X,VPS11,VPS13A,WFS1,WIPF1,XRN1,ZMPSTE24	164
Cancer,Organismal Injury and Abnormalities	Breast or colorectal cancer	0,0000157			ACADS,ACO2,ACP2,ACSL4,ADD1,AGL,AK3,AKR1B1,AKR1B10,AKR7A2,ALDH2,ALDH6A1,ANXA4,APPL2,ARFGAP2,ARFGAP3,ARHGEF1,ARHGEF18,ARMC8,ASRGL1,ATIC,ATP2A2,ATXN10,BCAS1,BCAT2,BIRC6,C17orf49,C1QBP,CACYBP,CANX,CAVIN1,CBX3,CDK1,CDK4,CDK6,CEP55,CERT1,CNN2,CNPY2,CRAT,CSNK1A1,CTBP2,CTTNBP2,CYB5R1,Cyb5r3,DCTN6,DDB1,DDX5,DGKZ,DGLUCY,DNAJC5,DNMT1,DOCK1,DOCK5,DPY19L1,DPYSL2,DYNC2H1,EEF1B2,ELOA,ELP1,ELP3,EMC7,ERLIN2,EXOC2,EXOC4,EXTL3,FADD,FAF2,FASN,FDFT1,FLII,FLNA,FLNB,FRMD4A,GABPA,GCDH,GCLM,GHDC,GLRX,GLRX3,GMPPB,GRB14,GSTZ1,GTF2B,GTSE1,HDGFL3,HEBP2,HM13,HMGCS1,HMMR,HMOX1,HNRNPUL1,IDH2,IMPA1,INPPL1,IRGQ,ISCA2,ITPR1,ITPR3,ITPRID2,KANK1,KPNA2,LARP7,LETM1,LIMD1,LOXL3,MARK2,MAT2A,MEGF10,MGST2,MON2,MTDH,NAA25,NAB2,NCLN,NDC80,NEK9,NLE1,NQO1,NRDC,NT5C3B,NUP54,OXCT1,PAK1,PAPSS1,PCBP2,PCK2,PDE4DIP,PDZRN3,PHLDA1,PIGU,PITRM1,PKN2,PLCB1,POLA1,POLD1,POLD3,POLDIP3,PPOX,PPP2R5D,PPP4R3A,PRKACB,PRKCA,PRRC2A,PRRX1,PTPN1,RAD51,RAF1,RANGAP1,RETSAT,RIOK3,RNASEH2B,RPA1,RPL5,RPL6,RRM1,RRM2,SAMD4B,SEC14L1,SEC24A,SEC24D,SEC61A1,SERPINE2,SLC1A4,SLC9A3R1,SLC9A3R2,SNX1,SNX17,SOAT1,SPP1,SQLE,SQSTM1,STAT1,STAT3,STAT5B,TAGLN2,TAX1BP1,TCF12,TLE3,TLN1,TM9SF3,TM9SF4,TMEM115,TMEM43,TNS3,TPD52,TRIM28,TRIM3,TRIM47,TRIOBP,TRIP10,TRIP12,TRIP13,TRMT2A,UBAP2L,UBE2C,UCKL1,USP24,USP47,USP9X,VCP,VCPIP1,VPS13A,VPS36,VWA5A,WDR45B,WDR62,WFS1,WIPF1,XRN1,ZMPSTE24	211
Cancer,Cardiovascular Disease,Hematological Disease,Organismal Injury and Abnormalities	Myelodysplastic syndrome with ring sideroblasts	0,0000159			DNMT1,IDH2,POLA1,POLD1,PPAT,RRM1,RRM2	7
Cancer,Organismal Injury and Abnormalities,Reproductive System Disease	Female genital neoplasm	0,0000169			ABCD4,ACADS,ACSL3,ACSL4,ADD1,ADI1,AGL,AK3,AKR1B1,AKR7A2,ALDH2,ALDH4A1,AP3S2,APPL2,ARFGEF2,ARHGEF1,ARHGEF12,ARHGEF18,ASRGL1,ATIC,ATP6AP1,ATP6V1A,BCAS1,BIRC6,CAMSAP2,CAND1,CAVIN1,CBX3,CCDC47,CCZ1/CCZ1B,CDIPT,CDK1,CDK4,CEP55,CTTNBP2,DDB1,DDRGK1,DGKZ,DGLUCY,DNMT1,DOCK1,DOCK5,DPYSL2,DTYMK,DYNC2H1,EEF1B2,EEF2,ELOA,ELP2,ELP3,EMC7,EMD,ERLIN2,EXOC2,EXOC4,FAF2,FBXO7,FDFT1,FLII,FLNA,FLNB,FRMD4A,FUT8,GATM,GCDH,GFPT1,GHDC,GLRX3,GMDS,GMPPB,GOT2,GPT2,HAUS7,HDGFL3,HMMR,HMOX1,HSPA4L,IDH2,IFT74,IKBIP,INPPL1,ITPR1,ITPR3,KPNA2,LARP7,LAYN,LETM1,LIMD1,LPP,LRRC40,MEGF10,MKLN1,MMUT,MON2,MTDH,MTSS1,NAA25,NAPA,NCLN,NFKB2,NPTXR,NQO1,NRDC,NUP54,OAT,OGT,OSBPL8,OXCT1,PAK1,PARG,PCBP2,PCK2,PDE4DIP,PDZRN3,PEPD,PFKM,PHLDA1,PHYH,PIGU,PITRM1,PKN2,PLCB1,POLA1,POLD1,POLD3,POLDIP3,PPAT,PPOX,PRKCA,PRPF19,PRRC2A,PTPN1,RAD51,RAF1,RANGAP1,RER1,RETSAT,RIOK3,RNASEH2B,RPL27,RPL5,RRM1,RRM2,SAMD4B,SDF4,SEC14L1,SEC61A1,SNF8,SNX1,SNX17,SOAT1,SORBS3,SPP1,STAT1,STAT3,STAT5B,STT3A,SUCLG2,TAX1BP1,TCF12,TKFC,TLN1,TMEM43,TNS3,TOM1L2,TPD52,TRIM28,TRIM3,TRIOBP,TRIP12,TRIP13,TRMT2A,TST,UBE2C,USP24,USP8,USP9X,VCP,VCPIP1,VPS11,VPS13A,WFS1,WIPF1,XRN1,ZMPSTE24	185
Cancer,Hematological Disease,Immunological Disease,Organismal Injury and Abnormalities	PDGBR rearrangement negative Philadelphia chromosome negative t(5;12)(q33;p13) negative chronic myelomonocytic leukemia	0,000017			DNMT1,RRM1,RRM2	3
Cancer,Hematological Disease,Immunological Disease,Organismal Injury and Abnormalities	Cytogenetic abnormality positive loss of Y chromosome negative Philadelphia chromosome negative t(5;12)(q33;p13) negative CMML	0,000017			DNMT1,RRM1,RRM2	3
Cancer,Hematological Disease,Immunological Disease,Organismal Injury and Abnormalities	Relapsed angioimmunoblastic T-cell lymphoma	0,000017			DNMT1,RRM1,RRM2	3
Cancer,Hematological Disease,Immunological Disease,Organismal Injury and Abnormalities	Myeloproliferative chronic myelomonocytic leukemia	0,000017			DNMT1,RRM1,RRM2	3
Cancer,Hematological Disease,Immunological Disease,Organismal Injury and Abnormalities	Refractory angioimmunoblastic T-cell lymphoma	0,000017			DNMT1,RRM1,RRM2	3
Cancer,Organismal Injury and Abnormalities	Multiple cancers	0,0000173			ACACA,ACADS,ACP2,AGL,AK3,AKR1B1,AKR7A2,ARFGAP3,ARHGEF1,ARHGEF12,ARHGEF18,ASRGL1,BIRC6,C17orf49,C1QBP,CAND1,CANX,CAVIN1,CBX3,CCDC47,CDK1,CDK4,CDK6,CERT1,CNN2,CNPY2,CRAT,CSNK1A1,CTBP2,Cyb5r3,DDB1,DDX5,DGKZ,DGLUCY,DNMT1,DOCK5,DPYSL2,DYNC2H1,EEF1B2,ELOA,ELP1,ELP2,ELP3,EMD,ERLIN2,EXOC4,FADD,FASN,FBXO7,FDFT1,FLII,FLNA,FLNB,GABPA,GCDH,GMDS,GMPPB,GOT2,GTSE1,HDGFL3,HMGCS1,HMMR,HMOX1,HNRNPUL1,IDH2,INPPL1,ITPR1,KPNA2,LETM1,LOXL3,MARK2,MGST2,MMUT,MTDH,NAA25,NAB2,NCLN,NDC80,NEK9,NFKB2,NPTXR,NQO1,OSBPL8,OXCT1,PAK1,PCK2,PDE4DIP,PEPD,PHLDA1,PITRM1,PKN2,PLCB1,POLA1,POLD1,POLDIP3,PPOX,PRKCA,PRRX1,PTPN1,RAD51,RAF1,RANGAP1,RIOK3,RNASEH2B,RPL5,RPL6,RPN1,RRM1,RRM2,SAMD4B,SEC14L1,SEC61A1,SLC9A3R1,SNX17,SPP1,SQLE,STAT1,STAT3,STAT5B,SUCLG2,TAGLN2,TLE3,TLN1,TMEM43,TNS3,TPD52,TRIM28,TRIM3,TRIP10,TRIP12,TRIP13,TRMT2A,UBAP2L,UBE2C,USP24,USP47,USP9X,VCPIP1,VPS13A,VWA5A,WFS1,WIPF1,ZMPSTE24	143
Cell Cycle	G2 phase	0,0000184			CDK1,CDK4,DDB1,DNMT1,EEF2K,FADD,FASN,FBXL15,FLNA,HAUS7,MTDH,OGT,PAFAH1B1,PAK1,PHLDA1,PLCB1,PRPF19,RAD51,RAF1,RNASEH2B,RPA1,RPL7A	22
Nucleic Acid Metabolism,Small Molecule Biochemistry	Synthesis of nucleoside diphosphates	0,0000184			AK3,DTYMK,GMDS,GMPPB,MPI	5
Cancer,Hematological Disease,Immunological Disease,Organismal Injury and Abnormalities	Newly diagnosed type M3 acute myeloid leukemia	0,0000184			POLA1,POLD1,PPAT,RRM1,RRM2	5
Cancer,Hematological Disease,Immunological Disease,Organismal Injury and Abnormalities	Acute refractory myeloid leukemia	0,0000184			DNMT1,POLA1,POLD1,RRM1,RRM2	5
Cancer,Hematological Disease,Immunological Disease,Organismal Injury and Abnormalities	Refractory high-grade B-cell lymphoma	0,0000184			DNMT1,POLA1,POLD1,RRM1,RRM2	5
Cancer,Hematological Disease,Immunological Disease,Organismal Injury and Abnormalities	De novo FAB M4 acute myeloid leukemia	0,000021			POLA1,POLD1,RRM1,RRM2	4
Cancer,Hematological Disease,Immunological Disease,Organismal Injury and Abnormalities	De novo FAB M0 acute myeloid leukemia	0,000021			POLA1,POLD1,RRM1,RRM2	4
Cancer,Hematological Disease,Immunological Disease,Organismal Injury and Abnormalities	De novo FAB M2 acute myeloid leukemia	0,000021			POLA1,POLD1,RRM1,RRM2	4
Developmental Disorder,Neurological Disease,Organismal Injury and Abnormalities	Neuronal heterotopia	0,000021			ARFGEF2,EML1,FLNA,PAFAH1B1	4
Cancer,Hematological Disease,Immunological Disease,Organismal Injury and Abnormalities	De novo FAB M1 acute myeloid leukemia	0,000021			POLA1,POLD1,RRM1,RRM2	4
Cancer,Hematological Disease,Immunological Disease,Organismal Injury and Abnormalities	De novo FAB M5 acute myeloid leukemia	0,000021			POLA1,POLD1,RRM1,RRM2	4
Developmental Disorder,Hereditary Disorder,Neurological Disease,Organismal Injury and Abnormalities	Familial mental retardation	0,0000214			ACSL4,CERT1,CUL3,EEF1B2,ELP2,ERLIN2,FASN,FLNA,GATM,GMPPB,GPT2,IMPA1,LARP7,NARS1,OGT,PAK1,POLA1,PPP2R5D,SLC1A4,STRN4,TRIP12,UBR7,USP9X,WDR45B,WDR62	25
Developmental Disorder,Neurological Disease,Organismal Injury and Abnormalities	Mental retardation	0,000022			ACSL4,AGA,ALDH4A1,ARFGEF2,ATP6V1A,CERT1,CUL3,CWF19L1,CYFIP1,DYNC2H1,EEF1B2,ELP2,ERLIN2,FASN,FLNA,GATM,GMPPB,GPT2,IMPA1,ITPR1,LARP7,NARS1,OGT,PAFAH1B1,PAK1,POLA1,POLD1,PPP2R5D,SLC1A4,STRN4,TRIP12,UBR7,USP9X,VCP,WDR45B,WDR62	36
Cancer,Hematological Disease,Organismal Injury and Abnormalities	Myeloid sarcoma	0,0000233			IDH2,POLA1,POLD1,RAF1,RRM1,RRM2	6
Cancer,Organismal Injury and Abnormalities,Reproductive System Disease	Tumorigenesis of reproductive tract	0,0000242			ABCD4,ACADS,ACSL3,ACSL4,ADD1,ADI1,AGL,AK3,AKR1B1,AKR7A2,ALDH2,ALDH4A1,AP3S2,APPL2,ARFGEF2,ARHGEF1,ARHGEF12,ARHGEF18,ASRGL1,ATIC,ATP6AP1,ATP6V1A,BCAS1,BIRC6,CAMSAP2,CAND1,CAVIN1,CBX3,CCDC47,CCZ1/CCZ1B,CDIPT,CDK1,CDK4,CEP55,CTTNBP2,DDB1,DDRGK1,DGKZ,DGLUCY,DNMT1,DOCK1,DOCK5,DPYSL2,DTYMK,DYNC2H1,EEF1B2,EEF2,ELOA,ELP2,ELP3,EMC7,EMD,ERLIN2,EXOC2,EXOC4,FAF2,FBXO7,FDFT1,FLII,FLNA,FLNB,FRMD4A,FUT8,GATM,GCDH,GFPT1,GHDC,GLRX3,GMDS,GMPPB,GOT2,GPT2,HAUS7,HDGFL3,HMMR,HMOX1,HSPA4L,IDH2,IFT74,IKBIP,INPPL1,ITPR1,ITPR3,KPNA2,LARP7,LAYN,LETM1,LIMD1,LPP,LRRC40,MEGF10,MKLN1,MMUT,MON2,MTDH,MTSS1,NAA25,NAPA,NCLN,NFKB2,NPTXR,NQO1,NRDC,NUP54,OAT,OGT,OSBPL8,OXCT1,PARG,PCBP2,PCK2,PDE4DIP,PDZRN3,PEPD,PFKM,PHLDA1,PHYH,PIGU,PITRM1,PKN2,PLCB1,POLA1,POLD1,POLD3,POLDIP3,PPAT,PPOX,PRKCA,PRPF19,PRRC2A,PTPN1,RAD51,RAF1,RANGAP1,RER1,RETSAT,RIOK3,RNASEH2B,RPL27,RPL5,RRM1,RRM2,SAMD4B,SDF4,SEC14L1,SEC61A1,SNF8,SNX1,SNX17,SOAT1,SORBS3,SPP1,STAT1,STAT3,STAT5B,STT3A,SUCLG2,TAX1BP1,TCF12,TKFC,TLN1,TMEM43,TNS3,TOM1L2,TPD52,TRIM28,TRIM3,TRIOBP,TRIP12,TRIP13,TRMT2A,TST,UBE2C,USP24,USP8,USP9X,VCP,VCPIP1,VPS11,VPS13A,WFS1,WIPF1,XRN1,ZMPSTE24	184
Cancer,Organismal Injury and Abnormalities	Primary tumor	0,0000253			ATIC,CDK4,CDK6,CSNK1A1,CTBP2,DGKZ,DNMT1,DOCK1,FASN,FLNA,GOT2,IDH2,KANK2,KPNA2,MTDH,PAFAH1B1,POLA1,POLD1,RAF1,RRM1,RRM2,SH3GLB1,SLC9A3R1,SPP1,STAT3	25
Cancer,Organismal Injury and Abnormalities	Refractory tumor	0,0000284			ATIC,CDK4,CDK6,DNMT1,FDFT1,HMOX1,IDH2,POLA1,POLD1,PPAT,RAF1,RPL3,RPL6,RRM1,RRM2,SPP1	16
Developmental Disorder,Hereditary Disorder,Metabolic Disease,Organismal Injury and Abnormalities	Inborn error of amino acid metabolism	0,0000289			ABCD4,ALDH4A1,ALDH6A1,BCAT2,GATM,GCDH,GLRX5,HMGCL,LOXL3,MMUT,SUCLG2	11
Neurological Disease,Organismal Injury and Abnormalities	Syndromic encephalopathy	0,0000293			CUL3,DNMT1,FLNA,GATM,GMPPB,GOT2,HMOX1,IFT74,ITPR1,LARP7,NARS1,OGT,PAK1,PDE4DIP,PIGU,PLCB1,POLA1,PPAT,PRKCA,RAF1,RNASEH2B,SERPINE2,SLC1A4,STRN4,SUCLG2,TRAPPC4,TTC21B,UBR7,USP9X,VARS1,VPS13A,WDR45B,WDR62	33
Cancer,Hematological Disease,Immunological Disease,Organismal Injury and Abnormalities	M6 childhood acute erythroid leukemia	0,0000297			POLA1,POLD1,PPAT,RRM1,RRM2	5
Cancer,Hematological Disease,Immunological Disease,Organismal Injury and Abnormalities	M5a childhood acute myeloid leukemia	0,0000297			POLA1,POLD1,PPAT,RRM1,RRM2	5
Cancer,Hematological Disease,Immunological Disease,Organismal Injury and Abnormalities	M5b childhood acute monocytic leukemia	0,0000297			POLA1,POLD1,PPAT,RRM1,RRM2	5
Cancer,Hematological Disease,Immunological Disease,Organismal Injury and Abnormalities	M2 childhood acute myeloid leukemia	0,0000297			POLA1,POLD1,PPAT,RRM1,RRM2	5
Cancer,Hematological Disease,Immunological Disease,Organismal Injury and Abnormalities	M7 childhood acute myeloid leukemia	0,0000297			POLA1,POLD1,PPAT,RRM1,RRM2	5
Cancer,Hematological Disease,Immunological Disease,Organismal Injury and Abnormalities	M1 childhood acute myeloid leukemia	0,0000297			POLA1,POLD1,PPAT,RRM1,RRM2	5
Cancer,Hematological Disease,Immunological Disease,Organismal Injury and Abnormalities	M4 childhood acute myeloid leukemia	0,0000297			POLA1,POLD1,PPAT,RRM1,RRM2	5
Cancer,Hematological Disease,Immunological Disease,Organismal Injury and Abnormalities	Recurrent acute leukemia	0,00003			DNMT1,IDH2,POLA1,POLD1,PPAT,RAF1,RPL3,RRM1,RRM2	9
Cancer,Organismal Injury and Abnormalities,Reproductive System Disease	Stage II-III breast cancer	0,0000301			CBX3,CDK4,CDK6,DPYSL2,FDFT1,HMOX1,RRM1,RRM2,SLC9A3R1,SQLE	10
Cancer,Organismal Injury and Abnormalities,Reproductive System Disease	Stage I-III mammary tumor	0,0000301			CBX3,CDK4,CDK6,DPYSL2,FDFT1,HMOX1,RRM1,RRM2,SLC9A3R1,SQLE	10
Cancer,Organismal Injury and Abnormalities	Refractory metastasis	0,0000303			ATIC,CDK4,CDK6,POLA1,POLD1,RAF1,RRM1,RRM2	8
Cancer,Hematological Disease,Immunological Disease,Organismal Injury and Abnormalities	Refractory CD20 positive diffuse large B-cell non-Hodgkin lymphoma	0,0000311			POLA1,POLD1,RRM1,RRM2	4
Cancer,Hematological Disease,Immunological Disease,Organismal Injury and Abnormalities	De novo FAB M6 acute myeloid leukemia	0,0000311			POLA1,POLD1,RRM1,RRM2	4
Cancer,Cardiovascular Disease,Hematological Disease,Organismal Injury and Abnormalities	Refractory anemia with excess blasts type 1	0,0000311			DNMT1,POLA1,RRM1,RRM2	4
Developmental Disorder,Organismal Injury and Abnormalities	Global developmental delay	0,0000321			ARFGEF2,CCDC47,CUL3,CYFIP1,DDX5,GMPPB,GOT2,KANK2,NARS1,PEPD,PPP2R5D,RNASEH2B,TRAPPC4,UBAP2L,UBR7,USP9X	16
Cancer,Hematological Disease,Organismal Injury and Abnormalities	Secondary myelodysplastic syndrome	0,0000321			DNMT1,POLA1,POLD1,PPAT,RRM1,RRM2	6
Amino Acid Metabolism,Small Molecule Biochemistry	Metabolism of alpha-amino acid	0,0000323			ALDH4A1,ASRGL1,DGLUCY,GCDH,GCLC,GCLM,GOT2,GPT2,GSTZ1,NIT2	10

Categories	Diseases or Functions Annotation	p-value	Predicted Activation State	Activation z-score	Molecules	# Molecules
Cancer,Organismal Injury and Abnormalities	Development of adenocarcinoma	0,0000327			ABCD4,ACADS,ACO1,ACSL3,ACSL4,ADD1,AGL,AK3,AKR1B1,ALDH2,ALDH4A1,ANKRD13A,AP3S2,APPL2,ARFGAP2,ARFGEF2,ARHGEF1,ARHGEF12,ARHGEF18,ASRGL1,ATIC,ATP2A2,ATP6AP1,BIRC6,C1QBP,CAMSAP2,CAND1,CAVIN1,CBX3,CC2D1B,CCDC47,CDIPT,CDK1,CDK4,CEP55,CERT1,CTTNBP2,CUL3,DDB1,DDRGK1,DGKZ,DGLUCY,DNMT1,DOCK1,DOCK5,DPY19L1,DPYSL2,DTYMK,DYNC2H1,EEF1B2,EEF2,ELOA,ELP2,ELP3,EMC7,EMD,ERLIN2,EXOC2,EXOC4,FAF2,FBXO7,FDFT1,FLII,FLNA,FLNB,FRMD4A,FUT8,GATM,GCDH,GFPT1,GLRX3,GLRX5,GMDS,GMPPB,GOT2,GPT2,HAUS7,HDGFL3,HMMR,HSPA4L,IDH2,IFT74,IKBIP,INPPL1,ITPR1,ITPR3,KPNA2,LARP7,LETM1,LPP,LRRC40,LTA4H,MARK2,MEGF10,MKLN1,MMUT,MON2,MTDH,MTSS1,NAA25,NAPA,NCLN,NDC80,NFKB2,NMD3,NPTXR,NQO1,NRDC,NUP54,OAT,OGT,OSBPL8,OXCT1,PAFAH1B1,PARG,PCBP2,PCK2,PDE4DIP,PEPD,PFKM,PHLDA1,PHYH,PIGU,PITRM1,PKN2,PLCB1,POLA1,POLD1,POLD3,POLDIP3,PPAT,PPOX,PPP4R3A,PRDX2,PRKCA,PRPF19,PRRC2A,PTPN1,QSOX2,RAB32,RAD51,RAF1,RAI14,RANGAP1,RCN2,RER1,RETSAT,RIOK3,RNASEH2B,RPA1,RPL5,RRM1,RRM2,SAMD4B,SEC14L1,SEC24A,SEC61A1,SLC1A4,SLC9A3R1,SNF8,SNX17,SOAT1,SORBS3,SPP1,SRGAP3,STAT1,STAT3,STT3A,SUCLG2,TAX1BP1,TCF12,TKFC,TLN1,TMEM43,TNS3,TOM1L2,TRIM2,TRIM28,TRIM3,TRIOBP,TRIP12,TRMT2A,UBAP2L,UBE2C,USP24,USP8,USP9X,VPS11,VPS13A,WDR62,WFS1,WIPF1,XRN1,ZMPSTE24	194
Cancer,Organismal Injury and Abnormalities	Breast or gastric cancer	0,0000333			AARSD1,ACACA,ACADS,ACO1,ACP2,ACSF2,ACSL3,AGL,AK3,AKR1B1,AKR7A2,ALDH2,ANKRD13A,APPL2,ARFGAP2,ARFGAP3,ARHGEF1,ARHGEF12,ARHGEF18,ASRGL1,ATIC,BIRC6,C17orf49,C1QBP,CAMSAP2,CAND1,CANX,CAVIN1,CBX3,CCDC47,CDK1,CDK4,CDK6,CEP55,CERT1,CNN2,CNPY2,CRAT,CSNK1A1,CTBP2,Cyb5r3,CYFIP1,DDB1,DDX5,DGKZ,DGLUCY,DNMT1,DOCK1,DOCK5,DPYSL2,DPYSL3,DYNC2H1,EEF1B2,EEF2,EEF2K,EIF2B4,ELOA,ELP1,ELP2,ELP3,EMC7,EMD,EML1,ERLIN2,EXOC2,EXOC4,EXTL3,FADD,FASN,FBXO7,FDFT1,FLII,FLNA,FLNB,FRMD4A,GABPA,GCDH,GMDS,GMPPB,GOT2,GRB14,GTSE1,HDGFL3,HM13,HMGCS1,HMMR,HMOX1,HNRNPUL1,HSPA4L,INPPL1,ITPR1,ITPR3,KPNA2,LARP7,LAYN,LETM1,LIMD1,LOXL3,LPP,MARK2,MEGF10,MGST2,MMUT,MTDH,MTSS1,NAA25,NAB2,NCLN,NDC80,NEK9,NFKB2,NPTXR,NQO1,NRBP1,NRDC,OSBPL8,OXCT1,PAK1,PAPSS1,PCK2,PDE4DIP,PEPD,PHLDA1,PIGU,PITRM1,PKN2,PLCB1,POLA1,POLD1,POLDIP3,PPOX,PRKCA,PRRX1,PTPN1,RAD51,RAF1,RANGAP1,RBPJ,RIOK3,RNASEH2B,RPA1,RPL5,RPL6,RPN1,RRM1,RRM2,SAMD4B,SDF4,SEC14L1,SEC61A1,SERPINE2,SLC9A3R1,SNX17,SORBS3,SPP1,SQLE,SRGAP3,STAT1,STAT3,STAT5B,STT3A,SUCLG2,TAGLN2,TDRKH,TLE3,TLN1,TMEM43,TNS3,TPD52,TRIM28,TRIM3,TRIOBP,TRIP10,TRIP12,TRIP13,TRMT2A,UBAP2L,UBE2C,UCKL1,USP24,USP47,USP9X,VCP,VCPIP1,VPS11,VPS13A,VPS36,VWA5A,WDR12,WFS1,WIPF1,XRN1,ZMPSTE24	193
Cancer,Organismal Injury and Abnormalities,Reproductive System Disease	Male genital neoplasm	0,0000345			AARSD1,ACADS,ACO1,ACSF2,ACSL3,ACSL4,AKR1B1,ALDH2,ALDH4A1,ALDH9A1,ANKRD13A,ARFGAP2,ARFGAP3,ARFGEF2,ARHGEF1,ATP2B4,ATP6V1A,BIRC6,CAND1,CARMIL1,CBX3,CC2D1B,CDC42EP4,CDK4,CDK6,CRAT,CTTNBP2,CUL3,DDB1,DGKZ,DNMT1,DOCK5,DPYSL3,DYNC2H1,EEF1B2,EEF2,EEF2K,ELP1,ELP2,EML1,EXOC2,EXOC4,FASN,FDFT1,FLNA,FLNB,GDA,GFPT1,GHDC,GOT2,GSDMD,HEBP2,HMOX1,HNRNPUL1,HSPA4L,INPPL1,IRGQ,ITPR1,ITPR3,KANK1,LARP7,LAYN,MKLN1,MMUT,MON2,MPST,NAB2,NAPA,NCLN,NEK9,NLE1,NMD3,NPTXR,NRDC,OGT,PAFAH1B1,PAK1,PAPSS1,PCK2,PDE4DIP,PDZRN3,PHLDA1,PIP4P2,PITRM1,PKN2,POLA1,POLD1,PPAT,PRKACB,PTPN1,RAD51,RAF1,RANGAP1,RAP2C,RETSAT,RIOK3,RPA1,RPA2,RRM1,RRM2,SAMD4B,SEC14L1,SEC24D,SERPINE2,SGTA,SH3GLB1,SLC1A4,SPP1,SQSTM1,STAT1,STAT3,STAT5B,TCF12,TKFC,TLE3,TNS3,TOM1L2,TRAPPC4,TRIM28,TRIOBP,TRIP12,UBAP2L,UBE2O,USP24,USP47,USP9X,VPS13A,VPS36,VWA5A,WDR12,WIPF1,XRN1	132
Cancer,Organismal Injury and Abnormalities	Grade 3-4 tumor	0,000035			ABCD4,ACP2,ACSF2,ADD1,AGA,AGFG1,AKR1B10,ALDH2,ALDH9A1,ANAPC7,AP3S2,APPL2,ARFGAP2,ARFGAP3,ARFGEF2,ARHGEF1,ARHGEF18,ATIC,ATP2A2,ATP2B4,ATP6AP1,ATP6V1A,ATXN10,BCAS1,BCAT2,BIRC6,CACYBP,CAMSAP2,CAND1,CANX,CARMIL1,CC2D1B,CCDC47,CDC42EP4,CDK4,CDK6,CEP55,CNN2,CNN3,CRAT,CTBP2,CTTNBP2,CUL3,CWF19L1,CYFIP1,DDB1,DDRGK1,DNMT1,DOCK1,DOCK5,DYNC2H1,EEF2K,EIF2B4,EIF2B5,ELP1,ELP3,ERLIN2,EXOC4,FADD,FASN,FBXO7,FLII,FLNA,FLNB,GABPA,GCLC,GCLM,GDA,GFPT1,GMDS,GMPPB,GPT2,GRB14,GSDMD,GTSE1,HDGFL3,HEBP2,HMGCS1,HMMR,HMOX1,IDH2,INPPL1,ITPR1,ITPR3,ITPRID2,KANK1,KATNB1,KPNA2,LPP,MARK2,MEGF10,MMUT,MON2,MPI,NAA25,NCLN,NFKB2,NIT2,NLE1,NLN,NRDC,NT5C3B,OGT,OXCT1,PAFAH1B1,PAK1,PAPSS1,PCBP2,PCK2,PDE4DIP,PDZRN3,PEPD,PFKM,PHLDA1,PIP4P2,PITRM1,PKN2,PLCB1,POLA1,POLD1,PPAT,PPP4R3A,PRRC2A,PTPN1,QSOX2,RAB32,RABGGTA,RAF1,RANGAP1,RBPJ,RPA1,RPL5,RPN1,RRM1,RRM2,SDF4,SEC14L1,SEC24A,SEC24D,SH3GLB1,SLC9A3R1,SNX1,SNX17,SOAT1,SORBS3,SPCS3,SPP1,SQSTM1,SRGAP3,STARD3NL,STAT1,STAT5B,STRN4,STT3A,TAX1BP1,TDRKH,TLN1,TM9SF4,TMEM43,TNS3,TOM1L2,TPD52,TRIM28,TRIOBP,TRIP10,TRIP12,UBE2C,UBR7,USP24,USP47,USP8,USP9X,VCP,VCPIP1,VPS13A,VWA5A,WDR62,WFS1,XRN1	179
Developmental Disorder,Hereditary Disorder,Neurological Disease,Organismal Injury and Abnormalities	Autosomal recessive mental retardation	0,0000364			EEF1B2,ELP2,ERLIN2,FASN,GATM,GMPPB,GPT2,IMPA1,LARP7,NARS1,STRN4,UBR7,WDR45B,WDR62	14
Cancer,Neurological Disease,Organismal Injury and Abnormalities	Grade 3-4 glioma	0,0000373			ABCD4,ACP2,ACSF2,ADD1,AGA,AGFG1,AKR1B10,ALDH2,ALDH9A1,ANAPC7,AP3S2,APPL2,ARFGAP2,ARFGAP3,ARFGEF2,ARHGEF1,ARHGEF18,ATIC,ATP2A2,ATP2B4,ATP6AP1,ATP6V1A,ATXN10,BCAS1,BCAT2,BIRC6,CACYBP,CAMSAP2,CAND1,CANX,CARMIL1,CC2D1B,CCDC47,CDC42EP4,CDK6,CEP55,CNN2,CNN3,CRAT,CTBP2,CTTNBP2,CUL3,CWF19L1,CYFIP1,DDB1,DDRGK1,DNMT1,DOCK1,DOCK5,DYNC2H1,EEF2K,EIF2B4,EIF2B5,ELP1,ELP3,ERLIN2,EXOC4,FADD,FASN,FBXO7,FLII,FLNA,FLNB,GABPA,GCLC,GCLM,GDA,GFPT1,GMDS,GMPPB,GPT2,GRB14,GSDMD,GTSE1,HDGFL3,HEBP2,HMGCS1,HMMR,HMOX1,IDH2,INPPL1,ITPR1,ITPR3,ITPRID2,KANK1,KATNB1,KPNA2,LPP,MARK2,MEGF10,MMUT,MON2,MPI,NAA25,NCLN,NFKB2,NIT2,NLE1,NLN,NRDC,NT5C3B,OGT,OXCT1,PAFAH1B1,PAK1,PAPSS1,PCBP2,PCK2,PDE4DIP,PDZRN3,PEPD,PFKM,PHLDA1,PIP4P2,PITRM1,PKN2,PLCB1,POLA1,POLD1,PPAT,PPP4R3A,PRRC2A,PTPN1,QSOX2,RAB32,RABGGTA,RAF1,RANGAP1,RBPJ,RPA1,RPL5,RPN1,RRM1,RRM2,SDF4,SEC14L1,SEC24A,SEC24D,SH3GLB1,SLC9A3R1,SNX1,SNX17,SOAT1,SORBS3,SPCS3,SPP1,SQSTM1,SRGAP3,STARD3NL,STAT1,STAT5B,STRN4,STT3A,TAX1BP1,TDRKH,TLN1,TM9SF4,TMEM43,TNS3,TOM1L2,TPD52,TRIM28,TRIOBP,TRIP10,TRIP12,UBE2C,UBR7,USP24,USP47,USP8,USP9X,VCP,VCPIP1,VPS13A,VWA5A,WDR62,WFS1,XRN1	178
Cancer,Organismal Injury and Abnormalities	Endometrioid carcinoma	0,0000381			ABCD4,ACADS,ACSL3,ACSL4,ADD1,AGL,AK3,AKR1B1,ALDH2,ALDH4A1,AP3S2,APPL2,ARFGEF2,ARHGEF1,ARHGEF12,ARHGEF18,ASRGL1,ATIC,ATP6AP1,BIRC6,CAMSAP2,CAND1,CBX3,CCDC47,CDIPT,CEP55,CTTNBP2,DDB1,DGKZ,DGLUCY,DNMT1,DOCK1,DOCK5,DPYSL2,DTYMK,DYNC2H1,EEF1B2,EEF2,ELP2,ELP3,EMC7,EMD,ERLIN2,EXOC2,EXOC4,FAF2,FBXO7,FDFT1,FLII,FLNA,FLNB,FRMD4A,FUT8,GATM,GCDH,GFPT1,GLRX3,GMDS,GMPPB,GPT2,HAUS7,HDGFL3,HMMR,HSPA4L,IDH2,IFT74,IKBIP,INPPL1,ITPR1,ITPR3,LARP7,LETM1,LRRC40,MEGF10,MKLN1,MMUT,MON2,MTDH,MTSS1,NAPA,NCLN,NFKB2,NPTXR,NQO1,NRDC,NUP54,OAT,OGT,OSBPL8,OXCT1,PARG,PCBP2,PCK2,PDE4DIP,PEPD,PFKM,PHLDA1,PHYH,PIGU,PITRM1,PKN2,PLCB1,POLA1,POLD1,POLD3,POLDIP3,PPAT,PPOX,PRKCA,PRPF19,PRRC2A,PTPN1,RAF1,RANGAP1,RER1,RETSAT,RIOK3,RPL5,RRM1,RRM2,SAMD4B,SEC14L1,SEC61A1,SNF8,SNX17,SOAT1,SORBS3,SPP1,STAT1,STAT3,STT3A,TAX1BP1,TCF12,TKFC,TLN1,TMEM43,TOM1L2,TRIM28,TRIM3,TRIOBP,TRIP12,TRMT2A,USP24,USP8,USP9X,VPS11,VPS13A,WIPF1,XRN1,ZMPSTE24	150
Cancer,Organismal Injury and Abnormalities,Reproductive System Disease	Uterine cancer	0,0000383			ABCD4,ACSL3,ACSL4,ADD1,ADI1,AK3,AKR7A2,ALDH2,ALDH4A1,AP3S2,APPL2,ARFGEF2,ARHGEF12,ARHGEF18,ASRGL1,ATIC,ATP6AP1,ATP6V1A,BCAS1,BIRC6,CAMSAP2,CAND1,CAVIN1,CBX3,CCZ1/CCZ1B,CDIPT,CDK4,CEP55,CTTNBP2,DDB1,DDRGK1,DGKZ,DGLUCY,DNMT1,DOCK1,DOCK5,DPYSL2,DTYMK,DYNC2H1,EEF1B2,EEF2,ELP3,EMC7,EMD,EXOC2,EXOC4,FAF2,FBXO7,FDFT1,FLII,FLNA,FLNB,FRMD4A,FUT8,GATM,GFPT1,GHDC,GLRX3,GPT2,HAUS7,HMMR,HMOX1,HSPA4L,IDH2,IFT74,IKBIP,INPPL1,ITPR1,ITPR3,KPNA2,LARP7,LAYN,LETM1,LIMD1,LPP,LRRC40,MEGF10,MKLN1,MMUT,MON2,MTDH,MTSS1,NAPA,NFKB2,NQO1,NRDC,NUP54,OAT,OGT,OSBPL8,OXCT1,PARG,PCBP2,PCK2,PDE4DIP,PDZRN3,PEPD,PFKM,PHYH,PIGU,PITRM1,PKN2,PLCB1,POLA1,POLD1,POLD3,POLDIP3,PPAT,PPOX,PRKCA,PRPF19,PRRC2A,PTPN1,RAD51,RAF1,RANGAP1,RER1,RETSAT,RIOK3,RPL5,RRM1,RRM2,SAMD4B,SDF4,SEC14L1,SEC61A1,SNF8,SNX1,SNX17,SOAT1,SORBS3,SPP1,STAT1,STAT3,STAT5B,STT3A,TAX1BP1,TCF12,TKFC,TLN1,TMEM43,TOM1L2,TRIM28,TRIM3,TRIOBP,TRIP12,TRIP13,TRMT2A,TST,UBE2C,USP24,USP8,USP9X,VCPIP1,VPS11,VPS13A,WIPF1,XRN1,ZMPSTE24	159
Neurological Disease,Organismal Injury and Abnormalities	Progressive encephalopathy	0,0000395			ALDH2,BCAS1,CANX,CDK1,CDK4,DPYSL2,DPYSL3,EEF2,EEF2K,ELP3,FADD,FASN,FBXO7,FDFT1,GATM,GLRX,HMGCL,HMGCS1,HMOX1,HMOX2,LOXL3,MEGF10,MMUT,NAXE,NFKB2,NFS1,NQO1,OGT,PAK1,PIP4P2,PMPCB,PRDX2,PRKACB,PRKCA,RAB6A,RAF1,RPL13,RPL3,SERPINE2,SLC1A4,SOAT1,SPP1,SQSTM1,STAT3,TRAPPC4,USP24,VCP	47
Cellular Movement	Initiation of cell movement of neurons	0.000042			ARFGEF2,FLNA,PAFAH1B1	3
Connective Tissue Disorders,Developmental Disorder,Neurological Disease,Organismal Injury and Abnormalities,Skeletal and Muscular Disorders	Microcephaly with neurodevelopmental impairment	0,0000443			NARS1,SLC1A4,TRAPPC4,VARS1	4
Cancer,Hematological Disease,Organismal Injury and Abnormalities	Advanced myelodysplastic syndrome	0,0000443			POLA1,POLD1,RRM1,RRM2	4
Cell Signaling	Small GTPase mediated signal transduction	0,0000452			ARHGEF1,ARHGEF12,ARHGEF18,CDC42EP4,CUL3,CYFIP1,DGKZ,DNMT1,HACD3,HMOX1,KANK1,KANK2,OGT,PAK1,RAF1,SRGAP3,TRIM28,TRIP10,USP8	19
Cancer,Organismal Injury and Abnormalities,Reproductive System Disease	Uterine tumor	0,0000465			ABCD4,ACSL3,ACSL4,ADD1,ADI1,AK3,AKR1B1,AKR7A2,ALDH2,ALDH4A1,AP3S2,APPL2,ARFGEF2,ARHGEF12,ARHGEF18,ASRGL1,ATIC,ATP6AP1,ATP6V1A,BCAS1,BIRC6,CAMSAP2,CAND1,CAVIN1,CBX3,CCZ1/CCZ1B,CDIPT,CDK4,CEP55,CTTNBP2,DDB1,DDRGK1,DGKZ,DGLUCY,DNMT1,DOCK1,DOCK5,DPYSL2,DTYMK,DYNC2H1,EEF1B2,EEF2,ELP3,EMC7,EMD,EXOC2,EXOC4,FAF2,FBXO7,FDFT1,FLII,FLNA,FLNB,FRMD4A,FUT8,GATM,GFPT1,GHDC,GLRX3,GPT2,HAUS7,HMMR,HMOX1,HSPA4L,IDH2,IFT74,IKBIP,INPPL1,ITPR1,ITPR3,KPNA2,LARP7,LAYN,LETM1,LIMD1,LPP,LRRC40,MEGF10,MKLN1,MMUT,MON2,MTDH,MTSS1,NAPA,NFKB2,NQO1,NRDC,NUP54,OAT,OGT,OSBPL8,OXCT1,PARG,PCBP2,PCK2,PDE4DIP,PDZRN3,PEPD,PFKM,PHYH,PIGU,PITRM1,PKN2,PLCB1,POLA1,POLD1,POLD3,POLDIP3,PPAT,PPOX,PRKCA,PRPF19,PRRC2A,PTPN1,RAD51,RAF1,RANGAP1,RER1,RETSAT,RIOK3,RPL27,RPL5,RRM1,RRM2,SAMD4B,SDF4,SEC14L1,SEC61A1,SNF8,SNX1,SNX17,SOAT1,SORBS3,SPP1,STAT1,STAT3,STAT5B,STT3A,TAX1BP1,TCF12,TKFC,TLN1,TMEM43,TOM1L2,TRIM28,TRIM3,TRIOBP,TRIP12,TRIP13,TRMT2A,TST,UBE2C,USP24,USP8,USP9X,VCP,VCPIP1,VPS11,VPS13A,WIPF1,XRN1,ZMPSTE24	162
Cancer,Organismal Injury and Abnormalities	Refractory advanced cancer	0,0000484			ATIC,CDK4,CDK6,IDH2,POLA1,POLD1,RAF1,RRM1,RRM2	9
Cancer,Hematological Disease,Immunological Disease,Organismal Injury and Abnormalities	De novo acute myeloid leukemia	0,0000485			DNMT1,POLA1,POLD1,PRKCA,RAF1,RPL3,RRM1,RRM2	8
Cancer,Gastrointestinal Disease,Organismal Injury and Abnormalities	Upper gastrointestinal carcinoma	0,0000506			AARSD1,ACO1,ACSF2,ACSL3,ACSL4,AGL,ALDH2,ANKRD13A,APPL2,ARFGAP2,ARFGEF2,ARHGEF1,ARHGEF18,ASRGL1,ATIC,ATP2A2,ATP6V1A,BIRC6,C1QBP,CAMSAP2,CANX,CDK4,CDK6,CEP55,CRAT,CSNK1A1,CTBP2,CTTNBP2,CYFIP1,DDB1,DDX5,DNAJC9,DNMT1,DOCK1,DOCK5,DPYSL3,DYNC2H1,EEF2,EEF2K,EIF2B4,ELOA,ELP1,EMC7,EMD,EML1,EXOC2,EXTL3,FASN,FDFT1,FLNA,FLNB,FRMD4A,GCDH,GDA,GLRX5,GMPPB,GRB14,HDGFL3,HM13,HMMR,HNRNPUL1,HSPA4L,IDH2,INPPL1,ITPR1,ITPR3,ITPRID2,KANK2,LARP7,LAYN,LIMD1,LOXL3,LPP,MARK2,MEGF10,MTDH,MTSS1,NAA25,NAB2,NCLN,NLE1,NQO1,NRBP1,NRDC,OAT,OXCT1,PAFAH1B1,PAK1,PAPSS1,PCK2,PDE4DIP,PEPD,PIGU,PKN2,PLCB1,POLA1,POLD1,POLD3,POLDIP3,PRKACB,PRKCA,PRRC2A,PRRX1,QSOX2,RAD51,RAF1,RBPJ,RNASEH2B,RPA1,RPL5,RRM1,RRM2,SDF4,SEC14L1,SERPINE2,SORBS3,SPP1,SQLE,SRGAP3,STAT1,STAT3,STAT5B,STT3A,SUCLG2,TAX1BP1,TDRKH,TLE3,TLN1,TNS3,TPD52,TRIM28,TRIM3,TRIOBP,TRIP10,TRIP12,TRIP13,TST,UBAP2L,UBE2C,UBR7,UCKL1,USP24,USP47,USP8,USP9X,VAT1,VCP,VCPIP1,VPS11,VPS13A,VPS36,WDR12,WFS1,WIPF1,XRN1,ZMPSTE24	156
Cancer,Hematological Disease,Immunological Disease,Organismal Injury and Abnormalities	Acute biphenotypic leukemia	0,0000533			DNMT1,POLA1,POLD1,PPAT,RPL3,RRM1,RRM2	7
Cancer,Neurological Disease,Organismal Injury and Abnormalities	Grade 3-4 glioma cancer	0,0000552			ABCD4,ACP2,ACSF2,ADD1,AGA,AGFG1,AKR1B10,ALDH2,ALDH9A1,ANAPC7,AP3S2,APPL2,ARFGAP2,ARFGAP3,ARFGEF2,ARHGEF1,ARHGEF18,ATIC,ATP2A2,ATP2B4,ATP6AP1,ATP6V1A,ATXN10,BCAS1,BCAT2,BIRC6,CACYBP,CAMSAP2,CAND1,CANX,CARMIL1,CC2D1B,CCDC47,CDC42EP4,CDK6,CEP55,CNN2,CNN3,CRAT,CTBP2,CTTNBP2,CUL3,CWF19L1,CYFIP1,DDB1,DDRGK1,DNMT1,DOCK1,DOCK5,DYNC2H1,EEF2K,EIF2B4,EIF2B5,ELP1,ELP3,ERLIN2,EXOC4,FADD,FASN,FBXO7,FLII,FLNA,FLNB,GABPA,GCLC,GCLM,GDA,GFPT1,GMDS,GMPPB,GPT2,GRB14,GSDMD,GTSE1,HDGFL3,HEBP2,HMGCS1,HMMR,HMOX1,IDH2,INPPL1,ITPR1,ITPR3,ITPRID2,KANK1,KATNB1,KPNA2,LPP,MARK2,MEGF10,MMUT,MON2,MPI,NAA25,NCLN,NFKB2,NIT2,NLE1,NLN,NRDC,NT5C3B,OGT,OXCT1,PAFAH1B1,PAK1,PAPSS1,PCBP2,PCK2,PDE4DIP,PDZRN3,PEPD,PFKM,PHLDA1,PIP4P2,PITRM1,PKN2,PLCB1,POLA1,POLD1,PPAT,PPP4R3A,PRRC2A,PTPN1,QSOX2,RAB32,RABGGTA,RAF1,RANGAP1,RBPJ,RPA1,RPL5,RPN1,RRM1,RRM2,SDF4,SEC14L1,SEC24A,SEC24D,SH3GLB1,SLC9A3R1,SNX1,SNX17,SOAT1,SORBS3,SPCS3,SQSTM1,SRGAP3,STARD3NL,STAT1,STAT5B,STRN4,STT3A,TAX1BP1,TDRKH,TLN1,TM9SF4,TMEM43,TNS3,TOM1L2,TPD52,TRIM28,TRIOBP,TRIP10,TRIP12,UBE2C,UBR7,USP24,USP47,USP8,USP9X,VCP,VCPIP1,VPS13A,VWA5A,WDR62,WFS1,XRN1	177
Cancer,Neurological Disease,Organismal Injury and Abnormalities	High grade astrocytoma	0,0000555			ABCD4,ACP2,ACSF2,ADD1,AGA,AGFG1,AKR1B10,ALDH2,ALDH9A1,ANAPC7,AP3S2,APPL2,ARFGAP2,ARFGAP3,ARFGEF2,ARHGEF1,ARHGEF18,ATIC,ATP2A2,ATP2B4,ATP6AP1,ATP6V1A,ATXN10,BCAS1,BCAT2,BIRC6,CACYBP,CAMSAP2,CAND1,CANX,CARMIL1,CC2D1B,CCDC47,CDC42EP4,CDK4,CDK6,CEP55,CNN2,CRAT,CSRP1,CTBP2,CTTNBP2,CUL3,CWF19L1,CYFIP1,DDB1,DDRGK1,DNMT1,DOCK1,DOCK5,DYNC2H1,EEF2K,EIF2B4,ELP1,ELP3,ERLIN2,EXOC4,FADD,FASN,FBXO7,FLII,FLNA,FLNB,GABPA,GCLC,GCLM,GDA,GFPT1,GMDS,GMPPB,GPT2,GRB14,GSDMD,HDGFL3,HEBP2,HMGCS1,HMOX1,IDH2,INPPL1,ITPR1,ITPR3,ITPRID2,KANK1,KATNB1,KPNA2,MARK2,MMUT,MON2,MPI,NAA25,NCLN,NFKB2,NIT2,NLE1,NLN,NRDC,NT5C3B,OGT,OXCT1,PAFAH1B1,PAPSS1,PCBP2,PCK2,PDE4DIP,PEPD,PFKM,PHLDA1,PIP4P2,PITRM1,PKN2,PLCB1,POLA1,POLD1,PPAT,PRRC2A,RAB32,RAF1,RANGAP1,RPA1,RPL5,RPN1,RRM1,RRM2,SDF4,SEC14L1,SEC24A,SEC24D,SH3GLB1,SLC9A3R1,SNX1,SOAT1,SORBS3,SPCS3,SPP1,SRGAP3,STARD3NL,STAT1,STAT3,STAT5B,STRN4,TAX1BP1,TDRKH,TLE3,TLN1,TM9SF4,TMEM43,TNS3,TOM1L2,TPD52,TRIM28,TRIOBP,TRIP10,TRIP12,UBE2C,UBR7,USP24,USP47,USP8,USP9X,VCP,VCPIP1,VWA5A,WDR62,WFS1,XRN1	165
Small Molecule Biochemistry	Metabolism of aldehyde	0,0000557			AKR7A2,ALDH2,ALDH4A1,ALDH9A1,GOT2	5

Categories	Diseases or Functions Annotation	p-value	Predicted Activation State	Activation z-score	Molecules	# Molecules
Cancer,Neurological Disease,Organismal Injury and Abnormalities	Grade 4 high grade glioma	0,0000574			ABCD4,ACP2,ACSF2,ADD1,AGA,AGFG1,AKR1B10,ALDH2,ALDH9A1,ANAPC7,AP3S2,APPL2,ARFGAP2,ARFGAP3,ARFGEF2,ARHGEF1,ARHGEF18,ATIC,ATP2B4,ATP6AP1,ATP6V1A,ATXN10,BCAS1,BCAT2,BIRC6,CACYBP,CAMSAP2,CAND1,CANX,CARMIL1,CC2D1B,CCDC47,CDC42EP4,CDK6,CEP55,CNN2,CRAT,CTBP2,CTTNBP2,CUL3,CWF19L1,CYFIP1,DDB1,DDRGK1,DNMT1,DOCK1,DOCK5,DYNC2H1,EEF2K,EIF2B4,ELP1,ELP3,ERLIN2,EXOC4,FADD,FASN,FBXO7,FLII,FLNA,FLNB,GABPA,GCLC,GCLM,GDA,GFPT1,GMDS,GMPPB,GPT2,GRB14,GSDMD,HDGFL3,HEBP2,HMGCS1,HMOX1,IDH2,INPPL1,ITPR1,ITPR3,ITPRID2,KANK1,KATNB1,KPNA2,MARK2,MMUT,MON2,MPI,NAA25,NCLN,NFKB2,NIT2,NLE1,NLN,NRDC,NT5C3B,OGT,OXCT1,PAFAH1B1,PAPSS1,PCBP2,PCK2,PDE4DIP,PEPD,PFKM,PHLDA1,PIP4P2,PITRM1,PKN2,PLCB1,POLA1,POLD1,PPAT,PRRC2A,RAB32,RAF1,RANGAP1,RPA1,RPL5,RPN1,RRM1,RRM2,SDF4,SEC14L1,SEC24A,SEC24D,SH3GLB1,SLC9A3R1,SNX1,SOAT1,SORBS3,SPCS3,SRGAP3,STARD3NL,STAT1,STAT5B,STRN4,TAX1BP1,TDRKH,TLN1,TM9SF4,TMEM43,TNS3,TOM1L2,TPD52,TRIM28,TRIOBP,TRIP10,TRIP12,UBE2C,UBR7,USP24,USP47,USP8,USP9X,VCP,VCPIP1,VWA5A,WDR62,WFS1,XRN1	159
Cancer,Neurological Disease,Organismal Injury and Abnormalities	Grade 4 malignant glioma	0,0000574			ABCD4,ACP2,ACSF2,ADD1,AGA,AGFG1,AKR1B10,ALDH2,ALDH9A1,ANAPC7,AP3S2,APPL2,ARFGAP2,ARFGAP3,ARFGEF2,ARHGEF1,ARHGEF18,ATIC,ATP2B4,ATP6AP1,ATP6V1A,ATXN10,BCAS1,BCAT2,BIRC6,CACYBP,CAMSAP2,CAND1,CANX,CARMIL1,CC2D1B,CCDC47,CDC42EP4,CDK6,CEP55,CNN2,CRAT,CTBP2,CTTNBP2,CUL3,CWF19L1,CYFIP1,DDB1,DDRGK1,DNMT1,DOCK1,DOCK5,DYNC2H1,EEF2K,EIF2B4,ELP1,ELP3,ERLIN2,EXOC4,FADD,FASN,FBXO7,FLII,FLNA,FLNB,GABPA,GCLC,GCLM,GDA,GFPT1,GMDS,GMPPB,GPT2,GRB14,GSDMD,HDGFL3,HEBP2,HMGCS1,HMOX1,IDH2,INPPL1,ITPR1,ITPR3,ITPRID2,KANK1,KATNB1,KPNA2,MARK2,MMUT,MON2,MPI,NAA25,NCLN,NFKB2,NIT2,NLE1,NLN,NRDC,NT5C3B,OGT,OXCT1,PAFAH1B1,PAPSS1,PCBP2,PCK2,PDE4DIP,PEPD,PFKM,PHLDA1,PIP4P2,PITRM1,PKN2,PLCB1,POLA1,POLD1,PPAT,PRRC2A,RAB32,RAF1,RANGAP1,RPA1,RPL5,RPN1,RRM1,RRM2,SDF4,SEC14L1,SEC24A,SEC24D,SH3GLB1,SLC9A3R1,SNX1,SOAT1,SORBS3,SPCS3,SRGAP3,STARD3NL,STAT1,STAT5B,STRN4,TAX1BP1,TDRKH,TLN1,TM9SF4,TMEM43,TNS3,TOM1L2,TPD52,TRIM28,TRIOBP,TRIP10,TRIP12,UBE2C,UBR7,USP24,USP47,USP8,USP9X,VCP,VCPIP1,VWA5A,WDR62,WFS1,XRN1	159
Cancer,Neurological Disease,Organismal Injury and Abnormalities	Grade 4 astrocytoma	0,0000574			ABCD4,ACP2,ACSF2,ADD1,AGA,AGFG1,AKR1B10,ALDH2,ALDH9A1,ANAPC7,AP3S2,APPL2,ARFGAP2,ARFGAP3,ARFGEF2,ARHGEF1,ARHGEF18,ATIC,ATP2B4,ATP6AP1,ATP6V1A,ATXN10,BCAS1,BCAT2,BIRC6,CACYBP,CAMSAP2,CAND1,CANX,CARMIL1,CC2D1B,CCDC47,CDC42EP4,CDK6,CEP55,CNN2,CRAT,CTBP2,CTTNBP2,CUL3,CWF19L1,CYFIP1,DDB1,DDRGK1,DNMT1,DOCK1,DOCK5,DYNC2H1,EEF2K,EIF2B4,ELP1,ELP3,ERLIN2,EXOC4,FADD,FASN,FBXO7,FLII,FLNA,FLNB,GABPA,GCLC,GCLM,GDA,GFPT1,GMDS,GMPPB,GPT2,GRB14,GSDMD,HDGFL3,HEBP2,HMGCS1,HMOX1,IDH2,INPPL1,ITPR1,ITPR3,ITPRID2,KANK1,KATNB1,KPNA2,MARK2,MMUT,MON2,MPI,NAA25,NCLN,NFKB2,NIT2,NLE1,NLN,NRDC,NT5C3B,OGT,OXCT1,PAFAH1B1,PAPSS1,PCBP2,PCK2,PDE4DIP,PEPD,PFKM,PHLDA1,PIP4P2,PITRM1,PKN2,PLCB1,POLA1,POLD1,PPAT,PRRC2A,RAB32,RAF1,RANGAP1,RPA1,RPL5,RPN1,RRM1,RRM2,SDF4,SEC14L1,SEC24A,SEC24D,SH3GLB1,SLC9A3R1,SNX1,SOAT1,SORBS3,SPCS3,SRGAP3,STARD3NL,STAT1,STAT5B,STRN4,TAX1BP1,TDRKH,TLN1,TM9SF4,TMEM43,TNS3,TOM1L2,TPD52,TRIM28,TRIOBP,TRIP10,TRIP12,UBE2C,UBR7,USP24,USP47,USP8,USP9X,VCP,VCPIP1,VWA5A,WDR62,WFS1,XRN1	159
Cancer,Hematological Disease,Immunological Disease,Organismal Injury and Abnormalities	Primary acute myeloid leukemia	0,0000578			IDH2,POLA1,POLD1,RAF1,RRM1,RRM2	6
Cancer,Organismal Injury and Abnormalities,Reproductive System Disease	Female genital carcinoma	0,0000579			ABCD4,ACADS,ACSL3,ACSL4,ADD1,AGL,AK3,AKR1B1,ALDH2,ALDH4A1,AP3S2,APPL2,ARFGEF2,ARHGEF1,ARHGEF12,ARHGEF18,ASRGL1,ATIC,ATP6AP1,ATP6V1A,BCAS1,BIRC6,CAMSAP2,CAND1,CAVIN1,CBX3,CCDC47,CDIPT,CDK4,CEP55,CTTNBP2,DDB1,DGKZ,DGLUCY,DNMT1,DOCK1,DOCK5,DPYSL2,DTYMK,DYNC2H1,EEF1B2,EEF2,ELOA,ELP2,ELP3,EMC7,EMD,ERLIN2,EXOC2,EXOC4,FAF2,FBXO7,FDFT1,FLII,FLNA,FLNB,FRMD4A,FUT8,GATM,GCDH,GFPT1,GLRX3,GMDS,GMPPB,GOT2,GPT2,HAUS7,HDGFL3,HMMR,HSPA4L,IDH2,IFT74,IKBIP,INPPL1,ITPR1,ITPR3,KPNA2,LARP7,LETM1,LPP,LRRC40,MEGF10,MKLN1,MMUT,MON2,MTDH,MTSS1,NAA25,NAPA,NCLN,NFKB2,NPTXR,NQO1,NRDC,NUP54,OAT,OGT,OSBPL8,OXCT1,PARG,PCBP2,PCK2,PDE4DIP,PDZRN3,PEPD,PFKM,PHLDA1,PHYH,PIGU,PITRM1,PKN2,PLCB1,POLA1,POLD1,POLD3,POLDIP3,PPAT,PPOX,PRKCA,PRPF19,PRRC2A,PTPN1,RAD51,RAF1,RANGAP1,RER1,RETSAT,RIOK3,RNASEH2B,RPL5,RRM1,RRM2,SAMD4B,SDF4,SEC14L1,SEC61A1,SNF8,SNX17,SOAT1,SORBS3,SPP1,STAT1,STAT3,STT3A,SUCLG2,TAX1BP1,TCF12,TKFC,TLN1,TMEM43,TNS3,TOM1L2,TRIM28,TRIM3,TRIOBP,TRIP12,TRIP13,TRMT2A,UBE2C,USP24,USP8,USP9X,VPS11,VPS13A,WFS1,WIPF1,XRN1,ZMPSTE24	168
Cancer,Organismal Injury and Abnormalities,Reproductive System Disease	Stage III-IV breast cancer	0,0000633			CBX3,CDK4,CDK6,DPYSL2,FDFT1,HMOX1,RRM1,RRM2,SLC9A3R1,SQLE	10
Cancer,Hematological Disease,Immunological Disease,Organismal Injury and Abnormalities	Intermediate-risk acute myeloid leukemia	0,0000661			DNMT1,IDH2,POLA1,POLD1,PRKCA,RRM1,RRM2	7
Cancer,Hematological Disease,Immunological Disease,Organismal Injury and Abnormalities	Acute monocytic leukemia (M5)	0,0000662			IDH2,POLA1,POLD1,PPAT,RRM1,RRM2	6
Cellular Compromise,Inflammatory Response	Degranulation of neutrophils	0,0000663			AGA,AGL,ARMC8,CAND1,CNN2,CYFIP1,DNAJC5,EEF2,FAF2,GHDC,GSDMD,HEBP2,LAMTOR2,LAMTOR3,LTA4H,NIT2,RAB6A,RAP2C,VAMP7,VAT1,VCP	21
Cancer,Hematological Disease,Organismal Injury and Abnormalities	Low risk myelodysplastic syndrome	0,0000675			DNMT1,POLA1,POLD1,RRM1,RRM2	5
Cancer,Organismal Injury and Abnormalities,Reproductive System Disease	Malignant neoplasm of male genital organ	0,0000729			AARSD1,ACADS,ACO1,ACSF2,ACSL3,ACSL4,AKR1B1,ALDH2,ALDH4A1,ALDH9A1,ANKRD13A,ARFGAP2,ARFGAP3,ARFGEF2,ARHGEF1,ATP2B4,ATP6V1A,BIRC6,CAND1,CARMIL1,CBX3,CC2D1B,CDC42EP4,CDK4,CDK6,CRAT,CTTNBP2,CUL3,DDB1,DGKZ,DNMT1,DOCK5,DYNC2H1,EEF1B2,EEF2,EEF2K,ELP1,ELP2,EML1,EXOC2,EXOC4,FASN,FDFT1,FLNA,FLNB,GDA,GFPT1,GHDC,GOT2,GSDMD,HEBP2,HMOX1,HNRNPUL1,HSPA4L,INPPL1,IRGQ,ITPR1,ITPR3,KANK1,LARP7,LAYN,MKLN1,MMUT,MON2,MPST,NAB2,NAPA,NCLN,NEK9,NLE1,NMD3,NPTXR,NRDC,OGT,PAFAH1B1,PAK1,PAPSS1,PCK2,PDE4DIP,PDZRN3,PHLDA1,PIP4P2,PITRM1,PKN2,POLA1,POLD1,PPAT,PRKACB,PTPN1,RAD51,RAF1,RANGAP1,RAP2C,RETSAT,RIOK3,RPA1,RPA2,RRM1,RRM2,SAMD4B,SEC14L1,SEC24D,SERPINE2,SGTA,SH3GLB1,SLC1A4,SPP1,SQSTM1,STAT1,STAT3,STAT5B,TLE3,TNS3,TOM1L2,TRAPPC4,TRIM28,TRIOBP,TRIP12,UBAP2L,UBE2O,USP24,USP47,USP9X,VPS13A,VPS36,VWA5A,WDR12,WIPF1,XRN1	129
Cancer,Organismal Injury and Abnormalities	Refractory malignant tumor	0,0000762			ATIC,CDK4,CDK6,DNMT1,HMOX1,IDH2,POLA1,POLD1,PPAT,RAF1,RPL3,RPL6,RRM1,RRM2,SPP1	15
Cancer,Organismal Injury and Abnormalities	Stage III cancer	0,0000773			ATIC,CBX3,DNMT1,DPYSL2,FDFT1,HMOX1,POLA1,POLD1,RAF1,RRM1,RRM2,SLC9A3R1,SPP1,SQLE	14
Cancer,Organismal Injury and Abnormalities,Reproductive System Disease	Breast cancer	0,0000787			ACP2,AKR1B1,AKR7A2,ARFGAP3,ARHGEF1,ASRGL1,BIRC6,C17orf49,C1QBP,CANX,CAVIN1,CBX3,CDK1,CDK4,CDK6,CERT1,CNN2,CNPY2,CRAT,CSNK1A1,CTBP2,Cyb5r3,DDB1,DDX5,DGKZ,DGLUCY,DOCK5,DPYSL2,DYNC2H1,ELOA,ELP1,ELP3,ERLIN2,EXOC4,FADD,FASN,FDFT1,FLNA,FLNB,GABPA,GTSE1,HMGCS1,HMMR,HMOX1,HNRNPUL1,INPPL1,ITPR1,KPNA2,LETM1,LOXL3,MARK2,MGST2,MTDH,NAB2,NDC80,NEK9,NQO1,OXCT1,PAK1,PCK2,PHLDA1,PITRM1,PKN2,PLCB1,POLD1,PPOX,PRKCA,PRRX1,PTPN1,RAD51,RAF1,RANGAP1,RIOK3,RPL5,RPL6,RRM1,RRM2,SAMD4B,SEC14L1,SEC61A1,SLC9A3R1,SPP1,SQLE,STAT1,STAT3,STAT5B,TAGLN2,TLE3,TLN1,TMEM43,TPD52,TRIM28,TRIM3,TRIP10,TRIP12,TRIP13,TRMT2A,UBAP2L,UBE2C,USP24,USP47,USP9X,VCPIP1,VPS13A,VWA5A,ZMPSTE24	106
Cancer,Organismal Injury and Abnormalities	Primary solid tumor	0,0000807			ATIC,CDK4,CDK6,CSNK1A1,CTBP2,DGKZ,DNMT1,DOCK1,FASN,FLNA,GOT2,KANK2,KPNA2,MTDH,PAFAH1B1,POLA1,POLD1,RAF1,RRM1,RRM2,SH3GLB1,SLC9A3R1,SPP1,STAT3	24
Cancer,Hematological Disease,Immunological Disease,Organismal Injury and Abnormalities	T(15;17) mutation negative acute myeloid leukemia	0,0000811			DNMT1,POLA1,POLD1,RRM1,RRM2	5
Cancer,Hematological Disease,Immunological Disease,Organismal Injury and Abnormalities	Type M2 acute myeloid leukemia	0,0000811			FLNA,IDH2,POLA1,POLD1,PPAT,RRM1,RRM2	7
Cancer,Hematological Disease,Immunological Disease,Organismal Injury and Abnormalities	Refractory ALK positive large B-cell lymphoma	0,0000824			POLA1,POLD1,RRM1,RRM2	4
Cancer,Hematological Disease,Immunological Disease,Organismal Injury and Abnormalities	High grade refractory B-cell non-Hodgkin lymphoma	0,0000824			POLA1,POLD1,RRM1,RRM2	4
Cancer,Hematological Disease,Immunological Disease,Organismal Injury and Abnormalities	High grade relapsed B-cell non-Hodgkin lymphoma	0,0000824			POLA1,POLD1,RRM1,RRM2	4
Cancer,Hematological Disease,Immunological Disease,Organismal Injury and Abnormalities,Tumor Morphology	Progressive diffuse large B-cell lymphoma	0,0000824			POLA1,POLD1,RRM1,RRM2	4
Cancer,Hematological Disease,Immunological Disease,Organismal Injury and Abnormalities	Inv(3) positive acute myeloid leukemia	0,0000824			POLA1,POLD1,RRM1,RRM2	4
Cancer,Hematological Disease,Organismal Injury and Abnormalities,Tumor Morphology	Progressive ALK fusion positive malignant hematologic neoplasm	0,0000824			POLA1,POLD1,RRM1,RRM2	4
Cancer,Hematological Disease,Immunological Disease,Organismal Injury and Abnormalities	FLT3 ITD mutation positive t(15;17) negative acute myeloid leukemia	0,0000824			POLA1,POLD1,RRM1,RRM2	4
Cancer,Hematological Disease,Immunological Disease,Organismal Injury and Abnormalities	Relapsed ALK positive large B-cell lymphoma	0,0000824			POLA1,POLD1,RRM1,RRM2	4
Cancer,Hematological Disease,Immunological Disease,Organismal Injury and Abnormalities	Refractory relapsed acute myeloid leukemia	0,000083			POLA1,RRM1,RRM2	3
Cancer,Hematological Disease,Immunological Disease,Organismal Injury and Abnormalities	Refractory CD20 positive T-cell/histiocyte rich diffuse large B-cell lymphoma	0,000083			POLA1,RRM1,RRM2	3
Cancer,Hematological Disease,Immunological Disease,Organismal Injury and Abnormalities	Relapsed CD20 positive T-cell/histiocyte rich diffuse large B-cell lymphoma	0,000083			POLA1,RRM1,RRM2	3
Cancer,Hematological Disease,Immunological Disease,Organismal Injury and Abnormalities	Relapsed c-MYC positive diffuse large B-cell lymphoma	0,000083			POLA1,RRM1,RRM2	3
Cancer,Hematological Disease,Immunological Disease,Organismal Injury and Abnormalities	Refractory acute myeloid leukemia with t(8;21)	0,000083			POLA1,RRM1,RRM2	3
Cancer,Hematological Disease,Immunological Disease,Organismal Injury and Abnormalities	Refractory MYC positive B-cell lymphoma	0,000083			POLA1,RRM1,RRM2	3
Small Molecule Biochemistry	Metabolism of dicarboxylic acid	0,0000861			GOT2,GPT2,NIT2,PCK2,PHYH,SUCLG2	6
Cancer,Organismal Injury and Abnormalities,Reproductive System Disease	Endometrial cancer	0,0000862			ABCD4,ACSL3,ACSL4,ADD1,ADI1,AK3,ALDH2,ALDH4A1,AP3S2,APPL2,ARFGEF2,ARHGEF12,ARHGEF18,ASRGL1,ATIC,ATP6AP1,BIRC6,CAMSAP2,CAND1,CAVIN1,CBX3,CCZ1/CCZ1B,CDIPT,CDK4,CEP55,CTTNBP2,DDB1,DDRGK1,DGKZ,DGLUCY,DNMT1,DOCK1,DOCK5,DPYSL2,DTYMK,DYNC2H1,EEF1B2,EEF2,ELP3,EMC7,EMD,EXOC2,EXOC4,FAF2,FBXO7,FDFT1,FLII,FLNA,FLNB,FRMD4A,FUT8,GATM,GFPT1,GHDC,GLRX3,GPT2,HAUS7,HMMR,HSPA4L,IDH2,IFT74,IKBIP,INPPL1,ITPR1,ITPR3,KPNA2,LARP7,LAYN,LETM1,LIMD1,LPP,LRRC40,MEGF10,MKLN1,MMUT,MON2,MTDH,MTSS1,NAPA,NFKB2,NQO1,NRDC,NUP54,OAT,OGT,OSBPL8,OXCT1,PARG,PCBP2,PCK2,PDE4DIP,PDZRN3,PEPD,PFKM,PHYH,PIGU,PITRM1,PKN2,PLCB1,POLA1,POLD1,POLD3,POLDIP3,PPAT,PPOX,PRKCA,PRPF19,PRRC2A,PTPN1,RAD51,RAF1,RANGAP1,RER1,RETSAT,RIOK3,RPL5,SAMD4B,SEC14L1,SEC61A1,SNF8,SNX1,SNX17,SOAT1,SORBS3,STAT1,STAT3,STT3A,TAX1BP1,TCF12,TKFC,TLN1,TMEM43,TOM1L2,TRIM28,TRIM3,TRIOBP,TRIP12,TRIP13,TRMT2A,UBE2C,USP24,USP8,USP9X,VCPIP1,VPS11,VPS13A,WIPF1,XRN1,ZMPSTE24	149
Cancer,Hereditary Disorder,Organismal Injury and Abnormalities	Hereditary cancer-predisposing syndrome	0,0000869			ACACA,AIP,ALDH2,BIRC6,CARMIL1,CDK4,FLNA,HDGFL3,HMMR,HMOX1,ITPR1,LPP,NFKB2,NQO1,POLA1,POLD1,PRKCA,RAD51,RAF1,RPL27,RPL5,RPN1,RRM1,RRM2,STAT3,TRIM28,VCP	27
Cancer,Organismal Injury and Abnormalities	BRAF mutation positive malignant solid tumor	0,0000885			ATIC,CDK4,CDK6,POLA1,POLD1,RAF1,RRM1,RRM2	8
Cancer,Hematological Disease,Organismal Injury and Abnormalities	Malignant myeloid neoplasm	0,0000902			ACSL3,ACSL4,ALDH4A1,ALDH6A1,ATP2B4,ATP6AP1,ATP6V1A,CDK1,CRAT,CSNK1A1,CTBP2,DGLUCY,DNAJC5,DNMT1,DYNC2H1,EEF2,EML1,ERLIN2,EXTL3,FBXO7,FLII,FLNA,GMDS,IDH2,IFT74,ITPR1,ITPR3,KANK2,LAMTOR2,LOXL3,LPP,NQO1,NUP54,PDE4DIP,PIGU,POLA1,POLD1,PPAT,PRKACB,PRKCA,PTPN1,RAD51,RAF1,RAI14,RPL13,RPL3,RPL5,RPL6,RRM1,RRM2,SQSTM1,STAT3,STAT5B,TCF12,TLE3,TLN1,TRIP12,TRIP13,UBE2C,UBE2I,USP9X,VAMP7,WDR62,XRN1	64
Cancer,Organismal Injury and Abnormalities,Reproductive System Disease	Prostatic tumor	0,0001			AARSD1,ACADS,ACO1,ACSF2,ACSL3,ACSL4,AKR1B1,ALDH2,ALDH4A1,ALDH9A1,ANKRD13A,ARFGAP2,ARFGAP3,ARFGEF2,ARHGEF1,ATP2B4,ATP6V1A,BIRC6,CAND1,CARMIL1,CBX3,CC2D1B,CDC42EP4,CDK4,CDK6,CRAT,CTTNBP2,CUL3,DDB1,DGKZ,DNMT1,DOCK5,DPYSL3,DYNC2H1,EEF1B2,EEF2,EEF2K,ELP1,ELP2,EML1,EXOC2,EXOC4,FASN,FDFT1,FLNA,FLNB,GDA,GFPT1,GHDC,GOT2,GSDMD,HEBP2,HMOX1,HNRNPUL1,HSPA4L,INPPL1,IRGQ,ITPR1,ITPR3,KANK1,LARP7,LAYN,MKLN1,MMUT,MON2,MPST,NAB2,NAPA,NCLN,NEK9,NLE1,NMD3,NPTXR,NRDC,OGT,PAFAH1B1,PAK1,PAPSS1,PCK2,PDE4DIP,PHLDA1,PIP4P2,PITRM1,PKN2,POLA1,POLD1,PPAT,PRKACB,PTPN1,RAD51,RAF1,RANGAP1,RETSAT,RIOK3,RPA1,RPA2,RRM1,RRM2,SAMD4B,SEC14L1,SEC24D,SERPINE2,SGTA,SH3GLB1,SLC1A4,SPP1,SQSTM1,STAT1,STAT3,STAT5B,TNS3,TOM1L2,TRAPPC4,TRIM28,TRIOBP,TRIP12,UBAP2L,UBE2O,USP24,USP47,USP9X,VPS13A,VPS36,VWA5A,WDR12,WIPF1,XRN1	127
Cellular Compromise,Cellular Function and Maintenance	Endoplasmic reticulum stress response	0,000103			ATP2A2,CCDC47,CERT1,DDRGK1,EIF2B5,HMOX1,HSPA4L,PAFAH1B1,PTPN1,RAB6A,SQSTM1,VCP,WFS1	13
Neurological Disease,Organismal Injury and Abnormalities,Psychological Disorders	Disorder of basal ganglia	0,000104			ALDH2,ALDH6A1,ARHGEF1,ATP2A2,ATP6V1A,BCAS1,CRAT,DPYSL3,EML1,FBXO7,FDFT1,FRMD4A,HMOX1,HMOX2,IMPA1,INPP1,ITPR1,LOXL3,LPP,MTDH,NAPA,NDUFAF7,OSBPL8,PAK1,PDE4DIP,PFKM,PIP4P2,PLCB1,PRDX2,RAB6A,RNASEH2B,RPL13,RPL3,SEC24A,SGTA,SLC1A4,SPP1,STAT1,STRN4,TPD52,USP24,VPS13A	42
Amino Acid Metabolism,Small Molecule Biochemistry	Metabolism of L-amino acid	0,000107			ALDH4A1,ASRGL1,DGLUCY,GCDH,GCLC,GCLM,GOT2,GPT2,GSTZ1	9

Categories	Diseases or Functions Annotation	p-value	Predicted Activation State	Activation z-score	Molecules	# Molecules
Cancer,Organismal Injury and Abnormalities,Reproductive System Disease	Breast or ovarian carcinoma	0,000108			ACADS,ACP2,AGL,AK3,AKR1B1,ARFGAP3,ARHGEF1,ARHGEF12,ARHGEF18,ASRGL1,BIRC6,C17orf49,C1QBP,CAND1,CANX,CAVIN1,CCDC47,CDK1,CDK4,CDK6,CNN2,CNPY2,CRAT,CSNK1A1,CTBP2,DDB1,DGKZ,DGLUCY,DNMT1,DOCK5,DPYSL2,DYNC2H1,EEF1B2,ELOA,ELP1,ELP2,ELP3,EMD,ERLIN2,EXOC4,FADD,FASN,FBXO7,FDFT1,FLII,FLNA,FLNB,GABPA,GCDH,GMDS,GMPPB,GOT2,GTSE1,HDGFL3,HMGCS1,INPPL1,ITPR1,LETM1,LOXL3,MARK2,MMUT,MTDH,NAA25,NAB2,NCLN,NDC80,NEK9,NPTXR,OSBPL8,OXCT1,PAK1,PCK2,PDE4DIP,PEPD,PHLDA1,PITRM1,PKN2,PLCB1,POLDIP3,PPOX,PRKCA,PRRX1,PTPN1,RAD51,RAF1,RANGAP1,RIOK3,RNASEH2B,RPL5,RRM1,RRM2,SAMD4B,SEC14L1,SEC61A1,SNX17,SPP1,STAT1,STAT3,STAT5B,SUCLG2,TLE3,TMEM43,TNS3,TPD52,TRIM28,TRIM3,TRIP10,TRIP12,TRMT2A,UBE2C,USP24,USP47,USP9X,VCPIP1,VPS13A,WFS1,WIPF1,ZMPSTE24	118
Cancer,Organismal Injury and Abnormalities	Stable disease stage cancer	0,000109			ATIC,POLA1,RRM1,RRM2	4
Connective Tissue Disorders,Developmental Disorder,Metabolic Disease,Organismal Injury and Abnormalities,Skeletal and Muscular Disorders	Paget disease of bone	0,000109			FDFT1,NFKB2,SQSTM1,VCP	4
Cancer,Hematological Disease,Immunological Disease,Organismal Injury and Abnormalities	Acute myeloid leukemia associated with myelodysplastic syndrome	0,00011			DNMT1,IDH2,POLA1,POLD1,RRM1,RRM2	6
Cell Cycle	Arrest in G2 phase	0,000112			CDK1,DDB1,DNMT1,EEF2K,FADD,FASN,FLNA,MTDH,OGT,PAK1,RAD51,RAF1,RPA1,RPL7A	14
Cancer,Hematological Disease,Immunological Disease,Organismal Injury and Abnormalities	Chronic myelomonocytic leukemia	0,000114			DNMT1,IDH2,POLA1,POLD1,PPAT,RAD51,RRM1,RRM2,STAT5B	9
Developmental Disorder,Neurological Disease,Organismal Injury and Abnormalities	Neuronal migration disorder	0,00012			ARFGEF2,EML1,FLNA,GMPPB,KATNB1,PAFAH1B1,STRN4	7
Cancer,Hematological Disease,Immunological Disease,Organismal Injury and Abnormalities	Type M3 acute myeloid leukemia	0,00012			DNMT1,POLA1,POLD1,PPAT,RRM1,RRM2,STAT5B	7
Cancer,Gastrointestinal Disease,Organismal Injury and Abnormalities	Upper gastrointestinal tract tumor	0,000122			AARSD1,ACO1,ACSF2,ACSL3,ACSL4,AGL,AKR1B1,ALDH2,ANKRD13A,APPL2,ARFGAP2,ARFGEF2,ARHGEF1,ARHGEF18,ASRGL1,ATIC,ATP2A2,ATP6V1A,BIRC6,C1QBP,CAMSAP2,CANX,CDK4,CDK6,CEP55,CRAT,CSNK1A1,CTBP2,CTTNBP2,CYFIP1,DDB1,DDX5,DNAJC9,DNMT1,DOCK1,DOCK5,DPYSL3,DYNC2H1,EEF2,EEF2K,EIF2B4,ELOA,ELP1,EMC7,EMD,EML1,EXOC2,EXTL3,FASN,FDFT1,FLNA,FLNB,FRMD4A,GCDH,GDA,GLRX5,GMPPB,GRB14,HDGFL3,HM13,HMMR,HNRNPUL1,HSPA4L,IDH2,INPPL1,ITPR1,ITPR3,ITPRID2,KANK2,LARP7,LAYN,LETM1,LIMD1,LOXL3,LPP,MARK2,MEGF10,MTDH,MTSS1,NAA25,NAB2,NCLN,NLE1,NQO1,NRBP1,NRDC,OAT,OXCT1,PAFAH1B1,PAK1,PAPSS1,PCK2,PDE4DIP,PEPD,PIGU,PKN2,PLCB1,POLA1,POLD1,POLD3,POLDIP3,PRKACB,PRKCA,PRRC2A,PRRX1,QSOX2,RAD51,RAF1,RBPJ,RNASEH2B,RPA1,RPL5,RRM1,RRM2,SDF4,SEC14L1,SERPINE2,SORBS3,SPP1,SQLE,SRGAP3,STAT1,STAT3,STAT5B,STT3A,SUCLG2,TAX1BP1,TDRKH,TLE3,TLN1,TNS3,TPD52,TRIM28,TRIM3,TRIOBP,TRIP10,TRIP12,TRIP13,TST,UBAP2L,UBE2C,UBR7,UCKL1,USP24,USP47,USP8,USP9X,VAT1,VCP,VCPIP1,VPS11,VPS13A,VPS36,WDR12,WFS1,WIPF1,XRN1,ZMPSTE24	158
Cancer,Organismal Injury and Abnormalities,Reproductive System Disease	Prostate cancer	0,000124			AARSD1,ACADS,ACO1,ACSF2,ACSL3,ACSL4,AKR1B1,ALDH2,ALDH4A1,ALDH9A1,ANKRD13A,ARFGAP2,ARFGAP3,ARFGEF2,ARHGEF1,ATP2B4,ATP6V1A,BIRC6,CAND1,CARMIL1,CBX3,CC2D1B,CDC42EP4,CDK4,CDK6,CRAT,CTTNBP2,CUL3,DDB1,DGKZ,DNMT1,DOCK5,DYNC2H1,EEF1B2,EEF2,EEF2K,ELP1,ELP2,EML1,EXOC2,EXOC4,FASN,FDFT1,FLNA,FLNB,GDA,GFPT1,GHDC,GOT2,GSDMD,HEBP2,HMOX1,HNRNPUL1,HSPA4L,INPPL1,IRGQ,ITPR1,ITPR3,KANK1,LARP7,LAYN,MKLN1,MMUT,MON2,MPST,NAB2,NAPA,NCLN,NEK9,NLE1,NMD3,NPTXR,NRDC,OGT,PAFAH1B1,PAK1,PAPSS1,PCK2,PDE4DIP,PHLDA1,PIP4P2,PITRM1,PKN2,POLA1,POLD1,PPAT,PRKACB,PTPN1,RAD51,RAF1,RANGAP1,RETSAT,RIOK3,RPA1,RPA2,RRM1,RRM2,SAMD4B,SEC14L1,SEC24D,SERPINE2,SGTA,SH3GLB1,SLC1A4,SPP1,SQSTM1,STAT1,STAT3,STAT5B,TNS3,TOM1L2,TRAPPC4,TRIM28,TRIOBP,TRIP12,UBAP2L,UBE2O,USP24,USP47,USP9X,VPS13A,VPS36,VWA5A,WDR12,WIPF1,XRN1	126
Developmental Disorder	Autism spectrum disorder or intellectual disability	0,000134			ACSL4,AGA,AGFG1,ALDH4A1,ARFGEF2,ATP6V1A,BIRC6,CERT1,CUL3,CWF19L1,CYFIP1,DYNC2H1,EEF1B2,ELP2,ERLIN2,FASN,FLNA,GATM,GMPPB,GPT2,IMPA1,ITPR1,LARP7,NARS1,OGT,PAFAH1B1,PAK1,POLA1,POLD1,PPP2R5D,RPN2,SLC1A4,STRN4,TRIP12,UBR7,USP9X,VCP,WDR45B,WDR62	39
Cancer,Organismal Injury and Abnormalities	Growth of benign tumor	0,000135			DNMT1,PRKCA,RAF1,SLC9A3R2,STAT3	5
Developmental Disorder,Neurological Disease	Syndromic neurodevelopmental disorder	0,000137			CUL3,DYNC2H1,ELP1,EXTL3,GOT2,NARS1,NCLN,PIGU,PLCB1,RAF1,RPL6,SLC1A4,SNF8,TRAPPC4,UBR7,VARS1,WDR45B,WDR62	18
Cancer,Gastrointestinal Disease,Organismal Injury and Abnormalities	Non-colon gastrointestinal cancer	0,000138			AARSD1,ACO1,ACSF2,ACSL3,ACSL4,AGL,ALDH2,ANKRD13A,APPL2,ARFGAP2,ARFGEF2,ARHGEF1,ARHGEF18,ARMC8,ASRGL1,ATIC,ATP2A2,ATP6V1A,BIRC6,C1QBP,CAMSAP2,CANX,CAVIN1,CDK4,CDK6,CEP55,CRAT,CSNK1A1,CTBP2,CTTNBP2,CYFIP1,DDB1,DDX5,DGKZ,DNAJC9,DNMT1,DOCK1,DOCK5,DPYSL3,DYNC2H1,EEF2,EEF2K,EIF2B4,ELOA,ELP1,EMC7,EMD,EML1,EXOC2,EXTL3,FASN,FDFT1,FLNA,FLNB,FRMD4A,GCDH,GDA,GLRX5,GMPPB,GRB14,HDGFL3,HM13,HMMR,HNRNPUL1,HSPA4L,IDH2,INPPL1,ITPR1,ITPR3,ITPRID2,KANK2,LARP7,LAYN,LETM1,LIMD1,LOXL3,LPP,MARK2,MEGF10,MTDH,MTSS1,NAA25,NAB2,NCLN,NEK9,NLE1,NQO1,NRBP1,NRDC,OAT,OXCT1,PAFAH1B1,PAK1,PAPSS1,PCBP2,PCK2,PDE4DIP,PEPD,PIGU,PKN2,PLCB1,POLA1,POLD1,POLD3,POLDIP3,PRKACB,PRKCA,PRRC2A,PRRX1,QSOX2,RAD51,RAF1,RBPJ,RNASEH2B,RPA1,RPL5,RRM1,RRM2,SDF4,SEC14L1,SERPINE2,SORBS3,SPP1,SQLE,SRGAP3,STAT1,STAT3,STAT5B,STT3A,SUCLG2,TAX1BP1,TCF12,TDRKH,TLE3,TLN1,TNS3,TPD52,TRIM28,TRIM3,TRIOBP,TRIP10,TRIP12,TRIP13,TST,TTC21B,UBAP2L,UBE2C,UBR7,UCKL1,USP24,USP47,USP8,USP9X,VAT1,VCP,VCPIP1,VPS11,VPS13A,VPS36,WDR12,WFS1,WIPF1,XRN1,ZMPSTE24	164
Cancer,Gastrointestinal Disease,Organismal Injury and Abnormalities	Colon cancer	0,000139			ACO2,ACSL4,ADD1,AGL,AK3,AKR1B1,AKR1B10,AKR7A2,ALDH2,ALDH6A1,ANXA4,APPL2,ARFGAP2,ARFGAP3,ARHGEF18,ATIC,ATP2A2,ATXN10,BCAS1,BCAT2,BIRC6,C1QBP,CACYBP,CANX,CAVIN1,CBX3,CDK4,CDK6,CEP55,CNPY2,CRAT,CSNK1A1,CTBP2,CTTNBP2,CYB5R1,DCTN6,DDX5,DNAJC5,DNMT1,DOCK5,DPY19L1,DYNC2H1,EEF1B2,ELOA,ELP1,EMC7,ERLIN2,EXOC2,EXOC4,EXTL3,FAF2,FASN,FLII,FLNA,FLNB,FRMD4A,GCLM,GHDC,GLRX3,GMPPB,GRB14,GSTZ1,GTF2B,GTSE1,HDGFL3,HEBP2,HM13,HMMR,HNRNPUL1,IMPA1,INPPL1,IRGQ,ISCA2,ITPR1,ITPRID2,KANK1,KPNA2,LARP7,LETM1,LIMD1,LOXL3,MEGF10,MON2,NAA25,NCLN,NEK9,NLE1,NQO1,NRDC,NT5C3B,NUP54,PAK1,PAPSS1,PCBP2,PDE4DIP,PDZRN3,PHLDA1,PIGU,PKN2,PLCB1,POLA1,POLD1,POLD3,POLDIP3,PPP2R5D,PPP4R3A,PRKACB,PRKCA,PRRC2A,PTPN1,RAD51,RAF1,RETSAT,RIOK3,RNASEH2B,RPA1,RRM1,RRM2,SAMD4B,SEC14L1,SEC24A,SEC24D,SLC1A4,SLC9A3R2,SNX1,SNX17,SOAT1,SPP1,SQLE,SQSTM1,STAT1,STAT3,STAT5B,TCF12,TLN1,TM9SF3,TM9SF4,TMEM115,TMEM43,TNS3,TRIM28,TRIM3,TRIM47,TRIOBP,TRIP12,TRIP13,UBAP2L,UCKL1,USP9X,VCP,VPS13A,VPS36,WDR45B,WDR62,WFS1,WIPF1,XRN1,ZMPSTE24	158
Cancer,Hematological Disease,Immunological Disease,Organismal Injury and Abnormalities	Relapsed acute leukemia	0,000139			DNMT1,IDH2,POLA1,POLD1,PPAT,RAF1,RPL3,RRM1,RRM2	9
Cancer,Hematological Disease,Immunological Disease,Organismal Injury and Abnormalities	CD5 positive CD23 negative cyclin D1 positive mantle cell lymphoma	0,000143			POLA1,RRM1,RRM2	3
Cancer,Hematological Disease,Immunological Disease,Organismal Injury and Abnormalities	Relapsed double hit type high grade B-cell lymphoma	0,000143			POLA1,RRM1,RRM2	3
Cancer,Hematological Disease,Immunological Disease,Organismal Injury and Abnormalities	Non-promyelocytic acute myeloid leukemia	0,000143			DNMT1,POLA1,POLD1	3
Cancer,Hematological Disease,Immunological Disease,Organismal Injury and Abnormalities	Refractory low-grade B-cell non-Hodgkin lymphoma	0,000143			POLA1,RRM1,RRM2	3
Cancer,Hematological Disease,Immunological Disease,Organismal Injury and Abnormalities	Stable disease stage CD20-positive B-cell non-Hodgkin lymphoma	0,000143			POLA1,RRM1,RRM2	3
Cell Death and Survival,Organismal Survival	Survival of Saccharomyces cerevisiae	0,000143			HMOX1,NDC80,RAD51	3
Cancer,Hematological Disease,Immunological Disease,Organismal Injury and Abnormalities	Relapsed refractory acute myeloid leukemia	0,000143			POLA1,RRM1,RRM2	3
Cancer,Hematological Disease,Immunological Disease,Organismal Injury and Abnormalities	Active stage refractory acute myeloid leukemia	0,000143			POLA1,RRM1,RRM2	3
Cancer,Hematological Disease,Immunological Disease,Organismal Injury and Abnormalities	Active stage relapsed acute myeloid leukemia	0,000143			POLA1,RRM1,RRM2	3
Cancer,Hematological Disease,Immunological Disease,Organismal Injury and Abnormalities	Pretreated chronic lymphocytic leukemia	0,000143			POLA1,RRM1,RRM2	3
Cancer,Hematological Disease,Immunological Disease,Organismal Injury and Abnormalities	Refractory double hit type high grade B-cell lymphoma	0,000143			POLA1,RRM1,RRM2	3
Nucleic Acid Metabolism,Small Molecule Biochemistry	Synthesis of NADPH	0,000143			ALDH2,FASN,PGD	3
Cancer,Hematological Disease,Immunological Disease,Organismal Injury and Abnormalities	Type M1 acute myeloid leukemia	0,000144			FLNA,IDH2,POLA1,POLD1,PPAT,RRM1,RRM2	7
Cancer,Neurological Disease,Organismal Injury and Abnormalities	Brain astrocytoma	0,000153			ABCD4,ACP2,ACSF2,ADD1,AGA,AGFG1,AKR1B10,ALDH2,ALDH9A1,ANAPC7,AP3S2,APPL2,ARFGAP2,ARFGAP3,ARFGEF2,ARHGEF1,ARHGEF18,ATIC,ATP2B4,ATP6AP1,ATP6V1A,ATXN10,BCAS1,BCAT2,BIRC6,CACYBP,CAMSAP2,CAND1,CANX,CARMIL1,CC2D1B,CCDC47,CDC42EP4,CDK6,CEP55,CNN2,CRAT,CTBP2,CTTNBP2,CUL3,CWF19L1,CYFIP1,DDB1,DDRGK1,DNMT1,DOCK1,DOCK5,DYNC2H1,EEF2K,EIF2B4,ELP1,ELP3,ERLIN2,EXOC4,FADD,FASN,FBXO7,FLII,FLNA,FLNB,GABPA,GCLC,GCLM,GDA,GFPT1,GMDS,GMPPB,GPT2,GRB14,GSDMD,HDGFL3,HEBP2,HMGCS1,HMOX1,IDH2,INPPL1,ITPR1,ITPR3,ITPRID2,KANK1,KATNB1,KPNA2,MARK2,MMUT,MON2,MPI,NAA25,NCLN,NFKB2,NIT2,NLE1,NLN,NRDC,NT5C3B,OGT,OXCT1,PAFAH1B1,PAPSS1,PCBP2,PCK2,PDE4DIP,PEPD,PFKM,PHLDA1,PIP4P2,PITRM1,PKN2,PLCB1,POLA1,POLD1,PPAT,PRRC2A,RAB32,RAF1,RANGAP1,RPA1,RPL5,RPN1,RRM1,RRM2,SDF4,SEC14L1,SEC24A,SEC24D,SH3GLB1,SNX1,SOAT1,SORBS3,SPCS3,SRGAP3,STARD3NL,STAT1,STAT5B,STRN4,TAX1BP1,TDRKH,TLN1,TM9SF4,TMEM43,TNS3,TOM1L2,TPD52,TRIM28,TRIOBP,TRIP10,TRIP12,UBR7,USP24,USP47,USP8,USP9X,VCP,VCPIP1,VWA5A,WDR62,WFS1,XRN1	157
Cancer,Gastrointestinal Disease,Organismal Injury and Abnormalities	Upper gastrointestinal tract cancer	0,000155			AARSD1,ACO1,ACSF2,ACSL3,ACSL4,AGL,ALDH2,ANKRD13A,APPL2,ARFGAP2,ARFGEF2,ARHGEF1,ARHGEF18,ASRGL1,ATIC,ATP2A2,ATP6V1A,BIRC6,C1QBP,CAMSAP2,CANX,CDK4,CDK6,CEP55,CRAT,CSNK1A1,CTBP2,CTTNBP2,CYFIP1,DDB1,DDX5,DNAJC9,DNMT1,DOCK1,DOCK5,DPYSL3,DYNC2H1,EEF2,EEF2K,EIF2B4,ELOA,ELP1,EMC7,EMD,EML1,EXOC2,EXTL3,FASN,FDFT1,FLNA,FLNB,FRMD4A,GCDH,GDA,GLRX5,GMPPB,GRB14,HDGFL3,HM13,HMMR,HNRNPUL1,HSPA4L,IDH2,INPPL1,ITPR1,ITPR3,ITPRID2,KANK2,LARP7,LAYN,LETM1,LIMD1,LOXL3,LPP,MARK2,MEGF10,MTDH,MTSS1,NAA25,NAB2,NCLN,NLE1,NQO1,NRBP1,NRDC,OAT,OXCT1,PAFAH1B1,PAK1,PAPSS1,PCK2,PDE4DIP,PEPD,PIGU,PKN2,PLCB1,POLA1,POLD1,POLD3,POLDIP3,PRKACB,PRKCA,PRRC2A,PRRX1,QSOX2,RAD51,RAF1,RBPJ,RNASEH2B,RPA1,RPL5,RRM1,RRM2,SDF4,SEC14L1,SERPINE2,SORBS3,SPP1,SQLE,SRGAP3,STAT1,STAT3,STAT5B,STT3A,SUCLG2,TAX1BP1,TDRKH,TLE3,TLN1,TNS3,TPD52,TRIM28,TRIM3,TRIOBP,TRIP10,TRIP12,TRIP13,TST,UBAP2L,UBE2C,UBR7,UCKL1,USP24,USP47,USP8,USP9X,VAT1,VCP,VCPIP1,VPS11,VPS13A,VPS36,WDR12,WFS1,WIPF1,XRN1,ZMPSTE24	157
Cancer,Hematological Disease,Immunological Disease,Organismal Injury and Abnormalities	Stage I-IV diffuse large B-cell lymphoma	0,000157			DNMT1,POLA1,POLD1,RRM1,RRM2	5
Cancer,Cardiovascular Disease,Hematological Disease,Immunological Disease,Organismal Injury and Abnormalities	Intravascular large B-cell lymphoma	0,000157			DNMT1,POLA1,POLD1,RRM1,RRM2	5
Cancer,Organismal Injury and Abnormalities	KRAS codon 13 mutation positive cancer	0,000157			CDK4,CDK6,POLA1,POLD1,RAF1	5
Cancer,Gastrointestinal Disease,Organismal Injury and Abnormalities	Tumorigenesis of gastrointestinal tumor	0,00016			ACO2,ACSL4,ADD1,AGL,AK3,AKR1B1,AKR1B10,AKR7A2,ALDH2,ALDH6A1,ANXA4,APPL2,ARFGAP2,ARFGAP3,ARHGEF18,ARMC8,ATIC,ATP2A2,ATXN10,BCAT2,BIRC6,C1QBP,CACYBP,CANX,CAVIN1,CBX3,CDK4,CDK6,CEP55,CNPY2,CRAT,CSNK1A1,CTBP2,CTTNBP2,CYB5R1,DCTN6,DDX5,DGKZ,DNAJC5,DNMT1,DOCK1,DOCK5,DPY19L1,DYNC2H1,EEF1B2,ELOA,ELP1,EMC7,ERLIN2,EXOC2,EXOC4,EXTL3,FAF2,FASN,FLII,FLNA,FLNB,FRMD4A,GCLM,GHDC,GMPPB,GRB14,GSTZ1,GTF2B,GTSE1,HDGFL3,HEBP2,HM13,HMMR,HNRNPUL1,IDH2,IMPA1,INPPL1,IRGQ,ISCA2,ITPR1,ITPR3,ITPRID2,KANK1,KPNA2,LARP7,LIMD1,LOXL3,MEGF10,MON2,NAA25,NCLN,NEK9,NLE1,NQO1,NRDC,NT5C3B,NUP54,PAK1,PCBP2,PDE4DIP,PDZRN3,PHLDA1,PIGU,PKN2,PLCB1,POLA1,POLD1,POLD3,POLDIP3,PPP2R5D,PPP4R3A,PRKACB,PRKCA,PRRC2A,PTPN1,RAF1,RETSAT,RIOK3,RNASEH2B,RPA1,RRM1,SAMD4B,SEC14L1,SEC24A,SEC24D,SERPINE2,SLC1A4,SLC9A3R2,SNX1,SNX17,SOAT1,SQLE,SQSTM1,STAT1,STAT3,STAT5B,TAX1BP1,TCF12,TLN1,TM9SF3,TM9SF4,TMEM115,TMEM43,TNS3,TRIM28,TRIM3,TRIM47,TRIOBP,TRIP12,TRIP13,UBAP2L,UBE2C,UCKL1,USP24,USP9X,VCP,VPS13A,VPS36,WDR45B,WDR62,WFS1,WIPF1,XRN1,ZMPSTE24	160
DNA Replication, Recombination, and Repair	Excision repair	0,000164			DDB1,PARG,POLD1,POLD3,PRPF19,RAD51,RNASEH2B,RPA1,RPA2,USP47	10
Cancer,Gastrointestinal Disease,Organismal Injury and Abnormalities	Colorectal carcinoma	0,000177			ACO2,ACSL4,ADD1,AGL,AK3,AKR1B1,AKR1B10,AKR7A2,ALDH2,ALDH6A1,ANXA4,APPL2,ARFGAP2,ARFGAP3,ARHGEF18,ARMC8,ATIC,ATXN10,BCAT2,BIRC6,C1QBP,CACYBP,CANX,CAVIN1,CBX3,CDK4,CDK6,CEP55,CNPY2,CRAT,CSNK1A1,CTBP2,CTTNBP2,CYB5R1,DCTN6,DDX5,DGKZ,DNAJC5,DNMT1,DOCK1,DOCK5,DPY19L1,DYNC2H1,EEF1B2,ELOA,ELP1,EMC7,ERLIN2,EXOC2,EXOC4,EXTL3,FAF2,FASN,FLII,FLNA,FLNB,FRMD4A,GCLM,GHDC,GMPPB,GRB14,GSTZ1,GTF2B,GTSE1,HDGFL3,HEBP2,HM13,HMMR,HNRNPUL1,IDH2,IMPA1,INPPL1,IRGQ,ISCA2,ITPR1,ITPR3,ITPRID2,KANK1,KPNA2,LARP7,LIMD1,LOXL3,MEGF10,MON2,NAA25,NCLN,NEK9,NLE1,NQO1,NRDC,NT5C3B,NUP54,PAK1,PCBP2,PDE4DIP,PDZRN3,PHLDA1,PIGU,PKN2,PLCB1,POLA1,POLD1,POLD3,POLDIP3,PPP2R5D,PPP4R3A,PRKACB,PRKCA,PRRC2A,PTPN1,RAF1,RETSAT,RIOK3,RNASEH2B,RPA1,RRM1,SAMD4B,SEC14L1,SEC24A,SEC24D,SERPINE2,SLC1A4,SLC9A3R2,SNX1,SNX17,SOAT1,SQLE,SQSTM1,STAT1,STAT3,STAT5B,TAX1BP1,TCF12,TLN1,TM9SF3,TM9SF4,TMEM115,TMEM43,TNS3,TRIM28,TRIM3,TRIM47,TRIOBP,TRIP12,TRIP13,UBAP2L,UBE2C,UCKL1,USP24,USP9X,VCP,VPS13A,VPS36,WDR45B,WDR62,WFS1,WIPF1,XRN1,ZMPSTE24	159
DNA Replication, Recombination, and Repair	Excision repair of DNA	0,000179			DDB1,PARG,POLD1,POLD3,PRPF19,RAD51,RPA1,RPA2,USP47	9

Categories	Diseases or Functions Annotation	p-value	Predicted Activation State	Activation z-score	Molecules	# Molecules
Cell Death and Survival,Cell Morphology,Cellular Function and Maintenance	Autophagic cell death	0,000183			ACP2,FADD,MTSS1,SH3GLB1,SQSTM1	5
Cancer,Organismal Injury and Abnormalities,Tumor Morphology	Progressive malignant lymphatic system neoplasm	0,000183			ATIC,POLA1,POLD1,RRM1,RRM2	5
Cancer,Organismal Injury and Abnormalities	HER2 negative cancer	0,000193			CDK1,CDK4,CDK6,CERT1,DPYSL2,MARK2,NQO1,POLA1,POLD1,PRKCA,RRM1,RRM2,SPP1,STAT1	14
Cancer,Organismal Injury and Abnormalities	HER2 negative solid tumor	0,0002			CDK1,CDK4,CDK6,CERT1,DPYSL2,MARK2,NQO1,POLA1,POLD1,PRKCA,RRM1,RRM2,SPP1,STAT1	14
Organismal Development	Abnormal morphology of body cavity	0,000209			ACSL4,AGA,AGL,AKR1B1,AKR1B10,Anp32b,ARHGEF1,ARHGEF12,ATP2A2,BCAT2,CDK4,CDK6,DDB1,DNMT1,DOCK1,EEF2K,EIF2B5,ELOA,ELP1,EMD,FADD,FASN,FDFT1,FLNA,FUT8,GLRX3,GLRX5,GSTZ1,HMGCL,HMOX1,HSPA4L,LOXL3,MARK2,MMUT,MTSS1,NFKB2,NQO1,PAK1,PFKM,PLCB1,POLD1,PRDX2,PRKCA,PRRX1,PTPN1,RAF1,RBPJ,RRM1,RRM2,SERPINE2,SGTA,SLC9A3R1,SOAT1,SPP1,STAT1,STAT3,STAT5B,TAX1BP1,TLN1,TMEM43,WIPF1,ZMPSTE24	62
Cancer,Hematological Disease,Immunological Disease,Organismal Injury and Abnormalities	BCL2 rearrangement positive BCL6 rearrangement positive MYC rearrangement positive B-cell lymphoma	0,000212			DNMT1,POLA1,POLD1,RRM1,RRM2	5
Cancer,Neurological Disease,Organismal Injury and Abnormalities	Grade 4 glioblastoma	0,000212			DOCK1,IDH2,RAF1,SLC9A3R1,UBE2C	5
Cancer,Hematological Disease,Immunological Disease,Organismal Injury and Abnormalities	Diffuse large B-cell lymphoma associated with chronic inflammation	0,000212			DNMT1,POLA1,POLD1,RRM1,RRM2	5
Cancer,Gastrointestinal Disease,Organismal Injury and Abnormalities	Colorectal cancer	0,000212			ACADS,ACO2,ACSL4,ADD1,AGL,AK3,AKR1B1,AKR1B10,AKR7A2,ALDH2,ALDH6A1,ANXA4,APPL2,ARFGAP2,ARFGAP3,ARHGEF18,ARMC8,ATIC,ATP2A2,ATXN10,BCAS1,BCAT2,BIRC6,C1QBP,CACYBP,CANX,CAVIN1,CBX3,CDK4,CDK6,CEP55,CNPY2,CRAT,CSNK1A1,CTBP2,CTTNBP2,CYB5R1,DCTN6,DDX5,DGKZ,DNAJC5,DNMT1,DOCK1,DOCK5,DPY19L1,DPYSL2,DYNC2H1,EEF1B2,ELOA,ELP1,EMC7,ERLIN2,EXOC2,EXOC4,EXTL3,FAF2,FASN,FLII,FLNA,FLNB,FRMD4A,GCDH,GCLM,GHDC,GLRX,GLRX3,GMPPB,GRB14,GSTZ1,GTF2B,GTSE1,HDGFL3,HEBP2,HM13,HMMR,HMOX1,HNRNPUL1,IDH2,IMPA1,INPPL1,IRGQ,ISCA2,ITPR1,ITPR3,ITPRID2,KANK1,KPNA2,LARP7,LETM1,LIMD1,LOXL3,MAT2A,MEGF10,MON2,NAA25,NCLN,NEK9,NLE1,NQO1,NRDC,NT5C3B,NUP54,PAK1,PAPSS1,PCBP2,PDE4DIP,PDZRN3,PHLDA1,PIGU,PKN2,PLCB1,POLA1,POLD1,POLD3,POLDIP3,PPP2R5D,PPP4R3A,PRKACB,PRKCA,PRRC2A,PRRX1,PTPN1,RAD51,RAF1,RETSAT,RIOK3,RNASEH2B,RPA1,RRM1,RRM2,SAMD4B,SEC14L1,SEC24A,SEC24D,SERPINE2,SLC1A4,SLC9A3R2,SNX1,SNX17,SOAT1,SPP1,SQLE,SQSTM1,STAT1,STAT3,STAT5B,TAGLN2,TAX1BP1,TCF12,TLN1,TM9SF3,TM9SF4,TMEM115,TMEM43,TNS3,TRIM28,TRIM3,TRIM47,TRIOBP,TRIP12,TRIP13,UBAP2L,UBE2C,UCKL1,USP24,USP9X,VCP,VPS13A,VPS36,WDR45B,WDR62,WFS1,WIPF1,XRN1,ZMPSTE24	175
Hematological Disease	Failure of bone marrow	0,000219			ALDH2,NQO1,POLA1,RAD51,RPL27,RPL5,RRM1,RRM2	8
Hematological Disease,Metabolic Disease,Organismal Injury and Abnormalities	Acidemia	0,000222			ALDH4A1,BCAT2,GLRX5,MMUT	4
Cell-To-Cell Signaling and Interaction,Cellular Assembly and Organization	Quantity of actin stress fibers	0,000222			FLNA,FLNB,PAK1,RAF1,SLC9A3R2,SPP1,STAT3	7
Cardiovascular Disease,Developmental Disorder,Hematological Disease,Organismal Injury and Abnormalities	Congenital aplastic anemia	0,000222			NQO1,POLA1,RAD51,RPL27,RPL5,RRM1,RRM2	7
Cancer,Hematological Disease,Immunological Disease,Organismal Injury and Abnormalities	CCND1 non-overexpressing IGH/CCND1 negative mantle cell lymphoma	0,000227			POLA1,RRM1,RRM2	3
Cardiovascular Disease,Organismal Injury and Abnormalities,Tissue Morphology	Degeneration of blood vessel	0,000227			AKR1B1,HMOX1,SPP1	3
Post-Translational Modification	O-GlcNAcylation of protein	0,000227			ITPR1,NQO1,OGT	3
Cancer,Hematological Disease,Immunological Disease,Organismal Injury and Abnormalities	CCND1 negative CD5 positive CD23 positive small lymphocytic lymphoma	0,000227			POLA1,RRM1,RRM2	3
Cancer,Hematological Disease,Immunological Disease,Organismal Injury and Abnormalities	Philadelphia chromosome negative t(8;21) mutation negative acute myeloid leukemia	0,000227			DNMT1,POLA1,POLD1	3
Nucleic Acid Metabolism,Small Molecule Biochemistry	Biosynthesis of GDP	0,000227			GMDS,GMPPB,MPI	3
Cancer,Hematological Disease,Organismal Injury and Abnormalities	Bone marrow cancer	0,000228			ACSL3,ACSL4,ALDH4A1,ALDH6A1,ATP2B4,ATP6AP1,ATP6V1A,CDK1,CRAT,CSNK1A1,CTBP2,DGLUCY,DNAJC5,DNMT1,DYNC2H1,EML1,ERLIN2,EXTL3,FBXO7,FLII,FLNA,GMDS,IDH2,IFT74,ITPR1,ITPR3,KANK2,LAMTOR2,LOXL3,LPP,NQO1,NUP54,PDE4DIP,PIGU,POLA1,POLD1,PPAT,PRKACB,PRKCA,PTPN1,RAD51,RAF1,RAI14,RPL13,RPL3,RPL5,RPL6,RRM1,RRM2,SQSTM1,STAT3,STAT5B,TCF12,TLE3,TLN1,TRIP12,TRIP13,UBE2C,UBE2I,USP9X,VAMP7,WDR62,XRN1	63
Cancer,Hematological Disease,Immunological Disease,Organismal Injury and Abnormalities	Adult leukemia	0,000229			CSNK1A1,IDH2,NQO1,POLA1,POLD1,PPAT,RRM1,RRM2,STAT3	9
Cancer,Organismal Injury and Abnormalities	KRAS mutation negative cancer	0,000241			ATIC,POLA1,POLD1,RAF1,RRM1,RRM2	6
Amino Acid Metabolism,Small Molecule Biochemistry	Metabolism of glutamine family amino acid	0,000241			ALDH4A1,DGLUCY,GCLC,GCLM,NIT2,PPAT	6
Cancer,Hematological Disease,Immunological Disease,Organismal Injury and Abnormalities	Stage II-IV T-cell non-Hodgkin lymphoma	0,000244			POLA1,POLD1,PPAT,RRM1,RRM2	5
Cancer,Cardiovascular Disease,Developmental Disorder,Hematological Disease,Hereditary Disorder,Organismal Injury and Abnormalities	Diamond-Blackfan anemia	0,000244			POLA1,RPL27,RPL5,RRM1,RRM2	5
Gastrointestinal Disease,Organismal Injury and Abnormalities	Colorectal lesion	0,00025			ACADS,ACO2,ACSL4,ADD1,AGL,AK3,AKR1B1,AKR1B10,AKR7A2,ALDH2,ALDH6A1,ANXA4,APPL2,ARFGAP2,ARFGAP3,ARHGEF18,ARMC8,ATIC,ATP2A2,ATXN10,BCAS1,BCAT2,BIRC6,C1QBP,CACYBP,CANX,CAVIN1,CBX3,CDK1,CDK4,CDK6,CEP55,CNPY2,CRAT,CSNK1A1,CTBP2,CTTNBP2,CUL3,CYB5R1,DCTN6,DDX5,DGKZ,DNAJC5,DNMT1,DOCK1,DOCK5,DPY19L1,DPYSL2,DYNC2H1,EEF1B2,ELOA,ELP1,EMC7,ERLIN2,EXOC2,EXOC4,EXTL3,FAF2,FASN,FLII,FLNA,FLNB,FRMD4A,GCDH,GCLM,GHDC,GLRX,GLRX3,GMPPB,GRB14,GSTZ1,GTF2B,GTSE1,HDGFL3,HEBP2,HM13,HMMR,HMOX1,HNRNPUL1,IDH2,IMPA1,INPPL1,IRGQ,ISCA2,ITPR1,ITPR3,ITPRID2,KANK1,KPNA2,LARP7,LETM1,LIMD1,LOXL3,MAT2A,MEGF10,MON2,NAA25,NCLN,NEK9,NLE1,NQO1,NRDC,NT5C3B,NUP54,PAK1,PAPSS1,PCBP2,PDE4DIP,PDZRN3,PHLDA1,PIGU,PKN2,PLCB1,POLA1,POLD1,POLD3,POLDIP3,PPP2R5D,PPP4R3A,PRKACB,PRKCA,PRRC2A,PRRX1,PTPN1,RAD51,RAF1,RETSAT,RIOK3,RNASEH2B,RPA1,RRM1,RRM2,SAMD4B,SEC14L1,SEC24A,SEC24D,SERPINE2,SLC1A4,SLC9A3R2,SNX1,SNX17,SOAT1,SPP1,SQLE,SQSTM1,STAT1,STAT3,STAT5B,TAGLN2,TAX1BP1,TCF12,TLN1,TM9SF3,TM9SF4,TMEM115,TMEM43,TNS3,TRIM28,TRIM3,TRIM47,TRIOBP,TRIP12,TRIP13,UBAP2L,UBE2C,UCKL1,USP24,USP9X,VCP,VPS13A,VPS36,WDR45B,WDR62,WFS1,WIPF1,XRN1,ZMPSTE24	177
Cancer,Organismal Injury and Abnormalities,Reproductive System Disease	Uterine carcinoma	0,000257			ABCD4,ACSL3,ACSL4,ADD1,AK3,ALDH2,ALDH4A1,AP3S2,APPL2,ARFGEF2,ARHGEF12,ARHGEF18,ASRGL1,ATIC,ATP6AP1,ATP6V1A,BCAS1,BIRC6,CAMSAP2,CAND1,CAVIN1,CBX3,CDIPT,CDK4,CEP55,CTTNBP2,DDB1,DGKZ,DGLUCY,DNMT1,DOCK1,DOCK5,DPYSL2,DTYMK,DYNC2H1,EEF1B2,EEF2,ELP3,EMC7,EMD,EXOC2,EXOC4,FAF2,FBXO7,FDFT1,FLII,FLNA,FLNB,FRMD4A,FUT8,GATM,GFPT1,GLRX3,GPT2,HAUS7,HMMR,HSPA4L,IDH2,IFT74,IKBIP,INPPL1,ITPR1,ITPR3,KPNA2,LARP7,LETM1,LPP,LRRC40,MEGF10,MKLN1,MMUT,MON2,MTDH,MTSS1,NAPA,NFKB2,NQO1,NRDC,NUP54,OAT,OGT,OSBPL8,OXCT1,PARG,PCBP2,PCK2,PDE4DIP,PDZRN3,PEPD,PFKM,PHYH,PIGU,PITRM1,PKN2,PLCB1,POLA1,POLD1,POLD3,POLDIP3,PPAT,PPOX,PRKCA,PRPF19,PRRC2A,PTPN1,RAF1,RANGAP1,RER1,RETSAT,RIOK3,RPL5,RRM1,RRM2,SAMD4B,SDF4,SEC14L1,SEC61A1,SNF8,SNX17,SOAT1,SORBS3,STAT1,STAT3,STT3A,TAX1BP1,TCF12,TKFC,TLN1,TMEM43,TOM1L2,TRIM28,TRIM3,TRIOBP,TRIP12,TRIP13,TRMT2A,UBE2C,USP24,USP8,USP9X,VPS11,VPS13A,WIPF1,XRN1,ZMPSTE24	145
Developmental Disorder,Hereditary Disorder,Neurological Disease,Organismal Injury and Abnormalities	Familial neurodevelopmental disorder	0,000258			CUL3,DYNC2H1,ELP1,EXTL3,GOT2,GPT2,NARS1,NCLN,PIGU,PLCB1,RAF1,RPL6,SLC1A4,TRAPPC4,TRIP12,UBR7,USP9X,VARS1,WDR45B,WDR62	20
Connective Tissue Disorders,Developmental Disorder,Organismal Injury and Abnormalities,Skeletal and Muscular Disorders	Microcephaly	0,000265			ARFGEF2,ATPAF2,CDK6,FLNA,FRMD4A,IFT74,KATNB1,NARS1,POLA1,PRRX1,RBPJ,SLC1A4,STT3A,TRAPPC4,VARS1,WDR62	16
Cancer,Endocrine System Disorders,Gastrointestinal Disease,Organismal Injury and Abnormalities	Unresectable locally advanced pancreatic cancer	0,000265			RRM1,RRM2	2
Energy Production,Small Molecule Biochemistry	Oxidation of heme	0,000265			HMOX1,HMOX2	2
Cancer,Hematological Disease,Immunological Disease,Organismal Injury and Abnormalities	Relapsed enteropathy-associated T-cell lymphoma	0,000265			RRM1,RRM2	2
Cancer,Organismal Injury and Abnormalities,Reproductive System Disease	Stage 3 fallopian tube carcinoma	0,000265			RRM1,RRM2	2
Cancer,Endocrine System Disorders,Organismal Injury and Abnormalities,Reproductive System Disease	Advanced hormone receptor positive HER2 negative metastatic breast cancer	0,000265			CDK4,CDK6	2
Small Molecule Biochemistry	Conversion of heme	0,000265			HMOX1,HMOX2	2
Cancer,Endocrine System Disorders,Gastrointestinal Disease,Organismal Injury and Abnormalities	Localized pancreatic cancer	0,000265			RRM1,RRM2	2
Cancer,Endocrine System Disorders,Gastrointestinal Disease,Organismal Injury and Abnormalities	Stage 3 locally advanced pancreatic adenocarcinoma	0,000265			RRM1,RRM2	2
Cancer,Organismal Injury and Abnormalities	Stage IV primary peritoneal carcinoma	0,000265			RRM1,RRM2	2
Cancer,Gastrointestinal Disease,Hematological Disease,Hepatic System Disease,Immunological Disease,Organismal Injury and Abnormalities	Relapsed hepatosplenic T-cell lymphoma	0,000265			RRM1,RRM2	2
Cancer,Hematological Disease,Immunological Disease,Organismal Injury and Abnormalities	Refractory enteropathy-associated T-cell lymphoma	0,000265			RRM1,RRM2	2
Cellular Movement	Initiation of migration of leukemia cell lines	0,000265			FLNA,FLNB	2
Cell Morphology	Ruffling of cell periphery	0,000265			FLNA,FLNB	2
Cancer,Gastrointestinal Disease,Hepatic System Disease,Organismal Injury and Abnormalities	Resectable extrahepatic cholangiocarcinoma	0,000265			RRM1,RRM2	2
Small Molecule Biochemistry	Cleavage of heme	0,000265			HMOX1,HMOX2	2
Cancer,Organismal Injury and Abnormalities,Reproductive System Disease	Metastatic BRCA1 mutation negative BRCA2 mutation negative breast cancer	0,000265			RRM1,RRM2	2
Cancer,Endocrine System Disorders,Gastrointestinal Disease,Organismal Injury and Abnormalities	Poorly differentiated pancreatic carcinoma	0,000265			RRM1,RRM2	2
Cancer,Gastrointestinal Disease,Hepatic System Disease,Organismal Injury and Abnormalities	Resectable gallbladder cancer	0,000265			RRM1,RRM2	2
Cancer,Organismal Injury and Abnormalities,Reproductive System Disease	Ipsilateral breast cancer	0,000265			CDK4,CDK6	2
Cancer,Organismal Injury and Abnormalities,Reproductive System Disease	Stage IV fallopian tube carcinoma	0,000265			RRM1,RRM2	2
Cancer,Connective Tissue Disorders,Organismal Injury and Abnormalities	CDK4 amplification positive well differentiated/dedifferentiated liposarcoma	0,000265			CDK4,CDK6	2
Cancer,Organismal Injury and Abnormalities,Reproductive System Disease	TNM stage cT2 primary breast cancer	0,000265			CDK4,CDK6	2
Small Molecule Biochemistry	Catabolism of cyanate	0,000265			MPST,TST	2
Connective Tissue Disorders,Developmental Disorder,Hereditary Disorder,Neurological Disease,Organismal Injury and Abnormalities,Skeletal and Muscular Disorders	Autosomal recessive periventricular heterotopia with microcephaly	0,000265			ARFGEF2,FLNA	2
Hematological Disease,Immunological Disease,Organismal Injury and Abnormalities,Tissue Development	Chemotherapy-induced myelosuppression	0,000265			CDK4,CDK6	2
Cancer,Endocrine System Disorders,Gastrointestinal Disease,Organismal Injury and Abnormalities	Stage II advanced pancreatic adenocarcinoma	0,000265			RRM1,RRM2	2

Categories	Diseases or Functions Annotation	p-value	Predicted Activation State	Activation z-score	Molecules	# Molecules
Cancer,Organismal Injury and Abnormalities,Reproductive System Disease	Locally recurrent HER2 negative hormone receptor negative breast cancer	0,000265			RRM1,RRM2	2
Cellular Assembly and Organization,Cellular Function and Maintenance,Molecular Transport	Exocytosis of dense core granules	0,000265			DNAJC5,NAPA	2
Cancer,Endocrine System Disorders,Gastrointestinal Disease,Organismal Injury and Abnormalities	Stage III advanced pancreatic adenocarcinoma	0,000265			RRM1,RRM2	2
Cancer,Organismal Injury and Abnormalities,Reproductive System Disease	Stage 2-3 multicentric invasive breast cancer	0,000265			CDK4,CDK6	2
Cancer,Organismal Injury and Abnormalities,Reproductive System Disease	Stage II-III bilateral invasive breast cancer	0,000265			CDK4,CDK6	2
Cancer,Organismal Injury and Abnormalities,Reproductive System Disease	TNM stage cT3 primary breast cancer	0,000265			CDK4,CDK6	2
Cancer,Gastrointestinal Disease,Hematological Disease,Hepatic System Disease,Immunological Disease,Organismal Injury and Abnormalities	Refractory hepatosplenic T-cell lymphoma	0,000265			RRM1,RRM2	2
Cancer,Organismal Injury and Abnormalities	Stage III primary peritoneal carcinoma	0,000265			RRM1,RRM2	2
Cancer,Endocrine System Disorders,Gastrointestinal Disease,Organismal Injury and Abnormalities	Metastatic adenosquamous pancreatic carcinoma	0,000265			RRM1,RRM2	2
Cancer,Organismal Injury and Abnormalities,Reproductive System Disease	Stage II-III multicentric HER2 negative hormone receptor positive invasive breast cancer	0,000265			CDK4,CDK6	2
Cell Cycle,Renal and Urological System Development and Function	Initiation of S phase of kidney cell lines	0,000265			CDK4,CDK6	2
Lipid Metabolism,Nucleic Acid Metabolism,Small Molecule Biochemistry	Conversion of malonyl-coenzyme A	0,000265			ACACA,FASN	2
Cancer,Organismal Injury and Abnormalities,Reproductive System Disease	Stage II-III multifocal HER2 negative hormone receptor positive invasive breast cancer	0,000265			CDK4,CDK6	2
Cancer,Organismal Injury and Abnormalities	Stage II primary peritoneal carcinoma	0,000265			RRM1,RRM2	2
Carbohydrate Metabolism,Nucleic Acid Metabolism,Small Molecule Biochemistry	Synthesis of nucleoside diphosphate sugar	0,000274			GFPT1,GMDS,GMPPB,MPI	4
Cancer,Gastrointestinal Disease,Organismal Injury and Abnormalities	Metastatic KRAS-mutation-positive colorectal cancer	0,000279			CDK4,CDK6,POLA1,POLD1,RAF1	5
Cancer,Hematological Disease,Immunological Disease,Organismal Injury and Abnormalities	Acute myeloid leukemia in remission	0,000279			DNMT1,POLA1,POLD1,RRM1,RRM2	5
Cancer,Hematological Disease,Immunological Disease,Organismal Injury and Abnormalities	T-cell acute lymphoblastic lymphoma	0,000295			DNMT1,POLA1,POLD1,PPAT,RPL3,RRM1	6
Cancer,Hematological Disease,Immunological Disease,Organismal Injury and Abnormalities	Acute myeloid leukemia	0,000311			ACSL3,ACSL4,ALDH4A1,ALDH6A1,ATP2B4,ATP6AP1,ATP6V1A,CDK1,CSNK1A1,CTBP2,DGLUCY,DNAJC5,DNMT1,DYNC2H1,EML1,ERLIN2,EXTL3,FBXO7,FLII,FLNA,GMDS,IDH2,IFT74,ITPR1,ITPR3,KANK2,LAMTOR2,LOXL3,LPP,NQO1,NUP54,PDE4DIP,POLA1,POLD1,PPAT,PRKCA,PTPN1,RAD51,RAF1,RAI14,RPL3,RPL6,RRM1,RRM2,SQSTM1,STAT3,STAT5B,TCF12,TLN1,TRIP12,TRIP13,UBE2C,UBE2I,USP9X,VAMP7,WDR62,XRN1	57
Hematological Disease,Immunological Disease	Abnormal neutrophil count	0,000324			DNMT1,NQO1,POLA1,POLD1,PPAT,PRKCA,RRM1,RRM2,SPP1,STAT3	10
Cancer,Organismal Injury and Abnormalities	Cancer of head	0,000329			ABCD4,ACADS,ACSL4,ADD1,AIP,AKR1B1,AKR1B10,ALDH9A1,ARHGEF1,ARPIN/ARPIN-AP3S2,ASRGL1,ATIC,ATP2A2,ATP6V1A,ATPAF2,BIRC6,CAMSAP2,CAND1,CARMIL1,CDK4,CDK6,CSNK1A1,CTBP2,CTTNBP2,CUL3,DDX5,DNAJC9,DNMT1,DNPEP,DOCK5,DPY19L1,DYNC2H1,EEF2K,ELP2,EMD,EXOC4,FASN,FBXL15,FBXO7,FDFT1,FLII,FLNA,FLNB,FUT8,GCDH,GDA,GLRX5,GMDS,GMPPB,GPT2,HEBP2,HM13,HMMR,HMOX1,IDH2,ITPR1,ITPR3,ITPRID2,KANK2,LETM1,LRRC40,MARK2,MEGF10,MKLN1,MTDH,MTSS1,NFKB2,NLE1,NRBP1,NRDC,NT5C3B,OAT,PAFAH1B1,PCK2,PDE4DIP,PHYH,PLCB1,POLA1,POLD1,PPAT,PRKACB,PRRC2A,QSOX2,RAB32,RAF1,RASSF4,RPN2,RRM1,RRM2,SAMD4B,SEC14L1,SORBS3,SPP1,STAT1,STAT3,STAT5B,STRN4,TAX1BP1,TCF12,TLE3,TLN1,TM9SF4,TNS3,TRIM2,TRIP10,TRIP12,UBAP2L,UBE2O,UBR7,USP24,USP8,VPS36,WDR62,WIPF1,XRN1	115
Neurological Disease,Skeletal and Muscular Disorders	Progressive neuromuscular disease	0,000331			BCAS1,FBXO7,FDFT1,GSDMD,HMOX2,LOXL3,MMUT,PAK1,PIP4P2,POLA1,PPAT,PRDX2,PRRC2A,RPL13,RPL3,RPL5,RRM1,RRM2,SLC1A4,SPP1,SQLE,TRAPPC4,USP24	23
Cancer,Hematological Disease,Immunological Disease,Organismal Injury and Abnormalities	Acute lymphoblastic lymphoma	0,000332			DNMT1,POLA1,POLD1,PPAT,RPL3,RRM1,RRM2	7
Cancer,Hematological Disease,Immunological Disease,Organismal Injury and Abnormalities	Newly diagnosed de novo acute myeloid leukemia	0,000335			DNMT1,POLA1,POLD1,PRKCA	4
Cancer,Hematological Disease,Immunological Disease,Organismal Injury and Abnormalities	Refractory FLT3 internal tandem duplication positive acute myeloid leukemia	0,000335			POLA1,POLD1,RRM1,RRM2	4
Cancer,Hematological Disease,Immunological Disease,Organismal Injury and Abnormalities	Refractory classical hairy cell leukemia	0,000335			POLA1,RAF1,RRM1,RRM2	4
Cancer,Organismal Injury and Abnormalities,Reproductive System Disease	Locally recurrent HER2-negative breast cancer	0,000335			CDK4,CDK6,RRM1,RRM2	4
Cancer,Hematological Disease,Immunological Disease,Organismal Injury and Abnormalities	De novo intermediate-risk acute myeloid leukemia	0,000335			DNMT1,POLA1,RRM1,RRM2	4
Cancer,Hematological Disease,Immunological Disease,Organismal Injury and Abnormalities	17p deletion negative small B cell lymphocytic lymphoma	0,000336			POLA1,RRM1,RRM2	3
Connective Tissue Disorders,Developmental Disorder,Hereditary Disorder,Metabolic Disease,Organismal Injury and Abnormalities,Skeletal and Muscular Disorders	Familial Paget disease of bone	0,000336			NFKB2,SQSTM1,VCP	3
Cancer,Endocrine System Disorders,Gastrointestinal Disease,Organismal Injury and Abnormalities	Invasive pancreatic adenocarcinoma	0,000336			RRM1,RRM2,SPP1	3
Cancer,Hematological Disease,Immunological Disease,Organismal Injury and Abnormalities	Refractory childhood acute lymphoblastic leukemia	0,000336			POLA1,POLD1,RRM1	3
Cancer,Hematological Disease,Organismal Injury and Abnormalities	Myeloid neoplasm	0,000346			ACSL3,ACSL4,ALDH4A1,ALDH6A1,ATP2B4,ATP6AP1,ATP6V1A,CDK1,CRAT,CSNK1A1,CTBP2,DGLUCY,DNAJC5,DNMT1,DYNC2H1,EEF2,EML1,ERLIN2,EXTL3,FBXO7,FLII,FLNA,GMDS,IDH2,IFT74,ITPR1,ITPR3,KANK2,LAMTOR2,LOXL3,LPP,NQO1,NUP54,PDE4DIP,PIGU,POLA1,POLD1,PPAT,PRKACB,PRKCA,PTPN1,RAD51,RAF1,RAI14,RPL13,RPL3,RPL5,RPL6,RRM1,RRM2,SPP1,SQSTM1,STAT3,STAT5B,TCF12,TLE3,TLN1,TRIP12,TRIP13,UBE2C,UBE2I,USP9X,VAMP7,WDR62,XRN1	65
Cardiovascular Disease,Hematological Disease,Organismal Injury and Abnormalities	Aplastic anemia	0,000349			ALDH2,NQO1,POLA1,RAD51,RPL27,RPL5,RRM1,RRM2	8
Cancer,Hematological Disease,Immunological Disease,Organismal Injury and Abnormalities	Acute myeloid leukemia with recurrent genetic abnormalities	0,000349			DNMT1,IDH2,POLA1,POLD1,PPAT,RAF1,RRM1,RRM2	8
Cancer,Organismal Injury and Abnormalities,Reproductive System Disease	Uterine endometrioid carcinoma	0,000349			ABCD4,ACSL3,ACSL4,ADD1,AK3,ALDH2,ALDH4A1,AP3S2,APPL2,ARFGEF2,ARHGEF12,ARHGEF18,ASRGL1,ATIC,ATP6AP1,BIRC6,CAMSAP2,CAND1,CBX3,CDIPT,CEP55,CTTNBP2,DDB1,DGKZ,DGLUCY,DNMT1,DOCK1,DOCK5,DPYSL2,DTYMK,DYNC2H1,EEF1B2,EEF2,ELP3,EMC7,EMD,EXOC2,EXOC4,FAF2,FBXO7,FDFT1,FLII,FLNA,FLNB,FRMD4A,FUT8,GATM,GFPT1,GLRX3,GPT2,HAUS7,HMMR,HSPA4L,IDH2,IFT74,IKBIP,INPPL1,ITPR1,ITPR3,LARP7,LETM1,LRRC40,MEGF10,MKLN1,MMUT,MON2,MTDH,MTSS1,NAPA,NFKB2,NQO1,NRDC,NUP54,OAT,OGT,OSBPL8,OXCT1,PARG,PCBP2,PCK2,PDE4DIP,PEPD,PFKM,PHYH,PIGU,PITRM1,PKN2,PLCB1,POLA1,POLD1,POLD3,POLDIP3,PPAT,PPOX,PRKCA,PRPF19,PRRC2A,PTPN1,RAF1,RANGAP1,RER1,RETSAT,RIOK3,RPL5,SAMD4B,SEC14L1,SEC61A1,SNF8,SNX17,SOAT1,SORBS3,STAT1,STAT3,STT3A,TAX1BP1,TCF12,TKFC,TLN1,TMEM43,TOM1L2,TRIM28,TRIM3,TRIOBP,TRIP12,TRMT2A,USP24,USP8,USP9X,VPS11,VPS13A,WIPF1,XRN1,ZMPSTE24	133
Cancer,Organismal Injury and Abnormalities,Reproductive System Disease	Endometrial carcinoma	0,000358			ABCD4,ACSL3,ACSL4,ADD1,AK3,ALDH2,ALDH4A1,AP3S2,APPL2,ARFGEF2,ARHGEF12,ARHGEF18,ASRGL1,ATIC,ATP6AP1,BIRC6,CAMSAP2,CAND1,CAVIN1,CBX3,CDIPT,CDK4,CEP55,CTTNBP2,DDB1,DGKZ,DGLUCY,DNMT1,DOCK1,DOCK5,DPYSL2,DTYMK,DYNC2H1,EEF1B2,EEF2,ELP3,EMC7,EMD,EXOC2,EXOC4,FAF2,FBXO7,FDFT1,FLII,FLNA,FLNB,FRMD4A,FUT8,GATM,GFPT1,GLRX3,GPT2,HAUS7,HMMR,HSPA4L,IDH2,IFT74,IKBIP,INPPL1,ITPR1,ITPR3,LARP7,LETM1,LRRC40,MEGF10,MKLN1,MMUT,MON2,MTDH,MTSS1,NAPA,NFKB2,NQO1,NRDC,NUP54,OAT,OGT,OSBPL8,OXCT1,PARG,PCBP2,PCK2,PDE4DIP,PEPD,PFKM,PHYH,PIGU,PITRM1,PKN2,PLCB1,POLA1,POLD1,POLD3,POLDIP3,PPAT,PPOX,PRKCA,PRPF19,PRRC2A,PTPN1,RAF1,RANGAP1,RER1,RETSAT,RIOK3,RPL5,SAMD4B,SEC14L1,SEC61A1,SNF8,SNX17,SOAT1,SORBS3,STAT1,STAT3,STT3A,TAX1BP1,TCF12,TKFC,TLN1,TMEM43,TOM1L2,TRIM28,TRIM3,TRIOBP,TRIP12,TRMT2A,USP24,USP8,USP9X,VPS11,VPS13A,WIPF1,XRN1,ZMPSTE24	135
Cancer,Hematological Disease,Hereditary Disorder,Organismal Injury and Abnormalities	Constitutional bone marrow failure syndrome	0,000358			NQO1,POLA1,RAD51,RPL27,RPL5,RRM1,RRM2	7
Cancer,Gastrointestinal Disease,Organismal Injury and Abnormalities	Colorectal tumor	0,000359			ACADS,ACO2,ACSL4,ADD1,AGL,AK3,AKR1B1,AKR1B10,AKR7A2,ALDH2,ALDH6A1,ANXA4,APPL2,ARFGAP2,ARFGAP3,ARHGEF18,ARMC8,ATIC,ATP2A2,ATXN10,BCAS1,BCAT2,BIRC6,C1QBP,CACYBP,CANX,CAVIN1,CBX3,CDK1,CDK4,CDK6,CEP55,CNPY2,CRAT,CSNK1A1,CTBP2,CTTNBP2,CYB5R1,DCTN6,DDX5,DGKZ,DNAJC5,DNMT1,DOCK1,DOCK5,DPY19L1,DPYSL2,DYNC2H1,EEF1B2,ELOA,ELP1,EMC7,ERLIN2,EXOC2,EXOC4,EXTL3,FAF2,FASN,FLII,FLNA,FLNB,FRMD4A,GCDH,GCLM,GHDC,GLRX,GLRX3,GMPPB,GRB14,GSTZ1,GTF2B,GTSE1,HDGFL3,HEBP2,HM13,HMMR,HMOX1,HNRNPUL1,IDH2,IMPA1,INPPL1,IRGQ,ISCA2,ITPR1,ITPR3,ITPRID2,KANK1,KPNA2,LARP7,LETM1,LIMD1,LOXL3,MAT2A,MEGF10,MON2,NAA25,NCLN,NEK9,NLE1,NQO1,NRDC,NT5C3B,NUP54,PAK1,PAPSS1,PCBP2,PDE4DIP,PDZRN3,PHLDA1,PIGU,PKN2,PLCB1,POLA1,POLD1,POLD3,POLDIP3,PPP2R5D,PPP4R3A,PRKACB,PRKCA,PRRC2A,PRRX1,PTPN1,RAD51,RAF1,RETSAT,RIOK3,RNASEH2B,RPA1,RRM1,RRM2,SAMD4B,SEC14L1,SEC24A,SEC24D,SERPINE2,SLC1A4,SLC9A3R2,SNX1,SNX17,SOAT1,SPP1,SQLE,SQSTM1,STAT1,STAT3,STAT5B,TAGLN2,TAX1BP1,TCF12,TLN1,TM9SF3,TM9SF4,TMEM115,TMEM43,TNS3,TRIM28,TRIM3,TRIM47,TRIOBP,TRIP12,TRIP13,UBAP2L,UBE2C,UCKL1,USP24,USP9X,VCP,VPS13A,VPS36,WDR45B,WDR62,WFS1,WIPF1,XRN1,ZMPSTE24	176
Cancer,Organismal Injury and Abnormalities,Reproductive System Disease	Prostatic adenocarcinoma	0,000365			AARSD1,ACADS,ACSF2,ACSL3,ACSL4,AKR1B1,ALDH2,ALDH4A1,ANKRD13A,ARFGAP2,ARFGAP3,ARFGEF2,ARHGEF1,ATP2B4,BIRC6,CARMIL1,CBX3,CC2D1B,CDC42EP4,CDK4,CDK6,CRAT,CTTNBP2,CUL3,DDB1,DGKZ,DNMT1,DOCK5,DYNC2H1,EEF1B2,EEF2,EEF2K,ELP1,ELP2,EML1,EXOC4,FASN,FLNB,GDA,GHDC,GOT2,GSDMD,HNRNPUL1,HSPA4L,INPPL1,ITPR1,ITPR3,KANK1,LARP7,LAYN,MKLN1,MMUT,MON2,MPST,NAB2,NAPA,NCLN,NLE1,NPTXR,OGT,PAK1,PAPSS1,PCK2,PDE4DIP,PHLDA1,PIP4P2,PITRM1,PKN2,POLA1,POLD1,PRKACB,PTPN1,RAD51,RANGAP1,RETSAT,RIOK3,RPA1,RPA2,SAMD4B,SEC14L1,SEC24D,SERPINE2,SGTA,SH3GLB1,SLC1A4,SQSTM1,STAT1,STAT3,STAT5B,TNS3,TOM1L2,TRAPPC4,TRIM28,TRIOBP,TRIP12,UBAP2L,UBE2O,USP24,USP47,USP9X,VPS13A,VPS36,VWA5A,WDR12,WIPF1,XRN1	106
Cancer,Hematological Disease,Immunological Disease,Organismal Injury and Abnormalities	Mature B-cell lymphoma	0,000383			ANXA4,ARHGEF1,BIRC6,CDK1,CDK6,CTBP2,DDB1,DNMT1,ECI2,EXOC4,FADD,FASN,GMPPB,GTF2B,HMOX1,IKBIP,ITPR1,KPNA2,LPP,PDZRN3,POLA1,POLD1,PRKCA,PTPN1,RAF1,RRM1,RRM2,SNX1,STAT3	29
Cancer,Hematological Disease,Immunological Disease,Organismal Injury and Abnormalities	B-cell lymphoma	0,000385			ANXA4,ARHGEF1,BIRC6,CARMIL1,CDK1,CDK6,CTBP2,DDB1,DNMT1,DOCK1,ECI2,EXOC4,FADD,FASN,GMPPB,GTF2B,HMMR,HMOX1,IKBIP,ITPR1,ITPR3,KPNA2,LPP,NDC80,NFKB2,PDZRN3,POLA1,POLD1,PPAT,PRKCA,PTPN1,RAD51,RAF1,RPA1,RRM1,RRM2,SEC14L1,SNX1,STAT3,STAT5B,TRIP12,TRIP13,TTC21B,UBE2C,USP8	45
Cancer,Organismal Injury and Abnormalities	Malignant connective or soft tissue neoplasm	0,000388			AARSD1,ABCD4,ACAA2,ACO1,ACO2,ACP2,ACSF2,ACSL3,ACSL4,ADD1,ADI1,AGA,AGFG1,AGL,AKR1B1,AKR1B10,ALDH2,ALDH4A1,ALDH6A1,ALDH9A1,ANAPC7,ANKRD13A,ANKRD46,ANXA4,APPL2,ARFGAP2,ARFGAP3,ARFGEF2,ARHGEF1,ARHGEF12,ARHGEF18,ARPIN/ARPIN-AP3S2,ASRGL1,ATIC,ATP2A2,ATP6AP1,ATP6V1A,ATPAF2,BCAS1,BCAT2,BIRC6,CAMSAP2,CAND1,CANX,CARMIL1,CAVIN1,CCZ1/CCZ1B,CDC37L1,CDK1,CDK4,CDK6,CEP55,CNEP1R1,CNN2,CNPY2,CRAT,CSNK1A1,CSRP1,CTBP2,CTTNBP2,CUL3,DDB1,DDRGK1,DGKZ,DGLUCY,DNAJC5,DNMT1,DNPEP,DOCK1,DOCK5,DPY19L1,DPYSL2,DPYSL3,DYNC2H1,ECI2,EEF1B2,EEF2,EEF2K,EIF2B4,ELOA,ELP3,EML1,ERLIN2,EXOC2,EXTL3,FADD,FASN,FDFT1,FLII,FLNA,FLNB,FRMD4A,GATM,GCDH,GCLC,GDA,GHDC,GMDS,GMPPB,GOT2,GPT2,GRB14,GTSE1,HAUS7,HDGFL3,HMGCL,HMGCS1,HMOX1,HMOX2,HNRNPUL1,IDH2,IKBIP,INPPL1,IRGQ,ITPR1,ITPR3,ITPRID2,KANK2,KATNB1,KPNA2,LAYN,LETM1,LIMD1,LPP,LTA4H,MARK2,MAT2A,MEGF10,MKLN1,MMUT,MON2,MPI,MPST,MTSS1,NAA25,NAB2,NAPA,NAXE,NCLN,NEK9,NFKB2,NFS1,NPTXR,NRDC,OAT,OSBPL8,PAPSS1,PCBP2,PDE4DIP,PDZRN3,PEPD,PGD,PHLDA1,PHYH,PIGU,PITRM1,PKN2,PLCB1,POLA1,POLD1,POLDIP3,PPP4R3A,PRKACB,PRKCA,PRRC2A,PRRX1,QSOX2,RAB32,RAD51,RAF1,RAI14,RASSF4,RBPJ,RIOK3,RPA1,RPL13,RPL3,RPL5,RPN1,RPN2,RRM1,RRM2,SAMD4B,SEC14L1,SEC24A,SEC24D,SERPINE2,SFR1,SLC1A4,SPP1,SQLE,SRGAP3,STAT1,STAT3,STAT5B,STT3A,TCF12,TDRKH,TKFC,TLE3,TLN1,TM9SF4,TMEM43,TNS3,TOM1L2,TRIM2,TRIM28,TRIM3,TRIM47,TRIOBP,TRIP12,TRIP13,TRMT2A,TST,TTC21B,UBAP2L,UBE2C,UBE2O,UBR7,USP24,USP47,USP8,USP9X,VAMP7,VAT1,VCP,VCPIP1,VPS11,VPS13A,VPS4B,VWA5A,WDR12,WDR45B,WDR62,WFS1,XRN1	236
Cancer,Hematological Disease,Immunological Disease,Organismal Injury and Abnormalities	Early precursor T-cell acute lymphoblastic leukemia	0,000393			DNMT1,IDH2,POLA1,POLD1,PPAT,RPL3	6

Categories	Diseases or Functions Annotation	p-value	Predicted Activation State	Activation z-score	Molecules	# Molecules
Cancer,Organismal Injury and Abnormalities,Reproductive System Disease	Stage IIIA-IV breast cancer	0,000393			CDK4,CDK6,DPYSL2,FDFT1,RRM1,RRM2	6
Cell Signaling,Cellular Function and Maintenance,Small Molecule Biochemistry,Vitamin and Mineral Metabolism	Calcium homeostasis of endoplasmic reticulum	0,000404			ATP2A2,CCDC47,ITPR1,WFS1	4
Cancer,Hematological Disease,Immunological Disease,Organismal Injury and Abnormalities	Refractory primary mediastinal large B-cell lymphoma	0,000404			POLA1,POLD1,RRM1,RRM2	4
Cancer,Hematological Disease,Organismal Injury and Abnormalities	Therapy-related myelodysplastic syndrome	0,000404			POLA1,POLD1,RRM1,RRM2	4
Cancer,Hematological Disease,Immunological Disease,Organismal Injury and Abnormalities	Relapsed primary mediastinal large B-cell lymphoma	0,000404			POLA1,POLD1,RRM1,RRM2	4
Neurological Disease,Organismal Injury and Abnormalities	Cognitive impairment	0,000405			ACSL4,AGA,ALDH4A1,ARFGEF2,ATP6V1A,CDC42EP4,CERT1,CUL3,CWF19L1,CYFIP1,DYNC2H1,EEF1B2,ELP2,ERLIN2,FASN,FLNA,GATM,GMPPB,GPT2,IMPA1,ITPR1,LARP7,NARS1,OGT,PAFAH1B1,PAK1,PLCB1,POLA1,POLD1,PPP2R5D,SLC1A4,SRGAP3,STAT1,STRN4,TRIP12,UBR7,USP9X,VCP,WDR45B,WDR62,WFS1	41
Cancer,Hematological Disease,Immunological Disease,Organismal Injury and Abnormalities	Advanced aggressive non-Hodgkin lymphoma	0,00041			DNMT1,POLA1,POLD1,RRM1,RRM2	5
