# Supplementary figures and images for: Fetal Zone Steroids Show Discrete Effects on Hyperoxia-Induced Attenuation of Migration in Cultured Oligodendrocyte Progenitor Cells
Source: Oxid Med Cell Longev. 2022 May 9;2022:2606880. doi: 10.1155/2022/2606880 (PMC9110221; doi:10.1155/2022/2606880)

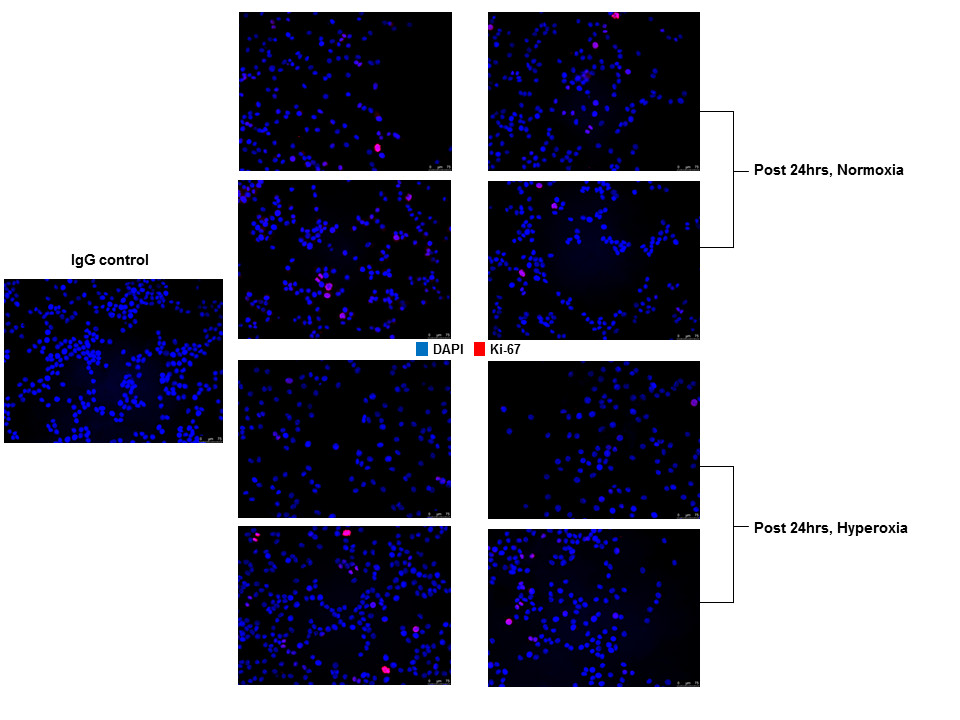

Supplement: Supplementary Materials — Figure S1 (supplementary figure 1): Ki67 staining of OLN93 cells post-24 hours of normoxic and hyperoxic (80% O2) treatments. Representative immunofluorescence images of OLN93 cells stained for Ki67 proliferation marker. Upper panel represents images taken post-24 hours of normoxic treatment. Lower panel represents images taken post-24 hours of hyperoxic treatment. Scale bar represents 75 μm. Data are representative of three individual experiments. Figure S2 (supplementary figure 2): changes in specific migration-related proteins post treatments. Intensities of (a) Hmox1, (b) PAK1, (c) RAF1, and (d) Cdc42ep4 plotted from the mass spectrometry results. Graphs show the changes in protein intensities upon different treatment conditions. Data are representative of five independent experiments. Bars and error represent mean ± SEM of replicate measurements. ∗ represents statistically significant differences in comparison to normoxic control, # represents statistically significant differences in comparison to hyperoxic control, and § represents statistically significant differences between normoxic and hyperoxic treatments within the same group. Single signs represent a p value < 0.05, double signs represent p < 0.01, triple signs represent p < 0.001, and quadruple signs represent p < 0.0001(Student's t-test). Figure S3 (supplementary figure 3): complete heat map of canonical pathway analysis of significantly enriched proteins in the OLN93 cells post cotreatment of adiol+E2 in comparison to normoxic and hyperoxic controls using IPA. Negative z-score values are indicated in blue, and positive z-score values are indicated in red. Cutoff p value < 0.05 (Fisher's exact test). Table T1 (supplementary table 1): details of mass spectrometry procedure. (a) LC-MS/MS parameter (data independent mode; quantitative data). (b) Spectronaut parameters for peptide/protein identification and intensity extraction. Table T2 (supplementary table 2): functional categorization of proteins with [file 2606880.f1.zip › Figure S1.jpg]

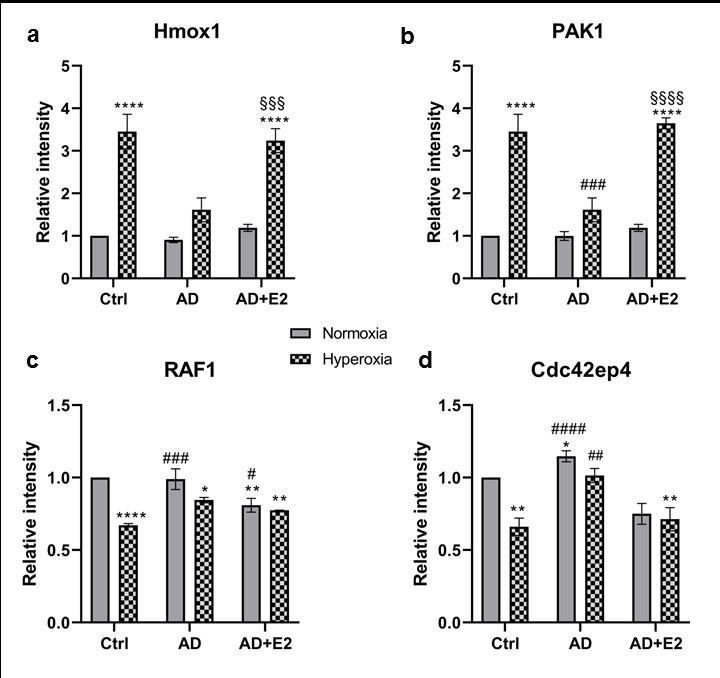

Supplement: Supplementary Materials — Figure S1 (supplementary figure 1): Ki67 staining of OLN93 cells post-24 hours of normoxic and hyperoxic (80% O2) treatments. Representative immunofluorescence images of OLN93 cells stained for Ki67 proliferation marker. Upper panel represents images taken post-24 hours of normoxic treatment. Lower panel represents images taken post-24 hours of hyperoxic treatment. Scale bar represents 75 μm. Data are representative of three individual experiments. Figure S2 (supplementary figure 2): changes in specific migration-related proteins post treatments. Intensities of (a) Hmox1, (b) PAK1, (c) RAF1, and (d) Cdc42ep4 plotted from the mass spectrometry results. Graphs show the changes in protein intensities upon different treatment conditions. Data are representative of five independent experiments. Bars and error represent mean ± SEM of replicate measurements. ∗ represents statistically significant differences in comparison to normoxic control, # represents statistically significant differences in comparison to hyperoxic control, and § represents statistically significant differences between normoxic and hyperoxic treatments within the same group. Single signs represent a p value < 0.05, double signs represent p < 0.01, triple signs represent p < 0.001, and quadruple signs represent p < 0.0001(Student's t-test). Figure S3 (supplementary figure 3): complete heat map of canonical pathway analysis of significantly enriched proteins in the OLN93 cells post cotreatment of adiol+E2 in comparison to normoxic and hyperoxic controls using IPA. Negative z-score values are indicated in blue, and positive z-score values are indicated in red. Cutoff p value < 0.05 (Fisher's exact test). Table T1 (supplementary table 1): details of mass spectrometry procedure. (a) LC-MS/MS parameter (data independent mode; quantitative data). (b) Spectronaut parameters for peptide/protein identification and intensity extraction. Table T2 (supplementary table 2): functional categorization of proteins with [file 2606880.f1.zip › Figure S2.jpg]
